# Jet energy measurement and its systematic uncertainty in proton–proton collisions at $\sqrt{s}=7$ TeV with the ATLAS detector

**DOI:** 10.1140/epjc/s10052-014-3190-y

**Published:** 2015-01-15

**Authors:** Atlas Collaboration, G. Aad, T. Abajyan, B. Abbott, J. Abdallah, S. Abdel Khalek, O. Abdinov, R. Aben, B. Abi, M. Abolins, O. S. AbouZeid, H. Abramowicz, H. Abreu, Y. Abulaiti, B. S. Acharya, L. Adamczyk, D. L. Adams, T. N. Addy, J. Adelman, S. Adomeit, T. Adye, S. Aefsky, T. Agatonovic-Jovin, J. A. Aguilar-Saavedra, M. Agustoni, S. P. Ahlen, A. Ahmad, F. Ahmadov, G. Aielli, T. P. A. Åkesson, G. Akimoto, A. V. Akimov, M. A. Alam, J. Albert, S. Albrand, M. J. Alconada Verzini, M. Aleksa, I. N. Aleksandrov, F. Alessandria, C. Alexa, G. Alexander, G. Alexandre, T. Alexopoulos, M. Alhroob, M. Aliev, G. Alimonti, L. Alio, J. Alison, B. M. M. Allbrooke, L. J. Allison, P. P. Allport, S. E. Allwood-Spiers, J. Almond, A. Aloisio, R. Alon, A. Alonso, F. Alonso, A. Altheimer, B. Alvarez Gonzalez, M. G. Alviggi, K. Amako, Y. Amaral Coutinho, C. Amelung, V. V. Ammosov, S. P. Amor Dos Santos, A. Amorim, S. Amoroso, N. Amram, G. Amundsen, C. Anastopoulos, L. S. Ancu, N. Andari, T. Andeen, C. F. Anders, G. Anders, K. J. Anderson, A. Andreazza, V. Andrei, X. S. Anduaga, S. Angelidakis, P. Anger, A. Angerami, F. Anghinolfi, A. V. Anisenkov, N. Anjos, A. Annovi, A. Antonaki, M. Antonelli, A. Antonov, J. Antos, F. Anulli, M. Aoki, L. Aperio Bella, R. Apolle, G. Arabidze, I. Aracena, Y. Arai, A. T. H. Arce, S. Arfaoui, J-F. Arguin, S. Argyropoulos, E. Arik, M. Arik, A. J. Armbruster, O. Arnaez, V. Arnal, O. Arslan, A. Artamonov, G. Artoni, S. Asai, N. Asbah, S. Ask, B. Åsman, L. Asquith, K. Assamagan, R. Astalos, A. Astbury, M. Atkinson, N. B. Atlay, B. Auerbach, E. Auge, K. Augsten, M. Aurousseau, G. Avolio, G. Azuelos, Y. Azuma, M. A. Baak, C. Bacci, A. M. Bach, H. Bachacou, K. Bachas, M. Backes, M. Backhaus, J. Backus Mayes, E. Badescu, P. Bagiacchi, P. Bagnaia, Y. Bai, D. C. Bailey, T. Bain, J. T. Baines, O. K. Baker, S. Baker, P. Balek, F. Balli, E. Banas, Sw. Banerjee, D. Banfi, A. Bangert, V. Bansal, H. S. Bansil, L. Barak, S. P. Baranov, T. Barber, E. L. Barberio, D. Barberis, M. Barbero, T. Barillari, M. Barisonzi, T. Barklow, N. Barlow, B. M. Barnett, R. M. Barnett, A. Baroncelli, G. Barone, A. J. Barr, F. Barreiro, J. Barreiro Guimarães da Costa, R. Bartoldus, A. E. Barton, P. Bartos, V. Bartsch, A. Bassalat, A. Basye, R. L. Bates, L. Batkova, J. R. Batley, M. Battistin, F. Bauer, H. S. Bawa, T. Beau, P. H. Beauchemin, R. Beccherle, P. Bechtle, H. P. Beck, K. Becker, S. Becker, M. Beckingham, A. J. Beddall, A. Beddall, S. Bedikian, V. A. Bednyakov, C. P. Bee, L. J. Beemster, T. A. Beermann, M. Begel, K. Behr, C. Belanger-Champagne, P. J. Bell, W. H. Bell, G. Bella, L. Bellagamba, A. Bellerive, M. Bellomo, A. Belloni, O. L. Beloborodova, K. Belotskiy, O. Beltramello, O. Benary, D. Benchekroun, K. Bendtz, N. Benekos, Y. Benhammou, E. Benhar Noccioli, J. A. Benitez Garcia, D. P. Benjamin, J. R. Bensinger, K. Benslama, S. Bentvelsen, D. Berge, E. Bergeaas Kuutmann, N. Berger, F. Berghaus, E. Berglund, J. Beringer, C. Bernard, P. Bernat, R. Bernhard, C. Bernius, F. U. Bernlochner, T. Berry, P. Berta, C. Bertella, F. Bertolucci, M. I. Besana, G. J. Besjes, O. Bessidskaia, N. Besson, S. Bethke, W. Bhimji, R. M. Bianchi, L. Bianchini, M. Bianco, O. Biebel, S. P. Bieniek, K. Bierwagen, J. Biesiada, M. Biglietti, J. Bilbao De Mendizabal, H. Bilokon, M. Bindi, S. Binet, A. Bingul, C. Bini, B. Bittner, C. W. Black, J. E. Black, K. M. Black, D. Blackburn, R. E. Blair, J.-B. Blanchard, T. Blazek, I. Bloch, C. Blocker, J. Blocki, W. Blum, U. Blumenschein, G. J. Bobbink, V. S. Bobrovnikov, S. S. Bocchetta, A. Bocci, C. R. Boddy, M. Boehler, J. Boek, T. T. Boek, N. Boelaert, J. A. Bogaerts, A. G. Bogdanchikov, A. Bogouch, C. Bohm, J. Bohm, V. Boisvert, T. Bold, V. Boldea, A. S. Boldyrev, N. M. Bolnet, M. Bomben, M. Bona, M. Boonekamp, S. Bordoni, C. Borer, A. Borisov, G. Borissov, M. Borri, S. Borroni, J. Bortfeldt, V. Bortolotto, K. Bos, D. Boscherini, M. Bosman, H. Boterenbrood, J. Bouchami, J. Boudreau, E. V. Bouhova-Thacker, D. Boumediene, C. Bourdarios, N. Bousson, S. Boutouil, A. Boveia, J. Boyd, I. R. Boyko, I. Bozovic-Jelisavcic, J. Bracinik, P. Branchini, A. Brandt, G. Brandt, O. Brandt, U. Bratzler, B. Brau, J. E. Brau, H. M. Braun, S. F. Brazzale, B. Brelier, K. Brendlinger, R. Brenner, S. Bressler, T. M. Bristow, D. Britton, F. M. Brochu, I. Brock, R. Brock, F. Broggi, C. Bromberg, J. Bronner, G. Brooijmans, T. Brooks, W. K. Brooks, J. Brosamer, E. Brost, G. Brown, J. Brown, P. A. Bruckman de Renstrom, D. Bruncko, R. Bruneliere, S. Brunet, A. Bruni, G. Bruni, M. Bruschi, L. Bryngemark, T. Buanes, Q. Buat, F. Bucci, P. Buchholz, R. M. Buckingham, A. G. Buckley, S. I. Buda, I. A. Budagov, B. Budick, F. Buehrer, L. Bugge, M. K. Bugge, O. Bulekov, A. C. Bundock, M. Bunse, H. Burckhart, S. Burdin, T. Burgess, B. Burghgrave, S. Burke, I. Burmeister, E. Busato, V. Büscher, P. Bussey, C. P. Buszello, B. Butler, J. M. Butler, A. I. Butt, C. M. Buttar, J. M. Butterworth, W. Buttinger, A. Buzatu, M. Byszewski, S. Cabrera Urbán, D. Caforio, O. Cakir, P. Calafiura, G. Calderini, P. Calfayan, R. Calkins, L. P. Caloba, R. Caloi, D. Calvet, S. Calvet, R. Camacho Toro, P. Camarri, D. Cameron, L. M. Caminada, R. Caminal Armadans, S. Campana, M. Campanelli, V. Canale, F. Canelli, A. Canepa, J. Cantero, R. Cantrill, T. Cao, M. D. M. Capeans Garrido, I. Caprini, M. Caprini, M. Capua, R. Caputo, R. Cardarelli, T. Carli, G. Carlino, L. Carminati, S. Caron, E. Carquin, G. D. Carrillo-Montoya, A. A. Carter, J. R. Carter, J. Carvalho, D. Casadei, M. P. Casado, C. Caso, E. Castaneda-Miranda, A. Castelli, V. Castillo Gimenez, N. F. Castro, P. Catastini, A. Catinaccio, J. R. Catmore, A. Cattai, G. Cattani, S. Caughron, V. Cavaliere, D. Cavalli, M. Cavalli-Sforza, V. Cavasinni, F. Ceradini, B. Cerio, K. Cerny, A. S. Cerqueira, A. Cerri, L. Cerrito, F. Cerutti, A. Cervelli, S. A. Cetin, A. Chafaq, D. Chakraborty, I. Chalupkova, K. Chan, P. Chang, B. Chapleau, J. D. Chapman, D. Charfeddine, D. G. Charlton, V. Chavda, C. A. Chavez Barajas, S. Cheatham, S. Chekanov, S. V. Chekulaev, G. A. Chelkov, M. A. Chelstowska, C. Chen, H. Chen, K. Chen, L. Chen, S. Chen, X. Chen, Y. Chen, Y. Cheng, A. Cheplakov, R. Cherkaoui El Moursli, V. Chernyatin, E. Cheu, L. Chevalier, V. Chiarella, G. Chiefari, J. T. Childers, A. Chilingarov, G. Chiodini, A. S. Chisholm, R. T. Chislett, A. Chitan, M. V. Chizhov, S. Chouridou, B. K. B. Chow, I. A. Christidi, D. Chromek-Burckhart, M. L. Chu, J. Chudoba, G. Ciapetti, A. K. Ciftci, R. Ciftci, D. Cinca, V. Cindro, A. Ciocio, M. Cirilli, P. Cirkovic, Z. H. Citron, M. Citterio, M. Ciubancan, A. Clark, P. J. Clark, R. N. Clarke, W. Cleland, J. C. Clemens, B. Clement, C. Clement, Y. Coadou, M. Cobal, A. Coccaro, J. Cochran, S. Coelli, L. Coffey, J. G. Cogan, J. Coggeshall, J. Colas, B. Cole, S. Cole, A. P. Colijn, C. Collins-Tooth, J. Collot, T. Colombo, G. Colon, G. Compostella, P. Conde Muiño, E. Coniavitis, M. C. Conidi, I. A. Connelly, S. M. Consonni, V. Consorti, S. Constantinescu, C. Conta, G. Conti, F. Conventi, M. Cooke, B. D. Cooper, A. M. Cooper-Sarkar, N. J. Cooper-Smith, K. Copic, T. Cornelissen, M. Corradi, F. Corriveau, A. Corso-Radu, A. Cortes-Gonzalez, G. Cortiana, G. Costa, M. J. Costa, D. Costanzo, D. Côté, G. Cottin, L. Courneyea, G. Cowan, B. E. Cox, K. Cranmer, G. Cree, S. Crépé-Renaudin, F. Crescioli, M. Crispin Ortuzar, M. Cristinziani, G. Crosetti, C.-M. Cuciuc, C. Cuenca Almenar, T. Cuhadar Donszelmann, J. Cummings, M. Curatolo, C. Cuthbert, H. Czirr, P. Czodrowski, Z. Czyczula, S. D’Auria, M. D’Onofrio, A. D’Orazio, M. J. Da Cunha Sargedas De Sousa, C. Da Via, W. Dabrowski, A. Dafinca, T. Dai, F. Dallaire, C. Dallapiccola, M. Dam, A. C. Daniells, M. Dano Hoffmann, V. Dao, G. Darbo, G. L. Darlea, S. Darmora, J. A. Dassoulas, W. Davey, C. David, T. Davidek, E. Davies, M. Davies, O. Davignon, A. R. Davison, Y. Davygora, E. Dawe, I. Dawson, R. K. Daya-Ishmukhametova, K. De, R. de Asmundis, S. De Castro, S. De Cecco, J. de Graat, N. De Groot, P. de Jong, C. De La Taille, H. De la Torre, F. De Lorenzi, L. De Nooij, D. De Pedis, A. De Salvo, U. De Sanctis, A. De Santo, J. B. De Vivie De Regie, G. De Zorzi, W. J. Dearnaley, R. Debbe, C. Debenedetti, B. Dechenaux, D. V. Dedovich, J. Degenhardt, J. Del Peso, T. Del Prete, T. Delemontex, F. Deliot, M. Deliyergiyev, A. Dell’Acqua, L. Dell’Asta, M. Della Pietra, D. della Volpe, M. Delmastro, P. A. Delsart, C. Deluca, S. Demers, M. Demichev, A. Demilly, B. Demirkoz, S. P. Denisov, D. Derendarz, J. E. Derkaoui, F. Derue, P. Dervan, K. Desch, P. O. Deviveiros, A. Dewhurst, B. DeWilde, S. Dhaliwal, R. Dhullipudi, A. Di Ciaccio, L. Di Ciaccio, A. Di Domenico, C. Di Donato, A. Di Girolamo, B. Di Girolamo, A. Di Mattia, B. Di Micco, R. Di Nardo, A. Di Simone, R. Di Sipio, D. Di Valentino, M. A. Diaz, E. B. Diehl, J. Dietrich, T. A. Dietzsch, S. Diglio, K. Dindar Yagci, J. Dingfelder, C. Dionisi, P. Dita, S. Dita, F. Dittus, F. Djama, T. Djobava, M. A. B. do Vale, A. Do Valle Wemans, T. K. O. Doan, D. Dobos, E. Dobson, J. Dodd, C. Doglioni, T. Doherty, T. Dohmae, J. Dolejsi, Z. Dolezal, B. A. Dolgoshein, M. Donadelli, S. Donati, P. Dondero, J. Donini, J. Dopke, A. Doria, A. Dos Anjos, A. Dotti, M. T. Dova, A. T. Doyle, M. Dris, J. Dubbert, S. Dube, E. Dubreuil, E. Duchovni, G. Duckeck, O. A. Ducu, D. Duda, A. Dudarev, F. Dudziak, L. Duflot, L. Duguid, M. Dührssen, M. Dunford, H. Duran Yildiz, M. Düren, M. Dwuznik, J. Ebke, W. Edson, C. A. Edwards, N. C. Edwards, W. Ehrenfeld, T. Eifert, G. Eigen, K. Einsweiler, E. Eisenhandler, T. Ekelof, M. El Kacimi, M. Ellert, S. Elles, F. Ellinghaus, K. Ellis, N. Ellis, J. Elmsheuser, M. Elsing, D. Emeliyanov, Y. Enari, O. C. Endner, M. Endo, R. Engelmann, J. Erdmann, A. Ereditato, D. Eriksson, G. Ernis, J. Ernst, M. Ernst, J. Ernwein, D. Errede, S. Errede, E. Ertel, M. Escalier, H. Esch, C. Escobar, X. Espinal Curull, B. Esposito, F. Etienne, A. I. Etienvre, E. Etzion, D. Evangelakou, H. Evans, L. Fabbri, G. Facini, R. M. Fakhrutdinov, S. Falciano, Y. Fang, M. Fanti, A. Farbin, A. Farilla, T. Farooque, S. Farrell, S. M. Farrington, P. Farthouat, F. Fassi, P. Fassnacht, D. Fassouliotis, B. Fatholahzadeh, A. Favareto, L. Fayard, P. Federic, O. L. Fedin, W. Fedorko, M. Fehling-Kaschek, L. Feligioni, C. Feng, E. J. Feng, H. Feng, A. B. Fenyuk, W. Fernando, S. Ferrag, J. Ferrando, V. Ferrara, A. Ferrari, P. Ferrari, R. Ferrari, D. E. Ferreira de Lima, A. Ferrer, D. Ferrere, C. Ferretti, A. Ferretto Parodi, M. Fiascaris, F. Fiedler, A. Filipčič, M. Filipuzzi, F. Filthaut, M. Fincke-Keeler, K. D. Finelli, M. C. N. Fiolhais, L. Fiorini, A. Firan, J. Fischer, M. J. Fisher, E. A. Fitzgerald, M. Flechl, I. Fleck, P. Fleischmann, S. Fleischmann, G. T. Fletcher, G. Fletcher, T. Flick, A. Floderus, L. R. Flores Castillo, A. C. Florez Bustos, M. J. Flowerdew, T. Fonseca Martin, A. Formica, A. Forti, D. Fortin, D. Fournier, H. Fox, P. Francavilla, M. Franchini, S. Franchino, D. Francis, M. Franklin, S. Franz, M. Fraternali, S. Fratina, S. T. French, C. Friedrich, F. Friedrich, D. Froidevaux, J. A. Frost, C. Fukunaga, E. Fullana Torregrosa, B. G. Fulsom, J. Fuster, C. Gabaldon, O. Gabizon, A. Gabrielli, A. Gabrielli, S. Gadatsch, T. Gadfort, S. Gadomski, G. Gagliardi, P. Gagnon, C. Galea, B. Galhardo, E. J. Gallas, V. Gallo, B. J. Gallop, P. Gallus, G. Galster, K. K. Gan, R. P. Gandrajula, J. Gao, Y. S. Gao, F. M. Garay Walls, F. Garberson, C. García, J. E. García Navarro, M. Garcia-Sciveres, R. W. Gardner, N. Garelli, V. Garonne, C. Gatti, G. Gaudio, B. Gaur, L. Gauthier, P. Gauzzi, I. L. Gavrilenko, C. Gay, G. Gaycken, E. N. Gazis, P. Ge, Z. Gecse, C. N. P. Gee, D. A. A. Geerts, Ch. Geich-Gimbel, K. Gellerstedt, C. Gemme, A. Gemmell, M. H. Genest, S. Gentile, M. George, S. George, D. Gerbaudo, A. Gershon, H. Ghazlane, N. Ghodbane, B. Giacobbe, S. Giagu, V. Giangiobbe, P. Giannetti, F. Gianotti, B. Gibbard, S. M. Gibson, M. Gilchriese, T. P. S. Gillam, D. Gillberg, A. R. Gillman, D. M. Gingrich, N. Giokaris, M. P. Giordani, R. Giordano, F. M. Giorgi, P. Giovannini, P. F. Giraud, D. Giugni, C. Giuliani, M. Giunta, B. K. Gjelsten, I. Gkialas, L. K. Gladilin, C. Glasman, J. Glatzer, A. Glazov, G. L. Glonti, M. Goblirsch-Kolb, J. R. Goddard, J. Godfrey, J. Godlewski, C. Goeringer, S. Goldfarb, T. Golling, D. Golubkov, A. Gomes, L. S. Gomez Fajardo, R. Gonçalo, J. Goncalves Pinto Firmino Da Costa, L. Gonella, S. González de la Hoz, G. Gonzalez Parra, M. L. Gonzalez Silva, S. Gonzalez-Sevilla, J. J. Goodson, L. Goossens, P. A. Gorbounov, H. A. Gordon, I. Gorelov, G. Gorfine, B. Gorini, E. Gorini, A. Gorišek, E. Gornicki, A. T. Goshaw, C. Gössling, M. I. Gostkin, M. Gouighri, D. Goujdami, M. P. Goulette, A. G. Goussiou, C. Goy, S. Gozpinar, H. M. X. Grabas, L. Graber, I. Grabowska-Bold, P. Grafström, K-J. Grahn, J. Gramling, E. Gramstad, F. Grancagnolo, S. Grancagnolo, V. Grassi, V. Gratchev, H. M. Gray, J. A. Gray, E. Graziani, O. G. Grebenyuk, Z. D. Greenwood, K. Gregersen, I. M. Gregor, P. Grenier, J. Griffiths, N. Grigalashvili, A. A. Grillo, K. Grimm, S. Grinstein, Ph. Gris, Y. V. Grishkevich, J.-F. Grivaz, J. P. Grohs, A. Grohsjean, E. Gross, J. Grosse-Knetter, G. C. Grossi, J. Groth-Jensen, Z. J. Grout, K. Grybel, F. Guescini, D. Guest, O. Gueta, C. Guicheney, E. Guido, T. Guillemin, S. Guindon, U. Gul, C. Gumpert, J. Gunther, J. Guo, S. Gupta, P. Gutierrez, N. G. Gutierrez Ortiz, C. Gutschow, N. Guttman, C. Guyot, C. Gwenlan, C. B. Gwilliam, A. Haas, C. Haber, H. K. Hadavand, P. Haefner, S. Hageboeck, Z. Hajduk, H. Hakobyan, M. Haleem, D. Hall, G. Halladjian, K. Hamacher, P. Hamal, K. Hamano, M. Hamer, A. Hamilton, S. Hamilton, L. Han, K. Hanagaki, K. Hanawa, M. Hance, P. Hanke, J. R. Hansen, J. B. Hansen, J. D. Hansen, P. H. Hansen, P. Hansson, K. Hara, A. S. Hard, T. Harenberg, S. Harkusha, D. Harper, R. D. Harrington, O. M. Harris, P. F. Harrison, F. Hartjes, A. Harvey, S. Hasegawa, Y. Hasegawa, S. Hassani, S. Haug, M. Hauschild, R. Hauser, M. Havranek, C. M. Hawkes, R. J. Hawkings, A. D. Hawkins, T. Hayashi, D. Hayden, C. P. Hays, H. S. Hayward, S. J. Haywood, S. J. Head, T. Heck, V. Hedberg, L. Heelan, S. Heim, B. Heinemann, S. Heisterkamp, J. Hejbal, L. Helary, C. Heller, M. Heller, S. Hellman, D. Hellmich, C. Helsens, J. Henderson, R. C. W. Henderson, A. Henrichs, A. M. Henriques Correia, S. Henrot-Versille, C. Hensel, G. H. Herbert, C. M. Hernandez, Y. Hernández Jiménez, R. Herrberg-Schubert, G. Herten, R. Hertenberger, L. Hervas, G. G. Hesketh, N. P. Hessey, R. Hickling, E. Higón-Rodriguez, J. C. Hill, K. H. Hiller, S. Hillert, S. J. Hillier, I. Hinchliffe, E. Hines, M. Hirose, D. Hirschbuehl, J. Hobbs, N. Hod, M. C. Hodgkinson, P. Hodgson, A. Hoecker, M. R. Hoeferkamp, J. Hoffman, D. Hoffmann, J. I. Hofmann, M. Hohlfeld, T. R. Holmes, T. M. Hong, L. Hooft van Huysduynen, J-Y. Hostachy, S. Hou, A. Hoummada, J. Howard, J. Howarth, M. Hrabovsky, I. Hristova, J. Hrivnac, T. Hryn’ova, P. J. Hsu, S.-C. Hsu, D. Hu, X. Hu, Y. Huang, Z. Hubacek, F. Hubaut, F. Huegging, A. Huettmann, T. B. Huffman, E. W. Hughes, G. Hughes, M. Huhtinen, T. A. Hülsing, M. Hurwitz, N. Huseynov, J. Huston, J. Huth, G. Iacobucci, G. Iakovidis, I. Ibragimov, L. Iconomidou-Fayard, J. Idarraga, E. Ideal, P. Iengo, O. Igonkina, T. Iizawa, Y. Ikegami, K. Ikematsu, M. Ikeno, D. Iliadis, N. Ilic, Y. Inamaru, T. Ince, P. Ioannou, M. Iodice, K. Iordanidou, V. Ippolito, A. Irles Quiles, C. Isaksson, M. Ishino, M. Ishitsuka, R. Ishmukhametov, C. Issever, S. Istin, A. V. Ivashin, W. Iwanski, H. Iwasaki, J. M. Izen, V. Izzo, B. Jackson, J. N. Jackson, M. Jackson, P. Jackson, M. R. Jaekel, V. Jain, K. Jakobs, S. Jakobsen, T. Jakoubek, J. Jakubek, D. O. Jamin, D. K. Jana, E. Jansen, H. Jansen, J. Janssen, M. Janus, R. C. Jared, G. Jarlskog, L. Jeanty, G.-Y. Jeng, I. Jen-La Plante, D. Jennens, P. Jenni, J. Jentzsch, C. Jeske, S. Jézéquel, M. K. Jha, H. Ji, W. Ji, J. Jia, Y. Jiang, M. Jimenez Belenguer, S. Jin, A. Jinaru, O. Jinnouchi, M. D. Joergensen, D. Joffe, K. E. Johansson, P. Johansson, K. A. Johns, K. Jon-And, G. Jones, R. W. L. Jones, T. J. Jones, P. M. Jorge, K. D. Joshi, J. Jovicevic, X. Ju, C. A. Jung, R. M. Jungst, P. Jussel, A. Juste Rozas, M. Kaci, A. Kaczmarska, P. Kadlecik, M. Kado, H. Kagan, M. Kagan, E. Kajomovitz, S. Kalinin, S. Kama, N. Kanaya, M. Kaneda, S. Kaneti, T. Kanno, V. A. Kantserov, J. Kanzaki, B. Kaplan, A. Kapliy, D. Kar, K. Karakostas, N. Karastathis, M. Karnevskiy, S. N. Karpov, K. Karthik, V. Kartvelishvili, A. N. Karyukhin, L. Kashif, G. Kasieczka, R. D. Kass, A. Kastanas, Y. Kataoka, A. Katre, J. Katzy, V. Kaushik, K. Kawagoe, T. Kawamoto, G. Kawamura, S. Kazama, V. F. Kazanin, M. Y. Kazarinov, R. Keeler, P. T. Keener, R. Kehoe, M. Keil, J. S. Keller, H. Keoshkerian, O. Kepka, B. P. Kerševan, S. Kersten, K. Kessoku, J. Keung, F. Khalil-zada, H. Khandanyan, A. Khanov, D. Kharchenko, A. Khodinov, A. Khomich, T. J. Khoo, G. Khoriauli, A. Khoroshilov, V. Khovanskiy, E. Khramov, J. Khubua, H. Kim, S. H. Kim, N. Kimura, O. Kind, B. T. King, M. King, R. S. B. King, S. B. King, J. Kirk, A. E. Kiryunin, T. Kishimoto, D. Kisielewska, T. Kitamura, T. Kittelmann, K. Kiuchi, E. Kladiva, M. Klein, U. Klein, K. Kleinknecht, P. Klimek, A. Klimentov, R. Klingenberg, J. A. Klinger, E. B. Klinkby, T. Klioutchnikova, P. F. Klok, E.-E. Kluge, P. Kluit, S. Kluth, E. Kneringer, E. B. F. G. Knoops, A. Knue, T. Kobayashi, M. Kobel, M. Kocian, P. Kodys, S. Koenig, P. Koevesarki, T. Koffas, E. Koffeman, L. A. Kogan, S. Kohlmann, Z. Kohout, T. Kohriki, T. Koi, H. Kolanoski, I. Koletsou, J. Koll, A. A. Komar, Y. Komori, T. Kondo, K. Köneke, A. C. König, T. Kono, R. Konoplich, N. Konstantinidis, R. Kopeliansky, S. Koperny, L. Köpke, A. K. Kopp, K. Korcyl, K. Kordas, A. Korn, A. A. Korol, I. Korolkov, E. V. Korolkova, V. A. Korotkov, O. Kortner, S. Kortner, V. V. Kostyukhin, S. Kotov, V. M. Kotov, A. Kotwal, C. Kourkoumelis, V. Kouskoura, A. Koutsman, R. Kowalewski, T. Z. Kowalski, W. Kozanecki, A. S. Kozhin, V. Kral, V. A. Kramarenko, G. Kramberger, M. W. Krasny, A. Krasznahorkay, J. K. Kraus, A. Kravchenko, S. Kreiss, J. Kretzschmar, K. Kreutzfeldt, N. Krieger, P. Krieger, K. Kroeninger, H. Kroha, J. Kroll, J. Kroseberg, J. Krstic, U. Kruchonak, H. Krüger, T. Kruker, N. Krumnack, Z. V. Krumshteyn, A. Kruse, M. C. Kruse, M. Kruskal, T. Kubota, S. Kuday, S. Kuehn, A. Kugel, T. Kuhl, V. Kukhtin, Y. Kulchitsky, S. Kuleshov, M. Kuna, J. Kunkle, A. Kupco, H. Kurashige, M. Kurata, Y. A. Kurochkin, R. Kurumida, V. Kus, E. S. Kuwertz, M. Kuze, J. Kvita, R. Kwee, A. La Rosa, L. La Rotonda, L. Labarga, S. Lablak, C. Lacasta, F. Lacava, J. Lacey, H. Lacker, D. Lacour, V. R. Lacuesta, E. Ladygin, R. Lafaye, B. Laforge, T. Lagouri, S. Lai, H. Laier, E. Laisne, L. Lambourne, C. L. Lampen, W. Lampl, E. Lançon, U. Landgraf, M. P. J. Landon, V. S. Lang, C. Lange, A. J. Lankford, F. Lanni, K. Lantzsch, A. Lanza, S. Laplace, C. Lapoire, J. F. Laporte, T. Lari, A. Larner, M. Lassnig, P. Laurelli, V. Lavorini, W. Lavrijsen, P. Laycock, B. T. Le, O. Le Dortz, E. Le Guirriec, E. Le Menedeu, T. LeCompte, F. Ledroit-Guillon, C. A. Lee, H. Lee, J. S. H. Lee, S. C. Lee, L. Lee, G. Lefebvre, M. Lefebvre, F. Legger, C. Leggett, A. Lehan, M. Lehmacher, G. Lehmann Miotto, A. G. Leister, M. A. L. Leite, R. Leitner, D. Lellouch, B. Lemmer, V. Lendermann, K. J. C. Leney, T. Lenz, G. Lenzen, B. Lenzi, R. Leone, K. Leonhardt, S. Leontsinis, C. Leroy, J-R. Lessard, C. G. Lester, C. M. Lester, J. Levêque, D. Levin, L. J. Levinson, A. Lewis, G. H. Lewis, A. M. Leyko, M. Leyton, B. Li, B. Li, H. Li, H. L. Li, S. Li, X. Li, Z. Liang, H. Liao, B. Liberti, P. Lichard, K. Lie, J. Liebal, W. Liebig, C. Limbach, A. Limosani, M. Limper, S. C. Lin, F. Linde, B. E. Lindquist, J. T. Linnemann, E. Lipeles, A. Lipniacka, M. Lisovyi, T. M. Liss, D. Lissauer, A. Lister, A. M. Litke, B. Liu, D. Liu, J. B. Liu, K. Liu, L. Liu, M. Liu, M. Liu, Y. Liu, M. Livan, S. S. A. Livermore, A. Lleres, J. Llorente Merino, S. L. Lloyd, F. Lo Sterzo, E. Lobodzinska, P. Loch, W. S. Lockman, T. Loddenkoetter, F. K. Loebinger, A. E. Loevschall-Jensen, A. Loginov, C. W. Loh, T. Lohse, K. Lohwasser, M. Lokajicek, V. P. Lombardo, J. D. Long, R. E. Long, L. Lopes, D. Lopez Mateos, B. Lopez Paredes, J. Lorenz, N. Lorenzo Martinez, M. Losada, P. Loscutoff, M. J. Losty, X. Lou, A. Lounis, J. Love, P. A. Love, A. J. Lowe, F. Lu, H. J. Lubatti, C. Luci, A. Lucotte, D. Ludwig, I. Ludwig, F. Luehring, W. Lukas, L. Luminari, E. Lund, J. Lundberg, O. Lundberg, B. Lund-Jensen, M. Lungwitz, D. Lynn, R. Lysak, E. Lytken, H. Ma, L. L. Ma, G. Maccarrone, A. Macchiolo, B. Maček, J. Machado Miguens, D. Macina, R. Mackeprang, R. Madar, R. J. Madaras, H. J. Maddocks, W. F. Mader, A. Madsen, M. Maeno, T. Maeno, L. Magnoni, E. Magradze, K. Mahboubi, J. Mahlstedt, S. Mahmoud, G. Mahout, C. Maiani, C. Maidantchik, A. Maio, S. Majewski, Y. Makida, N. Makovec, P. Mal, B. Malaescu, Pa. Malecki, V. P. Maleev, F. Malek, U. Mallik, D. Malon, C. Malone, S. Maltezos, V. M. Malyshev, S. Malyukov, J. Mamuzic, L. Mandelli, I. Mandić, R. Mandrysch, J. Maneira, A. Manfredini, L. Manhaes de Andrade Filho, J. A. Manjarres Ramos, A. Mann, P. M. Manning, A. Manousakis-Katsikakis, B. Mansoulie, R. Mantifel, L. Mapelli, L. March, J. F. Marchand, F. Marchese, G. Marchiori, M. Marcisovsky, C. P. Marino, C. N. Marques, F. Marroquim, Z. Marshall, L. F. Marti, S. Marti-Garcia, B. Martin, B. Martin, J. P. Martin, T. A. Martin, V. J. Martin, B. Martin dit Latour, H. Martinez, M. Martinez, S. Martin-Haugh, A. C. Martyniuk, M. Marx, F. Marzano, A. Marzin, L. Masetti, T. Mashimo, R. Mashinistov, J. Masik, A. L. Maslennikov, I. Massa, N. Massol, P. Mastrandrea, A. Mastroberardino, T. Masubuchi, H. Matsunaga, T. Matsushita, P. Mättig, S. Mättig, J. Mattmann, C. Mattravers, J. Maurer, S. J. Maxfield, D. A. Maximov, R. Mazini, L. Mazzaferro, M. Mazzanti, G. Mc Goldrick, S. P. Mc Kee, A. McCarn, R. L. McCarthy, T. G. McCarthy, N. A. McCubbin, K. W. McFarlane, J. A. Mcfayden, G. Mchedlidze, T. Mclaughlan, S. J. McMahon, R. A. McPherson, A. Meade, J. Mechnich, M. Mechtel, M. Medinnis, S. Meehan, R. Meera-Lebbai, S. Mehlhase, A. Mehta, K. Meier, C. Meineck, B. Meirose, C. Melachrinos, B. R. Mellado Garcia, F. Meloni, L. Mendoza Navas, A. Mengarelli, S. Menke, E. Meoni, K. M. Mercurio, S. Mergelmeyer, N. Meric, P. Mermod, L. Merola, C. Meroni, F. S. Merritt, H. Merritt, A. Messina, J. Metcalfe, A. S. Mete, C. Meyer, C. Meyer, J-P. Meyer, J. Meyer, J. Meyer, S. Michal, R. P. Middleton, S. Migas, L. Mijović, G. Mikenberg, M. Mikestikova, M. Mikuž, D. W. Miller, C. Mills, A. Milov, D. A. Milstead, D. Milstein, A. A. Minaenko, M. Miñano Moya, I. A. Minashvili, A. I. Mincer, B. Mindur, M. Mineev, Y. Ming, L. M. Mir, G. Mirabelli, T. Mitani, J. Mitrevski, V. A. Mitsou, S. Mitsui, P. S. Miyagawa, J. U. Mjörnmark, T. Moa, V. Moeller, S. Mohapatra, W. Mohr, S. Molander, R. Moles-Valls, A. Molfetas, K. Mönig, C. Monini, J. Monk, E. Monnier, J. Montejo Berlingen, F. Monticelli, S. Monzani, R. W. Moore, C. Mora Herrera, A. Moraes, N. Morange, J. Morel, D. Moreno, M. Moreno Llácer, P. Morettini, M. Morgenstern, M. Morii, S. Moritz, A. K. Morley, G. Mornacchi, J. D. Morris, L. Morvaj, H. G. Moser, M. Mosidze, J. Moss, R. Mount, E. Mountricha, S. V. Mouraviev, E. J. W. Moyse, R. D. Mudd, F. Mueller, J. Mueller, K. Mueller, T. Mueller, T. Mueller, D. Muenstermann, Y. Munwes, J. A. Murillo Quijada, W. J. Murray, I. Mussche, E. Musto, A. G. Myagkov, M. Myska, O. Nackenhorst, J. Nadal, K. Nagai, R. Nagai, Y. Nagai, K. Nagano, A. Nagarkar, Y. Nagasaka, M. Nagel, A. M. Nairz, Y. Nakahama, K. Nakamura, T. Nakamura, I. Nakano, H. Namasivayam, G. Nanava, A. Napier, R. Narayan, M. Nash, T. Nattermann, T. Naumann, G. Navarro, H. A. Neal, P. Yu. Nechaeva, T. J. Neep, A. Negri, G. Negri, M. Negrini, S. Nektarijevic, A. Nelson, T. K. Nelson, S. Nemecek, P. Nemethy, A. A. Nepomuceno, M. Nessi, M. S. Neubauer, M. Neumann, A. Neusiedl, R. M. Neves, P. Nevski, F. M. Newcomer, P. R. Newman, D. H. Nguyen, V. Nguyen Thi Hong, R. B. Nickerson, R. Nicolaidou, B. Nicquevert, J. Nielsen, N. Nikiforou, A. Nikiforov, V. Nikolaenko, I. Nikolic-Audit, K. Nikolics, K. Nikolopoulos, P. Nilsson, Y. Ninomiya, A. Nisati, R. Nisius, T. Nobe, L. Nodulman, M. Nomachi, I. Nomidis, S. Norberg, M. Nordberg, J. Novakova, M. Nozaki, L. Nozka, K. Ntekas, A.-E. Nuncio-Quiroz, G. Nunes Hanninger, T. Nunnemann, E. Nurse, B. J. O’Brien, F. O’Grady, D. C. O’Neil, V. O’Shea, L. B. Oakes, F. G. Oakham, H. Oberlack, J. Ocariz, A. Ochi, M. I. Ochoa, S. Oda, S. Odaka, H. Ogren, A. Oh, S. H. Oh, C. C. Ohm, T. Ohshima, W. Okamura, H. Okawa, Y. Okumura, T. Okuyama, A. Olariu, A. G. Olchevski, S. A. Olivares Pino, M. Oliveira, D. Oliveira Damazio, E. Oliver Garcia, D. Olivito, A. Olszewski, J. Olszowska, A. Onofre, P. U. E. Onyisi, C. J. Oram, M. J. Oreglia, Y. Oren, D. Orestano, N. Orlando, C. Oropeza Barrera, R. S. Orr, B. Osculati, R. Ospanov, G. Otero y Garzon, H. Otono, M. Ouchrif, E. A. Ouellette, F. Ould-Saada, A. Ouraou, K. P. Oussoren, Q. Ouyang, A. Ovcharova, M. Owen, S. Owen, V. E. Ozcan, N. Ozturk, K. Pachal, A. Pacheco Pages, C. Padilla Aranda, S. Pagan Griso, E. Paganis, C. Pahl, F. Paige, P. Pais, K. Pajchel, G. Palacino, S. Palestini, D. Pallin, A. Palma, J. D. Palmer, Y. B. Pan, E. Panagiotopoulou, J. G. Panduro Vazquez, P. Pani, N. Panikashvili, S. Panitkin, D. Pantea, Th. D. Papadopoulou, K. Papageorgiou, A. Paramonov, D. Paredes Hernandez, M. A. Parker, F. Parodi, J. A. Parsons, U. Parzefall, S. Pashapour, E. Pasqualucci, S. Passaggio, A. Passeri, F. Pastore, Fr. Pastore, G. Pásztor, S. Pataraia, N. D. Patel, J. R. Pater, S. Patricelli, T. Pauly, J. Pearce, M. Pedersen, S. Pedraza Lopez, R. Pedro, S. V. Peleganchuk, D. Pelikan, H. Peng, B. Penning, J. Penwell, D. V. Perepelitsa, T. Perez Cavalcanti, E. Perez Codina, M. T. Pérez García-Estañ, V. Perez Reale, L. Perini, H. Pernegger, R. Perrino, R. Peschke, V. D. Peshekhonov, K. Peters, R. F. Y. Peters, B. A. Petersen, J. Petersen, T. C. Petersen, E. Petit, A. Petridis, C. Petridou, E. Petrolo, F. Petrucci, M. Petteni, R. Pezoa, P. W. Phillips, G. Piacquadio, E. Pianori, A. Picazio, E. Piccaro, M. Piccinini, S. M. Piec, R. Piegaia, D. T. Pignotti, J. E. Pilcher, A. D. Pilkington, J. Pina, M. Pinamonti, A. Pinder, J. L. Pinfold, A. Pingel, B. Pinto, C. Pizio, M.-A. Pleier, V. Pleskot, E. Plotnikova, P. Plucinski, S. Poddar, F. Podlyski, R. Poettgen, L. Poggioli, D. Pohl, M. Pohl, G. Polesello, A. Policicchio, R. Polifka, A. Polini, C. S. Pollard, V. Polychronakos, D. Pomeroy, K. Pommès, L. Pontecorvo, B. G. Pope, G. A. Popeneciu, D. S. Popovic, A. Poppleton, X. Portell Bueso, G. E. Pospelov, S. Pospisil, K. Potamianos, I. N. Potrap, C. J. Potter, C. T. Potter, G. Poulard, J. Poveda, V. Pozdnyakov, R. Prabhu, P. Pralavorio, A. Pranko, S. Prasad, R. Pravahan, S. Prell, D. Price, J. Price, L. E. Price, D. Prieur, M. Primavera, M. Proissl, K. Prokofiev, F. Prokoshin, E. Protopapadaki, S. Protopopescu, J. Proudfoot, X. Prudent, M. Przybycien, H. Przysiezniak, S. Psoroulas, E. Ptacek, E. Pueschel, D. Puldon, M. Purohit, P. Puzo, Y. Pylypchenko, J. Qian, A. Quadt, D. R. Quarrie, W. B. Quayle, D. Quilty, V. Radeka, V. Radescu, S. K. Radhakrishnan, P. Radloff, F. Ragusa, G. Rahal, S. Rajagopalan, M. Rammensee, M. Rammes, A. S. Randle-Conde, C. Rangel-Smith, K. Rao, F. Rauscher, T. C. Rave, T. Ravenscroft, M. Raymond, A. L. Read, D. M. Rebuzzi, A. Redelbach, G. Redlinger, R. Reece, K. Reeves, A. Reinsch, H. Reisin, I. Reisinger, M. Relich, C. Rembser, Z. L. Ren, A. Renaud, M. Rescigno, S. Resconi, B. Resende, P. Reznicek, R. Rezvani, R. Richter, M. Ridel, P. Rieck, M. Rijssenbeek, A. Rimoldi, L. Rinaldi, E. Ritsch, I. Riu, G. Rivoltella, F. Rizatdinova, E. Rizvi, S. H. Robertson, A. Robichaud-Veronneau, D. Robinson, J. E. M. Robinson, A. Robson, J. G. Rocha de Lima, C. Roda, D. Roda Dos Santos, L. Rodrigues, S. Roe, O. Røhne, S. Rolli, A. Romaniouk, M. Romano, G. Romeo, E. Romero Adam, N. Rompotis, L. Roos, E. Ros, S. Rosati, K. Rosbach, A. Rose, M. Rose, P. L. Rosendahl, O. Rosenthal, V. Rossetti, E. Rossi, L. P. Rossi, R. Rosten, M. Rotaru, I. Roth, J. Rothberg, D. Rousseau, C. R. Royon, A. Rozanov, Y. Rozen, X. Ruan, F. Rubbo, I. Rubinskiy, V. I. Rud, C. Rudolph, M. S. Rudolph, F. Rühr, A. Ruiz-Martinez, L. Rumyantsev, Z. Rurikova, N. A. Rusakovich, A. Ruschke, J. P. Rutherfoord, N. Ruthmann, P. Ruzicka, Y. F. Ryabov, M. Rybar, G. Rybkin, N. C. Ryder, A. F. Saavedra, S. Sacerdoti, A. Saddique, I. Sadeh, H. F-W. Sadrozinski, R. Sadykov, F. Safai Tehrani, H. Sakamoto, Y. Sakurai, G. Salamanna, A. Salamon, M. Saleem, D. Salek, P. H. Sales De Bruin, D. Salihagic, A. Salnikov, J. Salt, B. M. Salvachua Ferrando, D. Salvatore, F. Salvatore, A. Salvucci, A. Salzburger, D. Sampsonidis, A. Sanchez, J. Sánchez, V. Sanchez Martinez, H. Sandaker, H. G. Sander, M. P. Sanders, M. Sandhoff, T. Sandoval, C. Sandoval, R. Sandstroem, D. P. C. Sankey, A. Sansoni, C. Santoni, R. Santonico, H. Santos, I. Santoyo Castillo, K. Sapp, A. Sapronov, J. G. Saraiva, E. Sarkisyan-Grinbaum, B. Sarrazin, G. Sartisohn, O. Sasaki, Y. Sasaki, N. Sasao, I. Satsounkevitch, G. Sauvage, E. Sauvan, J. B. Sauvan, P. Savard, V. Savinov, D. O. Savu, C. Sawyer, L. Sawyer, D. H. Saxon, J. Saxon, C. Sbarra, A. Sbrizzi, T. Scanlon, D. A. Scannicchio, M. Scarcella, J. Schaarschmidt, P. Schacht, D. Schaefer, A. Schaelicke, S. Schaepe, S. Schaetzel, U. Schäfer, A. C. Schaffer, D. Schaile, R. D. Schamberger, V. Scharf, V. A. Schegelsky, D. Scheirich, M. Schernau, M. I. Scherzer, C. Schiavi, J. Schieck, C. Schillo, M. Schioppa, S. Schlenker, E. Schmidt, K. Schmieden, C. Schmitt, C. Schmitt, S. Schmitt, B. Schneider, Y. J. Schnellbach, U. Schnoor, L. Schoeffel, A. Schoening, B. D. Schoenrock, A. L. S. Schorlemmer, M. Schott, D. Schouten, J. Schovancova, M. Schram, S. Schramm, M. Schreyer, C. Schroeder, N. Schroer, N. Schuh, M. J. Schultens, H.-C. Schultz-Coulon, H. Schulz, M. Schumacher, B. A. Schumm, Ph. Schune, A. Schwartzman, Ph. Schwegler, Ph. Schwemling, R. Schwienhorst, J. Schwindling, T. Schwindt, M. Schwoerer, F. G. Sciacca, E. Scifo, G. Sciolla, W. G. Scott, F. Scutti, J. Searcy, G. Sedov, E. Sedykh, S. C. Seidel, A. Seiden, F. Seifert, J. M. Seixas, G. Sekhniaidze, S. J. Sekula, K. E. Selbach, D. M. Seliverstov, G. Sellers, M. Seman, N. Semprini-Cesari, C. Serfon, L. Serin, L. Serkin, T. Serre, R. Seuster, H. Severini, F. Sforza, A. Sfyrla, E. Shabalina, M. Shamim, L. Y. Shan, J. T. Shank, Q. T. Shao, M. Shapiro, P. B. Shatalov, K. Shaw, P. Sherwood, S. Shimizu, M. Shimojima, T. Shin, M. Shiyakova, A. Shmeleva, M. J. Shochet, D. Short, S. Shrestha, E. Shulga, M. A. Shupe, S. Shushkevich, P. Sicho, D. Sidorov, A. Sidoti, F. Siegert, Dj. Sijacki, O. Silbert, J. Silva, Y. Silver, D. Silverstein, S. B. Silverstein, V. Simak, O. Simard, Lj. Simic, S. Simion, E. Simioni, B. Simmons, R. Simoniello, M. Simonyan, P. Sinervo, N. B. Sinev, V. Sipica, G. Siragusa, A. Sircar, A. N. Sisakyan, S. Yu. Sivoklokov, J. Sjölin, T. B. Sjursen, L. A. Skinnari, H. P. Skottowe, K. Yu. Skovpen, P. Skubic, M. Slater, T. Slavicek, K. Sliwa, V. Smakhtin, B. H. Smart, L. Smestad, S. Yu. Smirnov, Y. Smirnov, L. N. Smirnova, O. Smirnova, K. M. Smith, M. Smizanska, K. Smolek, A. A. Snesarev, G. Snidero, J. Snow, S. Snyder, R. Sobie, F. Socher, J. Sodomka, A. Soffer, D. A. Soh, C. A. Solans, M. Solar, J. Solc, E. Yu. Soldatov, U. Soldevila, E. Solfaroli Camillocci, A. A. Solodkov, O. V. Solovyanov, V. Solovyev, N. Soni, A. Sood, V. Sopko, B. Sopko, M. Sosebee, R. Soualah, P. Soueid, A. M. Soukharev, D. South, S. Spagnolo, F. Spanò, W. R. Spearman, R. Spighi, G. Spigo, M. Spousta, T. Spreitzer, B. Spurlock, R. D. St. Denis, J. Stahlman, R. Stamen, E. Stanecka, R. W. Stanek, C. Stanescu, M. Stanescu-Bellu, M. M. Stanitzki, S. Stapnes, E. A. Starchenko, J. Stark, P. Staroba, P. Starovoitov, R. Staszewski, P. Stavina, G. Steele, P. Steinbach, P. Steinberg, I. Stekl, B. Stelzer, H. J. Stelzer, O. Stelzer-Chilton, H. Stenzel, S. Stern, G. A. Stewart, J. A. Stillings, M. C. Stockton, M. Stoebe, K. Stoerig, G. Stoicea, S. Stonjek, A. R. Stradling, A. Straessner, J. Strandberg, S. Strandberg, A. Strandlie, E. Strauss, M. Strauss, P. Strizenec, R. Ströhmer, D. M. Strom, R. Stroynowski, S. A. Stucci, B. Stugu, I. Stumer, J. Stupak, P. Sturm, N. A. Styles, D. Su, J. Su, HS. Subramania, R. Subramaniam, A. Succurro, Y. Sugaya, C. Suhr, M. Suk, V. V. Sulin, S. Sultansoy, T. Sumida, X. Sun, J. E. Sundermann, K. Suruliz, G. Susinno, M. R. Sutton, Y. Suzuki, M. Svatos, S. Swedish, M. Swiatlowski, I. Sykora, T. Sykora, D. Ta, K. Tackmann, J. Taenzer, A. Taffard, R. Tafirout, N. Taiblum, Y. Takahashi, H. Takai, R. Takashima, H. Takeda, T. Takeshita, Y. Takubo, M. Talby, A. A. Talyshev, J. Y. C. Tam, M. C. Tamsett, K. G. Tan, J. Tanaka, R. Tanaka, S. Tanaka, S. Tanaka, A. J. Tanasijczuk, K. Tani, N. Tannoury, S. Tapprogge, S. Tarem, F. Tarrade, G. F. Tartarelli, P. Tas, M. Tasevsky, T. Tashiro, E. Tassi, A. Tavares Delgado, Y. Tayalati, C. Taylor, F. E. Taylor, G. N. Taylor, W. Taylor, F. A. Teischinger, M. Teixeira Dias Castanheira, P. Teixeira-Dias, K. K. Temming, H. Ten Kate, P. K. Teng, S. Terada, K. Terashi, J. Terron, S. Terzo, M. Testa, R. J. Teuscher, J. Therhaag, T. Theveneaux-Pelzer, S. Thoma, J. P. Thomas, E. N. Thompson, P. D. Thompson, P. D. Thompson, A. S. Thompson, L. A. Thomsen, E. Thomson, M. Thomson, W. M. Thong, R. P. Thun, F. Tian, M. J. Tibbetts, T. Tic, V. O. Tikhomirov, Yu. A. Tikhonov, S. Timoshenko, E. Tiouchichine, P. Tipton, S. Tisserant, T. Todorov, S. Todorova-Nova, B. Toggerson, J. Tojo, S. Tokár, K. Tokushuku, K. Tollefson, L. Tomlinson, M. Tomoto, L. Tompkins, K. Toms, N. D. Topilin, E. Torrence, H. Torres, E. Torró Pastor, J. Toth, F. Touchard, D. R. Tovey, H. L. Tran, T. Trefzger, L. Tremblet, A. Tricoli, I. M. Trigger, S. Trincaz-Duvoid, M. F. Tripiana, N. Triplett, W. Trischuk, B. Trocmé, C. Troncon, M. Trottier-McDonald, M. Trovatelli, P. True, M. Trzebinski, A. Trzupek, C. Tsarouchas, J. C-L. Tseng, P. V. Tsiareshka, D. Tsionou, G. Tsipolitis, N. Tsirintanis, S. Tsiskaridze, V. Tsiskaridze, E. G. Tskhadadze, I. I. Tsukerman, V. Tsulaia, J.-W. Tsung, S. Tsuno, D. Tsybychev, A. Tua, A. Tudorache, V. Tudorache, J. M. Tuggle, A. N. Tuna, S. A. Tupputi, S. Turchikhin, D. Turecek, I. Turk Cakir, R. Turra, P. M. Tuts, A. Tykhonov, M. Tylmad, M. Tyndel, K. Uchida, I. Ueda, R. Ueno, M. Ughetto, M. Ugland, M. Uhlenbrock, F. Ukegawa, G. Unal, A. Undrus, G. Unel, F. C. Ungaro, Y. Unno, D. Urbaniec, P. Urquijo, G. Usai, A. Usanova, L. Vacavant, V. Vacek, B. Vachon, N. Valencic, S. Valentinetti, A. Valero, L. Valery, S. Valkar, E. Valladolid Gallego, S. Vallecorsa, J. A. Valls Ferrer, R. Van Berg, P. C. Van Der Deijl, R. van der Geer, H. van der Graaf, R. Van Der Leeuw, D. van der Ster, N. van Eldik, P. van Gemmeren, J. Van Nieuwkoop, I. van Vulpen, M. C. van Woerden, M. Vanadia, W. Vandelli, A. Vaniachine, P. Vankov, F. Vannucci, G. Vardanyan, R. Vari, E. W. Varnes, T. Varol, D. Varouchas, A. Vartapetian, K. E. Varvell, V. I. Vassilakopoulos, F. Vazeille, T. Vazquez Schroeder, J. Veatch, F. Veloso, S. Veneziano, A. Ventura, D. Ventura, M. Venturi, N. Venturi, A. Venturini, V. Vercesi, M. Verducci, W. Verkerke, J. C. Vermeulen, A. Vest, M. C. Vetterli, O. Viazlo, I. Vichou, T. Vickey, O. E. Vickey Boeriu, G. H. A. Viehhauser, S. Viel, R. Vigne, M. Villa, M. Villaplana Perez, E. Vilucchi, M. G. Vincter, V. B. Vinogradov, J. Virzi, O. Vitells, M. Viti, I. Vivarelli, F. Vives Vaque, S. Vlachos, D. Vladoiu, M. Vlasak, A. Vogel, P. Vokac, G. Volpi, M. Volpi, G. Volpini, H. von der Schmitt, H. von Radziewski, E. von Toerne, V. Vorobel, M. Vos, R. Voss, J. H. Vossebeld, N. Vranjes, M. Vranjes Milosavljevic, V. Vrba, M. Vreeswijk, T. Vu Anh, R. Vuillermet, I. Vukotic, Z. Vykydal, W. Wagner, P. Wagner, S. Wahrmund, J. Wakabayashi, S. Walch, J. Walder, R. Walker, W. Walkowiak, R. Wall, P. Waller, B. Walsh, C. Wang, H. Wang, H. Wang, J. Wang, J. Wang, K. Wang, R. Wang, S. M. Wang, T. Wang, X. Wang, A. Warburton, C. P. Ward, D. R. Wardrope, M. Warsinsky, A. Washbrook, C. Wasicki, I. Watanabe, P. M. Watkins, A. T. Watson, I. J. Watson, M. F. Watson, G. Watts, S. Watts, A. T. Waugh, B. M. Waugh, S. Webb, M. S. Weber, S. W. Weber, J. S. Webster, A. R. Weidberg, P. Weigell, J. Weingarten, C. Weiser, H. Weits, P. S. Wells, T. Wenaus, D. Wendland, Z. Weng, T. Wengler, S. Wenig, N. Wermes, M. Werner, P. Werner, M. Wessels, J. Wetter, K. Whalen, A. White, M. J. White, R. White, S. White, D. Whiteson, D. Whittington, D. Wicke, F. J. Wickens, W. Wiedenmann, M. Wielers, P. Wienemann, C. Wiglesworth, L. A. M. Wiik-Fuchs, P. A. Wijeratne, A. Wildauer, M. A. Wildt, I. Wilhelm, H. G. Wilkens, J. Z. Will, H. H. Williams, S. Williams, W. Willis, S. Willocq, J. A. Wilson, A. Wilson, I. Wingerter-Seez, S. Winkelmann, F. Winklmeier, M. Wittgen, T. Wittig, J. Wittkowski, S. J. Wollstadt, M. W. Wolter, H. Wolters, W. C. Wong, B. K. Wosiek, J. Wotschack, M. J. Woudstra, K. W. Wozniak, K. Wraight, M. Wright, S. L. Wu, X. Wu, Y. Wu, E. Wulf, T. R. Wyatt, B. M. Wynne, S. Xella, M. Xiao, C. Xu, D. Xu, L. Xu, B. Yabsley, S. Yacoob, M. Yamada, H. Yamaguchi, Y. Yamaguchi, A. Yamamoto, K. Yamamoto, S. Yamamoto, T. Yamamura, T. Yamanaka, K. Yamauchi, Y. Yamazaki, Z. Yan, H. Yang, H. Yang, U. K. Yang, Y. Yang, S. Yanush, L. Yao, Y. Yasu, E. Yatsenko, K. H. Yau Wong, J. Ye, S. Ye, A. L. Yen, E. Yildirim, M. Yilmaz, R. Yoosoofmiya, K. Yorita, R. Yoshida, K. Yoshihara, C. Young, C. J. S. Young, S. Youssef, D. R. Yu, J. Yu, J. Yu, L. Yuan, A. Yurkewicz, B. Zabinski, R. Zaidan, A. M. Zaitsev, A. Zaman, S. Zambito, L. Zanello, D. Zanzi, A. Zaytsev, C. Zeitnitz, M. Zeman, A. Zemla, K. Zengel, O. Zenin, T. Ženiš, D. Zerwas, G. Zevi della Porta, D. Zhang, H. Zhang, J. Zhang, L. Zhang, X. Zhang, Z. Zhang, Z. Zhao, A. Zhemchugov, J. Zhong, B. Zhou, L. Zhou, N. Zhou, C. G. Zhu, H. Zhu, J. Zhu, Y. Zhu, X. Zhuang, A. Zibell, D. Zieminska, N. I. Zimine, C. Zimmermann, R. Zimmermann, S. Zimmermann, S. Zimmermann, Z. Zinonos, M. Ziolkowski, R. Zitoun, G. Zobernig, A. Zoccoli, M. zur Nedden, G. Zurzolo, V. Zutshi, L. Zwalinski

**Affiliations:** 1 School of Chemistry and Physics, University of Adelaide, Adelaide, Australia; 2 Physics Department, SUNY Albany, Albany, NY USA; 3 Department of Physics, University of Alberta, Edmonton, AB Canada; 4 Department of Physics, Ankara University, Ankara, Turkey; Department of Physics, Gazi University, Ankara, Turkey; Division of Physics, TOBB University of Economics and Technology, Ankara, Turkey; Turkish Atomic Energy Authority, Ankara, Turkey; 5 LAPP, CNRS/IN2P3 and Université de Savoie, Annecy-le-Vieux, France; 6 High Energy Physics Division, Argonne National Laboratory, Argonne, IL USA; 7 Department of Physics, University of Arizona, Tucson, AZ USA; 8 Department of Physics, The University of Texas at Arlington, Arlington, TX USA; 9 Physics Department, University of Athens, Athens, Greece; 10 Physics Department, National Technical University of Athens, Zografou, Greece; 11 Institute of Physics, Azerbaijan Academy of Sciences, Baku, Azerbaijan; 12 Institut de Física d’Altes Energies and Departament de Física de la Universitat Autònoma de Barcelona, Barcelona, Spain; 13 Institute of Physics, University of Belgrade, Belgrade, Serbia; Vinca Institute of Nuclear Sciences, University of Belgrade, Belgrade, Serbia; 14 Department for Physics and Technology, University of Bergen, Bergen, Norway; 15 Physics Division, Lawrence Berkeley National Laboratory and University of California, Berkeley, CA USA; 16 Department of Physics, Humboldt University, Berlin, Germany; 17 Albert Einstein Center for Fundamental Physics and Laboratory for High Energy Physics, University of Bern, Bern, Switzerland; 18 School of Physics and Astronomy, University of Birmingham, Birmingham, UK; 19 Department of Physics, Bogazici University, Istanbul, Turkey; Department of Physics, Dogus University, Istanbul, Turkey; Department of Physics Engineering, Gaziantep University, Gaziantep, Turkey; 20 INFN Sezione di Bologna, Bologna, Italy; Dipartimento di Fisica e Astronomia, Università di Bologna, Bologna, Italy; 21 Physikalisches Institut, University of Bonn, Bonn, Germany; 22 Department of Physics, Boston University, Boston, MA USA; 23 Department of Physics, Brandeis University, Waltham, MA USA; 24 Universidade Federal do Rio De Janeiro COPPE/EE/IF, Rio de Janeiro, Brazil; Federal University of Juiz de Fora (UFJF), Juiz de Fora, Brazil; Federal University of Sao Joao del Rei (UFSJ), Sao Joao del Rei, Brazil; Instituto de Fisica, Universidade de Sao Paulo, São Paulo, Brazil; 25 Physics Department, Brookhaven National Laboratory, Upton, NY USA; 26 National Institute of Physics and Nuclear Engineering, Bucharest, Romania; Physics Department, National Institute for Research and Development of Isotopic and Molecular Technologies, Cluj-Napoca, Romania; University Politehnica Bucharest, Bucharest, Romania; West University in Timisoara, Timisoara, Romania; 27 Departamento de Física, Universidad de Buenos Aires, Buenos Aires, Argentina; 28 Cavendish Laboratory, University of Cambridge, Cambridge, UK; 29 Department of Physics, Carleton University, Ottawa, ON Canada; 30 CERN, Geneva, Switzerland; 31 Enrico Fermi Institute, University of Chicago, Chicago, IL USA; 32 Departamento de Física, Pontificia Universidad Católica de Chile, Santiago, Chile; Departamento de Física, Universidad Técnica Federico Santa María, Valparaiso, Chile; 33 Institute of High Energy Physics, Chinese Academy of Sciences, Beijing, China; Department of Modern Physics, University of Science and Technology of China, Hefei, Anhui, China; Department of Physics, Nanjing University, Nanjing, Jiangsu, China; School of Physics, Shandong University, Shandong, China; Physics Department, Shanghai Jiao Tong University, Shanghai, China; 34 Laboratoire de Physique Corpusculaire, Clermont Université and Université Blaise Pascal and CNRS/IN2P3, Clermont-Ferrand, France; 35 Nevis Laboratory, Columbia University, Irvington, NY USA; 36 Niels Bohr Institute, University of Copenhagen, Copenhagen, Denmark; 37 INFN Gruppo Collegato di Cosenza, Laboratori Nazionali di Frascati, Frascati, Italy; Dipartimento di Fisica, Università della Calabria, Rende, Italy; 38 Faculty of Physics and Applied Computer Science, AGH University of Science and Technology, Kraków, Poland; Marian Smoluchowski Institute of Physics, Jagiellonian University, Kraków, Poland; 39 The Henryk Niewodniczanski Institute of Nuclear Physics, Polish Academy of Sciences, Kraków, Poland; 40 Physics Department, Southern Methodist University, Dallas, TX USA; 41 Physics Department, University of Texas at Dallas, Richardson, TX USA; 42 DESY, Hamburg and Zeuthen, Germany; 43 Institut für Experimentelle Physik IV, Technische Universität Dortmund, Dortmund, Germany; 44 Institut für Kern- und Teilchenphysik, Technische Universität Dresden, Dresden, Germany; 45 Department of Physics, Duke University, Durham, NC USA; 46 SUPA-School of Physics and Astronomy, University of Edinburgh, Edinburgh, UK; 47 INFN Laboratori Nazionali di Frascati, Frascati, Italy; 48 Fakultät für Mathematik und Physik, Albert-Ludwigs-Universität, Freiburg, Germany; 49 Section de Physique, Université de Genève, Geneva, Switzerland; 50 INFN Sezione di Genova, Genoa, Italy; Dipartimento di Fisica, Università di Genova, Genoa, Italy; 51 E. Andronikashvili Institute of Physics, Iv. Javakhishvili Tbilisi State University, Tbilisi, Georgia; High Energy Physics Institute, Tbilisi State University, Tbilisi, Georgia; 52 II Physikalisches Institut, Justus-Liebig-Universität Giessen, Giessen, Germany; 53 SUPA-School of Physics and Astronomy, University of Glasgow, Glasgow, UK; 54 II Physikalisches Institut, Georg-August-Universität, Göttingen, Germany; 55 Laboratoire de Physique Subatomique et de Cosmologie, Université Joseph Fourier and CNRS/IN2P3 and Institut National Polytechnique de Grenoble, Grenoble, France; 56 Department of Physics, Hampton University, Hampton, VA USA; 57 Laboratory for Particle Physics and Cosmology, Harvard University, Cambridge, MA USA; 58 Kirchhoff-Institut für Physik, Ruprecht-Karls-Universität Heidelberg, Heidelberg, Germany; Physikalisches Institut, Ruprecht-Karls-Universität Heidelberg, Heidelberg, Germany; ZITI Institut für technische Informatik, Ruprecht-Karls-Universität Heidelberg, Mannheim, Germany; 59 Faculty of Applied Information Science, Hiroshima Institute of Technology, Hiroshima, Japan; 60 Department of Physics, Indiana University, Bloomington, IN USA; 61 Institut für Astro- und Teilchenphysik, Leopold-Franzens-Universität, Innsbruck, Austria; 62 University of Iowa, Iowa City, IA USA; 63 Department of Physics and Astronomy, Iowa State University, Ames, IA USA; 64 Joint Institute for Nuclear Research, JINR Dubna, Dubna, Russia; 65 KEK, High Energy Accelerator Research Organization, Tsukuba, Japan; 66 Graduate School of Science, Kobe University, Kobe, Japan; 67 Faculty of Science, Kyoto University, Kyoto, Japan; 68 Kyoto University of Education, Kyoto, Japan; 69 Department of Physics, Kyushu University, Fukuoka, Japan; 70 Instituto de Física La Plata, Universidad Nacional de La Plata and CONICET, La Plata, Argentina; 71 Physics Department, Lancaster University, Lancaster, UK; 72 INFN Sezione di Lecce, Lecce, Italy; Dipartimento di Matematica e Fisica, Università del Salento, Lecce, Italy; 73 Oliver Lodge Laboratory, University of Liverpool, Liverpool, UK; 74 Department of Physics, Jožef Stefan Institute and University of Ljubljana, Ljubljana, Slovenia; 75 School of Physics and Astronomy, Queen Mary University of London, London, UK; 76 Department of Physics, Royal Holloway University of London, Surrey, UK; 77 Department of Physics and Astronomy, University College London, London, UK; 78 Louisiana Tech University, Ruston, LA USA; 79 Laboratoire de Physique Nucléaire et de Hautes Energies, UPMC and Université Paris-Diderot and CNRS/IN2P3, Paris, France; 80 Fysiska institutionen, Lunds universitet, Lund, Sweden; 81 Departamento de Fisica Teorica C-15, Universidad Autonoma de Madrid, Madrid, Spain; 82 Institut für Physik, Universität Mainz, Mainz, Germany; 83 School of Physics and Astronomy, University of Manchester, Manchester, UK; 84 CPPM, Aix-Marseille Université and CNRS/IN2P3, Marseille, France; 85 Department of Physics, University of Massachusetts, Amherst, MA USA; 86 Department of Physics, McGill University, Montreal, QC Canada; 87 School of Physics, University of Melbourne, Parkville, VIC Australia; 88 Department of Physics, The University of Michigan, Ann Arbor, MI USA; 89 Department of Physics and Astronomy, Michigan State University, East Lansing, MI USA; 90 INFN Sezione di Milano, Milan, Italy; Dipartimento di Scienze Fisiche, Università di Milano, Milan, Italy; 91 B.I. Stepanov Institute of Physics, National Academy of Sciences of Belarus, Minsk, Republic of Belarus; 92 National Scientific and Educational Centre for Particle and High Energy Physics, Minsk, Republic of Belarus; 93 Department of Physics, Massachusetts Institute of Technology, Cambridge, MA USA; 94 Group of Particle Physics, University of Montreal, Montreal, QC Canada; 95 P.N. Lebedev Institute of Physics, Academy of Sciences, Moscow, Russia; 96 Institute for Theoretical and Experimental Physics (ITEP), Moscow, Russia; 97 Moscow Engineering and Physics Institute (MEPhI), Moscow, Russia; 98 D.V. Skobeltsyn Institute of Nuclear Physics, M.V. Lomonosov Moscow State University, Moscow, Russia; 99 Fakultät für Physik, Ludwig-Maximilians-Universität München, Munich, Germany; 100 Max-Planck-Institut für Physik (Werner-Heisenberg-Institut), Munich, Germany; 101 Nagasaki Institute of Applied Science, Nagasaki, Japan; 102 Graduate School of Science and Kobayashi-Maskawa Institute, Nagoya University, Nagoya, Japan; 103 INFN Sezione di Napoli, Naples, Italy; Dipartimento di Fisica, Università di Napoli, Naples, Italy; 104 Department of Physics and Astronomy, University of New Mexico, Albuquerque, NM USA; 105 Institute for Mathematics, Astrophysics and Particle Physics, Radboud University Nijmegen/Nikhef, Nijmegen, The Netherlands; 106 Nikhef National Institute for Subatomic Physics and University of Amsterdam, Amsterdam, The Netherlands; 107 Department of Physics, Northern Illinois University, DeKalb, IL USA; 108 Budker Institute of Nuclear Physics, SB RAS, Novosibirsk, Russia; 109 Department of Physics, New York University, New York, NY USA; 110 Ohio State University, Columbus, OH USA; 111 Faculty of Science, Okayama University, Okayama, Japan; 112 Homer L. Dodge Department of Physics and Astronomy, University of Oklahoma, Norman, OK USA; 113 Department of Physics, Oklahoma State University, Stillwater, OK USA; 114 Palacký University, RCPTM, Olomouc, Czech Republic; 115 Center for High Energy Physics, University of Oregon, Eugene, OR USA; 116 LAL, Université Paris-Sud and CNRS/IN2P3, Orsay, France; 117 Graduate School of Science, Osaka University, Osaka, Japan; 118 Department of Physics, University of Oslo, Oslo, Norway; 119 Department of Physics, Oxford University, Oxford, UK; 120 INFN Sezione di Pavia, Pavia, Italy; Dipartimento di Fisica, Università di Pavia, Pavia, Italy; 121 Department of Physics, University of Pennsylvania, Philadelphia, PA USA; 122 Petersburg Nuclear Physics Institute, Gatchina, Russia; 123 INFN Sezione di Pisa, Pisa, Italy; Dipartimento di Fisica E. Fermi, Università di Pisa, Pisa, Italy; 124 Department of Physics and Astronomy, University of Pittsburgh, Pittsburgh, PA USA; 125 Laboratorio de Instrumentacao e Fisica Experimental de Particulas-LIP, Lisbon, Portugal; Faculdade de Ciências, Universidade de Lisboa, Lisbon, Portugal; Department of Physics, University of Coimbra, Coimbra, Portugal; Centro de Física Nuclear da Universidade de Lisboa, Lisbon, Portugal; Departamento de Fisica, Universidade do Minho, Braga, Portugal; Departamento de Fisica Teorica y del Cosmos and CAFPE, Universidad de Granada, Granada, Spain; Dep Fisica and CEFITEC of Faculdade de Ciencias e Tecnologia, Universidade Nova de Lisboa, Caparica, Portugal; 126 Institute of Physics, Academy of Sciences of the Czech Republic, Prague, Czech Republic; 127 Czech Technical University in Prague, Prague, Czech Republic; 128 Faculty of Mathematics and Physics, Charles University in Prague, Prague, Czech Republic; 129 State Research Center Institute for High Energy Physics, Protvino, Russia; 130 Particle Physics Department, Rutherford Appleton Laboratory, Didcot, UK; 131 Physics Department, University of Regina, Regina, SK Canada; 132 Ritsumeikan University, Kusatsu, Shiga Japan; 133 INFN Sezione di Roma, Rome, Italy; Dipartimento di Fisica, Sapienza Università di Roma, Rome, Italy; 134 INFN Sezione di Roma Tor Vergata, Rome, Italy; Dipartimento di Fisica, Università di Roma Tor Vergata, Rome, Italy; 135 INFN Sezione di Roma Tre, Rome, Italy; Dipartimento di Matematica e Fisica, Università Roma Tre, Rome, Italy; 136 Faculté des Sciences Ain Chock, Réseau Universitaire de Physique des Hautes Energies-Université Hassan II, Casablanca, Morocco; Centre National de l’Energie des Sciences Techniques Nucleaires, Rabat, Morocco; Faculté des Sciences Semlalia, Université Cadi Ayyad, LPHEA-Marrakech, Marrakech, Morocco; Faculté des Sciences, Université Mohamed Premier and LPTPM, Oujda, Morocco; Faculté des Sciences, Université Mohammed V-Agdal, Rabat, Morocco; 137 DSM/IRFU (Institut de Recherches sur les Lois Fondamentales de l’Univers), CEA Saclay (Commissariat à l’Energie Atomique et aux Energies Alternatives), Gif-sur-Yvette, France; 138 Santa Cruz Institute for Particle Physics, University of California Santa Cruz, Santa Cruz, CA USA; 139 Department of Physics, University of Washington, Seattle, WA USA; 140 Department of Physics and Astronomy, University of Sheffield, Sheffield, UK; 141 Department of Physics, Shinshu University, Nagano, Japan; 142 Fachbereich Physik, Universität Siegen, Siegen, Germany; 143 Department of Physics, Simon Fraser University, Burnaby, BC Canada; 144 SLAC National Accelerator Laboratory, Stanford, CA USA; 145 Faculty of Mathematics, Physics and Informatics, Comenius University, Bratislava, Slovak Republic; Department of Subnuclear Physics, Institute of Experimental Physics of the Slovak Academy of Sciences, Kosice, Slovak Republic; 146 Department of Physics, University of Cape Town, Cape Town, South Africa; Department of Physics, University of Johannesburg, Johannesburg, South Africa; School of Physics, University of the Witwatersrand, Johannesburg, South Africa; 147 Department of Physics, Stockholm University, Stockholm, Sweden; The Oskar Klein Centre, Stockholm, Sweden; 148 Physics Department, Royal Institute of Technology, Stockholm, Sweden; 149 Departments of Physics and Astronomy and Chemistry, Stony Brook University, Stony Brook, NY USA; 150 Department of Physics and Astronomy, University of Sussex, Brighton, UK; 151 School of Physics, University of Sydney, Sydney, Australia; 152 Institute of Physics, Academia Sinica, Taipei, Taiwan; 153 Department of Physics, Technion: Israel Institute of Technology, Haifa, Israel; 154 Raymond and Beverly Sackler School of Physics and Astronomy, Tel Aviv University, Tel Aviv, Israel; 155 Department of Physics, Aristotle University of Thessaloniki, Thessaloniki, Greece; 156 International Center for Elementary Particle Physics and Department of Physics, The University of Tokyo, Tokyo, Japan; 157 Graduate School of Science and Technology, Tokyo Metropolitan University, Tokyo, Japan; 158 Department of Physics, Tokyo Institute of Technology, Tokyo, Japan; 159 Department of Physics, University of Toronto, Toronto, ON Canada; 160 TRIUMF, Vancouver, BC, Canada; Department of Physics and Astronomy, York University, Toronto, ON Canada; 161 Faculty of Pure and Applied Sciences, University of Tsukuba, Tsukuba, Japan; 162 Department of Physics and Astronomy, Tufts University, Medford, MA USA; 163 Centro de Investigaciones, Universidad Antonio Narino, Bogotá, Colombia; 164 Department of Physics and Astronomy, University of California Irvine, Irvine, CA USA; 165 INFN Gruppo Collegato di Udine, Sezione di Trieste, Italy; ICTP, Trieste, Italy; Dipartimento di Chimica, Fisica e Ambiente, Università di Udine, Udine, Italy; 166 Department of Physics, University of Illinois, Urbana, IL USA; 167 Department of Physics and Astronomy, University of Uppsala, Uppsala, Sweden; 168 Instituto de Física Corpuscular (IFIC) and Departamento de Física Atómica, Molecular y Nuclear and Departamento de Ingeniería Electrónica and Instituto de Microelectrónica de Barcelona (IMB-CNM), University of Valencia and CSIC, Valencia, Spain; 169 Department of Physics, University of British Columbia, Vancouver, BC Canada; 170 Department of Physics and Astronomy, University of Victoria, Victoria, BC Canada; 171 Department of Physics, University of Warwick, Coventry, UK; 172 Waseda University, Tokyo, Japan; 173 Department of Particle Physics, The Weizmann Institute of Science, Rehovot, Israel; 174 Department of Physics, University of Wisconsin, Madison, WI USA; 175 Fakultät für Physik und Astronomie, Julius-Maximilians-Universität, Würzburg, Germany; 176 Fachbereich C Physik, Bergische Universität Wuppertal, Wuppertal, Germany; 177 Department of Physics, Yale University, New Haven, CT USA; 178 Yerevan Physics Institute, Yerevan, Armenia; 179 Centre de Calcul de l’Institut National de Physique Nucléaire et de Physique des Particules (IN2P3), Villeurbanne, France; 180 CERN, 1211 Geneva 23, Switzerland

## Abstract

The jet energy scale (JES) and its systematic uncertainty are determined for jets measured with the ATLAS detector using proton–proton collision data with a centre-of-mass energy of $\sqrt{s}=7$ TeV corresponding to an integrated luminosity of $4.7$ $\,\,\text{ fb }^{-1}$. Jets are reconstructed from energy deposits forming topological clusters of calorimeter cells using the anti-$k_{t}$ algorithm with distance parameters $R=0.4$ or $R=0.6$, and are calibrated using MC simulations. A residual JES correction is applied to account for differences between data and MC simulations. This correction and its systematic uncertainty are estimated using a combination of in situ techniques exploiting the transverse momentum balance between a jet and a reference object such as a photon or a $Z$ boson, for ${20} \le p_{\mathrm {T}}^\mathrm {jet}<{1000}\, ~\mathrm{GeV }$ and pseudorapidities $|\eta |<{4.5}$. The effect of multiple proton–proton interactions is corrected for, and an uncertainty is evaluated using in situ techniques. The smallest JES uncertainty of less than 1 % is found in the central calorimeter region ($|\eta |<{1.2}$) for jets with ${55} \le p_{\mathrm {T}}^\mathrm {jet}<{500}\, ~\mathrm{GeV }$. For central jets at lower $p_{\mathrm {T}}$, the uncertainty is about 3 %. A consistent JES estimate is found using measurements of the calorimeter response of single hadrons in proton–proton collisions and test-beam data, which also provide the estimate for $p_{\mathrm {T}}^\mathrm {jet}> 1$ TeV. The calibration of forward jets is derived from dijet $p_{\mathrm {T}}$ balance measurements. The resulting uncertainty reaches its largest value of 6 % for low-$p_{\mathrm {T}}$ jets at $|\eta |=4.5$. Additional JES uncertainties due to specific event topologies, such as close-by jets or selections of event samples with an enhanced content of jets originating from light quarks or gluons, are also discussed. The magnitude of these uncertainties depends on the event sample used in a given physics analysis, but typically amounts to 0.5–3 %.

## Introduction

Jets are the dominant feature of high-energy, hard proton–proton interactions at the Large Hadron Collider (LHC) at CERN. They are key ingredients of many physics measurements and for searches for new phenomena. In this paper, jets are observed as groups of topologically related energy deposits in the ATLAS calorimeters, associated with tracks of charged particles as measured in the inner tracking detector. They are reconstructed with the anti-$k_{t}$ jet algorithm [1] and are calibrated using Monte Carlo (MC) simulation.

A first estimate of the jet energy scale (JES) uncertainty of about 5–9 % depending on the jet transverse momentum ($p_{\mathrm {T}}$), described in Ref. [2], is based on information available before the first proton–proton collisions at the LHC, and initial proton–proton collision data taken in 2010. A reduced uncertainty of about 2.5 % in the central calorimeter region over a wide $p_{\mathrm {T}}$ range of $60 \lesssim p_{\mathrm {T}}< 800$ GeV was achieved after applying the increased knowledge of the detector performance obtained during the analysis of this first year of ATLAS data taking [3]. This estimation used single-hadron calorimeter response measurements, systematic variations of MC simulation configurations, and in situ techniques, where the jet transverse momentum is compared to the $p_{\mathrm {T}}$ of a reference object. These measurements were performed using the 2010 dataset, corresponding to an integrated luminosity of 38 pb$^{-1}$ [4].

During the year 2011 the ATLAS detector [5] collected proton–proton collision data at a centre-of-mass energy of $\sqrt{s}=7$ TeV, corresponding to an integrated luminosity of about $4.7$ $\,\,\text{ fb }^{-1}$. The larger dataset makes it possible to further improve the precision of the jet energy measurement, and also to apply a correction derived from detailed comparisons of data and MC simulation using in situ techniques. This document presents the results of such an improved calibration of the jet energy measurement and the determination of the uncertainties using the 2011 dataset.

The energy measurement of jets produced in proton-proton and electron-proton collisions is also discussed by other experiments [6–17].

The outline of the paper is as follows. Section 2 describes the ATLAS detector. The Monte Carlo simulation framework is presented in Sect. 3, and the used dataset is described in Sect. 4. Section 5 summarises the jet reconstruction and calibration strategy. The correction method for the effect of additional proton–proton interactions is discussed in Sect. 6. Section 7 provides an overview of the techniques based on $p_{\mathrm {T}}$ balance that are described in detail in Sects. 8 to 11. First the intercalibration between the central and the forward detector using events with two high-$p_{\mathrm {T}}$ jets is presented in Sect. 8. Then, in situ techniques to assess differences of the jet energy measurement between data and Monte Carlo simulation exploiting the $p_{\mathrm {T}}$ balance between a jet and a well-measured reference object are detailed. The reference objects are $Z$ bosons in Sect. 9, photons in Sect. 10, and a system of low-$p_{\mathrm {T}}$ jets in Sect. 11. The validation of the forward-jet energy measurements with $p_{\mathrm {T}}$ balance methods using $Z$-jet and $\gamma \text {-jet}$ events follows in Sect. 12. The strategy on how to extract a final jet calibration out of the combination of in situ techniques, and the evaluation strategies for determining the corresponding systematic uncertainties, are discussed in Sect. 13. The same section also shows the final result of the jet calibration, including its systematic uncertainty, from the combination of the in situ techniques.

Section 14 compares the JES uncertainty as derived from the single-hadron calorimeter response measurements to that obtained from the in situ method based on $p_{\mathrm {T}}$ balance discussed in the preceding sections. Comparisons to JES uncertainties using the $W$ boson mass constraint in final states with hadronically decaying $W$ bosons are presented in Sect. 15.

Additional contributions to the systematic uncertainties of the jet measurement in ATLAS are presented in Sects. 16–18, where the correction for the effect of additional proton–proton interactions in the event, the presence of other close-by jets, and the response dependence on the jet fragmentation (jet flavour) are discussed. The uncertainties for explicitly tagged jets with heavy-flavour content are outlined in Sect. 19. A brief discussion of the correction of the calorimeter energy in regions with hardware failures and the associated uncertainty on the jet energy measurement is presented in Sect. 20.

A summary of the total jet energy scale uncertainty is given in Sect. 21. Conclusions follow in Sect. 22. A comparison of the systematic uncertainties of the JES in ATLAS with previous calibrations is presented in Appendix A.

## The ATLAS detector

### Detector description

The ATLAS detector consists of a tracking system (Inner Detector, or ID in the following), sampling electromagnetic and hadronic calorimeters and muon chambers. A detailed description of the ATLAS experiment can be found in Ref. [5].

The Inner Detector has complete azimuthal coverage and spans the pseudorapidity^1^ region $|\eta |<2.5$. It consists of layers of silicon pixel detectors, silicon microstrip detectors and transition radiation tracking detectors, all of which are immersed in a solenoid magnet that provides a uniform magnetic field of 2 T.

Jets are reconstructed using the ATLAS calorimeters, whose granularity and material varies as a function of $\eta$. The electromagnetic calorimetry (EM) is provided by high-granularity liquid-argon sampling calorimeters (LAr), using lead as an absorber. It is divided into one barrel ($|\eta |<1.475$) and two end-cap ($1.375<|\eta |<3.2$) regions. The hadronic calorimetry is divided into three distinct sections. The most central contains the central barrel region ($|\eta |<0.8$) and two extended barrel regions ($0.8<|\eta |<1.7$). These regions are instrumented with scintillator-tile/steel hadronic calorimeters (Tile). Each barrel region consists of 64 modules with individual $\phi$ coverages of $\sim0.1$ rad. The two hadronic end-cap calorimeters (HEC; $1.5<|\eta |<3.2$) feature liquid-argon/copper calorimeter modules. The two forward calorimeters (FCal; $3.1<|\eta |<4.9$) are instrumented with liquid-argon/copper and liquid-argon/tungsten modules to provide electromagnetic and hadronic energy measurements, respectively.

The muon spectrometer surrounds the ATLAS calorimeter. A system of three large air-core toroids, a barrel and two endcaps, generates a magnetic field in the pseudorapidity range of $|\eta | < 2.7$. The muon spectrometer measures muon tracks with three layers of precision tracking chambers and is instrumented with separate trigger chambers.

The trigger system for the ATLAS detector consists of a hardware-based Level 1 (L1) and a software-based High Level Trigger (HLT) [18]. At L1, jets are first built from coarse-granularity calorimeter towers using a sliding window algorithm, and then subjected to early trigger decisions. This is refined using jets reconstructed from calorimeter cells in the HLT, with algorithms similar to the ones applied offline.

### Calorimeter pile-up sensitivity

One important feature for the understanding of the contribution from additional proton–proton interactions (pile-up) to the signal in the 2011 dataset is the sensitivity of the ATLAS liquid argon calorimeters to the bunch crossing history. In any LAr calorimeter cell, the reconstructed energy is sensitive to the proton–proton interactions occurring in approximately 12 (2011 data, 24 at LHC design conditions) preceding and one immediately following bunch crossings (*out-of-time pile-up*), in addition to pile-up interactions in the current bunch crossing (*in-time pile-up*). This is due to the relatively long charge collection time in these calorimeters (typically 400–600 ns), as compared to the bunch crossing intervals at the LHC (design 25 ns and actually 50 ns in 2011 data). To reduce this sensitivity, a fast, bipolar shaped signal^2^ is used with net zero integral over time.

The signal shapes in the liquid argon calorimeters are optimised for this purpose, leading to cancellation on average of in-time and out-of-time pile-up in any given calorimeter cell. By design of the shaping amplifier, the most efficient suppression is achieved for 25 ns bunch spacing in the LHC beams. It is fully effective in the limit where, for each bunch crossing, about the same amount of energy is deposited in each calorimeter cell.

The 2011 beam conditions, with 50 ns bunch spacing and a relatively low cell occupancy from the achieved instantaneous luminosities, do not allow for full pile-up suppression by signal shaping, in particular in the central calorimeter region. Pile-up suppression is further limited by large fluctuations in the number of additional interactions from bunch crossing to bunch crossing, and in the energy flow patterns of the individual collisions in the time window of sensitivity of approximately 600 ns. Consequently, the shaped signal extracted by digital filtering shows a principal sensitivity to in-time and out-of-time pile-up, in particular in terms of a residual non-zero cell-signal baseline. This baseline can lead to relevant signal offsets once the noise suppression, an important part of the calorimeter signal extraction strategy presented in Sect. 5, is applied.

Corrections mitigating the effect of these signal offsets on the reconstructed jet energy are discussed in the context of the pile-up suppression strategy in Sect. 6.1. All details of the ATLAS liquid argon calorimeter readout and signal processing can be found in Ref. [19].

The Tile calorimeter shows very little sensitivity to pile-up since most of the associated (soft particle) energy flow is absorbed in the LAr calorimeters in front of it. Moreover, out-of-time pile-up is suppressed by a short shaping time with sensitivity to only about 3 bunch crossings [20].

## Monte Carlo simulation of jets in the ATLAS detector

The energy and direction of particles produced in proton–proton collisions are simulated using various MC event generators. An overview of these generators for LHC physics can be found in Ref. [21]. The samples using different event generators and theoretical models are described below. All samples are produced at $\sqrt{s}= 7$ TeV.

### Inclusive jet Monte Carlo simulation samples

1. Pythia (version 6.425) [22] is used for the generation of the baseline simulation event samples. It models the hard sub-process in the final states of the generated proton–proton collisions using a $2 \rightarrow 2$ matrix element at leading order in the strong coupling $\alpha _{\mathrm {S}}$. Additional radiation is modelled in the leading logarithmic (LL) approximation by $p_{\mathrm {T}}$-ordered parton showers [23]. Multiple parton interactions (MPI) [24], as well as fragmentation and hadronisation based on the Lund string model [25], are also generated. Relevant parameters for the modelling of the parton shower and multiple parton interactions in the underlying event (UE) are tuned to LHC data (ATLAS Pythia tune AUET2B [26] with the MRST LO** parton density function (PDF) [27]). Data from the LEP collider are included in this tune.
2. Herwig++ [28] is used to generate samples for evaluating systematic uncertainties. This generator uses a $2 \rightarrow 2$ matrix element and angular-ordered parton showers in the LL approximation [29–31]. The cluster model [32] is employed for the hadronisation. The underlying event and soft inclusive interactions are described using a hard and soft MPI model [33]. The parton densities are provided by the MRST LO** PDF set.
3. MadGraph [34] with the CTEQ6L1 PDF set [35] is used to generate proton–proton collision samples with up to three outgoing partons from the matrix element and with MLM matching [36] applied in the parton shower, which is performed with Pythia using the AUET2B tune.

### *Z*-jet and ${\gamma }$-jet Monte Carlo simulation samples

1. Pythia (version 6.425) is used to produce $Z$-jet events with the modified leading-order PDF set MRST LO**. The simulation uses a $2 \rightarrow 1$ matrix element to model the hard sub-process, and, as for the inclusive jet simulation, $p_{\mathrm {T}}$-ordered parton showers to model additional parton radiation in the LL approximation. In addition, weights are applied to the first branching of the shower, so as to bring agreement with the matrix-element rate in the hard emission region. The same tune and PDF is used as for the inclusive jet sample.
2. The Alpgen generator (version 2.13) [37] is used to produce $Z$-jet events, interfaced to Herwig (version 6.510) [31] for parton shower and fragmentation into particles, and to Jimmy (version 4.31) [38] to model UE contributions using the ATLAS AUET2 tune [39], here with the CTEQ6L1 [35] leading-order PDF set. Alpgen is a leading-order matrix-element generator for hard multi-parton processes ($2\rightarrow n$) in hadronic collisions. Parton showers are matched to the matrix element with the MLM matching scheme. The CTEQ6L1 PDF set is employed.
3. The baseline $\gamma \text {-jet}$ sample is produced with Pythia (version 6.425). It generates non-diffractive events using a $2 \rightarrow 2$ matrix element at leading order in $\alpha _{\mathrm {S}}$ to model the hard sub-process. Again, additional parton radiation is modelled by $p_{\mathrm {T}}$-ordered parton showers in the LL approximation. The modelling of non-perturbative physics effects arising in MPI, fragmentation, and hadronisation is based on the ATLAS AUET2B MRST LO** tune.
4. An alternative $\gamma \text {-jet}$ event sample is generated with Herwig (version 6.510) and Jimmy using the ATLAS AUET2 tune and the MRST LO** PDF. It is used to evaluate the systematic uncertainty due to physics modelling.
5. The systematic uncertainty from jets which are misidentified as photons (fake photons) is studied with a dedicated MC event sample. An inclusive jet sample is generated with Pythia (version 6.425) with the same parameter tuning and PDF set as the $\gamma \text {-jet}$ sample. An additional filter is applied to the jets built from the stable generated particles to select events containing a narrow particle jet, which is more likely to pass photon identification criteria. The surviving events are passed through the same detector simulation software as the MC $\gamma \text {-jet}$ sample.

### Top-quark pair Monte Carlo simulation samples

Top pair ($t\bar{t}$) production samples are relevant for jet reconstruction performance studies, as they are a significant source of hadronically decaying $W$ bosons and therefore important for light-quark jet response evaluations in a radiation environment very different from the inclusive jet and $Z$-jet/$\gamma \text {-jet}$ samples discussed above. In addition, they provide jets from a heavy-flavour ($b$-quark) decay, the response to which can be studied in this final state as well.

The nominal $t\bar{t}$ event sample is generated using MC@NLO (version 4.01) [40], which implements a next-to-leading-order (NLO) matrix element for top-pair production. Correspondingly, the CT10 [41] NLO PDF set is used. This matrix-element generator is interfaced to parton showers from Herwig (version 6.520) [42] and the underlying event modelled by Jimmy (version 4.31), with the CT10 PDF and the ATLAS AUET2 tune.

A number of systematic variation samples use alternative MC generators or different generator parameter sets. Additional $t\bar{t}$ samples are simulated using the POWHEG [43] generator interfaced with Pythia, as well as Herwig and Jimmy. POWHEG provides alternative implementations of the NLO matrix-element calculation and the interface to parton showers. These samples allow comparison of two different parton shower, hadronisation and fragmentation models. In addition, the particular implementations of the NLO matrix-element calculations in POWHEG and MC@NLO can be compared. Differences in the $b$-hadron decay tables between Pythia and Herwig are also significant enough to provide a conservative uncertainty envelope on the effects of the decay model.

In addition, samples with more or less parton shower activity are generated with the leading-order generator ACERMC [44] interfaced to Pythia with the MRST LO** PDF set. These are used to estimate the model dependence of the event selection. In these samples the initial state radiation (ISR) and the final state radiation (FSR) parameters are varied in value ranges not excluded by the current experimental data, as detailed in Refs. [45, 46].

**Fig. 1:**
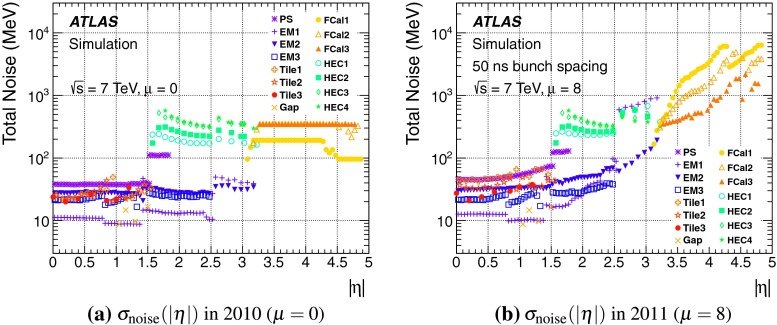
The energy-equivalent cell noise in the ATLAS calorimeters on the electromagnetic (EM) scale as a function of the direction $|\eta |$ in the detector, for the 2010 configuration with **a** $\mu = 0$ and the 2011 configuration with **b** $\mu = 8$. The various *colours* indicate the noise in the pre-sampler (PS) and the up to three layers of the LAr EM calorimeter, the up to three layers of the Tile calorimeter, the four layers for the hadronic end-cap (HEC) calorimeter, and the three modules of the forward (FCal) calorimeter

### Minimum bias samples

Minimum bias events are generated using Pythia8 [47] with the 4C tune [48] and MRST LO** PDF set. These minimum bias events are used to form pile-up events, which are overlaid onto the hard-scatter events following a Poisson distribution around the average number $\langle \mu \rangle$ of additional proton–proton collisions per bunch crossing measured in the experiment. The LHC bunch train structure with 36 proton bunches per train and 50 ns spacing between the bunches, is also modelled by organising the simulated collisions into four such trains. This allows the inclusion of out-of-time pile-up effects driven by the distance of the hard-scatter events from the beginning of the bunch train. The first ten bunch crossings in each LHC bunch train, approximately, are characterised by varying out-of-time pile-up contributions from the collision history, which is getting filled with an increasing number of bunch crossings with proton–proton interactions. For the remaining $\approx$26 bunch crossings in a train, the effect of the out-of-time pile-up contribution is stable, i.e. it does not vary with the bunch position within the bunch train, if the bunch-to-bunch intensity is constant. Bunch-to-bunch fluctuations in proton intensity at the LHC are not included in the simulation.

### Detector simulation

The Geant4 software toolkit [49] within the ATLAS simulation framework [50] propagates the stable particles^3^ produced by the event generators through the ATLAS detector and simulates their interactions with the detector material. Hadronic showers are simulated with the QGSP_BERT model [51–59]. Compared to the simulation used in the context of the 2010 data analysis, a newer version of Geant4 (version 9.4) is used and a more detailed description of the geometry of the LAr calorimeter absorber structure is available. These geometry changes introduce an increase in the calorimeter response to pions below 10 GeV of about 2 %.

For the estimation of the systematic uncertainties arising from detector simulation, several samples are also produced with the ATLAS fast (parameterised) detector simulation ATLFAST2 [50, 60].

## Dataset

The data used in this study were recorded by ATLAS between May and October 2011, with all ATLAS subdetectors operational. The corresponding total integrated luminosity is about $4.7$ $\,\,\text{ fb }^{-1}$ of proton–proton collisions at a centre-of-mass energy of $\sqrt{s}=7$ TeV.

As already indicated in Sect. 3.4, the LHC operated with bunch crossing intervals of 50 ns, and bunches organised in bunch trains. The average number of interactions per bunch crossing ($\mu$) as estimated from the luminosity measurement is $3 \le \mu \le 8$ until Summer 2011, with an average for this period of $\langle \mu \rangle \approx 6$. Between August 2011 and the end of the proton run, $\mu$ increased to about $5 \le \mu \le 17$, with an average $\langle \mu \rangle \approx 12$. The average number of interactions for the whole 2011 dataset is $\langle \mu \rangle = 8$.

The specific trigger requirements and precision signal object selections applied to the data are analysis dependent. They are therefore discussed in the context of each analysis presented in this paper.

## Jet reconstruction and calibration with the ATLAS detector

### Topological clusters in the calorimeter

Clusters of energy deposits in the calorimeter (topo-clusters) are built from topologically connected calorimeter cells that contain a significant signal above noise, see Refs. [3, 61, 62] for details. The topo-cluster formation follows cell signal significance patterns in the ATLAS calorimeters. The signal significance is measured by the absolute ratio of the cell signal to the energy-equivalent noise in the cell. The signal-to-noise thresholds for the cluster formation are not changed with respect to the settings given in Ref. [3]. However, the noise in the calorimeter increased due to the presence of multiple proton-proton interactions, as discussed in Sect. 2.2, and required the adjustments explained below.

While in ATLAS operations prior to 2011 the cell noise was dominated by electronic noise, the short bunch crossing interval in 2011 LHC running added a noise component from bunch-to-bunch variations in the instantaneous luminosity and in the energy deposited in a given cell from previous collisions inside the window of sensitivity of the calorimeters. The cell noise thresholds steering the topo-cluster formation thus needed to be increased from those used in 2010 to accommodate the corresponding fluctuations, which is done by raising the nominal noise according to

$$\begin{aligned} \sigma _{\text {noise}}= \left\{ \begin{array}{l@{\quad }l} \sigma _{\text {noise}}^{\text {electronic}}&{} \mathrm{(2010~operations)} \\ \sqrt{\left( \sigma _{\text {noise}}^{\text {electronic}}\right) ^{2} + \left( \sigma _{\text {noise}}^{\text {pile-up}}\right) ^{2}}&{}\mathrm{(2011~operations)} \end{array}\right. . \end{aligned}$$

Here, $\sigma _{\text {noise}}^{\text {electronic}}$ is the electronic noise, and $\sigma _{\text {noise}}^{\text {pile-up}}$ the noise from pile-up, determined with MC simulations and corresponding to an average of eight additional proton–proton interactions per bunch crossing ($\mu = 8$) in 2011. The change of the total nominal noise $\sigma _{\text {noise}}$ and its dependence on the calorimeter region in ATLAS can be seen by comparing Fig. 1a and b. In most calorimeter regions, the noise induced by pile-up is smaller than or of the same magnitude as the electronic noise, with the exception of the forward calorimeters, where $\sigma _{\text {noise}}^{\text {pile-up}}\gg \sigma _{\text {noise}}^{\text {electronic}}$.

The implicit noise suppression implemented by the topological cluster algorithm discussed above leads to significant improvements in the calorimeter performance for e.g. the energy and spatial resolutions in the presence of pile-up. On the other hand, contributions from larger negative and positive signal fluctuations introduced by pile-up can survive in a given event. They thus contribute to the sensitivity to pile-up observed in the jet response, in addition to the cell-level effects mentioned in Sect. 2.2.

**Fig. 2:**
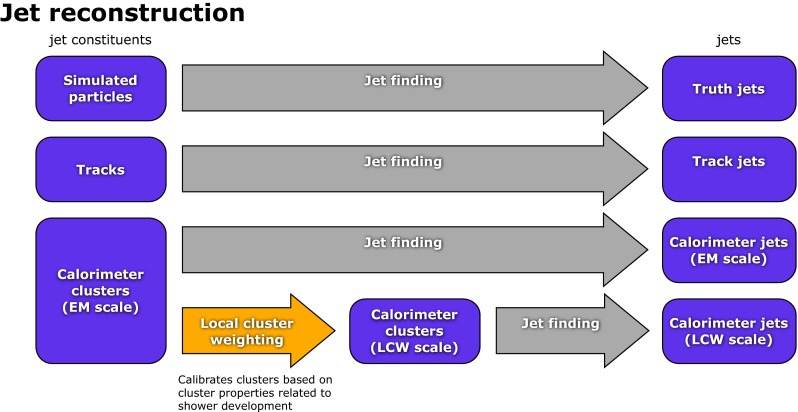
Overview of the ATLAS jet reconstruction. After the jet finding, the jet four momentum is defined as the four momentum sum of its constituents

**Fig. 3:**
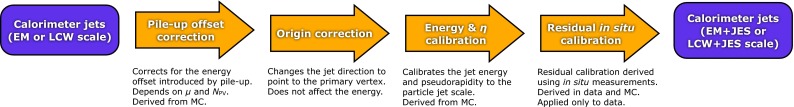
Overview of the ATLAS jet calibration scheme used for the 2011 dataset. The pile-up, absolute JES and the residual in situ corrections calibrate the scale of the jet, while the origin and the $\eta$ corrections affect the direction of the jet

### Jet reconstruction and calibration

Jets are reconstructed using the anti-$k_{t}$ algorithm [1] with distance parameters $R = 0.4$ or $R = 0.6$, utilising the FastJet software package [63, 64]. The four-momentum scheme is used at each recombination step in the jet clustering. The total jet four-momentum is therefore defined as the sum of the four-momenta sum of all its constituents. The inputs to the jet algorithm are stable simulated particles (*truth jets*, see Sect. 5.5 for details), reconstructed tracks in the inner detector (*track jets*, see Ref. [3] and Sect. 5.4 for details) or energy deposits in the calorimeter (*calorimeter jets*, see below for details). A schematic overview of the ATLAS jet reconstruction is presented in Fig. 2.

The calorimeter jets are built from the topo-clusters entering as massless particles in the jet algorithm as discussed in the previous section. Only clusters with positive energy are considered. The topo-clusters are initially reconstructed at the EM scale [61, 65–72], which correctly measures the energy deposited in the calorimeter by particles produced in electromagnetic showers. A second topo-cluster collection is built by calibrating the calorimeter cell such that the response of the calorimeter to hadrons is correctly reconstructed. This calibration uses the local cell signal weighting (LCW) method that aims at an improved resolution compared to the EM scale by correcting the signals from hadronic deposits, and thus reduces fluctuations due to the non-compensating nature of the ATLAS calorimeter. The LCW method first classifies topo-clusters as either electromagnetic or hadronic, primarily based on the measured energy density and the longitudinal shower depth. Energy corrections are derived according to this classification from single charged and neutral pion MC simulations. Dedicated corrections address effects of calorimeter non-compensation, signal losses due to noise threshold effects, and energy lost in non-instrumented regions close to the cluster [3].

**Fig. 4:**
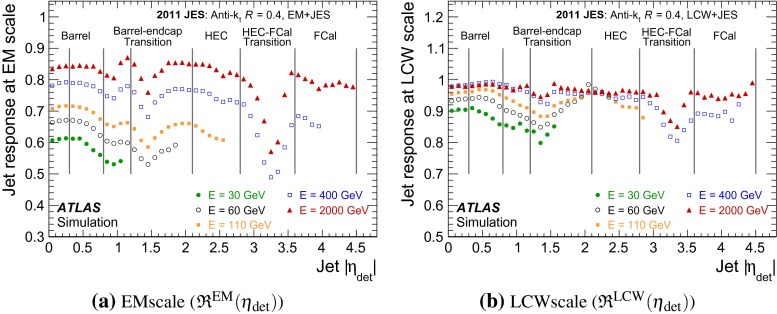
Average response of simulated jets formed from topo-clusters, calculated as defined in Eq. (1) and shown in **a** for the EM scale ($\mathfrak {R}^{\mathrm{EM}}$) and in **b** for the LCW scale ($\mathfrak {R}^{\mathrm{LCW}}$). The response is shown separately for various truth-jet energies as function of the uncorrected (detector) jet pseudorapidity $\eta _\mathrm{det}$. Also indicated are the different calorimeter regions. The inverse of $\mathfrak {R}^{\mathrm{EM}}$ ($\mathfrak {R}^{\mathrm{LCW}}$) corresponds to the average jet energy scale correction for EM (LCW) in each $\eta _\mathrm{det}$ bin. The results shown are based on the baseline Pythia inclusive jet sample

Figure 3 shows an overview of the ATLAS calibration scheme for calorimeter jets used for the 2011 dataset, which restores the jet energy scale to that of jets reconstructed from stable simulated particles (truth particle level, see Sect. 5.5). This procedure consists of four steps as described below.

1. **Pile-up correction** Jets formed from topo-clusters at the EM or LCW scale are first calibrated by applying a correction to account for the energy offset caused by pile-up interactions. The effects of pile-up on the jet energy scale are caused by both additional proton collisions in a recorded event (in-time pile-up) and by past and future collisions influencing the energy deposited in the current bunch-crossing (out-of-time pile-up), and are outlined in Sect. 6. This correction is derived from MC simulations as a function of the number of reconstructed primary vertices ($N_{\mathrm{PV}}$, measuring the actual collisions in a given event) and the expected average number of interactions ($\mu$, sensitive to out-of-time pile-up) in bins of jet pseudorapidity and transverse momentum (see Sect. 6).
2. **Origin correction** A correction to the calorimeter jet direction is applied that makes the jet pointing back to the primary event vertex instead of the nominal centre of the ATLAS detector.
3. **Jet calibration based on MC simulations** Following the strategy presented in Ref. [3], the calibration of the energy and pseudorapidity of a reconstructed jet is a simple correction derived from the relation of these quantities to the corresponding ones of the matching truth jet (see Sect. 5.5) in MC simulations. It can be applied to jets formed from topo-clusters at EM or at LCW scale with the resulting jets being referred to as calibrated with the EM+JES or with the LCW+JES scheme. This first JES correction uses isolated jets from an inclusive jet MC sample including pile-up events (the baseline sample described in Sect. 3). Figure 4 shows the average energy response
  1 $$\begin{aligned} \mathfrak {R}^{\mathrm{EM}(\mathrm{LCW})}=E^{\mathrm{EM}(\mathrm{LCW})}_\mathrm{jet}/E^\mathrm{truth}_\mathrm{jet}, \end{aligned}$$
  which is the inverse of the jet energy calibration function, for various jet energies as a function of the jet pseudorapidity $\eta _\mathrm{det}$ measured in the detector frame of reference (see Sect. 5.6).
4. **Residual in situ corrections** A residual correction derived in situ is applied as a last step to jets reconstructed in data. The derivation of this correction is described in Sect. 7.

**Fig. 5:**
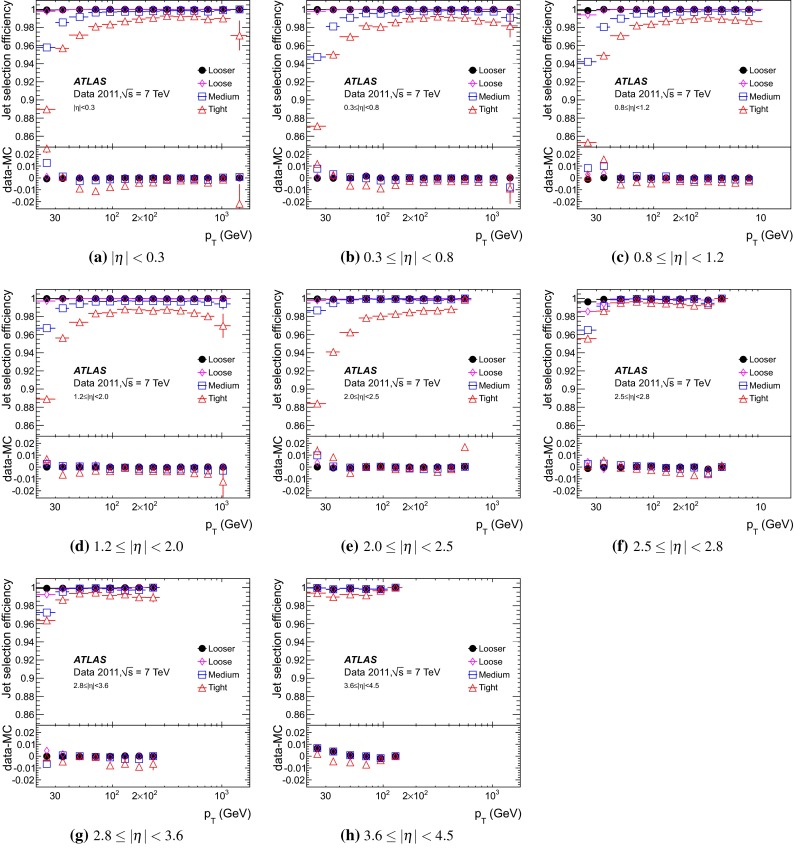
Jet quality selection efficiency for anti-$k_{t}$ jets with $R = 0.4$ measured with a tag-and-probe technique as a function of $p_{\mathrm {T}}^\mathrm {jet}$ in various $\eta$ ranges, for the four sets of selection criteria. Only statistical uncertainties are shown. Differences between data and MC simulations are also shown

### Jet quality selection

Jets with high transverse momenta produced in proton–proton collisions must be distinguished from background jet candidates not originating from hard-scattering events. A first strategy to select jets from collisions and to suppress background is presented in Ref. [3].

The main sources of potential background are:

1. Beam-gas events, where one proton of the beam collides with the residual gas within the beam pipe.
2. Beam-halo events, for example caused by interactions in the tertiary collimators in the beam-line far away from the ATLAS detector.
3. Cosmic-ray muons overlapping in-time with collision events.
4. Calorimeter noise.

The jet quality selection criteria should efficiently reject jets from these background processes while maintaining high efficiency for selecting jets produced in proton–proton collisions. Since the level and composition of background depend on the event topology and the jet kinematics, four sets of criteria called Looser, Loose, Medium and Tight are introduced in Ref. [73]. They correspond to different levels of fake-jet rejection and jet selection efficiency, with the Looser criterion being the one with the highest jet selection efficiency while the Tight criterion is the one with the best rejection. The discrimination between jets coming from the collisions and background jet candidates is based on several pieces of experimental information, including the quality of the energy reconstruction at the cell level, jet energy deposits in the direction of the shower development, and reconstructed tracks matched to the jets.

The efficiencies of the jet selection criteria are measured using the tag-and-probe method described in Ref. [3]. The resulting efficiencies for anti-$k_{t}$ jets with $R = 0.4$ for all selection criteria are shown in Fig. 5. The jet selection efficiency of the Looser selection is greater than 99.8 % over all calibrated transverse jet momenta $p_{\mathrm {T}}^\mathrm {jet}$ and $\eta$ bins. A slightly lower efficiency of about 1–2 % is measured for the Loose selection, in particular at low $p_{\mathrm {T}}^\mathrm {jet}$ and for $2.5 <|\eta |< 3.6$. The Medium and Tight selections have lower jet selection efficiencies mainly due to cuts on the jet charged fraction, which is the ratio of the scalar sum of the $p_{\mathrm {T}}$ of all reconstructed tracks matching the jet, and the jet $p_{\mathrm {T}}$ itself, see Ref. [73] for more details. For jets with $p_{\mathrm {T}}^\mathrm {jet}\approx 25$ GeV, the Medium and Tight selections have inefficiencies of 4 and 15 %, respectively. For $p_{\mathrm {T}}^\mathrm {jet}> 50$ GeV, the Medium and Tight selections have efficiencies greater than 99 and 98 %, respectively.

The event selection is based on the azimuthal distance between the probe and tag jet $\Delta \phi (\mathrm {tag},\mathrm {probe})$ and the significance of the missing transverse momentum ${E}_{\mathrm {T}}^{\mathrm {miss}}$ [74] reconstructed for the event, which is measured by the ratio ${E}_{\mathrm {T}}^{\mathrm {miss}}/\sqrt{\Sigma E_{\mathrm {T}}}$. Here $\Sigma E_{\mathrm {T}}$ is the scalar transverse momentum sum of all particles, jets, and soft signals in the event. The angle $\Delta \phi (\mathrm {tag},\mathrm {probe})$, ${E}_{\mathrm {T}}^{\mathrm {miss}}/\sqrt{\Sigma E_{\mathrm {T}}}$, and the Tight selection of the reference (tag) jet are varied to study the systematic uncertainties. For the Loose and Looser selections, the jet selection efficiency is almost unchanged by varying the selection cuts, with variations of less than 0.05 %. Slightly larger changes are observed for the two other selections, but they are not larger than 0.1 % for the Medium and 0.5 % for the Tight selection.

The jet selection efficiency is also measured using a MC simulation sample. A very good agreement between data and simulation is observed for the Looser and Loose selections. Differences not larger than 0.2 and 1 % are observed for the Medium and Tight selections, respectively, for $p_{\mathrm {T}}^\mathrm {jet}> 40$ GeV. Larger differences are observed at lower $p_{\mathrm {T}}^\mathrm {jet}$, but they do not exceed 1 % (2 %) for the Medium(Tight) selection.

### Track jets

In addition to the previously described calorimeter jets reconstructed from topo-clusters, track jets in ATLAS are built from reconstructed charged particle tracks associated with the reconstructed primary collision vertex, which is defined by

$$\begin{aligned} \sum (p_{\mathrm {T}}^{\mathrm {track}})^{2} = \max . \end{aligned}$$

Here $p_{\mathrm {T}}^{\mathrm {track}}$ is the transverse momentum of tracks pointing to a given vertex. The tracks associated with the primary vertex are required to have $p_{\mathrm {T}}^{\mathrm {track}}> 500$  MeV and to be within $|\eta | < 2.5$. Additional reconstruction quality criteria are applied, including the number of hits in the pixel detector (at least one) and in the silicon microstrip detector (at least six) of the ATLAS ID system. Further track selections are based on the transverse ($d_{0}$, perpendicular to the beam axis) and longitudinal ($z_{0}$, along the beam axis) impact parameters of the tracks measured with respect to the primary vertex ($|d_{0}|<1.5$ mm, $|z_{0}\sin \theta |<1.5$ mm). Here $\theta$ is the polar angle of the track.

Generally, track jets used in the studies presented in this paper are reconstructed with the same configurations as calorimeter jets, i.e. using the anti-$k_{t}$ algorithm with $R = 0.4$ and $R = 0.6$. As only tracks originating from the hardest primary vertex in the collision event are used in the jet finding, the transverse momentum of any of these track jets provides a rather stable kinematic reference for matching calorimeter jets, as it is independent of the pile-up activity. Track jets can of course only be formed within the tracking detector coverage ($|\eta |<{2.5}$), yielding an effective acceptance for track jets of $|\eta _{\mathrm {track jet}}| < 2.5 - R$.

Certain studies may require slight modifications of the track selection and the track-jet formation criteria and algorithms. Those are indicated in the respective descriptions of the applied methods. In particular, track jets may be further selected by requirements concerning the number of clustered tracks, the track-jet $p_{\mathrm {T}}$, and the track-jet direction.

### Truth jets

Truth jets can be formed from stable particles generated in MC simulations. In general those are particles with a lifetime $\tau$ defined by $c \tau > 10$ mm [75]. The jet definitions applied are the same as the ones used for calorimeter and track jets (anti-$k_{t}$ with distance parameters $R = 0.4$ and $R = 0.6$, respectively). If truth jets are employed as a reference for calibrations purposes in MC simulations, neither final-state muons nor neutrinos are included in the stable particles considered for its formation. The simulated calorimeter jets are calibrated with respect to truth jets consisting of stable particles leaving an observable signal (*visible energy*) in the detector.^4^ This is a particular useful strategy for inclusive jet measurements and the universal jet calibration discussed in this paper, but special truth-jet references including muons and/or neutrinos may be utilised as well, in particular to understand the heavy-flavour jet response, as discussed in detail in Sect. 19.

### Jet kinematics and directions

Kinematic properties of jets relevant for their use in final-state selections and final-state reconstruction are the transverse momentum $p_{\mathrm {T}}$ and the rapidity $y$. The full reconstruction of the jet kinematics including these variables takes into account the physics frame of reference, which in ATLAS is defined event-by-event by the primary collision vertex discussed in Sect. 5.4.

On the other hand, many effects corrected by the various JES calibrations discussed in this paper are highly localised, i.e. they are due to specific detector features and inefficiencies at certain directions or ranges. The relevant directional variable to use as a basis for these corrections is then the detector pseudorapidity $\eta _\mathrm{det}$, which is reconstructed in the nominal detector frame of reference in ATLAS, and is centred at the nominal collision vertex $(x = 0,y = 0,z=0)$.

Directional relations to jets, and e.g. between the constituents of jet and its principal axis, can then be measured either in the physics or the detector reference frame, with the choice depending on the analysis. In the physics reference frame (($y,\phi$) space) the distance between any two objects is given by

2 $$\begin{aligned} \Delta R= \sqrt{(\Delta y)^{2} + (\Delta \phi )^{2}} , \end{aligned}$$

where $\Delta y$ is the rapidity distance and $\Delta \phi$ is the azimuthal distance between them. The same distance measured in the detector frame of reference (($\eta ,\phi$) space) is calculated as

3 $$\begin{aligned} \Delta R= \sqrt{(\Delta \eta )^{2} + (\Delta \phi )^{2}} , \end{aligned}$$

where $\Delta \eta$ is the distance in pseudorapidity between any two objects. In case of jets and their constituents (topo-clusters or tracks), $\eta = \eta _\mathrm{det}$ is used. All jet clustering algorithms used in ATLAS apply the physics frame distance in Eq. (2) in their distance evaluations, as jets are considered to be massive physical objects, and the jet clustering is intended to follow energy flow patterns introduced by the physics of parton showers, fragmentation, and hadronisation from a common (particle) source. In this context topo-clusters and reconstructed tracks are considered *pseudo-particles* representing the true particle flow within the limitations introduced by the respective detector acceptances and resolutions.

**Fig. 6:**
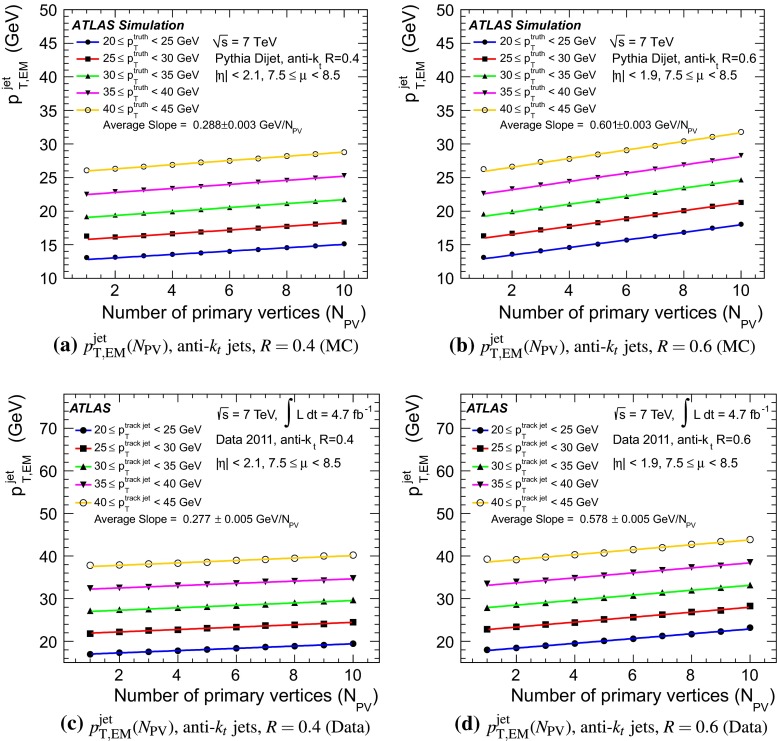
The average reconstructed transverse momentum $p_\mathrm{T, \mathrm{EM}}^\mathrm {jet}$ on EM scale for jets in MC simulations, as function of the number of reconstructed primary vertices $N_{\mathrm{PV}}$ and $7.5 \le \mu < 8.5$, in various bins of truth-jet transverse momentum $p_{\mathrm {T}}^\mathrm {truth}$, for jets with **a** $R =0.4$ and **b** $R = 0.6$. The dependence of $p_\mathrm{T, \mathrm{EM}}^\mathrm {jet}$ on $N_{\mathrm{PV}}$ in data, in bins of track-jet transverse momentum $p_{\mathrm {T}}^{\mathrm {track}}$, is shown in **c** for $R = 0.4$ jets, and in **d** for $R = 0.6$ jets

## Jet energy correction for pile-up interactions

### Pile-up correction method

The pile-up correction method applied to reconstructed jets in ATLAS is derived from MC simulations and validated with in situ and simulation based techniques. The approach is to calculate the amount of transverse momentum generated by pile-up in a jet in MC simulation, and subtract this offset $\mathcal {O}$ from the reconstructed jet $p_{\mathrm {T}}^{\mathrm {jet}}$ at any given signal scale (EM or LCW). At least to first order, pile-up contributions to the jet signal can be considered stochastic and diffuse with respect to the true jet signal. Therefore, both in-time and out-of-time pile-up are expected to depend only on the past and present pile-up activity, with linear relations between the amount of activity and the pile-up signal.

### Principal pile-up correction strategy

To characterise the in-time pile-up activity, the number of reconstructed primary vertices ($N_{\mathrm{PV}}$) is used. The ATLAS tracking detector timing resolution allows the reconstruction of only in-time tracks and vertices, so that $N_{\mathrm{PV}}$ provides a good measure of the actual number of proton–proton collisions in a recorded event.

For the out-of-time pile-up activity, the average number of interactions per bunch crossing ($\mu$) at the time of the recorded events provides a good estimator. It is derived by averaging the actual number of interactions per bunch crossing over a rather large window $\Delta t$ in time, which safely encompasses the time interval during which the ATLAS calorimeter signal is sensitive to the activity in the collision history ($\Delta t \gg 600$ ns for the liquid-argon calorimeters). The observable $\mu$ can be reconstructed from the average luminosity $L$ over this period $\Delta t$, the total inelastic proton–proton cross section ($\sigma _{\mathrm {inel}} = 71.5$ mb [76]), the number of colliding bunches in LHC ($N_{\mathrm {bunch}}$) and the LHC revolution frequency ($f_{\mathrm {LHC}}$) (see Ref. [77] for details):

$$\begin{aligned} \mu = \frac{L \times \sigma _{\mathrm {inel}}}{N_{\mathrm {bunch}} \times f_{\mathrm {LHC}}} . \end{aligned}$$

The MC-based jet calibration is derived for a given (reference) pile-up condition^5^ $(N_{\mathrm{PV}}^\mathrm{ref},\mu ^{\mathrm {ref}})$ such that $\mathcal {O}(N_{\mathrm{PV}}= N_{\mathrm{PV}}^\mathrm{ref},$ $\mu = \mu ^{\mathrm {ref}}) = 0$. As the amount of energy scattered into a jet by pile-up and the signal modification imposed by the pile-up history determine $\mathcal {O}$, a general dependence on the distances from the reference point is expected. From the nature of pile-up discussed earlier, the linear scaling of $\mathcal {O}$ in both $N_{\mathrm{PV}}$ and $\mu$ provides the ansatz for a correction,

4 $$\begin{aligned}&\mathcal {O}(N_{\mathrm{PV}},\mu ,\eta _\mathrm{det}) = p_{\mathrm {T}}^{\mathrm {jet}}(N_{\mathrm{PV}},\mu ,\eta _\mathrm{det}) - p_{\mathrm {T}}^\mathrm {truth}\nonumber \\&\quad = \frac{\partial p_{\mathrm {T}}}{\partial N_{\mathrm{PV}}}(\eta _\mathrm{det}) \left( N_{\mathrm{PV}}- N_{\mathrm{PV}}^\mathrm{ref}\right) + \frac{\partial p_{\mathrm {T}}}{\partial {\mu }}(\eta _\mathrm{det}) \left( \mu - \mu ^{\mathrm {ref}}\right) \nonumber \\&\quad = \alpha (\eta _\mathrm{det})\cdot \left( N_{\mathrm{PV}}- N_{\mathrm{PV}}^\mathrm{ref}\right) + \beta (\eta _\mathrm{det})\cdot \left( \mu - \mu ^{\mathrm {ref}}\right) \end{aligned}$$

Here, $p_{\mathrm {T}}^{\mathrm {jet}}(N_{\mathrm{PV}},\mu ,\eta _\mathrm{det})$ is the reconstructed transverse momentum of the jet (without the JES correction described in Sect. 5.2 applied) in a given pile-up condition ($N_{\mathrm{PV}}$,$\mu$) and at a given direction $\eta _\mathrm{det}$ in the detector. The true transverse momentum of the jet ($p_{\mathrm {T}}^\mathrm {truth}$) is available from the generated particle jet matching a reconstructed jet in MC simulations. The coefficients $\alpha (\eta _\mathrm{det})$ and $\beta (\eta _\mathrm{det})$ depend on $\eta _\mathrm{det}$, as both in-time and out-of-time pile-up signal contributions manifest themselves differently in different calorimeter regions, according to the following influences:

1. The energy flow from collisions into that region.
2. The calorimeter granularity and occupancy after topo-cluster reconstruction, leading to different acceptances at cluster level and different probabilities for multiple particle showers to overlap in a single cluster.
3. The effective sensitivity to out-of-time pile-up introduced by different calorimeter signal shapes.

The offset $\mathcal {O}$ can be determined in MC simulation for jets on the EM or the LCW scale by using the corresponding reconstructed transverse momentum on one of those scales, i.e. $p_{\mathrm {T}}^{\mathrm {jet}} = p_{\mathrm {T,\mathrm{EM}}}^{\mathrm {jet}}$ or $p_{\mathrm {T}}^{\mathrm {jet}} = p_{\mathrm {T,\mathrm{LCW}}}^{\mathrm {jet}}$ in Eq. (4), and $p_{\mathrm {T}}^\mathrm {truth}$. The particular choice of scale affects the magnitude of the coefficients and, therefore, the transverse momentum offset itself,

$$\begin{aligned}&\mathcal {O}^{\mathrm{EM}} \mapsto \left\{ \alpha ^{\mathrm{EM}}(\eta _\mathrm{det}),\beta ^{\mathrm{EM}}(\eta _\mathrm{det})\right\} \\&\mathcal {O}^{\mathrm{LCW}} \mapsto \left\{ \alpha ^{\mathrm{LCW}}(\eta _\mathrm{det}),\beta ^{\mathrm{LCW}}(\eta _\mathrm{det})\right\} . \end{aligned}$$

The corrected transverse momentum of the jet at either of the two scales ($p_{\mathrm {T,\mathrm{EM}}}^{\mathrm {corr}}$ or $p_{\mathrm {T,\mathrm{LCW}}}^{\mathrm {corr}}$) is then given by

5 $$\begin{aligned}&p_{\mathrm {T,\mathrm{EM}}}^{\mathrm {corr}} = p_{\mathrm {T,\mathrm{EM}}}^{\mathrm {jet}} - \mathcal {O}^{\mathrm{EM}}(N_{\mathrm{PV}},\mu ,\eta _\mathrm{det}) \end{aligned}$$

6 $$\begin{aligned}&p_{\mathrm {T,\mathrm{LCW}}}^{\mathrm {corr}} = p_{\mathrm {T,\mathrm{LCW}}}^{\mathrm {jet}} - \mathcal {O}^{\mathrm{LCW}}(N_{\mathrm{PV}},\mu ,\eta _\mathrm{det}) . \end{aligned}$$

After applying the correction, the original $p_{\mathrm {T,\mathrm{EM}}}^{\mathrm {jet}}$ and $p_{\mathrm {T,\mathrm{LCW}}}^{\mathrm {jet}}$ dependence on $N_{\mathrm{PV}}$ and $\mu$ is expected to vanish in the corresponding corrected $p_{\mathrm {T,\mathrm{EM}}}^{\mathrm {corr}}$ and $p_{\mathrm {T,\mathrm{LCW}}}^{\mathrm {corr}}$.

**Fig. 7:**
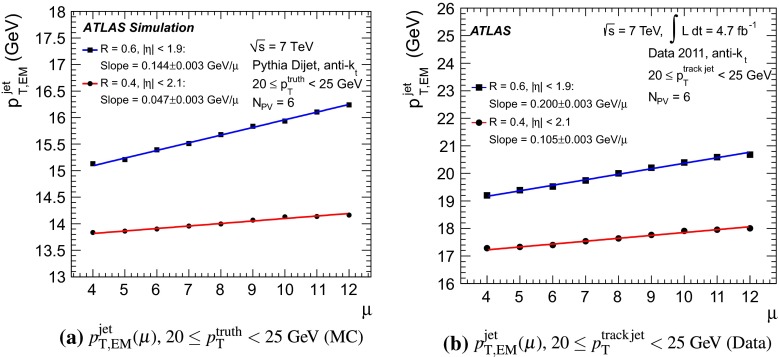
The average reconstructed jet transverse momentum $p_\mathrm{T, \mathrm{EM}}^\mathrm {jet}$ on EM scale as function of the average number of collisions $\mu$ at a fixed number of primary vertices $N_{\mathrm{PV}}= 6$, for truth jets in MC simulation **a** in the lowest bin of $p_{\mathrm {T}}^\mathrm {truth}$ and **b** in the lowest bin of track jet transverse momentum $p_{\mathrm {T}}^{\mathrm {track\ jet}}$ considered in data

### Derivation of pile-up correction parameters

Figure 6a and b shows the dependence of $p_{\mathrm {T,\mathrm{EM}}}^{\mathrm {jet}}$, and thus $\mathcal {O}^{\mathrm{EM}}$, on $N_{\mathrm{PV}}$. In this example, narrow ($R = 0.4$, $\left| \eta _\mathrm{det}\right| < 2.1$) and wide ($R = 0.6$, $\left| \eta _\mathrm{det}\right| < 1.9$) central jets reconstructed in MC simulation are shown for events within a given range $7.5 \le \mu < 8.5$. The jet $p_{\mathrm {T}}$ varies by $0.277 \pm 0.005$ GeV(in data) and $0.288 \pm 0.003$ GeV(in MC simulations) per primary vertex for jets with $R=0.4$ and by $0.578 \pm 0.005$ GeV(in data) and $0.601 \pm 0.003$ GeV(in MC simulations) per primary vertex for jets with $R=0.6$. The slopes $\alpha ^{\mathrm{EM}}$ are found to be independent of the true jet transverse momentum $p_{\mathrm {T}}^\mathrm {truth}$, as expected from the diffuse character of in-time pile-up signal contributions.

A qualitatively similar behaviour can be observed in collision data for calorimeter jets individually matched with track jets, the latter reconstructed as discussed in Sect. 5.4. The $N_{\mathrm{PV}}$ dependence of $p_{\mathrm {T,\mathrm{EM}}}^{\mathrm {jet}}$ can be measured in bins of the track-jet transverse momentum $p_{\mathrm {T}}^{\mathrm {track\ jet}}$. Jets formed from tracks are much less sensitive to pile-up and can be used as a stable reference to investigate pile-up effects. Figure 6c and d shows the results for the same calorimeter regions and out-of-time pile-up condition as for the MC-simulated jets in Fig. 6a, b. The results shown in Fig. 6 also confirm the expectation that the contributions from in-time pile-up to the jet signal are larger for wider jets ($\alpha ^{\mathrm{EM}}(R = 0.6) > \alpha ^{\mathrm{EM}}(R = 0.4)$), but scale only approximately with the size of the jet catchment area [78] determined by the choice of distance parameter $R$ in the anti-$k_{t}$ algorithm.

**Fig. 8:**
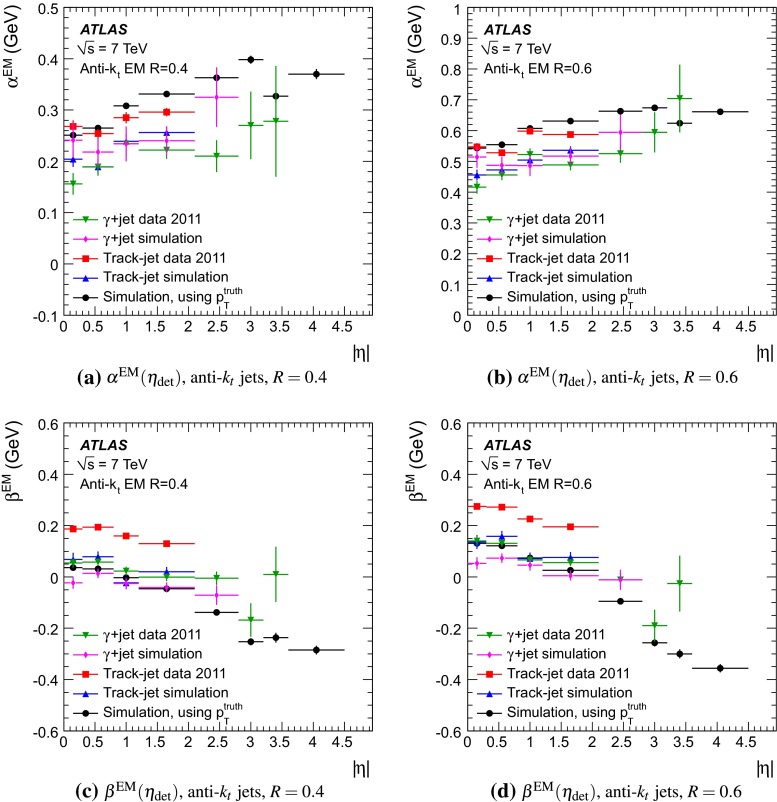
The pile-up contribution per additional vertex, measured as $\alpha ^{\mathrm{EM}}= \partial p_\mathrm{T, \mathrm{EM}}^\mathrm {jet}/\partial N_{\mathrm{PV}}$, as function of $|\eta _\mathrm{det}|$, for the various methods discussed in the text, for **a** $R = 0.4$ and **b** $R = 0.6$ jets. The contribution from $\mu$, calculated as $\beta ^{\mathrm{EM}}= \partial p_\mathrm{T, \mathrm{EM}}^\mathrm {jet}/\partial \mu$ and displayed for the various methods as function of $|\eta _\mathrm{det}|$, is shown for the two jet sizes in **c** and **d**, respectively. The points for the determination of $\alpha ^{\mathrm{EM}}$ and $\beta ^{\mathrm{EM}}$ from MC simulations use the offset calculated from the reconstructed $p_{\mathrm {T,\mathrm{EM}}}^{\mathrm {jet}}$ and the true (particle level) $p_{\mathrm {T}}^\mathrm {truth}$, as indicated in Eq. 4

The dependence of $p_\mathrm{T, \mathrm{EM}}^\mathrm {jet}$ on $\mu$, for a fixed $N_{\mathrm{PV}}= 6$, is shown in Fig. 7a for MC simulations using truth jets, and in Fig. 7b for collision data using track jets. The kinematic bins shown are the lowest bins considered, with $20 < p_{\mathrm {T}}^\mathrm {truth}< 25$ GeV and $20 < p_{\mathrm {T}}^{\mathrm {track\ jet}}< 25$ GeV for MC simulations and data, respectively. The jet $p_{\mathrm {T}}$ varies by $0.047 \pm 0.003$ GeV (in MC simulations) $0.105 \pm 0.003$ GeV (in data) per primary vertex for jets with $R=0.4$.

The result confirms the expectations that the dependence of $p_\mathrm{T, \mathrm{EM}}^\mathrm {jet}$ on the out-of-time pile-up is linear and significantly less than its dependence on the in-time pile-up contribution scaling with $N_{\mathrm{PV}}$. Its magnitude is still different for jets with $R = 0.6$, as the size of the jet catchment area again determines the absolute contribution to $p_\mathrm{T, \mathrm{EM}}^\mathrm {jet}$.

The correction coefficients for jets calibrated with the EM+JES scheme, $\alpha ^{\mathrm{EM}}$ and $\beta ^{\mathrm{EM}}$, are both determined from MC simulations as functions of the jet direction $\eta _\mathrm{det}$. For this, the $N_{\mathrm{PV}}$ dependence of $p_{\mathrm {T,\mathrm{EM}}}^{\mathrm {jet}}$($\eta _\mathrm{det}$) reconstructed in various bins of $\mu$ in the simulation is fitted and then averaged, yielding $\alpha ^{\mathrm{EM}}(\eta _\mathrm{det})$. Accordingly and independently, the dependence of $p_{\mathrm {T,\mathrm{EM}}}^{\mathrm {jet}}$ on $\mu$ is fitted in bins of $N_{\mathrm{PV}}$, yielding the average $\beta ^{\mathrm{EM}}(\eta _\mathrm{det})$, again using MC simulations. An identical procedure is used to find the correction functions $\alpha ^{\mathrm{LCW}}(\eta _\mathrm{det})$ and $\beta ^{\mathrm{LCW}}(\eta _\mathrm{det})$ for jets calibrated with the LCW+JES scheme.

The parameters $\alpha ^{\mathrm{EM}}$($\alpha ^{\mathrm{LCW}}$) and $\beta ^{\mathrm{EM}}$($\beta ^{\mathrm{LCW}}$) can be also measured with in situ techniques. This is discussed in Sect. 6.4.

### Pile-up validation with in situ techniques and effect of out-of-time pile-up in different calorimeter regions

The parameters $\alpha ^{\mathrm{EM}}$($\alpha ^{\mathrm{LCW}}$) and $\beta ^{\mathrm{EM}}$($\beta ^{\mathrm{LCW}}$) can be measured in data with respect to a reference that is stable under pile-up using track jets or photons in $\gamma \text {-jet}$ events as kinematic reference that does not depend on pile-up.

The variation of the $p_{\mathrm {T}}$ balance $p_{\mathrm {T,\mathrm{EM}}}^{\mathrm {jet}} - p_{\mathrm {T}}^{\gamma }$ ($p_{\mathrm {T,\mathrm{LCW}}}^{\mathrm {jet}} - p_{\mathrm {T}}^{\gamma }$) in $\gamma \text {-jet}$ events can be used in data and MC simulation (similarly to the strategy discussed in Sect. 10), as a function of $N_{\mathrm{PV}}$ and $\mu$. Figure 8 summarises $\alpha ^{\mathrm{EM}}(\eta _\mathrm{det})$ and $\beta ^{\mathrm{EM}}(\eta _\mathrm{det})$ determined with track jets and $\gamma \text {-jet}$ events, and their dependence on $\eta _\mathrm{det}$. Both methods suffer from lack of statistics or large systematic uncertainties in the 2011 data, but are used in data-to-MC comparisons to determine systematic uncertainties of the MC-based method (see the corresponding discussion in Sect. 16.2).

The decrease of $\beta ^{\mathrm{EM}}(\eta _\mathrm{det})$ towards higher $\eta _\mathrm{det}$, as shown in Fig. 8c and d, indicates a decreasing signal contribution to $p_{\mathrm {T,\mathrm{EM}}}^{\mathrm {jet}}$ per out-of-time pile-up interaction. For jets with $\left| \eta _\mathrm{det}\right| > 1.5$, the offset is increasingly suppressed in the signal with increasing $\mu$ ($\beta ^{\mathrm{EM}}(\eta _\mathrm{det})< 0$). This constitutes a qualitative departure from the behaviour of the pile-up history contribution in the central region of ATLAS, where this out-of-time pile-up leads to systematically increasing signal contributions with increasing $\mu$.

This is a consequence of two effects. First, for $\left| \eta _\mathrm{det}\right|$ larger than about 1.7 the hadronic calorimetry in ATLAS changes from the Tile calorimeter to the LAr end-cap (HEC) calorimeter. The Tile calorimeter has a unipolar and fast signal shape [20]. It has little sensitivity to out-of-time pile-up, with an approximate shape signal baseline of 150 ns. The out-of-time history manifests itself in this calorimeter as a small positive increase of its contribution to the jet signal with increasing $\mu$.

The HEC, on the other hand, has the typical ATLAS LAr calorimeter bipolar pulse shape with approximately 600 ns baseline. This leads to an increasing suppression of the contribution from this calorimeter to the jet signal with increasing $\mu$, as more activity from the pile-up history increases the contribution weighted by the negative pulse shape.

Second, for $\left| \eta _\mathrm{det}\right|$ larger than approximately 3.2, coverage is provided by the ATLAS forward calorimeter (FCal). While still a liquid-argon calorimeter, the FCal features a considerably faster signal due to very thin argon gaps. The shaping function for this signal is bipolar with a net zero integral and a similar positive shape as in other ATLAS liquid-argon calorimeters, but with a shorter overall pulse baseline (approximately 400 ns). Thus, the FCal shaping function has larger negative weights for out-of-time pile-up of up to 70 % of the (positive) pulse peak height, as compared to typically 10–20 % in the other LAr calorimeters [19]. These larger negative weights lead to larger signal suppression with increasing activity in the pile-up history and thus with increasing $\mu$.

## In situ transverse momentum balance techniques

In this section an overview is given on how the data-to-MC differences are assessed using in situ techniques exploiting the transverse momentum balance between the jet and a well-measured reference object.

The calibration of jets in the forward region of the detector relative to jets in the central regions is discussed in more detail in Sect. 8. Jets in the central region are calibrated using photons or $Z$ bosons as reference objects up to a transverse momentum of 800 GeV (see Sects. 9 and 10). Jets with higher $p_{\mathrm {T}}$ are calibrated using a system of low-$p_{\mathrm {T}}$ jets recoiling against a high-$p_{\mathrm {T}}$ jet (see Sect. 11).

### Relative in situ calibration between the central and forward rapidity regions

Transverse momentum balance in dijet events is exploited to study the pseudorapidity dependence of the jet response. A relative $\eta$-intercalibration is derived using the *matrix method* described in Ref. [3] to correct the jets in data for residual effects not captured by the initial calibration derived from MC simulations and based on truth jets. This method is applied for jets with $20 \le p_{\mathrm {T}}^\mathrm {jet}<1500$ GeV and $|\eta _\mathrm{det}| \le 4.5$. Jets up to $|\eta _\mathrm{det}| = 2.8$ are calibrated using $|\eta _\mathrm{det}| < 0.8$ as a reference region. For jets with $\eta _\mathrm{det}>2.8$ ($\eta _\mathrm{det}<-2.8$), for which the uncertainty on the derived calibration becomes large, the calibration determined at $\eta _\mathrm{det}=2.8$ ($\eta _\mathrm{det}=-2.8$) is used.^6^ Jets that fall in the reference region receive no additional correction on average. The $\eta$-intercalibration is applied to all jets prior to deriving the absolute calibration of the central region.

The largest uncertainty of the dijet balance technique is due to the modelling of the additional parton radiation altering the $p_{\mathrm {T}}$ balance. This uncertainty is estimated using MC simulations employing the Pythia and Herwig++ generators, respectively.

### In situ calibration methods for the central rapidity region

The energy scale of jets is tested in situ using a well-calibrated object as reference. The following techniques are used for the central rapidity region $\eta _\mathrm{det}< 1.2$:

1. **Direct transverse momentum balance between a photon or a** $\varvec{Z}$ **boson and a jet** Events with a photon or a $Z$ boson and a recoiling jet are used to directly compare the transverse momentum of the jet to that of the photon or the $Z$ boson (direct balance, DB). The data are compared to MC simulations in the jet pseudorapidity range $|\eta _\mathrm{det}| < 1.2$. The $\gamma \text {-jet}$ analysis covers a range in photon transverse momentum from 25 to 800 GeV, while the $Z$-jet analysis covers a range in $Z$ transverse momentum from 15 to 200 GeV. However, only the direct transverse momentum balance between the $Z$ and the jet is used in the derivation of the residual JES correction, as the method employing $p_{\mathrm {T}}$ balance between a photon and the full hadronic recoil, rather than the jet (see item 2 below), is used in place of the direct $\gamma \text {-jet}$ balance, see Sect. 13.5 for more details.
2. **Transverse momentum balance between a photon and the hadronic recoil** The photon transverse momentum is balanced against the full hadronic recoil using the projection of the missing transverse momentum onto the photon direction. With this missing transverse momentum projection fraction (MPF) technique, the calorimeter response for the hadronic recoil is measured, which is independent of any jet definition. The comparison is done in the same kinematic region as the direct photon balance method.
3. **Balance between a low-** $\varvec{p}_{{\mathbf {T}}}$ **jet system and a high-** $\varvec{p}_{{\mathbf {T}}}$ **jet** Jets at high $p_{\mathrm {T}}$ can be balanced against a recoil system of low $p_{\mathrm {T}}$ jets within $\eta _\mathrm{det}< 2.8$ if the low $p_{\mathrm {T}}$ jets are well calibrated using $\gamma \text {-jet}$ or $Z$-jet in situ techniques. The multijet balance can be iterated several times to increase the non-leading (in terms of $p_{\mathrm {T}}$) jets $p_{\mathrm {T}}$ range beyond the values covered by $\gamma \text {-jet}$ or $Z$-jet balance, and reaching higher $p_{\mathrm {T}}$ of the leading jet, until statistical limitations preclude a precise measurement. This method can probe the jet energy scale up to the  TeV regime.

In addition to the methods mentioned above, the mean transverse momentum sum of tracks within a cone around the jet direction provides an independent test of the calorimeter energy scale over the entire measured $p_{\mathrm {T}}^\mathrm {jet}$ range within the tracking acceptance. This method, described in Ref. [3], is used for the 2010 dataset and is also studied for the inclusive jet data sample in 2011. It is also used for *b*-jets (see Sect. 19). However, because of the relatively large associated systematic uncertainties, it is not included in the JES calibration derived from the combination of in situ methods for inclusive jets in 2011. This calibration can be constrained to much higher quality by applying the three methods described above.

## Relative forward-jet calibration using dijet events 

The calibration of the forward detector can be performed by exploiting the transverse momentum balance in events with two jets at high transverse momentum. A well calibrated jet in the central part of the detector is balanced against a jet in the forward region.

Thus the whole detector acceptance in $\eta$ can be equalised as a function of $p_{\mathrm {T}}^\mathrm {jet}$. In addition to this simple approach, a matrix method is used where jets in all regions (and not only the central one) are used for the $\eta$-intercalibration.

In the following the results for the EM+JES scheme are discussed as an example. While the measured relative response can deviate by a few percent between the EM+JES and the LCW+JES calibration schemes, the ratio between data and Monte Carlo simulation agrees within a few permille.

### Intercalibration using events with dijet topologies

#### Intercalibration using a central reference region

The standard approach for $\eta$-intercalibration with dijet events is to use the central region of the calorimeters as the reference region, as described in Ref. [79]. The relative calorimeter response of jets in other calorimeter regions is measured by the $p_{\mathrm {T}}$ balance between the reference jet (with $p_{\mathrm {T}}^{\mathrm {ref}}$) and the probe jet (with $p_{\mathrm {T}}^{\mathrm {probe}}$), exploiting the fact that these jets are expected to have equal $p_{\mathrm {T}}$ due to transverse momentum conservation. The $p_{\mathrm {T}}$ balance is expressed in terms of the asymmetry $\mathcal {A}$,

7 $$\begin{aligned} \mathcal {A}= \frac{p_{\mathrm {T}}^{\mathrm {probe}}- p_{\mathrm {T}}^{\mathrm {ref}}}{p_{\mathrm {T}}^\mathrm {avg}}, \end{aligned}$$

with $p_{\mathrm {T}}^\mathrm {avg}= (p_{\mathrm {T}}^{\mathrm {probe}}+ p_{\mathrm {T}}^{\mathrm {ref}})/2$. The reference region is chosen as the central region of the barrel calorimeter, given by $|\eta _\mathrm{det}|<0.8$. If both jets fall into the reference region, each jet is used, in turn, to probe the other. As a consequence, the average asymmetry in the reference region will be zero by construction.

The asymmetry is then used to measure an $\eta$-intercalibration factor $c$ of the probe jet, or its response relative to the reference jet $1/c$, using the relation

8 $$\begin{aligned} \frac{p_{\mathrm {T}}^{\mathrm {probe}}}{p_{\mathrm {T}}^{\mathrm {ref}}} =\frac{2+\mathcal {A}}{2-\mathcal {A}} =1/c. \end{aligned}$$

**Fig. 9:**
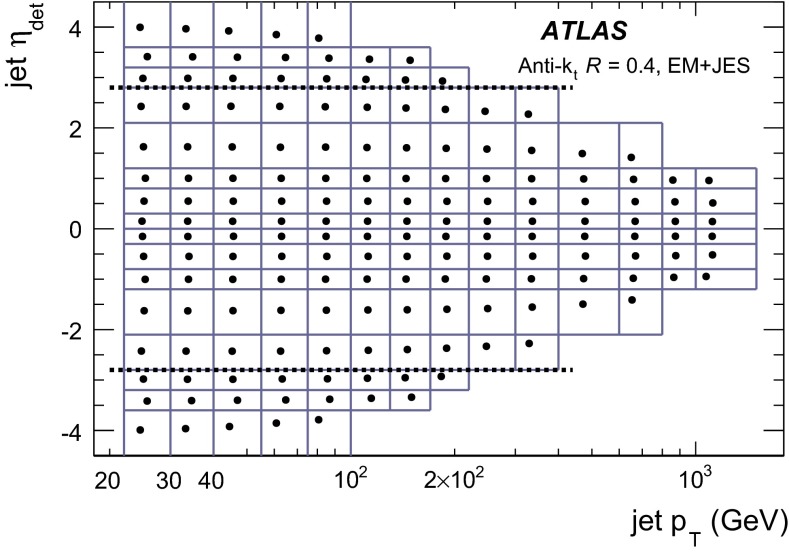
Overview of the $(p_{\mathrm {T}}^\mathrm {avg},\eta _\mathrm{det})$ bins of the dijet balance measurements for jets reconstructed with distance parameter $R=0.4$ calibrated using the EM+JES scheme. The *solid lines* indicate the $( p_{\mathrm {T}}^\mathrm {avg},\eta _{\mathrm {det}}^{\mathrm {probe}})$ bin edges, and the points show the average transverse momentum and pseudorapidity of the probe jet within each bin. The measurements within the $\eta _\mathrm{det}$ range spanned by the two *thick, dashed lines* are used to derive the residual calibration

The measurement of $c$ is performed in bins of jet $\eta _\mathrm{det}$ and $p_{\mathrm {T}}^\mathrm {avg}$, where $\eta _\mathrm{det}$ is defined as discussed in Sect. 5.6. Using the standard method outlined above, there is an asymmetry distribution $\mathcal {A}_{ik}$ for each probe jet $\eta _\mathrm{det}$ bin $i$ and each $p_{\mathrm {T}}^\mathrm {avg}$ bin $k$ An overview of the binning is given in Fig. 9 for jets with $R=0.4$ calibrated with the EM+JES scheme. The same bins are used for jets calibrated with the EM+JES or LCW+JES scheme. However, the binning is changed for jets with $R=0.6$ to take the different trigger thresholds into account. Intercalibration factors are calculated for each bin according to Eq. (8), resulting in

$$\begin{aligned} c_{ik}=\frac{2-\langle \mathcal {A}_{ik}\rangle }{2+\langle \mathcal {A}_{ik}\rangle }, \end{aligned}$$

where the $\langle \mathcal {A}_{ik}\rangle$ is the mean value of the asymmetry distribution in each bin. The uncertainty on $\langle \mathcal {A}_{ik}\rangle$ is taken to be the RMS/$\sqrt{N}$ of each distribution. For the data, $N$ is the number of events in the bin, while for the MC sample the number of effective events $N_{\mathrm {eff}}$ is used ($N = N_{\mathrm {eff}}$) to incorporate MC event weights $w_{k}$,

$$\begin{aligned} N_{\mathrm {eff}} = \left( \sum {w_k}\right) ^2/\sum {w_k^2}. \end{aligned}$$

Here the sums are running over all events of the MC sample. The above procedure is referred to as the *central reference method*.

#### Intercalibration using the matrix method

A disadvantage with the central reference method outlined above is that all events are required to have a jet in the central reference region. This results in a significant loss of event statistics, especially in the forward region, where the dijet cross section drops steeply as the rapidity interval between the jets increases. In order to use the full statistics, one can extend the central reference method by replacing the probe and reference jets by “left” and “right” jets, defined by $\eta _{\mathrm {det}}^{\mathrm {left}}<\eta _{\mathrm {det}}^{\mathrm {right}}$. Equations (7) and (8) then become

$$\begin{aligned} \mathcal {A}\!=\! \frac{p_{\mathrm {T}}^\mathrm{left}\! -\! p_{\mathrm {T}}^\mathrm{right}}{p_{\mathrm {T}}^\mathrm {avg}}, \qquad \mathrm {and} \qquad \mathcal {R}= \frac{p_{\mathrm {T}}^\mathrm{left}}{p_{\mathrm {T}}^\mathrm{right}}\!=\! \frac{c^\mathrm{right}}{c^\mathrm{left}}\!=\! \frac{2+\mathcal {A}}{2-\mathcal {A}}, \end{aligned}$$

where the term $\mathcal {R}$ denotes the ratio of the responses, and $c^\mathrm{left}$ and $c^\mathrm{right}$ are the $\eta$-intercalibration factors for the left and right jet, respectively.

This approach yields response ratio ($\mathcal {R}_{ijk}$) distributions with an average value $\langle R_{ijk}\rangle$, evaluated for each $\eta _{\mathrm {det}}^{\mathrm {left}}$ bin $i$, $\eta _{\mathrm {det}}^{\mathrm {right}}$ bin $j$, and $p_{\mathrm {T}}^\mathrm {avg}$ bin $k$. The relative correction factor $c_{ik}$ for a given jet in $\eta _\mathrm{det}$ bin $i$, with $i = 1 \ldots N$, and for a fixed $p_{\mathrm {T}}^\mathrm {avg}$ bin $k$ is then obtained by a minimisation procedure using a set of $N$ equations,

9 $$\begin{aligned} S(c_{1k},\ldots ,c_{Nk})&= \sum _{j=1}^{N} \sum _{i=1}^{j-1} \left( \frac{1}{\Delta \langle \mathcal {R}_{ijk} \rangle }\left( c_{ik} \langle \mathcal {R}_{ijk} \rangle -c_{jk}\right) \right) ^2\nonumber \\&\quad {} +X(c_{1k},\ldots ,c_{Nk}) . \end{aligned}$$

Here $\Delta \langle \mathcal {R}\rangle$ is the statistical uncertainty of $\langle \mathcal {R}\rangle$ and the function $X(c_{ik})$ is used to quadratically suppress deviations from unity of the average corrections,^7^

$$\begin{aligned} X(c_{1k},\ldots ,c_{Nk})=K\left( N^{-1} \sum _{i=1}^{N} c_{ik} - 1\right) ^{2}. \end{aligned}$$

The value of the constant $K$ does not influence the solution as long as it is sufficiently large, e.g. $K \approx N_{\mathrm {bins}}$, where $N_{\mathrm {bins}}$ is the number of $\eta _\mathrm{det}$ bins. The minimisation according to Eq. (9) is performed separately for each $p_{\mathrm {T}}$ bin $k$, and the resulting calibration factors $c_{i}$ obtained in each $\eta _\mathrm{det}$ bin $i$ are scaled such that the average calibration factor in the reference region $|\eta _\mathrm{det}|<0.8$ equals unity. This method is referred to as the *matrix method*.

### Event selection for dijet analysis

#### Trigger selection

Events are retained from the calorimeter trigger stream using a combination of central ($|\eta _\mathrm{det}|<3.2$) and forward ($|\eta _\mathrm{det}|>3.1$) jet triggers [18].

The selection is designed such that the trigger efficiency for a specific region of $p_{\mathrm {T}}^\mathrm {avg}{}$ is greater than 99 %, and approximately flat as a function of the pseudorapidity of the probe jet. Due to the different prescales for the central and forward jet triggers, the data collected by each trigger correspond to different integrated luminosities. To correctly normalise the data, events are assigned weights depending on the luminosity and the trigger decisions, according to the exclusion method described in Ref. [80].

**Table 1:** Summary of the event topology selection criteria applied in this analysis. The symbols “$\text {jet}1$” and “$\text {jet}2$” refer to the leading two jets (two highest-$p_{\mathrm {T}}$ jets), while “$\text {jet}3$” indicates the highest-$p_{\mathrm {T}}$ sub-leading (third) jet in the event

Variable	Selection
$\Delta \phi (\text {jet}1,\text {jet}2)$	$>$2.5 rad
$p_{\text {T}}^{\,\text {jet}3}$, $|\eta _\mathrm{det}(\text {jet}3)| < 2.5$	$<$ $\max (0.25\, p_{\mathrm {T}}^\mathrm {avg}{}, 12 ~\mathrm{GeV })$
$p_{\text {T}}^{\,\text {jet}3}$, $|\eta _\mathrm{det}(\text {jet}3)| > 2.5$	$<$ $\max (0.20\, p_{\mathrm {T}}^\mathrm {avg}{}, 10 ~\mathrm{GeV })$
$\mathrm {JVF}(\text {jet}3)$, $|\eta _\mathrm{det}(\text {jet}3)|<2.5$	$>$0.6

#### Dataset and jet quality selection

All ATLAS sub-detectors are required to be operational and events are rejected if any data-quality issues are present. The leading two jets are required to fulfil the default set of jet quality criteria (see Sect. 5.3). A dead calorimeter region was present for a subset of the data. To remove any bias from this region, events are removed if any jets are reconstructed close to this region.

**Fig. 10:**
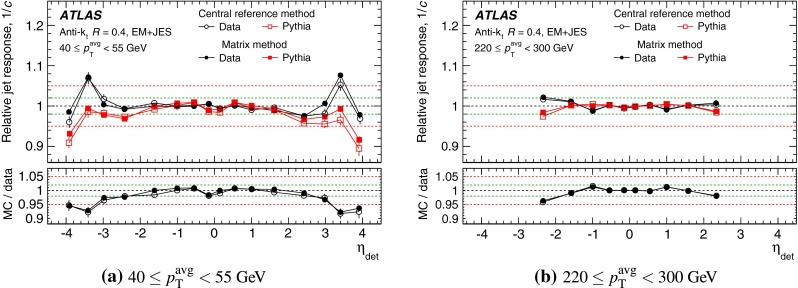
Relative jet response ($1/c$) for anti-$k_{t}$ jets with $R=0.4$ calibrated with the EM+JES scheme as a function of the probe jet pseudorapidity measured using the matrix and the central reference methods. Results are presented in **a** for $40 \le p_{\mathrm {T}}^\mathrm {avg}<55$ GeV and in **b** for $220 \le p_{\mathrm {T}}^\mathrm {avg}<300$ GeV. The *lower* parts of the figures show the ratios between relative response in data and MC

#### Dijet topology selection

In order to use the momentum balance of dijet events to measure the jet response, it is important that the events used have a relatively clean $2 \rightarrow 2$ topology. If a third jet is produced in the same hard-scatter proton–proton interaction, the balance between the leading (in $p_{\mathrm {T}}$) two jets is affected. To enhance the number of events in the sample that have this $2 \rightarrow 2$ topology, selection criteria on the azimuthal angle $\Delta \phi (\text {jet}1,\text {jet}2)$ between the two leading jets, and $p_{\mathrm {T}}$ requirements on additional jets are applied. Table 1 summarises these topology selection criteria.

In addition, all jets used for balancing and topology selection have to originate from the hard-scattering vertex, and not from a vertex reconstructed from a pile-up interaction. For this, each jet considered is evaluated with respect to its jet vertex fraction ($\mathrm {JVF}$), a likelihood measure estimating the vertex contribution to a jet [3]. To calculate $\mathrm {JVF}$, reconstructed tracks originating from reconstructed primary vertices $i = 1,\ldots ,N_{\mathrm{PV}}$ are matched to jets using an angular matching criterion in $(\eta ,\phi )$ space of $\Delta R < 0.4$ with respect to the jet axis. The track parameters calculated at the distance of closest approach to the selected hard-scattering vertex are used for this matching. For each jet, the scalar sum of the $p_{\mathrm {T}}$ of these matched tracks, $\Sigma _i$, is calculated for each vertex $i$ contributing to the jet. The $\mathrm {JVF}$ variable is then defined as the $p_{\mathrm {T}}$ sum for the hard-scattering vertex, $\Sigma _0$, divided by the sum of $\Sigma _i$ over all primary vertices. Any jet that has $|\eta _\mathrm{det}|<2.5$ and $\mathrm {JVF}>0.6$ is classified as “vertex confirmed” since it is likely to originate from the hard-scattering vertex.

This selection differs from that used in previous studies [3] due to the much higher instantaneous luminosities experienced during data taking and the consequentially increasing pile-up. In the forward region $|\eta _\mathrm{det}|>2.5$, no tracking is available, and events containing any additional forward jet with significant $p_{\mathrm {T}}$ are removed (see the third criteria in Table 1).

### Dijet balance results

#### Binning of the balance measurements

An overview of the ($p_{\mathrm {T}}^\mathrm {avg}$,$\eta _\mathrm{det}$) bins used in the analysis is presented in Fig. 9. All events falling in a given $p_{\mathrm {T}}^\mathrm {avg}$ bin are collected using a dedicated central and forward trigger combination. The statistics in each $p_{\mathrm {T}}^\mathrm {avg}$ bin are similar, except for the highest and lowest bins which contain fewer events. The loss of statistical precision of the measurements for the lower $p_{\mathrm {T}}^\mathrm {avg}$ bins is introduced by a larger sensitivity to the inefficiency of the pile-up suppression strategy, which rejects relatively more events due to the kinematic overlap of the hard-scatter jets with jets from pile-up. In addition, the asymmetry distribution broadens due to worsening relative jet $p_{\mathrm {T}}$ resolution, leading to larger fluctuations in this observable.

Each $p_{\mathrm {T}}^\mathrm {avg}$ bin is further divided into several $\eta _\mathrm{det}$ bins. The $\eta _\mathrm{det}$ binning is motivated by detector geometry and statistics.

**Fig. 11:**
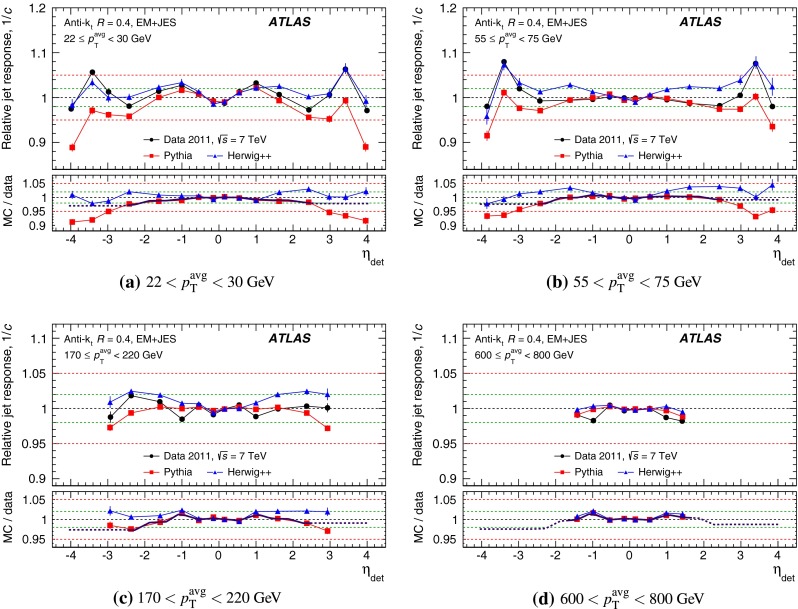
Relative jet response ($1/c$) as a function of the jet pseudorapidity for anti-$k_{t}$ jets with $R=0.4$ calibrated with the EM+JES scheme, separately for **a** $22 \le p_{\mathrm {T}}^\mathrm {avg}{}<30$ GeV, **b** $55 \le p_{\mathrm {T}}^\mathrm {avg}{}<75$ GeV, **c** $170 \le p_{\mathrm {T}}^\mathrm {avg}{}<220$ GeV and **d** $600 \le p_{\mathrm {T}}^\mathrm {avg}{}<800$ GeV. The *lower* parts of the figures show the ratios between the data and MC relative response. These measurements are performed using the matrix method. The applied correction is shown as a *thick line*. The line is *solid* over the range where the measurements is used to constrain the calibration, and *dashed* in the range where extrapolation is applied

**Fig. 12:**
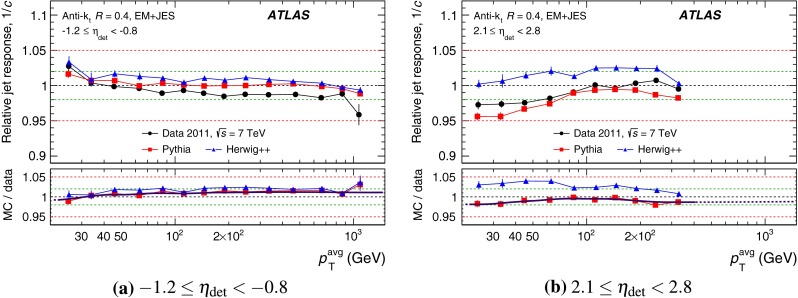
Relative jet response ($1/c$) as a function of the average jet $p_{\mathrm {T}}$ of the dijet system for anti-$k_{t}$ jets with $R=0.4$ calibrated with the EM+JES scheme, separately for **a** $-1.2\le \eta _\mathrm{det}<-0.8$ and **b** $2.1\le \eta _\mathrm{det}<2.8$. The *lower* parts of the figures show the ratios between the data and MC relative response. The applied correction is shown as a *thick line*

#### Comparison of intercalibration methods

The relative jet response obtained with the matrix method is compared to the relative jet response obtained using the central reference method. Figure 10a and b show the jet response relative to central jets ($1/c$) for two $p_{\mathrm {T}}^\mathrm {avg}$ bins, $40 \le p_{\mathrm {T}}^\mathrm {avg}{}<55$ GeV and $220\le p_{\mathrm {T}}^\mathrm {avg}{}<300$ GeV. In the most forward region at low $p_{\mathrm {T}}$, the matrix method tends to give a slightly higher relative response compared to the central reference method (see Fig. 10a). However, the same relative shift is observed both for data and MC simulations, and consequently the data-to-MC ratios are consistent. The matrix method is therefore used to measure the relative response as it has better statistical precision.

#### Comparison of data with Monte Carlo simulation

Figure 11 shows the relative response obtained using the matrix method as a function of the jet pseudorapidity for data and MC simulations. Four different $p_{\mathrm {T}}^\mathrm {avg}$ regions are shown, $22\le p_{\mathrm {T}}^\mathrm {avg}<30$ GeV, $55\le p_{\mathrm {T}}^\mathrm {avg}<75$ GeV, $170\le p_{\mathrm {T}}^\mathrm {avg}<220$ GeV, and $600\le p_{\mathrm {T}}^\mathrm {avg}<800$ GeV. Figure 12 shows the relative response as a function of $p_{\mathrm {T}}^\mathrm {avg}$ for two representative $\eta _\mathrm{det}$ bins, namely $-1.2\le \eta _\mathrm{det}<-0.8$ and $2.1\le \eta _\mathrm{det}<2.8$. The general features of the response in data are reasonably well reproduced by the MC simulations. However, as observed in previous studies [3], the Herwig++ MC generator predicts a higher relative response than Pythia for jets outside the central reference region ($|\eta _\mathrm{det}|>0.8$). Data tend to fall in-between the two predictions. This discrepancy was investigated and is observed both for truth jets built from stable particles (before any detector modelling), and also jets built from partons (before hadronisation). The differences therefore reflect a difference in physics modelling between the event generators, most likely due to the parton showering. The Pythia predictions are based upon a $p_{\mathrm {T}}$-ordered parton shower whereas the Herwig++ predictions are based on an angular-ordered parton shower.

For $p_{\mathrm {T}}{}>40$ GeV and $|\eta _\mathrm{det}|<2$, Pythia tends to agree better with data than Herwig++ does. In the more forward region, the spread between the Pythia and Herwig++ response predictions increases and reaches approximately 5 % at $|\eta _\mathrm{det}|=4$. In the more forward region ($|\eta _\mathrm{det}|>3$) the relative response prediction of Herwig++ generally agrees better with data than Pythia.

#### Derivation of the residual correction

The residual calibration is derived from the data/Pythia ratio $C_i = c^\mathrm{data}_i/c^{\textsc {Pythia}}_i$ of the measured $\eta$-intercalibration factors. Pythia is used as the reference as it is also used to obtain the initial (main) calibration, see Sect. 5. The correction is a function of jet $p_{\mathrm {T}}$ and $\eta _\mathrm{det}$ ($F_\mathrm{rel}(p_{\mathrm {T}},\eta _\mathrm{det})$) and is constructed by combining the $N_\mathrm{bins}$ measurements of the $(p_{\mathrm {T}}^\mathrm {avg},\eta _\mathrm{det})$ bins using a two-dimensional Gaussian kernel, like

$$\begin{aligned} F_\mathrm{rel}(p_{\mathrm {T}},\eta _\mathrm{det}) = \dfrac{\sum _{i=1}^{N_\mathrm{bins}} C_i w_i}{\sum _{i=1}^{N_\mathrm{bins}} w_i}, \end{aligned}$$

with

$$\begin{aligned} w_i \!=\! \dfrac{1}{\Delta C_i^2} \!\times \! \mathrm{Gaus}\left( \dfrac{\log p_{\mathrm {T}}\!-\! \log \langle p_{\mathrm {T}}^{\mathrm {probe}}\rangle _i}{\sigma _{\log p_{\mathrm {T}}}} \oplus \dfrac{\eta _\mathrm{det}\!-\! \langle \eta _\mathrm{det}\rangle _i}{\sigma _\eta }\!\right) \!. \end{aligned}$$

Here $i$ denotes the index of a $(p_{\mathrm {T}}^\mathrm {avg},\eta _\mathrm{det})$-bin, $\Delta C_i$ is the statistical uncertainty of $C_i$,$\langle p_{\mathrm {T}}^{\mathrm {probe}}\rangle _i$ and $\langle \eta _\mathrm{det}\rangle _i$ are the average $p_{\mathrm {T}}$ and $\eta _\mathrm{det}$ of the probe jets in the bin (see Fig. 9). The Gaussian function has a central value of zero and a width controlled by $\sigma _{\log p_{\mathrm {T}}}$ and $\sigma _\eta$.

Only the measurements with $|\eta _\mathrm{det}|<2.8$ are included in the derivation of the correction function because of the large discrepancy between the modelled response of the MC simulation samples in the more forward region. This $\eta _\mathrm{det}$ boundary is indicated by a thick, dashed line in Fig. 9. The residual correction is held fixed for pseudorapidities larger than those of the most forward measurements included ($|\eta _\mathrm{det}|\approx 2.4$). All jets with a given $p_{\mathrm {T}}$ and $|\eta _\mathrm{det}|>2.4$ will hence receive the same $\eta$-intercalibration correction. The kernel-width parameters used^8^ are found to capture the shape of the data-to-MC ratio, but at the same time provide stability against statistical fluctuations. This choice introduces a stronger constraint across $p_{\mathrm {T}}$. The resulting residual correction is shown as a thick line in the lower sections of Figs. 11 and 12. The line is solid over the range where the measurements is used to constrain the calibration, and dashed in the range where extrapolation is applied.

### Systematic uncertainty

The observed difference in the relative response between data and MC simulations could be due to mis-modelling of physics or detector effects used in the simulation. Suppression and selection criteria used in the analysis (e.g. topology selection and radiation suppression) can also affect the response through their influence on the mean asymmetry. The systematic uncertainty is evaluated by considering the following effects:

1. Response modelling uncertainty.
2. Additional soft radiation.
3. Response dependence on the $\Delta \phi$ selection between the two leading jets.
4. Uncertainty due to trigger inefficiencies.
5. Influence of pile-up on the relative response.
6. Influence of the jet energy resolution (JER) on the response measurements.

All systematic uncertainties are derived as a function of $p_{\mathrm {T}}$ and $|\eta _\mathrm{det}|$. No statistically significant difference is observed for positive and negative $\eta _\mathrm{det}$ for any of the uncertainties.

**Fig. 13:**
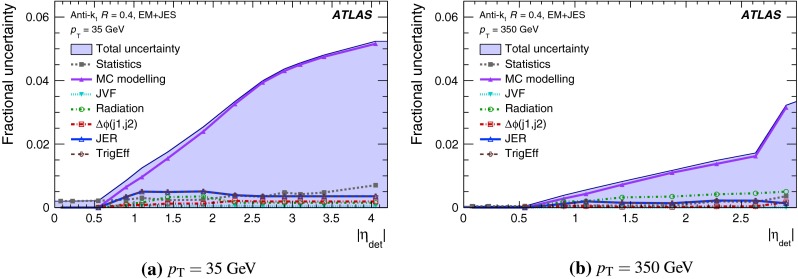
Summary of uncertainties on the intercalibration as a function of the jet $\eta _\mathrm{det}$ for anti-$k_{t}$ jets with $R=0.4$ calibrated with the EM+JES scheme, separately for **a** $p_{\mathrm {T}}{} = 35$ GeV and **b** $p_{\mathrm {T}}= 350$ GeV. The individual components are added in quadrature to obtain the total uncertainty. The MC modelling uncertainty is the dominant component

#### Modelling uncertainty

The two generators used for the MC simulation deviate in their predictions of the response for forward jets as discussed in Sect. 8.3.3. Since there is no a priori reason to trust one generator over the other, the full difference between the two predictions is used as the modelling uncertainty. This uncertainty is the largest component of the intercalibration uncertainty. In the reference region ($|\eta _\mathrm{det}|<0.8$), no uncertainty is assigned. For $0.8\le |\eta _\mathrm{det}|<2.4$, where data are corrected to the Pythia MC predictions, the full difference between Pythia and Herwig is taken as the uncertainty. For $|\eta _\mathrm{det}|>2.4$, where the calibration is extrapolated, the uncertainty is taken as the difference between the calibrated data and either Pythia or Herwig, whichever is larger.

#### Sub-leading jet radiation suppression

Additional radiation from sub-leading jets can affect the dijet balance. In order to mitigate these effects, selection criteria are imposed on the $p_{\mathrm {T}}$ of any additional jets in an event as discussed in Sect. 8.2. To assess the uncertainties due to the radiation suppression, the selection criteria are varied for both data and MC simulations, and the calibration is re-evaluated. The uncertainty is taken as the fractional difference between the varied and nominal calibrations. Each of the three selection criteria are varied independently. The $\mathrm {JVF}$ requirement is changed by $\pm 0.2$ from its nominal value (0.6) for central jets, and the fractional amount of $p_{\mathrm {T}}$ carried by the third jet relative to $p_{\mathrm {T}}^\mathrm {avg}$ is varied by $\pm$10 %. Finally, the minimum $p_{\mathrm {T}}$ cutoff is changed by $\pm 2$ $\mathrm {GeV}$.

#### $\Delta \phi (\text {jet}1,\text {jet}2)$ event selection

The event topology selection requires that the two leading jets have a $\Delta \phi$ separation greater than 2.5 rad. In order to assess the influence of this selection on the $p_{\mathrm {T}}$ balance, the residual calibration is re-derived twice after shifting the selection criterion by $\pm 0.4$ rad ($\Delta \phi (\text {jet}1,\text {jet}2) < (2.5 \pm 0.4)$ rad), separately in either direction. The largest difference between the shifted and nominal calibrations is taken as the uncertainty.

#### Trigger efficiencies

Trigger biases can be introduced if the trigger selection, which is applied only to data, is not fully efficient. To assess the uncertainty associated with the small inefficiency in the trigger, the measured efficiencies are applied to the MC samples. The effect on the MC response is found to be negligible in comparison to the other sources, even when exaggerating the effect by shifting the measured efficiency curves to reach the plateau 10 % earlier in $p_{\mathrm {T}}$. This uncertainty is hence neglected.

#### Impact of pile-up interactions

The influence of pile-up on the relative response is evaluated. To assess the magnitude of the effect, the differences between low and high pile-up subsets are investigated. Two different selections are used, high and low $\mu$ subsets ($\mu < 7$ and $\mu \ge 7$), and high and low $N_{\mathrm{PV}}$ subsets ($N_{\mathrm{PV}}<5$ and $N_{\mathrm{PV}}\ge 5$). The discrepancies observed are well within the systematic uncertainty for the pile-up correction itself (see Sect. 16). Therefore, no further contribution from pile-up is included in the evaluation of the full systematic uncertainty of the $\eta$-intercalibration.

#### Jet resolution uncertainty

The jet energy resolution (JER) [81] in the MC simulation is comparable to the resolution observed in data. To assess the impact of the JER on the $p_{\mathrm {T}}$ balance, a smearing factor is applied as a scale factor to the MC jets, which results in an increased jet resolution consistent with the JER measured in data plus its error. It is randomly sampled from a Gaussian with width

10 $$\begin{aligned} \sigma = \sqrt{(\sigma _{\mathrm {data}}+\Delta \sigma _{\mathrm {data}})^{2} - \sigma _{\mathrm {data}}^{2}} , \end{aligned}$$

where $\sigma _{\mathrm {data}}$ is the measured jet resolution in data and $\Delta \sigma _{\mathrm {data}}$ is the corresponding uncertainty. The difference between the nominal and smeared MC results is taken as the JER systematic uncertainty.

### Summary of the $\eta$-intercalibration and its uncertainties

The pseudorapidity dependence of the jet response is analysed using dijet pseudorapidity $\eta$-intercalibration techniques. A residual $p_{\mathrm {T}}$ and $\eta _\mathrm{det}$ dependent response correction is derived with a matrix method for jets with $|\eta _\mathrm{det}|<2.4$. The correction is applied to data to correct for effects not captured by the default MC-derived calibration. The correction to the jet response is measured to be approximately +1 % at $|\eta _\mathrm{det}| = 1.0$ and falling to $-$3 % and to $-$1 % for $|\eta _\mathrm{det}|=2.4$ and beyond. The total systematic uncertainty is obtained as the quadratic sum of the various components mentioned. Figure 13 presents a summary of the uncertainties as a function of $\eta _\mathrm{det}$ for two representative values of jet transverse momentum, namely $p_{\mathrm {T}}= 35$ GeV and $p_{\mathrm {T}}= 350$ GeV. The uncertainty is parameterised in the same way as the correction as described in Sect. 8.3.4. There is no strong variation of the uncertainties as a function of jet $p_{\mathrm {T}}$. For a $p_{\mathrm {T}}= 25$ GeV jet, the uncertainty is about 1 % at $|\eta _\mathrm{det}|=1.0$, 3 % at $|\eta _\mathrm{det}|=2.0$ and about 5 % for $|\eta _\mathrm{det}|>3.0$. The uncertainty is below 1 % for $p_{\mathrm {T}}= 500$ GeV jets with $|\eta _\mathrm{det}|<2$.

## Jet energy calibration using *Z*-jet events

This section presents results based on events where a $Z$ boson decaying to an $e^+ e^-$ pair is produced together with a jet, which balance each other in the transverse plane. The $p_{\mathrm {T}}$ balance is compared in data and in MC simulations, and a study of systematic uncertainties on the data-to-MC ratio is carried out. The results from a similar study with $\gamma \text {-jet}$ events are discussed in Sect. 10.

The advantage of $Z$-jet events is the possibility of probing low-$p_{\mathrm {T}}$ jets, which are difficult to reach with $\gamma \text {-jet}$ events due to trigger thresholds and background contamination in that region. On the other hand, $\gamma \text {-jet}$ events benefit from larger statistics for $p_{\mathrm {T}}$ above $150 ~\mathrm{GeV }$. In the $Z$-jet and $\gamma \text {-jet}$ analyses, jets with a pseudorapidity $|\eta _\mathrm{det}|<1.2$ are probed.

### Description of the $p_\mathrm{T }$ balance method

In events where one $Z$ boson and only one jet are produced, the jet recoils against the $Z$ boson, ensuring approximate momentum balance between them in the transverse plane. The direct $p_{\mathrm {T}}$ balance technique exploits this relationship in order to improve the jet energy calibration.

If the $Z$ boson decays into electrons, its four-momentum is reconstructed using the electrons, which are accurately measured in the electromagnetic calorimeter and the inner detector [72]. Ideally, if the jet includes all the particles that recoil against the $Z$ boson, and if the electron energies are perfectly measured, the response of the jet in the calorimeters can be determined by using $p_{\mathrm {T}}^{Z}$ as the reference truth-jet $p_{\mathrm {T}}$. However, this measurement is affected by the following:

1. Uncertainty on the electron energy measurements.
2. Particles contributing to the $p_{\mathrm {T}}$ balance that are not included in the jet cone (out-of-cone radiation).
3. Additional parton radiation contributing to the recoil against the $Z$ boson.
4. Contribution from the underlying event.
5. In-time and out-of-time pile-up.

Therefore, the direct $p_{\mathrm {T}}$ balance between a $Z$ boson and a jet ($p_{\mathrm {T}}^{\text {jet}}/p_{\mathrm {T}}^{\text {ref}}$) is not used to estimate the jet response, but only to assess how well the MC simulation can reproduce the data.

To at least partly reduce the effect of additional parton radiation perpendicular to the jet axis in the transverse plane, a reference $p_{\mathrm {T}}^{\text {ref}}=p_{\mathrm {T}}^{Z}\times |\cos (\Delta \phi (\mathrm {jet},Z))|$ is constructed from the azimuthal angle $\Delta \phi (\mathrm {jet},Z)$ between the $Z$ boson and the jet, and the $Z$ boson transverse momentum $p_{\mathrm {T}}^{Z}$.

The jet calibration in the data is then adjusted using the data-to-MC comparison of the $p_{\mathrm {T}}^{\text {jet}}/p_{\mathrm {T}}^{\text {ref}}$ ratio for the two jet calibration schemes EM+JES and LCW+JES described in Sect. 5. The effects altering this ratio are evaluated by changing kinematic and topological selections and MC event generators and other modelling parameters. In particular the extrapolation of the $\Delta \phi (\text {jet},Z)$ dependence of $p_{\mathrm {T}}^{\text {jet}}/p_{\mathrm {T}}^{\text {ref}}$ to the least radiation-biased regime ($\Delta \phi (\text {jet},Z) = \pi$) is sensitive to the MC-modelling quality and is investigated with data-to-MC comparisons.

### Selection of *Z*-jet events

Events are selected online using a trigger logic that requires the presence of at least one well-identified electron with transverse energy ($E^e_{\text {T}}$) above $20~\mathrm{GeV }$ (or $22~\mathrm{GeV }$, depending on the data-taking period) or two well-identified electrons with $E^e_{\text {T}} > 12~\mathrm{GeV }$, in the region $|\eta |<2.5$ [82]. Events are also required to have a primary hard-scattering vertex, as defined in Sect. 5.4, with at least three tracks associated to it. This renders the contribution from fake vertices due to beam backgrounds negligible.

Details of electron reconstruction and identification can be found in Ref. [72]. Three levels of electron identification quality are defined, based on different requirements on shower shapes, track quality, and track–cluster matching. The intermediate one (“medium”) is used in this analysis.

**Fig. 14:**
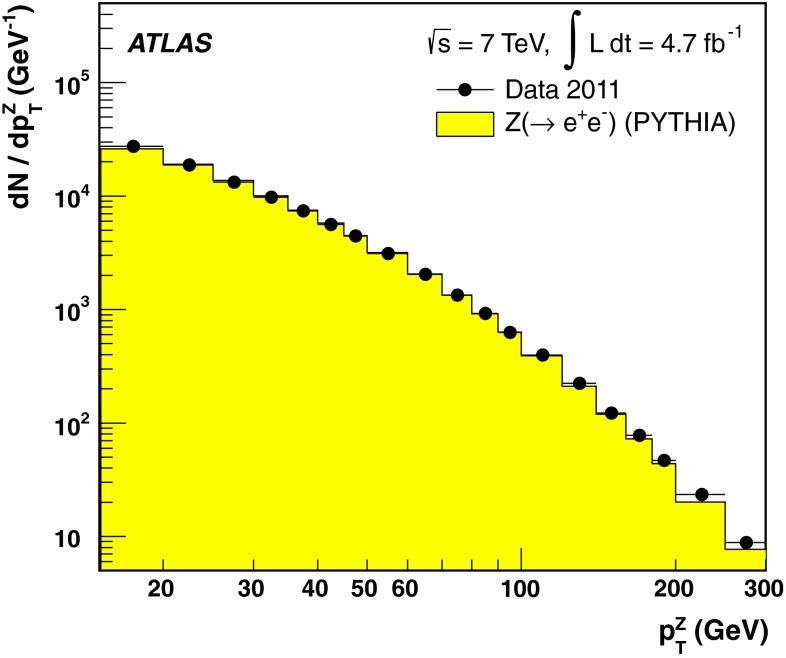
The $Z$ boson $p_{\mathrm {T}}$ distribution in selected $Z$ events. Data and prediction from the $Z \rightarrow ee$ Pythia simulation, normalised to the observed number of events, are compared

Events are required to contain exactly two such electron candidates with $E^e_{\text {T}}>20~\mathrm{GeV }$ and pseudorapidity in the range $|\eta ^e|<2.47$, where the transition region between calorimeter sections $1.37<|\eta ^e|<1.52$ is excluded, as well as small regions where an accurate energy measurement is not possible due to temporary hardware failures. If these electrons have opposite-sign charge, and yield a combined invariant mass in the range $66 < M_{e^+ e^-} < 116~\mathrm{GeV }$, the event is kept and the four-momentum of the $Z$ boson candidate is reconstructed from the four-momenta of the two electrons. The transverse momentum distribution of these $Z$ boson candidates is shown in Fig. 14.

All jets within the full calorimeter acceptance and with a JES-corrected transverse momentum $p_{\mathrm {T}}^{\text {jet}}>12~\mathrm{GeV }$ are considered. For each jet the $\mathrm {JVF}$ (see Sect. 8.2.3) is used to estimate the degree of pile-up contamination of a jet based on the vertex information. The highest-$p_{\mathrm {T}}$ (leading) jet must pass the quality criteria described in Sect. 5.3, have a $\mathrm {JVF}>0.5$, and be in the fiducial region $|\eta |<1.2$.

Furthermore, the leading jet is required to be isolated from the two electrons stemming from the $Z$ boson. The distance $\Delta R$ between the jet and each of the two electrons in ($\eta ,\phi$) space, measured according to Eq. (3) in Sect. 5.6, is required to be $\Delta R> 0.35\,(0.5)$ for anti-$k_{t}$ jets with $R=0.4\,(0.6)$.

The presence of additional high-$p_{\mathrm {T}}$ parton radiation altering the balance between the $Z$ boson and the leading jet is suppressed by requiring that the next-highest-$p_{\mathrm {T}}$ (sub-leading) jet has a calibrated $p_{\mathrm {T}}$ less than 20 % of the $p_{\mathrm {T}}$ of the $Z$ boson, with a minimal $p_{\mathrm {T}}$ of $12~\mathrm{GeV }$. For sub-leading jets within the tracking acceptance, this cut is only applied if the jet has a $\mathrm {JVF}>0.75$. A summary of the event selection is presented in Table 2.

**Table 2:** Summary of the event selection criteria applied in the $Z$-jet analysis

Variable	Selection
$E^e_{\text {T}}$	$>$ $20~\mathrm{GeV }$
$|\eta ^e|$	$<$2.47 (excluding calorimeter transition regions)
$p_{\mathrm {T}}^{\text {jet}}$	$>$ $12~\mathrm{GeV }$
$|\eta ^{\text {jet}}|$	$<$1.2
$M_{e^+ e^-}$	$66 <M_{e^+ e^-}<116~\mathrm{GeV }$
$\Delta R(\text {jet,electrons})$	$>$0.35 (0.5), anti-$k_{t}$ jets with $R=0.4\,(0.6)$
$p_{\mathrm {T}}^{\text {jet2}}/p_{\mathrm {T}}^{Z}$	$<$0.2

**Fig. 15:**
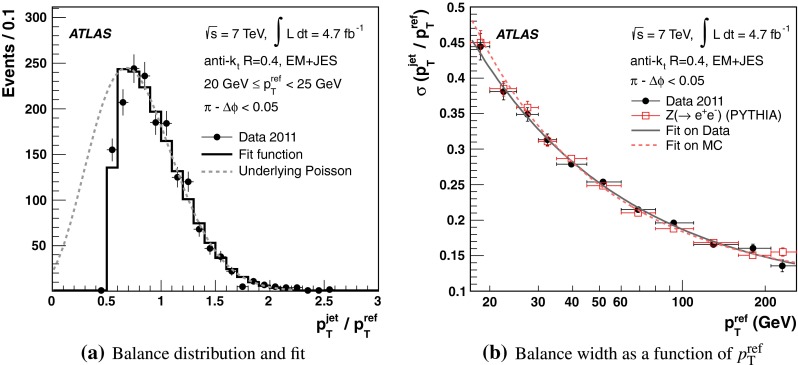
The $p_{\mathrm {T}}^{\text {jet}}/p_{\mathrm {T}}^{\text {ref}}$ distribution in the data for $20 \le p_{\mathrm {T}}^{\text {ref}} < 25~\mathrm{GeV }$ and $\pi -\Delta \phi (\mathrm {jet},Z)<0.05$ is shown in **a**. The *black solid* histogram represents the fit function, a Poisson distribution extended to non-integer values, multiplied by a turn-on curve. The value used in each bin is the mean value of that function in the bin. The *gray dashed line* shows the underlying Poisson distribution, from which the mean is taken as the measurement of the $p_{\mathrm {T}}$ balance. In **b** the measured widths of the $p_{\mathrm {T}}^{\text {jet}}/p_{\mathrm {T}}^{\text {ref}}$ distributions as a function of $p_{\mathrm {T}}^{\text {ref}}$ is shown for data and MC simulations, for events with $\pi -\Delta \phi (\mathrm {jet},Z)<0.05$. The fitted functional form defined by Eq. (11) is superimposed. In both figures, anti-$k_{t}$ jets with distance parameter $R=0.4$ calibrated with the EM+JES scheme are used. Only statistical uncertainties are shown

### Measurement of the $p_\mathrm{T }$ balance

The mean value of the $p_{\mathrm {T}}^\mathrm {jet}/p_{\mathrm {T}}^{\text {ref}}$ ratio distribution is computed in bins of $p_{\mathrm {T}}^{\text {ref}}$ and $\Delta \phi (\mathrm {jet},Z)$. This mean value is obtained with two methods, depending on $p_{\mathrm {T}}^{\text {ref}}$.

1. In the low-$p_{\mathrm {T}}^{\text {ref}}$ region ($17 \le p_{\mathrm {T}}^{\text {ref}}<35~\mathrm{GeV }$), it is obtained by a maximum likelihood fit applied to the distribution of the $p_{\mathrm {T}}^\mathrm {jet}/p_{\mathrm {T}}^{\text {ref}}$ ratio. The function used, hereafter denoted as the “fit function”, is a Poisson distribution extended to non-integer values^9^ and multiplied by a turn-on curve to model the effect of the $p_{\mathrm {T}}^\mathrm {jet}$ threshold, as depicted in Fig. 15a. For a given $[p_{\mathrm {T}}^{\text {ref,min}}, p_{\mathrm {T}}^{\text {ref,max}}]$ bin, the turn-on curve is equal to 1 above $12~\mathrm{GeV }/p_{\mathrm {T}}^{\text {ref,min}}$ and equal to 0 below $12~\mathrm{GeV }/p_{\mathrm {T}}^{\text {ref,max}}$. A linear function is used to interpolate the turn-on between these two values. The mean value of the underlying Poisson distribution is taken as the mean $p_{\mathrm {T}}$ balance. A fit is preferred to an arithmetic mean calculation because of the jet $p_{\mathrm {T}}$ cut, which biases the mean value of the balance distribution at low $p_{\mathrm {T}}^{\text {ref}}$ due to the jet energy resolution [83].
2. For larger $p_{\mathrm {T}}^{\text {ref}}$ ($p_{\mathrm {T}}^{\text {ref}}\ge 35~\mathrm{GeV }$), the arithmetic mean calculation is not sensitive to the jet threshold, and it gives results equivalent to those obtained with a fit. In this $p_{\mathrm {T}}^{\text {ref}}$ region, an arithmetic mean is therefore used as it leads to smaller uncertainties.

In the region where the fit is used, $17 \le p_{\mathrm {T}}^{\text {ref}}<35~\mathrm{GeV }$, the fit is actually performed twice, in order to reduce the impact of statistical fluctuations:

1. In each bin of $p_{\mathrm {T}}^{\text {ref}}$ and $\Delta \phi$, the mean and the width of the Poisson distribution are fitted simultaneously.
2. The distribution of the widths is parameterised as a function of $p_{\mathrm {T}}^{\text {ref}}$ in each $\Delta \phi$ bin according to:
  11 $$\begin{aligned} w(p_{\mathrm {T}}^{\text {ref}}) = \frac{a}{p_{\mathrm {T}}^{\text {ref}}} \oplus \frac{b}{\sqrt{p_{\mathrm {T}}^{\text {ref}}}} \oplus c . \end{aligned}$$
  The parameters $a$, $b$, and $c$ are obtained from a fit to the widths of the fitted Poisson distributions for $p_{\mathrm {T}}<35~\mathrm{GeV }$ and to the arithmetic RMS for larger $p_{\mathrm {T}}$ (see Fig. 15b). It is emphasised that this measured width can not directly be compared to the resolution determined in Ref. [83], since no extrapolation to a topology without radiation is performed here.
3. The fits to the $p_{\mathrm {T}}^\mathrm {jet}/p_{\mathrm {T}}^{\text {ref}}$ distributions are repeated, but now with the widths fixed to the values resulting from the parameterisations.

**Fig. 16:**
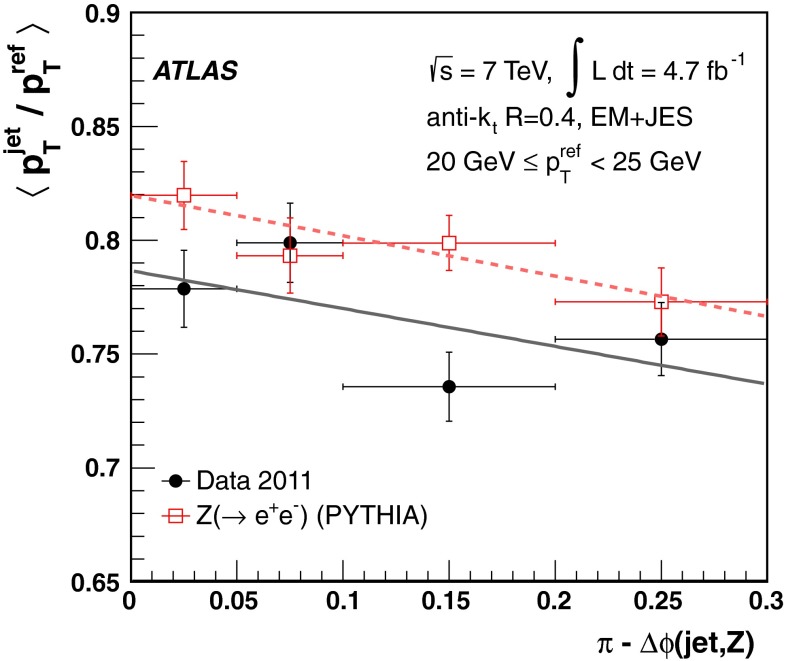
Mean $p_{\mathrm {T}}^\mathrm {jet}/p_{\mathrm {T}}^{\text {ref}}$ balance vs $\Delta \phi (\mathrm {jet},Z)$ for $20 \le p_{\mathrm {T}}^{\text {ref}} < 25~\mathrm{GeV }$ in the data and in the simulation. A linear function used to extrapolate the balance to $\Delta \phi = \pi$ is superimposed. Anti-$k_{t}$ jets with distance parameter $R=0.4$ calibrated with the EM+JES scheme are used. Only statistical uncertainties are shown

**Fig. 17:**
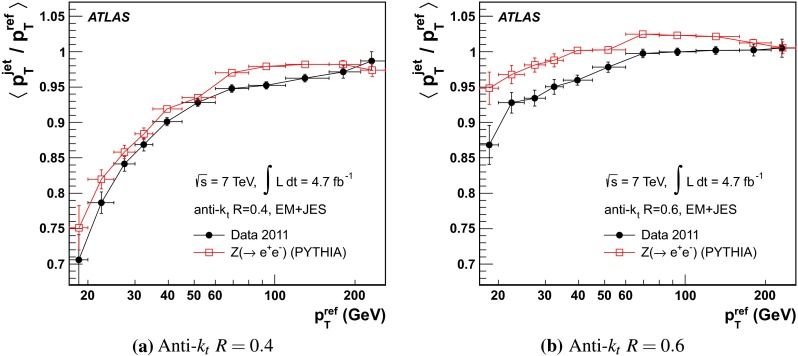
Mean $p_{\mathrm {T}}$ balance obtained in the data and with the Pythia simulation. Results for anti-$k_{t}$ jets with distance parameter **a** $R=0.4$ and **b** $R=0.6$ calibrated with the EM+JES scheme are shown. Only statistical uncertainties are shown

In order to estimate the mean balance for a topology where the jet and the $Z$ boson are back-to-back, the mean balances in $\Delta \phi$ bins are extrapolated to $\Delta \phi = \pi$ for each $p_{\mathrm {T}}^{\text {ref}}$ bin, using a linear function (see Fig. 16). This extrapolation reduces the sensitivity of the mean balance to additional parton radiation transverse to the leading jet axis, as discussed earlier in Sect. 9.1. The extrapolated mean balances for the data and MC-simulated samples generated by Pythia are shown in Fig. 17 for anti-$k_{t}$ jets with distance parameters of $R=0.4$ and $R=0.6$, calibrated with the EM+JES scheme. The mean balance obtained for jets with $R=0.6$ is larger compared to jets with $R=0.4$, which is a direct consequence of the larger jet size, and has smaller variations with the transverse momentum.

### Measuring out-of-cone radiation and underlying event contributions

The transverse momentum of the $Z$ boson is only approximately equal to the transverse momentum of the truth jet, because of out-of-cone radiation and contributions from the underlying event:

1. The $Z$ boson balances against all particles inside and outside the jet cone, whereas the truth jet clusters particles inside the jet cone only.
2. The truth jet’s $p_{\mathrm {T}}$ includes any UE particles that are clustered in the jet, whereas the $Z$ boson’s $p_{\mathrm {T}}$ receives almost no such contribution.

**Fig. 18:**
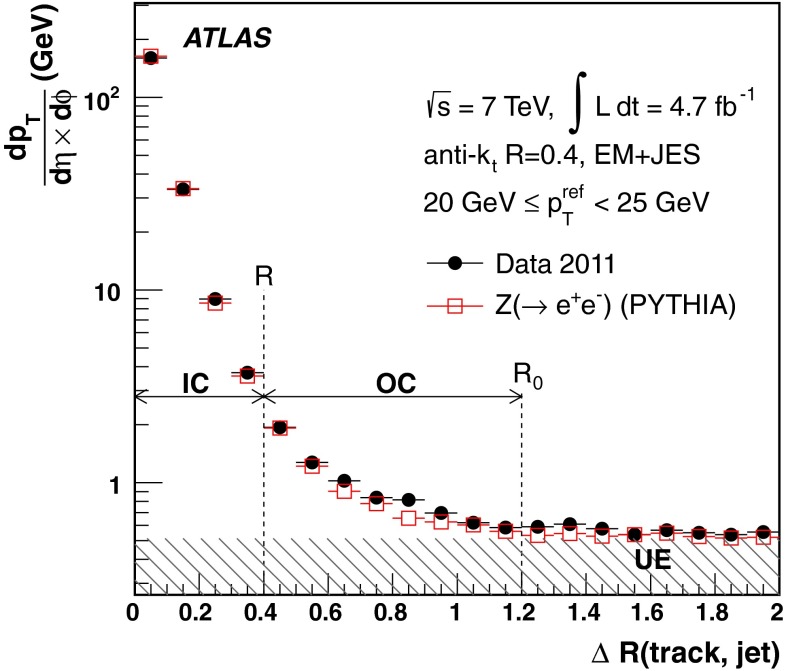
Transverse momentum profile of tracks around the leading jet axis for events with $20 \le p_\mathrm{T}^\mathrm{ref} < 25~\mathrm{GeV }$ in $Z$-jet events. The radii $R$ and $R_0$ are those used in Eq. (12) to define the IC and IC+OC regions. The *hatched area* indicates the contribution from the underlying event (UE). Anti-$k_{t}$ jets with $R=0.4$ calibrated with the EM+JES scheme are considered

These two contributions are estimated by measuring the transverse momentum profile of tracks around the leading jet axis (see Fig. 18). Tracks associated to the hard-scattering vertex are used instead of clusters of calorimeter cells in order to reduce the sensitivity to pile-up interactions. Tracks associated to the two electrons stemming from the $Z$ boson are removed when computing the transverse momentum profiles.

A factor is calculated from the out-of-cone and underlying event contributions:

12 $$\begin{aligned} k_\mathrm {OOC}{} = \frac{p_{\mathrm {T}}^\mathrm{IC, ALL}}{p_{\mathrm {T}}^\mathrm{IC+OC, ALL} - p_{\mathrm {T}}^\mathrm{IC+OC, UE}}\, , \end{aligned}$$

where $p_{\mathrm {T}}^\mathrm{IC, ALL}$ is the average scalar $p_{\mathrm {T}}$ sum of all the tracks inside the jet cone with radius $R$, $p_{\mathrm {T}}^\mathrm{IC+OC, ALL}$ is the average scalar $p_{\mathrm {T}}$ sum of all the tracks inside and outside the jet cone, and $p_{\mathrm {T}}^\mathrm{IC+OC, UE}$ is the average contribution of the underlying event to $p_{\mathrm {T}}^{\text {IC+OC, ALL}}$. The transverse momenta $p_{\mathrm {T}}^\mathrm{IC+OC, ALL}$ and $p_{\mathrm {T}}^\mathrm{IC+OC, UE}$ are estimated in a cone of radius $R_0$, above which only the UE contributes to $p_{\mathrm {T}}^\mathrm{IC+OC, ALL}$, and from where the transverse momentum density is constant (see Fig. 18). In practice, $R_0$ is the value where the logarithmic derivative of $k_\mathrm {OOC}$ with respect to $R_0$ is equal to 0.05.

### Systematic uncertainties

The differences between the balances observed in data and those observed in MC simulations may be due to physics or detector effects directly influencing the calorimeter response to jets (e.g. fragmentation or material in front of the calorimeter), which may not be correctly modelled in the simulation. They can also be due to effects that have an influence on the direct $p_{\mathrm {T}}$ balance method itself, e.g. the estimation of the mean balance or higher-order parton emissions. For a more detailed evaluation of the systematic uncertainties, the following steps are performed:

1. The uncertainty on the width parameterisation is propagated to the mean estimation.
2. The fit range used for the $\Delta \phi$ extrapolation is varied.
3. The cut on sub-leading jets is varied to assess the effect of additional high-$p_{\mathrm {T}}$ parton radiation altering the balance.
4. The effect of soft particles produced outside the jet cone and the underlying event contribution to the jet energy is compared in data and simulation.
5. The impact of additional (pile-up) interactions is studied.
6. The uncertainty in the electron energy measurement is propagated to the $p_{\mathrm {T}}$ balance.
7. The results obtained with Pythia and Alpgen+Herwig are compared.

#### Fitting procedure

For $p_{\mathrm {T}}^{\text {ref}}<35~\mathrm{GeV }$, the mean balance in a given bin of $p_{\mathrm {T}}^{\text {ref}}$ and $\Delta \phi$ is first obtained using the nominal parameterised width given in Eq. (11). The fit is then performed again with a larger and a smaller width according to the uncertainty on the parameterisation. The four differences obtained in the resulting mean balances for the up and down variations in data and Monte Carlo simulation are propagated independently, after $\Delta \phi$ extrapolation, to the data-to-MC ratio. The two positive and two negative deviations are both summed in quadrature and the final uncertainty is taken as the average of the absolute values of the two deviations.

#### Extrapolation procedure

The nominal extrapolated balance is determined with a linear fit from $\Delta \phi = \pi -0.3$ to $\Delta \phi = \pi$. The lower limit is decreased to $\pi -0.4$ and increased to $\pi -0.2$, and the average of the absolute values of the two deviations is taken as a systematic uncertainty on the data-to-MC ratio.

#### Additional radiation suppression

While the extrapolation of the $p_{\mathrm {T}}$ balance in $\Delta \phi$ attempts to reduce the effect of radiation perpendicular to the jet axis at angular scales within the range from $[\pi -0.3,\pi ]$, additional radiation not reflected by the $\Delta \phi$ measurement and extrapolation can still occur and modify the $p_{\mathrm {T}}$ balance between the $Z$ boson and the leading jet with respect to expectations for true back-to-back topologies. Therefore, events with energetic sub-leading jets are vetoed. Systematic uncertainties associated with this second jet veto are studied, and the mean $p_{\mathrm {T}}$ balances in the data and the simulation are compared when applying different second jet vetoes. The nominal

$$\begin{aligned} p_{\text {T}}^{\,\text {jet}2,\text {nom}}=\text {max}\left\{ 12~\mathrm{GeV },\, 0.2\times p_{\mathrm {T}}^{Z} \right\} \end{aligned}$$

is varied up and down to

$$\begin{aligned} \begin{array}{l@{\quad }l}p_{\text {T}}^{\,\text {jet}2,\text {nom}\downarrow } = \max \left\{ 10~\mathrm{GeV },\, 0.1\times p_{\mathrm {T}}^{Z} \right\} &{} \text {(up)} \\ p_{\text {T}}^{\,\text {jet}2,\text {nom}\uparrow } = p_{\text {T}}^{\,\text {jet}2,\text {nom}} + 0.1\times p_{\mathrm {T}}^{Z} &{} \text {(down)}. \end{array} \end{aligned}$$

The average of the absolute values of the two deviations is taken as a systematic uncertainty on the data-to-MC ratio.

#### Out-of-cone radiation and underlying event

This $k_\mathrm {OOC}$ factor defined in Eq. (12) and measured as described in Sect. 9.4 indicates how the $Z$ boson’s $p_{\mathrm {T}}$ differs from the truth jet’s $p_{\mathrm {T}}$. In order to evaluate the systematic uncertainties coming from out-of-cone radiation and the underlying event, this factor is applied to the $Z$ boson’s $p_{\mathrm {T}}$. It is measured in the data and in the simulation in bins of $p_{\mathrm {T}}^{\text {ref}}$. Its value depends on the $p_{\mathrm {T}}$ as well as on the jet size. For jets with $R=0.4$, $k_\mathrm {OOC}$ increases from about 0.93 at low $p_{\mathrm {T}}$ to about 0.99 at high $p_{\mathrm {T}}$. For jets with $R=0.6$, it varies between 1 and 1.02 without any systematic $p_{\mathrm {T}}$ dependence. A modified data-to-MC ratio of the balance is calculated using the $k_\mathrm {OOC}$ factors and the difference with respect to the nominal ratio is taken as a systematic uncertainty.

#### Impact of additional pile-up interactions

The impact of in-time and out-of-time pile-up is studied by comparing the $p_{\mathrm {T}}$ balance in two samples with different numbers of primary vertices ($N_{\mathrm{PV}}\le 5$ and $N_{\mathrm{PV}}>5$), and two samples with different average number of interactions per bunch crossing ($\mu <8$ and $\mu >8$). The differences observed between the samples are small compared to the uncertainty on the pile-up offset correction (see Sect. 6.4). Therefore, they are not taken into account in this analysis in order to avoid double-counting between the different steps of the jet calibration procedure.

The direct impact of additional interactions on the leading jet is also studied by relaxing the $\mathrm {JVF}$ cut, introduced in Sect. 9.2, for the leading jet. The difference with respect to the nominal result is taken as an additional uncertainty.

#### Electron energy scale

The $p_{\mathrm {T}}$ of the $Z$ boson, measured from the energy of the electrons, is used as a reference to probe the jet energy scale. The electron energy is shifted upwards and downwards according to the uncertainty on its measurement [72], updated using data recorded in 2011.

#### Impact of the Monte Carlo generator

The mean balances are obtained from Pythia and Alpgen samples, using the procedure described in Sect. 9.3. The difference between the data-to-Pythia and the data-to-Alpgen ratios is taken as a systematic uncertainty. The Alpgen MC generator uses different theoretical models for all steps of the event generation and therefore gives a reasonable estimate of the systematic variations. However, the possible compensation of modelling effects that shift the jet response in opposite directions cannot be excluded. To reduce the impact of statistical fluctuation the first three bins are merged, since they give the same result within their statistical uncertainties.

**Fig. 19:**
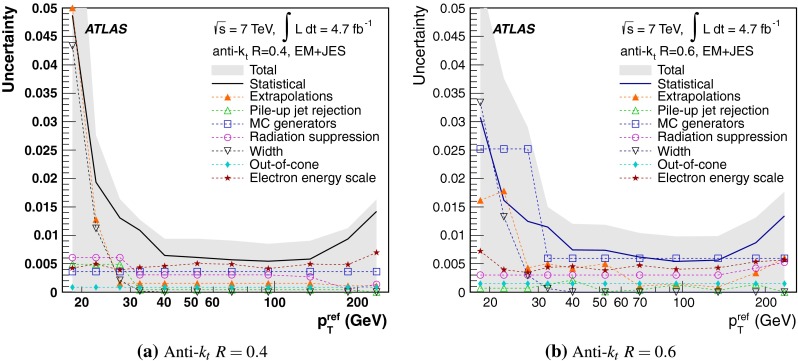
Summary of the $Z$-jet uncertainties on the data-to-MC ratio of the mean $p_{\mathrm {T}}$ balance, for anti-$k_{t}$ jets with distance parameter **a** $R=0.4$ and **b** $R=0.6$ calibrated with the EM+JES scheme

**Fig. 20:**
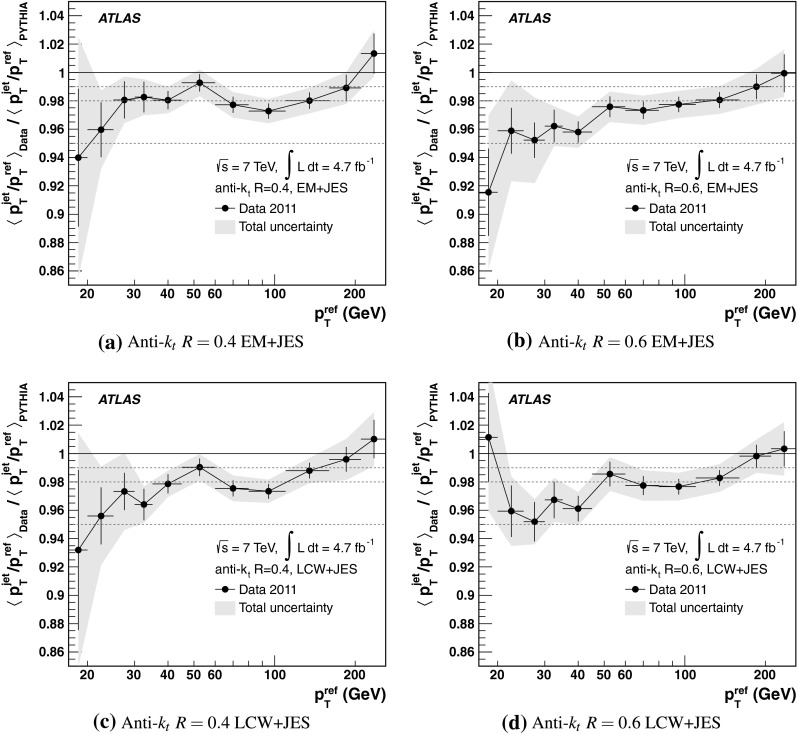
Data-to-MC ratio of the mean $p_{\mathrm {T}}$ balance for $Z$-jet events as a function of $p_{\mathrm {T}}^{\text {ref}}$ for anti-$k_{t}$ jets with distance parameter (**a**, **c**) $R=0.4$ and (**b**, **d**) $R=0.6$ calibrated with the (**a**, **b**) EM+JES and the (**c**, **d**) LCW+JES schemes. The total uncertainty on this ratio is depicted by *grey bands*. *Dashed lines* show the $-$1, $-$2, and $-$5 % shifts

#### Summary of systematic uncertainties

Additional sources of uncertainties are considered:

1. The main background to $Z$-jet events is from multijet events, and its fraction in the selected events is only of the order of 3 % [84]. Furthermore, jets passing the electron identification cuts contain an important electromagnetic component and the detector response should therefore be similar to the response for true electrons. No additional systematic uncertainty is considered for the contamination of $Z$-jet events with background events.
2. As already mentioned, the uncertainty on the pile-up offset correction is treated as extra uncertainty (see Sect. 6.4)
3. The uncertainty induced by quark and gluon response differences as well as different quark and gluon compositions in data and in the simulation is addressed in Sect. 18.

In the final evaluation of systematic uncertainties, only effects that are significant with respect to their statistical uncertainties are taken into account [85]. The systematic effects and their statistical uncertainties are first evaluated using the initial binning. Then the results in neighbouring bins are iteratively combined until the observed effects become significant. The quadratic sum of all the components previously described is taken as the overall systematic uncertainty. Figure 19 summarises the different contributions to the total uncertainty, for EM+JES jets, in the whole $p_{\mathrm {T}}$ range. For $R=0.4$ jets and $25~\mathrm{GeV }<p_{\mathrm {T}}^{\text {ref}}<260 ~\mathrm{GeV }$, uncertainties are typically between 1 and 2 %, and increase up to 10 % for low transverse momenta.

### Summary of the *Z*-jet analysis

The two ATLAS jet energy calibration schemes EM+JES and LCW+JES are probed using the direct $p_{\mathrm {T}}$ balance between a central jet and a $Z$ boson. The responses measured in the data and in the simulation are compared for jets defined by the anti-$k_{t}$ clustering algorithm with distance parameters of $R=0.4$ and $R=0.6$.

Figure 20 shows the data-to-MC ratio of the mean $p_{\mathrm {T}}$ balance for jets calibrated with the EM+JES and the LCW+JES schemes, with statistical and systematic uncertainties. For $R=0.4$ jets and $p_{\mathrm {T}}^{\text {ref}}>25~\mathrm{GeV }$, this ratio is shifted by at most $-$4 % from unity, and typically by $-$2 % over most of the $Z$ boson $p_{\mathrm {T}}$ range. Uncertainties are typically between 1 and 2 % for $25 <p_{\mathrm {T}}^{\text {ref}}<260~\mathrm{GeV }$, and increase up to 10 % for low transverse momenta.

## Jet energy calibration using $\varvec{\gamma }$-jet events

### In situ jet calibration techniques

Two in situ techniques probing the calorimeter response to the jet balancing the photon are employed in this analysis:

1. **Direct** $\varvec{p}_{\mathbf {T}}$ **balance (DB)** The transverse momentum of the jet with the highest $p_{\mathrm {T}}$ is compared to the transverse momentum of the reference photon ($p_{\mathrm {T}}^{\mathrm {\gamma }}$). The response is then computed as the ratio $p_{\mathrm {T}}^\mathrm {jet}/p_{\mathrm {T}}^{\mathrm {\gamma }}$.
2. **Missing transverse momentum projection fraction (MPF)** The total hadronic recoil is used to estimate the calorimeter response to jets. The hadronic recoil is reconstructed from the vectorial sum of the transverse projections of the energy deposits in the calorimeter projected onto the photon direction. As in the direct $p_{\mathrm {T}}$ balance, the photon $p_{\mathrm {T}}$ serves as reference. The MPF response is defined as
  $$\begin{aligned} \mathcal {R}_\mathrm{MPF}{} = 1 + \frac{ \vec {p}_\mathrm{T}^{\gamma } \cdot \vec {E}_{\mathrm {T}}^{\mathrm {miss}}}{|p_{\mathrm {T}}^{\mathrm {\gamma }}|^2}, \end{aligned}$$
  where the $\vec {E}_{\mathrm {T}}^{\mathrm {miss}}$ is computed with topo-clusters at the EM or LCW scales. A more detailed description of these two techniques can be found in Ref. [3].

Each technique has different sensitivities to additional soft-parton radiation, as well as to pile-up. The MPF is in general less sensitive to additional particle activity that is symmetric in the transverse plane, like for example pile-up and the underlying event.

The explicit use of jets in the jet response measurement from DB makes this technique clearly dependent on the jet reconstruction algorithm. Conversely, the dependence of the MPF technique on the jet algorithm is relegated to a second-order effect.^10^ Thus, in the following, when presenting the results from the MPF technique, no jet algorithm is in general explicitly mentioned.

### Event selection of $\gamma$-jet events

The event selection used in this analysis is basically the same as that described in Ref. [3] for the 2010 analysis, except for changes that are either to adapt to the higher instantaneous luminosity of the 2011 dataset or to the different detector conditions. The event selection proceeds as follows:

1. Events are required to have a primary vertex, as defined in Sect. 5.4, with at least five associated tracks ($N_{\mathrm {vertex}}^{\mathrm {tracks}} \ge 5$).
2. There must be at least one reconstructed photon; the highest-$p_{\mathrm {T}}$ (leading) photon is taken as the hard-process photon and must have $p_{\mathrm {T}}^{\mathrm {\gamma }}> 25$ GeV.
3. The event is required to pass a single-photon trigger, with trigger $p_{\mathrm {T}}$ threshold depending on the $p_{\mathrm {T}}$ of the leading photon.
4. The leading photon must pass strict identification criteria [86], meaning that the pattern of energy deposition in the calorimeter is consistent with the expected photon showering behaviour.
5. The leading photon must lie in the pseudorapidity range $|\eta ^{\gamma }| < 1.37$, meaning it is fully contained within the electromagnetic barrel calorimeter.
6. Jets with high electromagnetic content (e.g., jets fluctuating to a leading $\pi ^{0}$, with $\pi ^{0} \rightarrow \gamma \gamma$) may be misidentified as photons. In order to reduce this background, the leading photon is required to be isolated from other activity in the calorimeter. The isolation variable ($E_{\mathrm {T}}^{\gamma ~\mathrm {Iso}}$) [86] is computed in a cone of size $R = 0.4$ around the photon, and corrected for pile-up energy inside the isolation cone. Only photons with $E_{\mathrm {T}}^{\gamma ~\mathrm {Iso}} < 3$ GeV are selected.
7. The photon reconstruction algorithm attempts to retain photons that have converted into an electron-positron pair. While clusters without matching tracks are directly classified as “unconverted” photon candidates, clusters matched to pairs of tracks originating from reconstructed conversion vertices are considered as “converted” photon candidates (double-track conversions). To increase the reconstruction efficiency of converted photons, conversion candidates where only one of the two tracks is reconstructed (single-track conversions) are also retained. Jets that are misidentified as photons fall more often in the category of converted photons, because fake photons produce wider showers and have tracks associated to them. To suppress this background further, the ratio of the transverse energy of the photon candidate cluster to the scalar sum of the $p_{\mathrm {T}}$ of the matching tracks ($E_{\mathrm {T}}^{\gamma ~{\mathrm {cluster}}}/(\sum p_{\mathrm {T}}^{\mathrm {tracks}})$) is required to be in the range from 0 to 2 for single-track conversions, and from 0.5 to 1.5 for double-track conversions. The fraction of converted photons is $\sim$30 % throughout the $p_{\mathrm {T}}^{\mathrm {\gamma }}$ range under consideration.
8. Only jets with $p_{\mathrm {T}}^\mathrm {jet}> 12$ GeV are considered. From those, only jets that pass quality criteria designed to reject fake jets originating from noise bursts in the calorimeters or from non-collision background or cosmics (see Sect. 5.3), are used. After these jet selections, each event is required to have at least one jet.
9. The highest-$p_{\mathrm {T}}$ (leading) jet must be in the region $|\eta ^{\mathrm {jet}}| < 1.2$. This choice is motivated by the small $\eta$-intercalibration correction below 1.5 % in this region.
10. To suppress soft radiation that would affect the $p_{\mathrm {T}}$ balance between the jet and the photon, the following two conditions are required:
  - The leading jet must be back-to-back to the photon in the transverse plane ($\Delta \phi (\mathrm {jet},\gamma ) > 2.9$ rad).
  - The $p_{\mathrm {T}}$ of the sub-leading jet from the hard process ($p_{\mathrm {T}}^{\mathrm {jet2}}$) must be less than 20 % (30 %) of the $p_{\mathrm {T}}$ of the photon for DB (MPF^11^). In order to distinguish jets from the hard process against jets from pile-up, the sub-leading jet is defined as the highest-$p_{\mathrm {T}}$ jet from the subset of non-leading jets that either have $\mathrm {JVF}> 0.75$ or for which $\mathrm {JVF}$ could not be computed because they are outside the region covered by the tracking system. See Sect. 8.2.3 for the explanation of $\mathrm {JVF}$.
11. In the case of DB, the event is rejected if either the leading jet or the sub-leading jet falls in a region where, for a certain period, the read-out of the EM calorimeter was not functioning. For MPF, the condition is extended to all jets with $p_{\mathrm {T}}^\mathrm {jet}> 20$ GeV in the event. A similar condition is imposed on the reference photon.

A summary of the event selection criteria is given in Table 3. Table 4 shows the approximate number of selected events per $p_{\mathrm {T}}^{\mathrm {\gamma }}$ bin.

**Table 3:** Summary table of the criteria to select $\gamma \text {-jet}$ events

Variable	Selection
$N_{\mathrm {tracks}}^{\mathrm {vertex}}$	$>$4
$p_{\mathrm {T}}^{\mathrm {\gamma }}$	$>$25 GeV
$|\eta ^{\gamma }|$	$<$1.37
$p_{\mathrm {T}}^\mathrm {jet}$	$>$12 GeV
$|\eta ^{\mathrm {jet}}|$	$<$1.2
$E_{\mathrm {T}}^{\gamma ~\mathrm {Iso}}$	$<$3 GeV
$\Delta \phi _{\mathrm{jet}\text{- }\gamma }$	$>$2.9 rad
$p_{\mathrm {T}}^{\mathrm {jet2}}/p_{\mathrm {T}}^{\mathrm {\gamma }}$	$<$0.2 for DB ($<$0.3 for MPF)

**Table 4:** Table with the approximate number of selected events in each $p_{\mathrm {T}}^{\mathrm {\gamma }}$ bin

$p_{\mathrm {T}}^{\mathrm {\gamma }}$ (GeV)	Events	$p_{\mathrm {T}}^{\mathrm {\gamma }}$ (GeV)	Events
25–45	20480	210–260	10210
45–65	61220	260–310	4650
65–85	125040	310–400	2770
85–110	262220	400–500	800
110–160	143180	500–600	240
160–210	32300	600–800	100

**Fig. 21:**
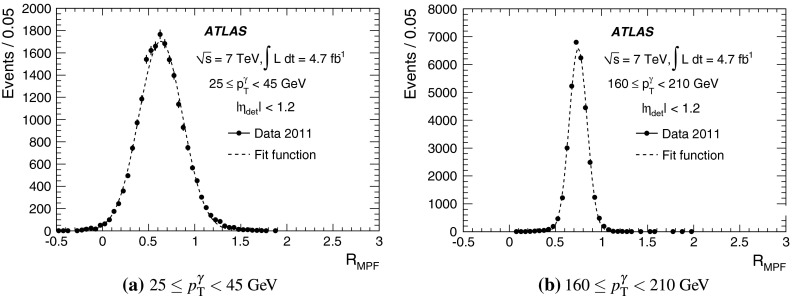
MPF response distributions in the $\gamma \text {-jet}$ data for **a** $25 \le p_{\mathrm {T}}^{\mathrm {\gamma }}< 45$ GeV and **b** $160 \le p_{\mathrm {T}}^{\mathrm {\gamma }}< 210$  GeV when using topo-clusters at the EM scale. The *dashed lines* represent the fits with a Gaussian function. The mean value from the fit in each $p_{\mathrm {T}}^{\mathrm {\gamma }}$ bin is the value used as the measured average MPF response

**Fig. 22:**
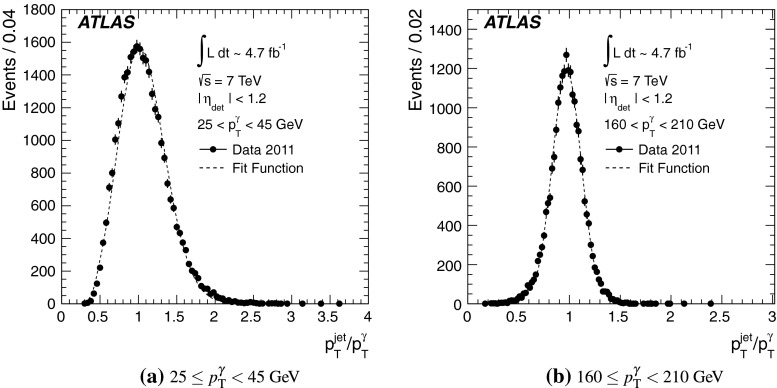
Jet response distributions in the $\gamma \text {-jet}$ data for **a** $25 \le p_{\mathrm {T}}^{\mathrm {\gamma }}< 45$ GeV and **b** $160 \le p_{\mathrm {T}}^{\mathrm {\gamma }}< 210$ GeV as measured by the DB technique for anti-$k_{t}$ jets with $R = 0.6$ at the EM+JES scale. The *dashed lines* represent fits of Gaussian functions, except in the lowest bin ($25 \le p_{\mathrm {T}}^{\mathrm {\gamma }}< 45$ GeV), where the fit function is a Poisson distribution. The mean value from the fit in each $p_{\mathrm {T}}^{\mathrm {\gamma }}$ bin is the value used as the measured average jet response in DB

**Fig. 23:**
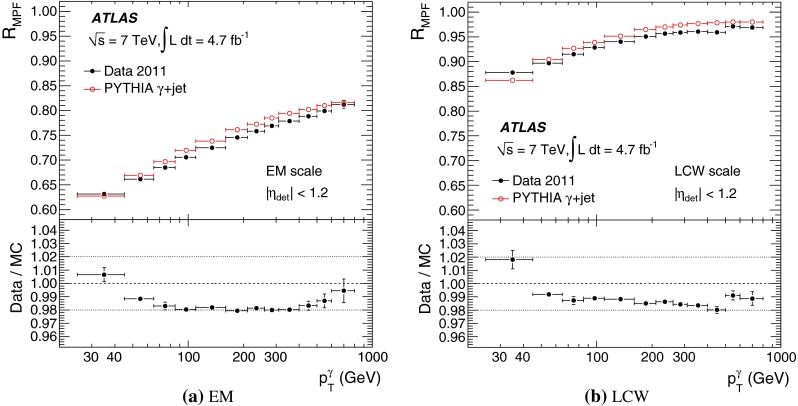
Average jet response as determined by the MPF technique in $\gamma \text {-jet}$ events using topo-clusters at the **a** EM and **b** LCW energy scales, for both data and MC simulations, as a function of the photon transverse momentum. The data-to-MC response ratio is shown in the *bottom inset* of each figure. Only the statistical uncertainties are shown

### Jet response measurement

**Fig. 24:**
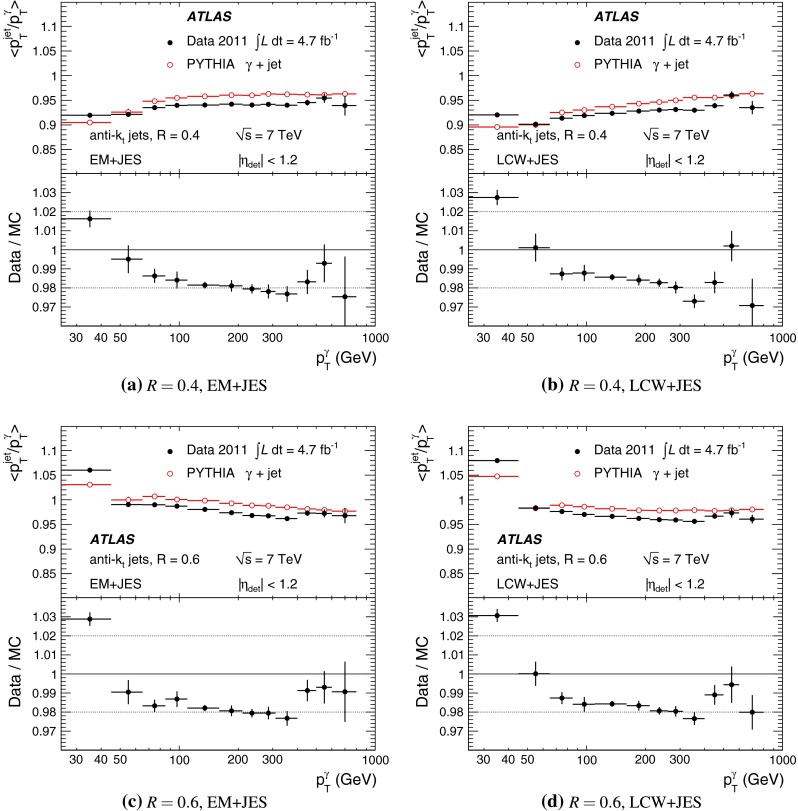
Average jet response as determined by the DB technique in $\gamma \text {-jet}$ events for anti-$k_{t}$ jets with (**a**, **b**) $R = 0.4$ and (**c**, **d**) $R = 0.6$, calibrated with the (**a**, **c**) EM+JES scheme and with the (**b**, **d**) LCW+JES scheme, for both data and MC simulations, as a function of the photon transverse momentum

The calorimeter response to jets is measured in bins of the photon transverse momentum. Distributions of the MPF and the jet responses in the data are shown in Figs. 21 and 22, respectively, for $25 \le p_{\mathrm {T}}^{\mathrm {\gamma }}{} < 45$ GeV and for $160 \le p_{\mathrm {T}}^{\mathrm {\gamma }}{} < 210$ GeV. The distributions are fitted with a Gaussian function, except in the lowest $p_{\mathrm {T}}^{\mathrm {\gamma }}$ bin for DB where a Poisson distribution is used to address the issues introduced by the jet reconstruction $p_{\mathrm {T}}$ threshold, as discussed in Sect. 9.3. The mean values from the fits define the average MPF and DB jet responses for each $p_{\mathrm {T}}^{\mathrm {\gamma }}$ bin. Figure 23 presents the results obtained in data and MC simulations for MPF when the ${E}_{\mathrm {T}}^{\mathrm {miss}}$ is calculated from topo-clusters at the (a) EM and (b) LCW scales. Figure 24 shows the results for DB for anti-$k_{t}$ jets with radius parameter $R = 0.4$ and $R = 0.6$ for the EM+JES and LCW+JES calibration schemes.

With increasing jet energies, the particles inside the jet get more energetic as well. Higher incident energies for hadrons in non-compensating calorimeters, like the ones in ATLAS, increase the amount of energy invested in intrinsically induced electromagnetic showers, thus leading to an increase of the calorimeter response [87]. This increase is clearly observed for MPF, especially when topo-clusters at the EM scale are used as for the observations shown in Fig. 23a. For DB, the effect is masked, because the jets used are already calibrated. DB is, in this case, measuring calibration residuals only.

Furthermore, a comparison of the MPF responses at EM scale in Fig. 23a and LCW scale in Fig. 23b shows the effect of having applied the LCW calibration to the topo-clusters. The response for jets built from LCW topo-clusters is much closer to unity, because the response differences between electromagnetic and hadronic particles in the jet are largely corrected by LCW at the level of the topo-clusters.

The lower part in Figs. 23 and 24 shows the ratio of the response in data to that in MC simulations. The MC simulation features a response that is 1–2 % higher than that in data for $p_{\mathrm {T}}^{\mathrm {\gamma }}> 110$ GeV. For lower values of $p_{\mathrm {T}}^{\mathrm {\gamma }}$, the data-to-MC ratio tends to increase. Systematic studies have shown that the increase at low $p_{\mathrm {T}}$ is due to the presence of contamination from multijet background events in the data, the different out-of-cone energy observed in data and in MC simulations, and the different effect of the 12 GeV jet $p_{\mathrm {T}}$ reconstruction threshold (due to differences in the jet $p_{\mathrm {T}}$ spectrum) on the response in data and in MC simulations.

### Systematic uncertainties of photon–jet balance

The following sections briefly describe the procedure to estimate the systematic uncertainties of the $\gamma \text {-jet}$ in situ techniques. The dominant sources of systematic uncertainties, for $p_{\mathrm {T}}^{\mathrm {\gamma }}\lesssim 75$ GeV, are the purity of the $\gamma \text {-jet}$ data sample and for DB also the out-of-cone correction (see Sect. 10.4.7) in the case of $R = 0.4$ jets. For $p_{\mathrm {T}}^{\mathrm {\gamma }}\gtrsim 75$ GeV, the uncertainty on the energy scale of the photon dominates.

#### Influence of pile-up interactions

The influence of in-time pile-up is evaluated by comparing the response in events with six or more reconstructed primary vertices ($N_{\mathrm{PV}}\ge 6$) to the response in events with one or two reconstructed primary vertices, inclusively in $\mu$. Similarly, the effect of out-of-time pile-up is estimated comparing the response in events with $\mu > 7$ to the response in events with $3.5 < \mu < 5.5$, inclusively in $N_{\mathrm{PV}}$. Since these two comparisons are highly correlated, the pile-up uncertainty is estimated in each $p_{\mathrm {T}}^{\mathrm {\gamma }}$ bin as the maximum difference between the two high pile-up responses and the two low pile-up responses. For MPF, the uncertainty due to pile-up is typically about $\sim$0.5 % or smaller.

In the case of DB however, the jet $p_{\mathrm {T}}$ is already corrected for the additional energy from pile-up interactions, as detailed in Sect. 5. The variations in the data-to-MC response ratio obtained with the procedure explained above are found to be much smaller than other uncertainties on the measurement. They are also well contained within the variations obtained by propagating the uncertainty on the pile-up offset correction (see Sect. 16.2).

#### Soft-radiation suppression

The stability of the data-to-MC response ratio under soft radiation is evaluated in two steps. First, the cut on the $p_{\mathrm {T}}$ of the sub-leading jet is varied, while keeping $\Delta \phi (\mathrm {jet},\gamma )$ fixed to its nominal cut value, and second, the cut on $\Delta \phi (\mathrm {jet},\gamma )$ is varied, with the cut on the sub-leading jet fixed to its nominal value. The cut on the sub-leading jet is varied to looser or tighter values as follows:

1. Tight:
  $$\begin{aligned} \begin{array}{l@{\quad }l} p_{\mathrm {T}}^{\mathrm {jet2}}< \max \left\{ 10~\mathrm{GeV }, 0.2\times p_{\mathrm {T}}^{\mathrm {\gamma }}\right\} &{} \mathrm {for\ \mathrm{MPF}{},\ and} \\ p_{\mathrm {T}}^{\mathrm {jet2}}< \max \left\{ 10~\mathrm{GeV }, 0.1\times p_{\mathrm {T}}^{\mathrm {\gamma }}\right\} &{} \mathrm {for\ \mathrm{DB}{}.} \end{array} \end{aligned}$$
2. Loose:
  $$\begin{aligned} \begin{array}{l@{\quad }l} p_{\mathrm {T}}^{\mathrm {jet2}}< \max \left\{ 12~\mathrm{GeV }, 0.3\times p_{\mathrm {T}}^{\mathrm {\gamma }}\right\} + 0.1\times p_{\mathrm {T}}^{\mathrm {\gamma }}&{} \mathrm {for\ \mathrm{MPF}{},\ and} \\ p_{\mathrm {T}}^{\mathrm {jet2}}< \max \left\{ 12~\mathrm{GeV }, 0.2\times p_{\mathrm {T}}^{\mathrm {\gamma }}\right\} + 0.1\times p_{\mathrm {T}}^{\mathrm {\gamma }}&{} \mathrm {for\ \mathrm{DB}{}.} \end{array} \end{aligned}$$

The typical variation on the data-to-MC response ratio is of the order of 0.5 % for DB and smaller for MPF. Similar variations are observed when the $\Delta \phi (\mathrm {jet},\gamma )$ cut is relaxed to be $\Delta \phi (\mathrm {jet},\gamma ) > 2.8$ or tightened to be $\Delta \phi (\mathrm {jet},\gamma ) > 3.0$. Other tests of the stability of the data-to-MC response ratio under soft radiation are explored, such as relaxing and tightening the $\Delta \phi (\mathrm {jet},\gamma )$ and $p_{\mathrm {T}}^{\mathrm {jet2}}$ selection criteria at the same time, and lead to similar results.

**Fig. 25:**
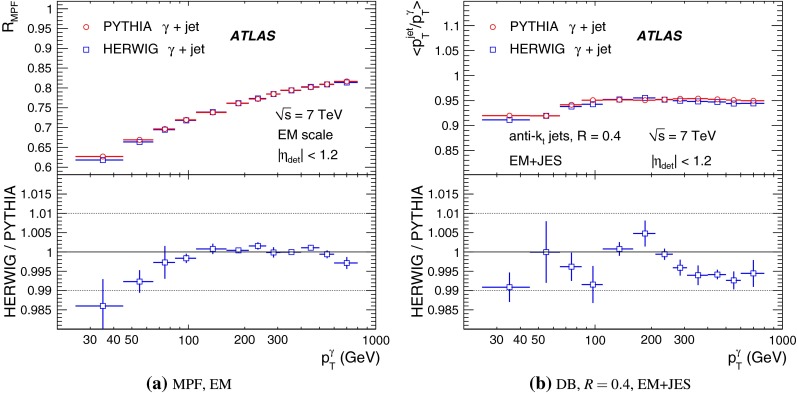
Average jet response as determined by the **a** MPF and **b** DB techniques, using anti-$k_{t}$ jets with $R = 0.4$ at the EM and EM+JES energy scales respectively, for Pythia (*circles*) and Herwig (*squares*) MC simulations, as a function of the photon transverse momentum. The HERWIG-to-PYTHIA response ratio is shown in the *bottom inset* of each figure. Only the statistical uncertainties are shown

#### Background from jet events

The uncertainty on the response due to the presence of jets that are identified as photons (fakes) in the data can be estimated, to first order, as $(1-P)\times (\mathcal {R}_{\mathrm {dijet}} - \mathcal {R}_{\gamma \text {-jet}})/\mathcal {R}_{\gamma \text {-jet}}$, where $P$ is the purity of the $\gamma \text {-jet}$ sample, and $\mathcal {R}_{\gamma \text {-jet}}$ and $\mathcal {R}_{\mathrm {dijet}}$ are the responses in signal and background events, respectively.

The difference in response is estimated from MC simulations as in the 2010 analysis [3], using the nominal signal Pythia sample, and an inclusive jet Pythia sample (see Sect. 3) enriched in events with narrow jets, which are more likely to be misidentified as photons. The comparisons indicate that the relative response differences are below 5 % for both techniques, which is taken as a conservative estimate. This is also confirmed by studying the response variation after relaxing the photon identification criterion.

The determination of the purity of the $\gamma \text {-jet}$ data sample is done in the data using a sideband technique which is described in detail in Refs. [3, 86]. The purity is about 60 % at $p_{\mathrm {T}}^{\mathrm {\gamma }}= 40$ GeV, rises with $p_{\mathrm {T}}^{\mathrm {\gamma }}$, and becomes greater than 95 % for $p_{\mathrm {T}}^{\mathrm {\gamma }}\gtrsim 200$ GeV. This purity is lower than that measured in the 2010 analysis [3], due to the larger number of pile-up events in the 2011 data. The effect of pile-up is tested by measuring the purity under the same high and low pile-up conditions used to estimate the uncertainty on the response due to pile-up (see Sect. 6). Variations in the purity of the order of 5–10 % are found. The systematic uncertainty on the purity measurement is not taken into account in the estimation of the uncertainty due to background events, because it becomes negligible when multiplied by the relative response difference between the signal and background events.

The same purity estimate is used for MPF and DB, since both techniques have the same photon selection. The uncertainty due to background from jet events is $\sim$2.5 % at low $p_{\mathrm {T}}^{\mathrm {\gamma }}$, and decreases to about 0.1 % towards high $p_{\mathrm {T}}^{\mathrm {\gamma }}$.

#### Photon energy scale

The electron energy is calibrated in situ using the measurements of the $Z$ mass in $e^{-}e^{+}$ decays [72]. The main sources of the electron energy scale uncertainty are the energy loss in the interactions with the material in front of the calorimeter and the leakage of energy transversely to the topo-clusters axis. The calibration factors obtained from the $Z \rightarrow e^{-}e^{+}$ measurements are also applied to photons, with a corresponding increase in the systematic uncertainty (the difference between the electron and the photon energy scales is caused mainly by the different interaction of electrons and photons with the material in front of the calorimeter). The photon calibration and its uncertainty are propagated to the jet response measurement, leading in both techniques to an uncertainty of approximately +0.8 and $-$0.5 %, independent of $p_{\mathrm {T}}^{\mathrm {\gamma }}$.

#### Jet energy resolution

The energy resolution for jets [81] in the MC simulation is very close to the resolution observed in data. The uncertainty on the jet energy resolution measurement in data is propagated as an uncertainty in the response in MC simulations. This is done as described in Sect. 8.4.6 and Eq. (10) therein. The observed difference in response between the varied and the nominal results is defined as the systematic uncertainty due to jet energy resolution.

#### Monte Carlo generator

Uncertainties due to different modelling of the parton shower, jet fragmentation and multiple parton interactions affecting the $p_{\mathrm {T}}$ balance between the photon and the jet, can be estimated using different MC generators which implement different models. The jet response derived with Pythia is compared to the response derived using Herwig. The results are shown in Fig. 25. The central value for the jet response in MC simulations is taken from Pythia, since this is the generator used to derive the JES corrections, and the observed full difference between Pythia and Herwig is taken as a (symmetric) systematic uncertainty. The difference in the responses between Herwig and Pythia is maximally about 1 %.

**Fig. 26:**
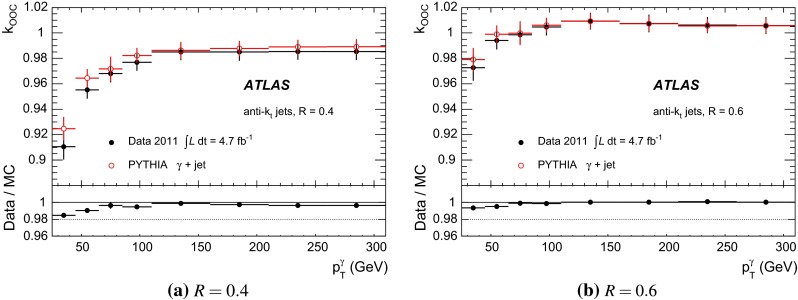
Out-of-cone radiation factor $k_\mathrm {OOC}$ relating the $p_{\mathrm {T}}$ of the photon with the $p_{\mathrm {T}}$ of the truth jet as a function of the photon transverse momentum, measured using charged particles, for anti-$k_{t}$ jets with **a** $R=0.4$ and **b** $R=0.6$, in data and in MC simulations. The data-to-MC response ratio is shown in the *bottom inset* of each plot. Statistical and systematic uncertainties are added in quadrature

**Fig. 27:**
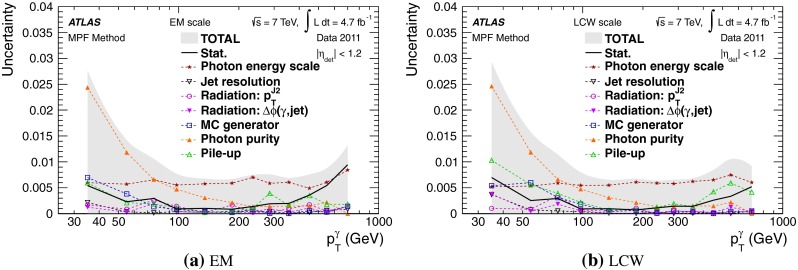
Systematic uncertainties on the data-to-MC ratio of the jet response, as determined by the MPF technique for $\gamma \text {-jet}$ events using topo-clusters at the **a** EM and **b** LCW energy scales, as a function of the photon transverse momentum

#### Out-of-cone radiation and underlying event

Even in a $2 \rightarrow 2$ $\gamma \text {-jet}$ event, where the outgoing photon and parton (quark or gluon) perfectly balance each other in transverse momentum, the transverse momentum of the photon is only approximately equal to the transverse momentum of the truth jet, formed as described in Sect. 5.5, originating from the parton. The two main reasons for this are the same already described for the $Z$-jet events in Sect. 9.4, namely the fact that the jet does not capture all particles recoiling from the photon, and the contribution to the jet from the underlying event. The amount of momentum carried by particles outside the jet and by particles coming from soft interactions not contributing to the $p_{\mathrm {T}}$ balance needs to be compared in data and MC simulation.

**Fig. 28:**
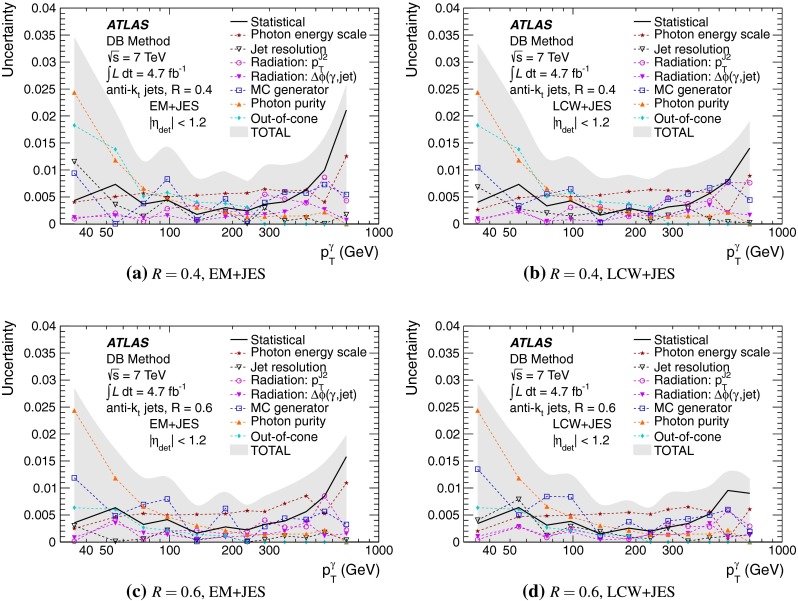
Systematic uncertainties on the data-to-MC ratio of the jet response, as determined by the DB technique in $\gamma \text {-jet}$ events, for anti-$k_{t}$ jets with (**a**, **b**) $R = 0.4$ and (**c**, **d**) $R = 0.6$, calibrated with the (**a**, **c**) EM+JES scheme and with the (**b**, **d**) LCW+JES scheme, as a function of the photon transverse momentum

When averaging over many events, particles not associated to the hard scattering are distributed isotropically, and therefore they do not contribute to the hadronic recoil vector constructed in the MPF method. Thus, their contribution to the MPF response is zero. This is also supported by studies in the MC simulation using the particles produced by the underlying event model. Moreover, in the MPF technique the photon is balanced against the full hadronic recoil, not only against the leading jet. For the DB method the out-of-cone radiation is computed as explained in Sect. 9.4.

The measured $k_\mathrm {OOC}$ factor (Eq. 12) is shown as a function of $p_{\mathrm {T}}^{\mathrm {\gamma }}$ in Fig. 26 for anti-$k_{t}$ jets with $R=0.4$ (Fig. 26a and with $R=0.6$ (Fig. 26b), for both data and MC simulations. Systematic uncertainties obtained by varying the parameters in the $k_\mathrm {OOC}$ factor definition are added in quadrature to the statistical uncertainties. The $k_\mathrm {OOC}$ varies from 0.92 (0.97) at low $p_{\mathrm {T}}$ to 0.99 (1.01) at high $p_{\mathrm {T}}$ for $R = 0.4$ (0.6), respectively. The data are described by the MC simulation within 1–2 % at low $p_{\mathrm {T}}$. This deviation is taken as a systematic uncertainty in the DB technique.

**Table 5:** Systematic uncertainties on the data-to-MC ratio of the jet response on the EM scale for both DB and MPF in two representative $p_{\mathrm {T}}^{\gamma }$ bins

$p_{\mathrm {T}}^{\gamma }$ range (GeV)	DB, $R=0.6$ (%)		MPF (%)	
	45–65	310–400	45–65	310–400
*Event*				
Pile-up	–	–	$\pm 0.21$	$\pm 0.16$
*Radiation*				
$p_{\mathrm {T}}^{\mathrm {jet2}}$	$\pm 0.43$	$\pm 0.28$	$\pm 0.09$	$\pm 0.10$
$\Delta \phi (\mathrm {jet},\gamma )$	$\pm 0.35$	$\pm 0.20$	$\pm 0.03$	$\pm 0.03$
*Photon*				
Purity	$\pm 1.18$	$\pm 0.15$	$\pm 1.18$	$\pm 0.15$
Energy	$\pm 0.46$	$\pm 0.71$	$\pm 0.57$	$\pm 0.61$
*Jet*				
JER	$\pm 0.01$	$\pm 0.11$	$\pm 0.04$	$\pm 0.01$
Out-of-cone	$\pm 0.60$	$\pm 0.00$	–	–
*Modelling*				
MC generator	$\pm 0.48$	$\pm 0.44$	$\pm 0.38$	$\pm 0.00$

#### Summary of systematic uncertainties

A summary of the systematic uncertainties for the MPF and the DB techniques as a function of the photon $p_{\mathrm {T}}$ are presented in Figs. 27 and 28, respectively. The systematic uncertainties are shown for jets calibrated with the EM and LCW schemes for MPF, and with the EM+JES and LCW+JES schemes for DB where also jets with $R=0.4$ and $R=0.6$ are considered. The figures also show the statistical uncertainty, and the total uncertainty, which corresponds to the quadratic sum of all individual components (statistical and systematic). Table 5 shows the components of the systematic uncertainty for both methods in two representative $p_{\mathrm {T}}^{\gamma }$ bins.

For the DB technique, the total uncertainty is as large as 2–3 % at very low and very high $p_{\mathrm {T}}$ values, and it is around 0.9 % in the $p_{\mathrm {T}}$ range from 100 GeV to 500 GeV. The uncertainties are smaller for MPF; the total uncertainty is $\sim$0.7 % in the range 100 GeV to 500 GeV and it is dominated by the photon energy scale uncertainty.

### Summary of the $\gamma$-jet analysis

The average jet response in events with an isolated photon and a jet at high transverse momentum is computed using the 2011 dataset, and compared to the average jet response obtained using MC simulations. Two different techniques are used, the direct $p_{\mathrm {T}}$ balance and the missing-$p_{\mathrm {T}}$ projection fraction methods. Both techniques are highly correlated and show consistent results within systematic uncertainties. The data-to-MC response ratio is close to 98 % for $p_{\mathrm {T}}^{\mathrm {\gamma }}> 85$ GeV. Systematic uncertainties are evaluated for both methods to be of the order of 1 % or smaller in most of the $p_{\mathrm {T}}^{\mathrm {\gamma }}$ range under consideration.

## High-*p*$_\mathsf{T }$ jet energy calibration using multijet events

### Multijet balance technique and uncertainty propagation

The multijet balance (MJB) technique described in Ref. [3] can be used to verify the energy scale of jets and obtain correction factors that can correct for any non-linearity at very high $p_{\mathrm {T}}$. The method exploits the $p_{\mathrm {T}}$ balance in events where the highest-$p_{\mathrm {T}}$ jet (leading jet) is produced back-to-back to a system composed of non-leading jets, referred to as a “recoil system”. The leading jet is required to have significantly larger $p_{\mathrm {T}}$ than the jets in the recoil system in order to ensure that MJB is testing the absolute high-$p_{\mathrm {T}}$ jet energy scale.

The vectorial sum of the $p_{\mathrm {T}}$ of all non-leading jets defines the transverse momentum of the recoil system ($p_{\mathrm {T}}^{\mathrm {recoil}}$) that is expected to approximately balance the $p_{\mathrm {T}}$ of the leading jet. The ratio

$$\begin{aligned} \mathrm{MJB}= \frac{|\vec {p}_{\mathrm {T}}^{\,\mathrm {leading}}|}{|\vec {p}_{\mathrm {T}}^{\,\mathrm {recoil}}|} \end{aligned}$$

thus allows the verification of the JES of the leading jet using the properly calibrated non-leading jets at a lower $p_{\mathrm {T}}$ scale. The asymmetry in the $p_{\mathrm {T}}$ scale between the leading jet and non-leading jets is established by introducing a maximum limit on the ratio between the $p_{\mathrm {T}}$ of the sub-leading (second-highest $p_{\mathrm {T}}$) jet ($p_{\text {T}}^{\,\text {jet}2}$) and $p_{\mathrm {T}}^{\mathrm {recoil}}$. The calibration for the non-leading jets in the recoil system is provided by the combination of the JES corrections derived from the $p_{\mathrm {T}}$ balance in events with a jet and a $Z$ boson (see Sect. 9) or a photon (see Sect. 10) for the absolute jet energy calibration, in addition to the $p_{\mathrm {T}}$ balance in dijet events (see Sect. 8) for the relative ($\eta _\mathrm{det}$ dependent) jet energy correction. See later Sect. 13.1 for detailed descriptions of the combination strategies in various $p_{\mathrm {T}}$ ranges.

The MJB measured in data with the corrected non-leading jets ($\mathrm{MJB}^\mathrm{Data}$) is compared with that in the simulation ($\mathrm{MJB}^\mathrm{MC}$) to evaluate the JES calibration for the leading jet and assess the systematic uncertainty for high-$p_{\mathrm {T}}$ jets. The statistical and systematic uncertainties of the $\gamma \text {-jet}$ and $Z$-jet measurements are propagated through the combination. They are taken into account, together with the systematic uncertainty of the $\eta$-intercalibration, by fluctuating each sub-leading jet four momentum within its uncertainties individually, and propagating those to higher $p_{\mathrm {T}}$ as a variation in the MJB measurement. This whole procedure is repeated by increasing the sub-leading jet $p_{\mathrm {T}}$ in steps, and applying the JES calibration derived in the previous step to the new event sample with harder non-leading jets. The MJB-based calibration is then calculated for the specific $p_{\mathrm {T}}$ range and applied in the following increase of the sub-leading jet $p_{\mathrm {T}}$. The procedure terminates once the number of events available for the next step becomes too low for a precise evaluation of MJB with the corresponding sample.

A cut on the ratio between $p_{\text {T}}^{\,\text {jet}2}$ and $p_{\mathrm {T}}^{\mathrm {recoil}}$, which defines the hard scale for the sub-leading jets, is also relaxed in the repetition sequences to effectively increase the statistics available in the calibration. The convolution of the propagated uncertainties from the JES calibrations applied to the non-leading jets with systematic uncertainties associated with the MJB method itself, as described in Sect. 11.4, gives rise to a JES systematic uncertainty across the whole jet $p_{\mathrm {T}}$ range accessible in 2011 data.

### Selection of multijet events

In order to cover a wide $p_{\mathrm {T}}$ range with enough event statistics, the analysis uses four single-jet triggers, each with a different jet-$p_{\mathrm {T}}$ threshold. The highest $p_{\mathrm {T}}$-threshold trigger that is active for the full dataset requires at least one jet with $p_{\mathrm {T}}>240$ GeV at the EM scale. The other three triggers are pre-scaled, i.e. only a defined fraction of them are recorded, and they require respective jet-$p_{\mathrm {T}}$ thresholds of 55, 100, and 135 GeV. As shown below, the analysis is not limited by the statistical accuracy even with these pre-scaled jet triggers. In the offline analysis the data collected by a given trigger are used in non-overlapping $p_{\mathrm {T}}^{\mathrm {recoil}}$ ranges where the trigger is $>$99 % efficient.

**Fig. 29:**
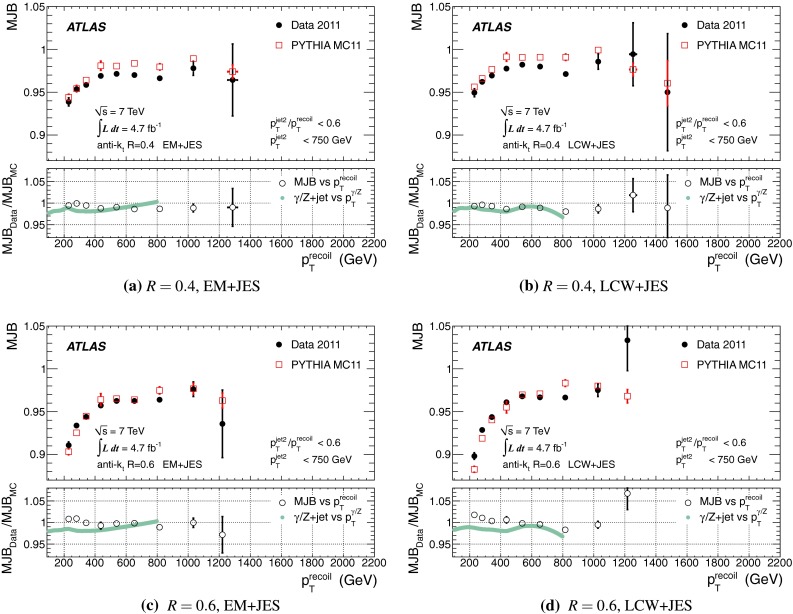
Multijet balance as a function of the recoil system $p_{\mathrm {T}}^{\mathrm {recoil}}$ for anti-$k_{t}$ jets with (**a**, **b**) $R = 0.4$ and (**c**, **d**) $R = 0.6$, calibrated with the (**a**, **c**) EM+JES scheme and with the (**b**, **d**) LCW+JES scheme, for both data and MC simulations. The non-leading jets in the data with $p_{\mathrm {T}}<750$ GeV are corrected by the combination of $\gamma \text {-jet}$ and $Z$-jet in situ calibrations as described in Sect. 11.1. The open points in the *bottom panel* show the ratio of the MJB values between data and MC simulations. The *curve* in the *same panel* shows the data-to-MC ratio of the jet $p_{\mathrm {T}}$ relative to the $p_{\mathrm {T}}$ of a photon ($p_{\mathrm {T}}^{\mathrm {\gamma }}$) or a $Z$ boson ($p_{\mathrm {T}}^{Z}$) as a function of the $p_{\mathrm {T}}^{\gamma }$ or $p_{\mathrm {T}}^{Z}$ in $\gamma \text {-jet}$ or $Z$-jet events, obtained in the combination mentioned above. Only the statistical uncertainties are shown

Only events containing at least one primary vertex, defined as described in Sect. 5.4 and associated with at least five tracks, are considered. Events are rejected if they contain either an identified lepton (electron or muon) or a photon. Events are also rejected if they contain at least one jet which has $p_{\mathrm {T}}>20$ GeV that does not pass the jet cleaning criteria discussed in Sect. 5.3 to suppress noise or detector problems and mismeasured jets. For a certain period of time the read-out of a part of the EM calorimeter was not functioning, and events containing jets pointing to the affected region are also rejected. At the last stage of the event pre-selection, events are required to have at least three good-quality jets that have $p_{\mathrm {T}}>25$ GeV and $|\eta |<2.8$. The leading jet is required to be within $|\eta |<1.2$.

**Table 6:** Summary of the event selection cuts used in the analysis. The first (second, third) values for $p_{\text {T}}^{\,\text {jet}2}$ and $p_{\text {T}}^{\,\text {jet}2}/p_{\mathrm {T}}^{\mathrm {recoil}}$ cuts are used in the first (second, subsequent) repetition of the MJB calibration procedure as described in Sect. 11.1

Variable	Cut value
Jet $p_{\mathrm {T}}$	$>$25 GeV
Jet rapidity	$|\eta |<2.8$
Leading jet rapidity	$|\eta |<1.2$
Number of good jets	$\ge$3
$p_{\mathrm {T}}^\mathrm {Recoil}$	$>$210 GeV
$\alpha$	$<$0.3 rad
$\beta$	$>$1 rad
$p_{\text {T}}^{\,\text {jet}2}$	$<$750 (1200, 1450) GeV
$p_{\text {T}}^{\,\text {jet}2}/p_{\mathrm {T}}^{\mathrm {recoil}}$	$<$0.6 (0.8, 0.8)

In order to select events having one jet produced against a well-defined recoil system, a selection is applied using two angular variables,

1. $\alpha = |\Delta \phi - \pi | < 0.3$ rad, where $\Delta \phi$ is the azimuthal opening angle between the highest-$p_{\mathrm {T}}$ jet and the recoil system, and
2. The azimuthal opening angle between the leading jet and the non-leading jet that is closest in $\phi$ ($\beta$) is required to be $\beta > 1$ rad.

Two more selection criteria ensure that the sub-leading jets have a $p_{\mathrm {T}}$ in the range where the in situ $\gamma \text {-jet}$ and $Z$-jet calibrations are available and the leading jet is well above this range. The former is achieved by requiring the sub-leading jet $p_{\text {T}}^{\,\text {jet}2}$ to be less than 750 GeV and the latter by requiring that the ratio $A$ between $p_{\text {T}}^{\,\text {jet}2}$ and $p_{\mathrm {T}}^{\mathrm {recoil}}$ satisfies $p_{\text {T}}^{\,\text {jet}2}/p_{\mathrm {T}}^{\mathrm {recoil}}< 0.6$. These two initial selections are modified when the analysis procedure is repeated as described above.

A summary of all cuts used in the analysis is given in Table 6.

**Fig. 30:**
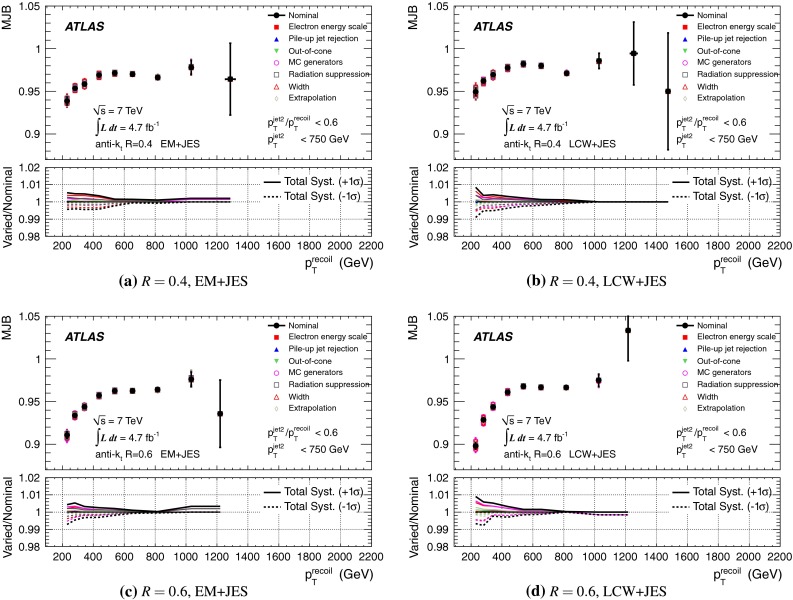
Multijet balance with the nominal and varied $Z$-jet in situ calibrations as a function of the recoil system $p_{\mathrm {T}}^{\mathrm {recoil}}$ for anti-$k_{t}$ jets with (**a**, **b**) $R = 0.4$ and (**c**, **d**) $R = 0.6$, calibrated with the (**a**, **c**) EM+JES scheme and with the (**b**, **d**) LCW+JES scheme. The varied distributions are obtained by fluctuating the jet energy scale for the non-leading jets by $\pm 1\sigma$ for each of the systematic uncertainties for the $Z$-jet calibration and repeating the analysis over the data sample. The *bottom panel* shows the relative variations of the MJB with respect to the nominal case. The *uppermost* (*lowermost*) *thick line* in the *bottom panel* shows the total variation obtained by adding all the positive (negative) variations in quadrature. The *colour* coding used in the *lower* part of the figure is the same as that used in the *upper* one

**Fig. 31:**
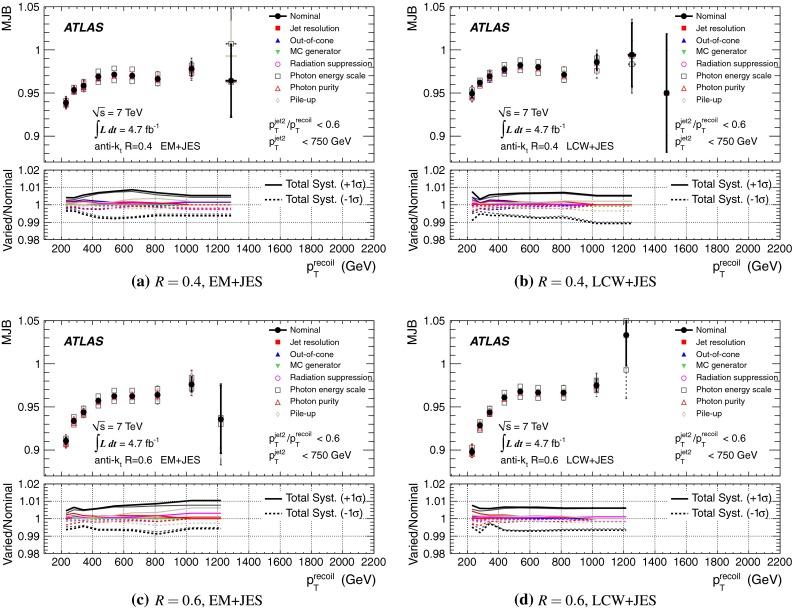
Multijet balance with the nominal and varied $\gamma \text {-jet}$ in situ calibrations as a function of the recoil system $p_{\mathrm {T}}^{\mathrm {recoil}}$ for anti-$k_{t}$ jets with (**a**, **b**) $R = 0.4$ and (**c**, **d**) $R = 0.6$, calibrated with the (**a**, **c**) EM+JES scheme and with the (**b**, **d**) LCW+JES scheme. The varied distributions are obtained by fluctuating the jet energy scale for the non-leading jets by $\pm 1\sigma$ for each of the systematic uncertainties and repeating the analysis over the data sample. The *bottom panel* shows the relative variations of the MJB with respect to the nominal case. The *uppermost* (*lowermost*) *thick line* in the *bottom panel* shows the total variation obtained by adding all the positive (negative) variations in quadrature. The *colour* coding used in the *lower* part of the figure is the same as that used in the *upper* one

**Fig. 32:**
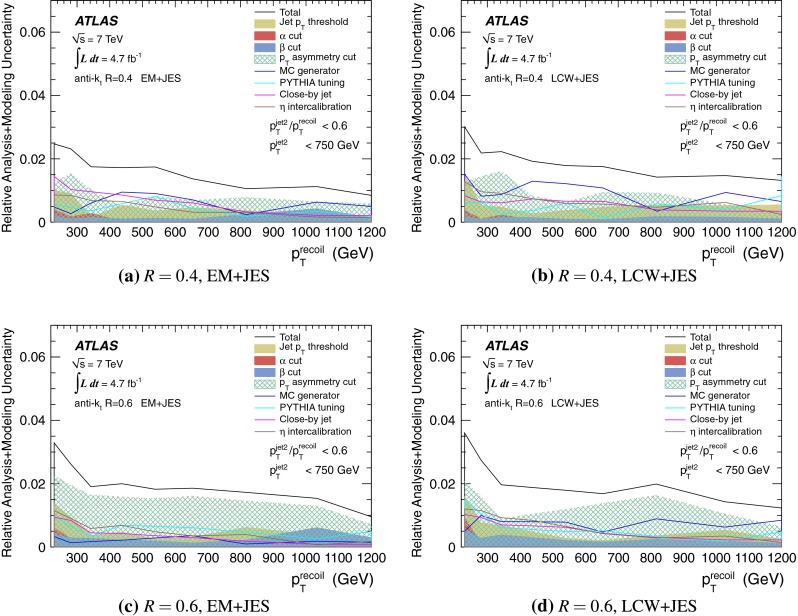
Relative uncertainties on the MJB due to the systematic uncertainty sources considered in the analysis as a function of the recoil system $p_{\mathrm {T}}$ for anti-$k_{t}$ jets with (**a**, **b**) $R = 0.4$ and (**c**, **d**) $R = 0.6$, calibrated with the (**a**, **c**) EM+JES scheme and with the (**b**, **d**) LCW+JES scheme. The *black line* shows the total uncertainty obtained as a sum of all uncertainties in quadrature

### Multijet balance measurement

The multijet balance obtained from the selected events for the EM+JES and LCW+JES calibrated jets with the anti-$k_{t}$ jet algorithm with $R = 0.4$ or $R = 0.6$ is shown in Fig. 29 for data and the MC simulations with Pythia.

The MJB decreases slightly at $p_{\mathrm {T}}^{\mathrm {recoil}}$ below 400  GeV, which is a consequence of the broadening of the $p_{\mathrm {T}}^{\mathrm {recoil}}$ distribution that can already be observed for jets formed from truth particles. The ratio between the distributions obtained from the data to the corresponding ones from MC simulations is shown in the lower part of each figure. It is compared with the data-to-MC ratio observed in the $\gamma \text {-jet}$ and $Z$-jet in situ measurements. The agreement between data and MC simulations in the $p_{\mathrm {T}}$ range covered by the $\gamma \text {-jet}$ and $Z$-jet calibration, evaluated as the average value of the data-to-MC ratio, is within 2 % (3 %) for jets with $R = 0.4$ (0.6).

### Systematic uncertainties on the multijet balance

Two main categories of systematic uncertainties are considered. The first category contains those which affect the reference $p_{\mathrm {T}}$ of the recoil system. The second category includes those that affect the MJB variables used to probe the leading jet $p_{\mathrm {T}}$, introduced mostly by effects from analysis cuts and imperfect MC modelling of the event.

The systematic uncertainty on the recoil system includes the following contributions:

1. **Absolute JES uncertainty** The standard absolute JES uncertainties obtained from the combination of $\gamma \text {-jet}$ and $Z$-jet techniques (see Sect. 13.1) are included for each jet composing the recoil system. Figures 30 and 31 show the MJB variations obtained by scaling the non-leading jet energy and momentum scale by $\pm 1 \sigma$ for each of the individual systematic uncertainties in the $\gamma \text {-jet}$ and $Z$-jet calibrations, for the four jet calibration schemes. Each source of systematic uncertainty is described in Sects. 9.5 and 10.4, respectively. In case there are fewer than 10 events in a bin, the uncertainty is taken to be the RMS of the last bin with more than 10 events divided by the square root of the number of events in that bin. The central value of the ratio is unchanged. This uncertainty ranges from 0.2 to 0.4 % for $Z$-jet and 0.6 to 1.0 % for $\gamma \text {-jet}$ in the jet $p_{\mathrm {T}}$ range of 0.5–1.2 TeV for the two jet sizes of $R = 0.4$ and 0.6.
2. **Relative JES uncertainty** Relative jet response uncertainties evaluated in the dijet $\eta$-intercalibration (Sect. 8.4) are included in a similar manner for each jet with $|\eta |<2.8$ in the recoil system.
3. **Close-by jet uncertainty** The jet response is known to depend on the angular distance to the closest jet in ($\eta ,\phi$) space [3], and the response variation is expected to be more significant for jets belonging to the recoil system. Any discrepancy between MC simulations and data in describing the jet response with close-by jets therefore results in an additional systematic uncertainty. The measurement performed to evaluate the effect and the resulting systematic uncertainty are described in Sect. 17. The close-by jet effect on MJB, shown in Fig. 32, is obtained by scaling the jet energy and momentum for each recoil jet using the results in Sect. 17.

The flavour composition of the jets could affect the agreement between MC simulations and data, and in principle cause an additional contribution to the JES uncertainty. Previous studies with 2010 data [3], however, show that the resulting uncertainty on MJB is less than 1 %, and is therefore ignored in this evaluation of systematic uncertainties.

The jet response is corrected for energy deposited by additional proton–proton collisions in the same bunch crossings using the pile-up offset correction described in Sect. 6. The residual pile-up effect on MJB is checked by comparing the MJB values using sub-samples of data and MC simulations with different $N_{\mathrm{PV}}$ and $\mu$ values. The result shows that the agreement between MC simulations and data is stable within its statistical uncertainty, and therefore an uncertainty due to pile-up is not considered.

The second systematic uncertainty category includes sources that affect the MJB variable which is used to probe the high-$p_{\mathrm {T}}$ jet energy scale. As said earlier, those are mainly due to effects from analysis cuts or imperfect MC modelling with the following considerations:

1. **Analysis cuts** A systematic uncertainty might be induced by event selection cuts on physical quantities that are not perfectly described by the MC simulation. In order to evaluate this systematic uncertainty, all relevant analysis cuts are varied in a range where the corresponding kinematic variables are not strongly biased and can be examined with small statistical fluctuations (see Table 7 for the range of variation). For each value of the cuts, the ratio of the value of MJB in data and simulation is evaluated. The maximum relative deviation of this ratio from the default value is taken as the systematic uncertainty from the source under consideration.
2. **Jet rapidity acceptance** The analysis uses only jets with $|y|<2.8$ in order to reduce the impact of the large JES uncertainties in the forward region. This selection, however, can cause additional systematic uncertainty because the fraction of jets produced outside the rapidity range can be different in the data and MC simulations, and hence affect the MJB values. This effect is checked, as is done in Ref. [3], by looking at the MJB for events with $p_{\mathrm {T}}^{\mathrm {recoil}}>210$ GeV, as a function of the total transverse energy ($\Sigma E_{\mathrm {T}}$) summed over all jets with $|y|>2.8$. The majority of events have a very small $\Sigma E_{\mathrm {T}}$ and the effect turns out to be negligible.
3. **Underlying event, fragmentation and ISR/FSR modelling** Imperfect modelling of the UE, fragmentation and ISR/FSR may influence the multijet balance by affecting variables used to select events and kinematic properties of the leading jet and the recoil system. The systematic uncertainty for each of the mentioned sources is estimated by evaluating the data-to-MC ratio of the MJB, measured using the default simulation based on Pythia and simulations using alternative MC generators. For the systematic uncertainty contribution from fragmentation, the Herwig++ samples are used as an alternative. For the underlying event and radiation modelling systematics, the Pythia Perugia 2011 [88] samples are used. The systematic uncertainty introduced by these effects is $2$ % or smaller in all cases except the lowest $p_{\mathrm {T}}^{\mathrm {recoil}}$ bins below 300 GeV.

**Table 7:** Default values and the range of variation used to evaluate the systematic uncertainty on the analysis cuts

Variable	Default	Range
Jet $p_{\mathrm {T}}$	25 GeV	20–30 GeV
$\alpha$	0.3 rad	0.1–0.4 rad
$\beta$	1.0 rad	0.50–1.50 rad
$p_{\text {T}}^{\,\text {jet}2}/p_{\mathrm {T}}^{\mathrm {recoil}}$	0.6	0.4–0.7

All systematic uncertainties due to the analysis cuts and event modelling, and the total uncertainty obtained by summing them in quadrature, are shown as a function of $p_{\mathrm {T}}^{\mathrm {recoil}}$ in Fig. 32 for jets with $R = 0.4$ and $R = 0.6$, calibrated with the EM+JES and LCW+JES schemes. The uncertainties due to dijet $\eta$-intercalibration and close-by jet effects are also included in the figure as well as the total uncertainty. Representative values of the uncertainties in the $p_{\mathrm {T}}^{\mathrm {recoil}}$ range between 0.5 and 1.2 TeV are summarised in Table 8.

**Fig. 33:**
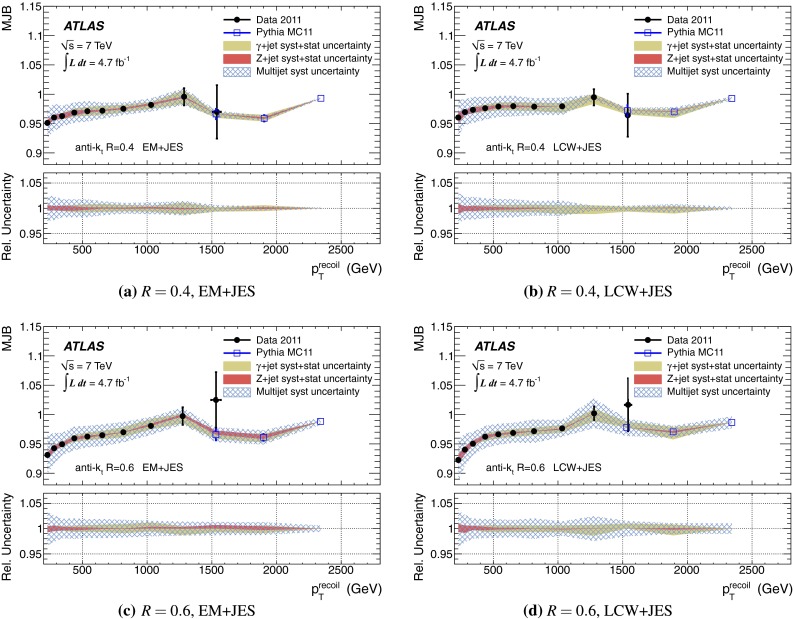
Multijet balance and systematic uncertainties related to the multijet balance technique and to the propagated uncertainties from the $\gamma \text {-jet}$ and $Z$-jet balance as a function of the recoil system $p_{\mathrm {T}}^{\mathrm {recoil}}$ for anti-$k_{t}$ jets with (**a**, **b**) $R = 0.4$ and (**c**, **d**) $R = 0.6$, calibrated with the (**a**, **c**) EM+JES scheme and with the (**b**, **d**) LCW+JES scheme. The subleading jets in the data are corrected by the combination of $\gamma \text {-jet}$ and $Z$-jet in situ calibrations at $p_{\mathrm {T}}<750$ GeV and MJB calibration at higher $p_{\mathrm {T}}$ as described in Sect. 11.1. The three systematic uncertainty bands are obtained by adding individual systematic uncertainties for each calibration technique in quadrature. Also shown are predictions of the MJB from MC simulations for the three highest $p_{\mathrm {T}}^{\mathrm {recoil}}$-values, together with their systematic uncertainties propagated by using distribution from MC simulations. The *bottom panel* shows the relative variations of the MJB with respect to the nominal case

The summary of all systematic uncertainties associated with the multijet balance technique and the propagated uncertainties from the $\gamma \text {-jet}$ and $Z$-jet in situ techniques overlaid on the data-to-MC ratio of the multijet balance, is shown in Fig. 33, for anti-$k_{t}$ jets with the distance parameters $R = 0.4$ and 0.6. The JES uncertainty is determined more precisely at jet $p_{\mathrm {T}}$ below $\sim 0.6$ TeV by the $\gamma \text {-jet}$ and $Z$-jet calibrations than the MJB calibration.

**Table 8:** Representative values of systematic uncertainties in the $p_{\mathrm {T}}^\mathrm {Recoil}$ range $500 ~\mathrm{GeV }<p_{\mathrm {T}}^\mathrm {Recoil}<1.2$ TeV for all effects considered in the analysis

Source	EM+JES		LCW+JES	
Jet size	$R = 0.4$	$R = 0.6$	$R = 0.4$	$R = 0.6$
Absolute JES (%)	0.8	0.7	0.7	0.7
Relative JES (%)	0.3	0.4	0.5	0.4
Close-by jet (%)	0.6	0.3	0.6	0.4
Jet $p_{\mathrm {T}}$ threshold (%)	$<$0.4			
$\alpha$ cut (%)	$<$0.1			
$\beta$ cut (%)	$<$0.2			
$p_{\text {T}}^{\,\text {jet}2}/p_{\mathrm {T}}^{\mathrm {recoil}}$ cut (%)	$<$0.1	1.5	$<$0.1	1.2
UE/radiation model (%)	$<$0.5			
Fragmentation model (%)	1.0	0.3	1.0	0.5

**Fig. 34:**
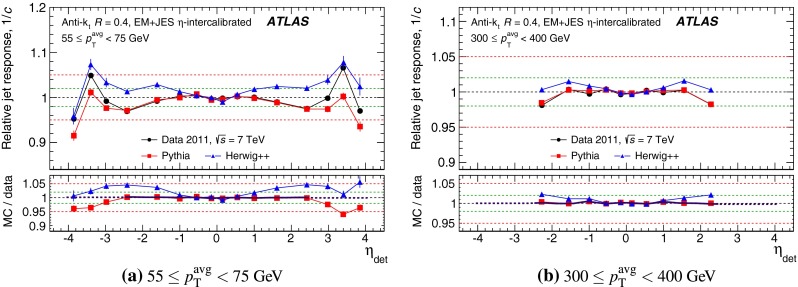
Relative jet response, $1/c$, as a function of the jet $\eta _\mathrm{det}$ for anti-$k_{t}$ jets with $R=0.4$ calibrated with the EM+JES scheme and in addition the derived $\eta$-intercalibration. Results are shown separately for **a** $55\le p_{\mathrm {T}}^\mathrm {avg}{}<75$ GeV and **b** $300\le p_{\mathrm {T}}^\mathrm {avg}{}<400$ GeV. For all points included in the original calibration ($|\eta _\mathrm{det}|<2.8$), the data are corrected to be consistent with the response in MC simulations using Pythia, as intended. The resulting calibration derived from the already calibrated data is shown as a *thick line* and is consistent with unity. The *lower* parts of the figures show the ratios between the relative jet response in data and MC

**Fig. 35:**
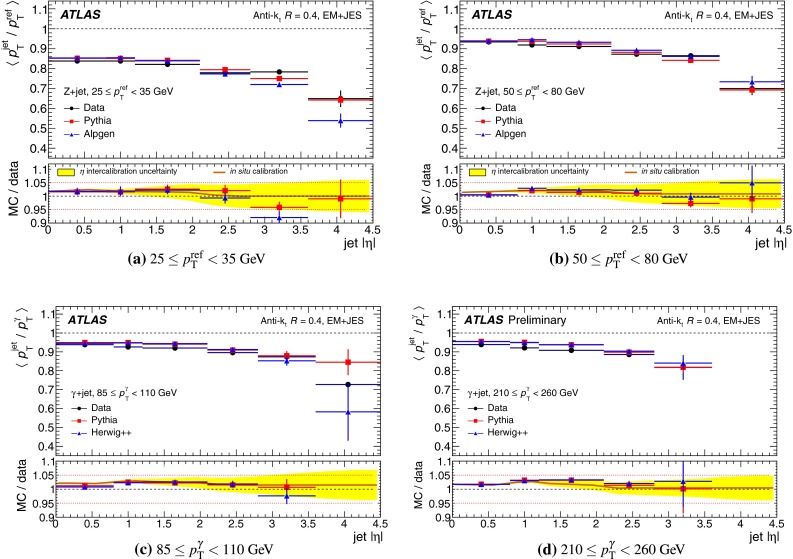
The (**a**, **b**) $Z$-jet and (**a**, **b**) $\gamma \text {-jet}$ balance for anti-$k_{t}$ jets with $R=0.4$, calibrated with the EM+JES scheme. In **a** and **b** the results for events with $25\le p_{\mathrm {T}}^{\mathrm {ref}}<35$  GeVand $50\le p_{\mathrm {T}}^{\mathrm {ref}}<80$  GeV are shown, respectively. Here $p_{\mathrm {T}}^{\mathrm {ref}}$ is the $p_{\mathrm {T}}$ of the reconstructed $Z$ boson projected onto the axis of the balancing jet. The $p_{\mathrm {T}}$ balance for $\gamma \text {-jet}$ events with photons with transverse momenta $p_{\mathrm {T}}^{\mathrm {\gamma }}$ within $85 \le p_{\mathrm {T}}^{\mathrm {\gamma }}< 100$ GeV is shown in **c**, while **d** shows the $p_{\mathrm {T}}$ balance for higher photon transverse momenta ($210 \le p_{\mathrm {T}}^{\mathrm {\gamma }}< 260$ GeV). As no in situ calibration is applied to these measurements, it is expected that data and MC simulations using Pythia are shifted relative to each other by the absolute correction multiplied by the relative ($\eta _\mathrm{det}$ dependent) correction presented herein. The resulting JES calibration is shown as a *solid line* in the *lower* part of the figures. The dijet modelling uncertainty is shown as a *filled band* around the in situ correction

### Summary of multijet analysis

The multijet balance technique is used to probe the jet energy scale in the  TeV region for anti-$k_{t}$ jets with distance parameters $R = 0.4$ and $R = 0.6$. Exploiting the $p_{\mathrm {T}}$ balance between the highest-$p_{\mathrm {T}}$ jet and the recoil system composed of jets corrected by the $\gamma \text {-jet}$ and $Z$-jet calibrations allows the extension of the in situ JES determination to higher $p_{\mathrm {T}}$, beyond the range covered by the $\gamma \text {-jet}$ calibration. Propagating systematic uncertainties associated with the $\gamma \text {-jet}$, $Z$-jet and dijet calibrations as well as the systematic uncertainty due to the knowledge of the recoil system transverse momentum in the MJB method (including the close-by jet uncertainty), the total systematic uncertainties for the $\gamma \text {-jet}$, $Z$-jet and MJB calibration methods are obtained to be about 0.6, 0.3 and 1.5 % respectively, for jets with $p_{\mathrm {T}}= 1$ TeV. At high transverse momentum, the main contribution to the systematic uncertainty is due to the uncertainty on the MJB calibration. Considering the statistical uncertainty of the MJB calibration based on the 2011 data, the high-$p_{\mathrm {T}}$ jet energy scale is validated at $p_{\mathrm {T}}>500$ GeV within 2.4 (2.0 %) and 2.2 % (3.0 %) up to 1.2 TeV for anti-$k_{t}$ jets with $R = 0.4$ and $R = 0.6$, both calibrated with the EM+JES (LCW+JES) scheme.

## Forward-jet energy measurement validation using *Z*-jet and $\varvec{\gamma }$-jet data

To test the performance of the forward-jet calibration derived in Sect. 8, this calibration is applied to all jets in the original dataset and the full analysis is repeated. The resulting intercalibration results are within 0.3 % of unity across the full $(p_{\mathrm {T}}^\mathrm {avg},\eta _\mathrm{det})$ phase space in which the calibration is derived, both for jets with $R=0.4$ and $R=0.6$, and for the EM+JES and LCW+JES calibrations. The measured relative response for two representative bins of $p_{\mathrm {T}}^\mathrm {avg}$ is shown in Fig. 34.

Similar to the analyses described in Sects. 9 and 10, the balance between a $Z$ boson decaying to an electron–positron pair and a recoiling forward jet, and the balance between a photon and a forward jet, are used to study the jet response in the forward direction. The results for $Z$-jet and $\gamma \text {-jet}$, as shown in Fig. 35, agree with the calibrations and uncertainty derived from the dijet analysis.

The $Z$-jet study also includes predictions from the Alpgen generator, which uses Herwig for parton shower and fragmentation into particles (see Sect. 3 for generator configuration details). The Alpgen+Herwig response predictions generally agree with the expectations within the modelling uncertainty of this analysis (see Sect. 8.4.1). The $\gamma \text {-jet}$ results include comparisons with Pythia events, generated with the same tune and version as the Pythia dijet samples used in this analysis, and a sample produced with Herwig, using the already mentioned ATLAS AUET2B MRST LO** tune and the MRST LO** PDF set (see Sect. 3).

**Table 9:** Summary of the number of events available for various in situ techniques after all selection cuts. The numbers are given for illustration in specific $p_{\mathrm {T}}^\mathrm {jet}$ ranges for anti-$k_{t}$ jets with $R=0.4$ reconstructed with the EM+JES scheme. The $\gamma \text {-jet}$ results are based on the MPF method

$Z$-jet method					
$p_{\mathrm {T}}^\mathrm {jet}$	20–25 GeV	35–45 GeV	210–260 GeV		
Number of events	8530	8640	309		
$\gamma \text {-jet}$ method					
$p_{\mathrm {T}}^\mathrm {jet}$	25–45 GeV	45–65 GeV	210–260 GeV	600–800 GeV	
Number of events	$20\,480$	$61\,220$	$10\,210$	100	
Multijet method					
$p_{\mathrm {T}}^\mathrm {jet}$			210–260 GeV	750–950 GeV	1.45–1.8 TeV
Number of events			$2\,638$	$3\,965$	48

## Jet energy calibration and uncertainty combination

### Overview of the combined JES calibration procedure

After the first JES calibration step described in Sect. 5, the jet transverse momenta $p_{\mathrm {T}}^\mathrm {jet}$ in data and MC simulation are compared using in situ techniques that exploit the balance^12^ between $p_{\mathrm {T}}^\mathrm {jet}$ and the $p_{\mathrm {T}}$ of a reference object ($p_{\mathrm {T}}^{\mathrm {ref}}$):

13 $$\begin{aligned} \mathcal {R}\left( p_{\mathrm {T}}^\mathrm {jet},\eta \right) = \dfrac{\left\langle p_{\mathrm {T}}^\mathrm {jet}/p_{\mathrm {T}}^{\mathrm {ref}}\right\rangle _{\mathrm {data}}}{\left\langle p_{\mathrm {T}}^\mathrm {jet}/p_{\mathrm {T}}^{\mathrm {ref}}\right\rangle _{\mathrm {MC}}} \end{aligned}$$

The inverse of this quantity is the residual JES correction factor for jets measured in data, and thus reflects the final JES calibration in ATLAS. It is derived from corrections individually described in Sect. 7. The sequence of these corrections is briefly summarised again below, with references to the corresponding more detailed descriptions:

1. Apply $\eta$-intercalibration to remove the $\eta _\mathrm{det}$ dependence of the detector response to jets within ${0.8}\le |\eta |<{4.5}$ by equalising it with the one for jets within $|\eta _\mathrm{det}|<0.8$ (see Sect. 7.1).
2. Apply the absolute correction, as derived using a combination of the $Z$-jet (Sect. 9) and the $\gamma \text {-jet}$ (Sect. 10) methods, to the central jet response ($|\eta _\mathrm{det}|<1.2$). The slightly larger $\eta _\mathrm{det}$ range used here, compared to the one used in $\eta$-intercalibration, provides more statistics while keeping systematic uncertainties small. The corresponding combined JES uncertainty is determined from the uncertainties of each of these techniques, as presented in detail in Sect. 13.3. The absolute scale correction, together with its systematic uncertainties, is also evaluated for jets in the end-cap and forward detector region ($|\eta _\mathrm{det}| \ge 1.2$), and accordingly applied to those as well.
3. Jets with energies in the  TeV regime are calibrated using the multijet transverse momentum balance technique (MJB in Sect. 11). The lower-$p_{\mathrm {T}}$ jets are within $|\eta _\mathrm{det}|<2.8$, while the leading jet is required to be within $|\eta _\mathrm{det}|<1.2$. The uncertainties derived from $\gamma \text {-jet}$, $Z$-jet and dijet $p_{\mathrm {T}}$ balance for the lower-$p_{\mathrm {T}}$ jets are propagated to the higher-$p_{\mathrm {T}}$ jets (Sect. 11.4).

The in situ JES calibration and the corresponding JES uncertainty for central jets ($|\eta _\mathrm{det}|<1.2|$) are hence derived by a combination of the data-to-MC ratios $\mathcal {R}$, individually determined as given in Eq. (13), obtained from the $\gamma \text {-jet}$, $Z$-jet and MJB correction methods. The JES uncertainties for forward jets $1.2<|\eta _\mathrm{det}|<4.5$ are then derived from those for central jets using the dijet $\eta$-intercalibration technique.

Table 9 summarises the number of events available for each correction method in various kinematic bins. Details of the combination method, including the full evaluation of the systematic uncertainties and its underlying components (nuisance parameters), are further explained in the remainder of this section.

### Combination technique

The data-to-MC response ratios (see Eq. 13) of the various in situ methods are combined using the procedure described in Ref. [3]. The in situ jet response measurements are made in bins of $p_{\mathrm {T}}^{\mathrm {ref}}$ and within $|\eta _\mathrm{det}|<1.2$, and are evaluated at the barycentre $\langle p_{\mathrm {T}}^{\mathrm {ref}}\rangle$ of each $p_{\mathrm {T}}^{\mathrm {ref}}$ bin, for each $\eta _\mathrm{det}$ range.^13^

First, a common, fine $p_{\mathrm {T}}$ binning is introduced for the combination of methods. In each of these $p_{\mathrm {T}}$ bins, and for each in situ method that contributes to that bin, the data-to-MC response ratio is determined using interpolating splines based on second-order polynomials. The combined data-to-MC ratio $\mathcal {R}_{\mathrm {extrap}}(\langle p_{\mathrm {T}}^\mathrm {jet}\rangle ,\eta _\mathrm{det})$ is then determined by the weighted average of the interpolated contributions from the various methods. The weights are obtained by a $\chi ^{2}$ minimisation of the response ratios in each $p_{\mathrm {T}}$ bin, and are therefore proportional to the inverse of the square of the uncertainties of the input measurements. The local $\chi ^{2}$ is also used to test the level of agreement between the in situ methods.

Each uncertainty source of the in situ methods is treated as fully correlated across $p_{\mathrm {T}}$ and $\eta _\mathrm{det}$, while the individual uncertainty sources inside a method and between the methods are assumed to be independent of each other. The full set of uncertainties is propagated from the in situ methods to the combined result in each $p_{\mathrm {T}}$ bin using pseudo-experiments [3]. For some applications like the combination and comparison of several experimental measurements using jets, it is necessary to understand the contribution of each uncertainty component to the final total uncertainty. For this purpose, each uncertainty component is propagated separately from each in situ method to the combined result. This is achieved by coherently shifting all the correction factors obtained by the in situ methods by one standard deviation of a given uncertainty component, and redoing the combination using the same set of averaging weights as in the nominal combination. The comparison of the shifted average correction factors with the nominal ones provides the propagated systematic uncertainty.

To account for potential disagreement between in situ measurements constraining the same term (referred to as measurements which are *in tension*), each uncertainty source is rescaled by the factor $\sqrt{\chi ^{2}/\mathrm{dof}}$, if this factor is larger than 1. This is conservative, as values of $\sqrt{\chi ^{2}/\mathrm{dof}}$ larger than 1 can also be reached due to statistical fluctuations.

$\mathcal {R}_{\mathrm {extrap}}(\langle p_{\mathrm {T}}^\mathrm {jet}\rangle ,\eta _\mathrm{det}) = 1/c$ is used as the in situ correction calibration factor and its inverse $c$ is applied to data. The correction factor still contains part of the statistical fluctuations of the in situ measurements. The influence of the statistical fluctuations is reduced by applying a minimal amount of smoothing using a sliding Gaussian kernel to the combined correction factors [3].

Each uncertainty component from the in situ methods is also propagated through the smoothing procedure. Propagating information between close-by $p_{\mathrm {T}}$ regions, the smoothing procedure changes the amplitude of the uncertainties (e.g. reducing them at low $p_{\mathrm {T}}$).

### Uncertainty sources of the in situ calibration techniques

The in situ techniques usually rely on assumptions that are only approximately fulfilled. One example is the assumption that the calibrated jet and the reference object are balanced in transverse momentum, while this balance can be altered by the presence of additional high-$p_{\mathrm {T}}$ particles. In order to determine the JES uncertainties, the modelling of physics effects has to be disentangled from detector effects. These effects can be studied by looking at the changes of the data-to-MC response ratios introduced by systematic variation of the event selection criteria. The ability of the MC simulation to describe these changes under large variations of the selection criteria determines the systematic uncertainty in the in situ methods, since physics effects can be suppressed or amplified by these variations. In addition, systematic uncertainties related to the selection, calibration and modelling of the reference object need to be considered.

When performing the variations of the selection criteria, only statistically significant variations of the response ratios are propagated to the systematic uncertainties. This is achieved by evaluating the systematic uncertainties in intervals which can be larger than the bins used for the measurement of the response ratios, meaning that several bins are iteratively combined until the observed deviations are significant. By doing so, one avoids multiple counting of the statistical uncertainties in the systematics that are evaluated. Using this approach, it is found that the radiation suppression uncertainty for the $\Delta \phi (\mathrm {jet},\gamma )$ cut on the MPF method (see Sect. 10.4.2) can be dropped.^14^

**Table 10:** Summary table of the uncertainty components for each in situ technique ($Z$-jet (see Sect. 9), $\gamma \text {-jet}$ (see Sect. 10), and multijet $p_{\mathrm {T}}$ balance (see Sect. 11) used to derive the jet energy scale uncertainty. Shown are the 21 systematic uncertainty components together with the 11, 12 and 10 statistical uncertainty components for each in situ technique. Each uncertainty component is categorised depending on its source as either detector (Detector), physics modelling (Model), mixed detector and modelling (Mixed), or as statistics and method (Stat/Meth)

Name	Description	Category
Common sources		
Electron/photon $E$ scale	Electron or photon energy scale	Detector
**DB** $Z$-jet $p_\mathrm{T}$ balance		
MC generator	MC generator difference between Alpgen/Herwig and Pythia	Model
Radiation suppression	Radiation suppression due to second jet cut	Model
Extrapolation	Extrapolation in $\Delta \phi _{\mathrm{jet}\text{- }Z}$ between jet and Z boson	Model
Pile-up jet rejection	Jet selection using jet vertex fraction	Mixed
Out-of-cone	Contribution of particles outside the jet cone	Model
Width	Width variation in Poisson fits to determine jet response	Stat/Meth
Statistical components	Statistical uncertainty for each of the 11 bins	Stat/Meth
**MPF** $\gamma \text {-jet}$ $p_{\mathrm {T}}$ balance (MPF)		
MC generator	MC generator difference Herwig and Pythia	Model
Radiation suppression	Radiation suppression due to second jet cut	Model
Jet resolution	Variation of jet resolution within uncertainty	Detector
Photon purity	Background response uncertainty and photon purity estimation	Detector
Pile-up	Sensitivity to pile-up interactions	Mixed
Out-of-cone	Contribution of particles outside the jet cone	Model
Statistical components	Statistical uncertainty for each of the 12 bins	Stat/Meth
**MJB** Multijet $p_{\mathrm {T}}$ balance		
$\alpha$ selection	Angle between leading jet and recoil system	Model
$\beta$ selection	Angle between leading jet and closest sub-leading jet	Model
Dijet balance	Dijet balance correction applied for $|\eta |<{2.8}$	Mixed
Close-by, recoil	JES uncertainty due to close-by jets in the recoil system	Mixed
Fragmentation	Jet fragmentation modelling uncertainty	Mixed
Jet $p_{\mathrm {T}}$ threshold	Jet $p_{\mathrm {T}}$ threshold	Mixed
$p_{\mathrm {T}}$ asymmetry selection	$p_{\mathrm {T}}$ asymmetry selection between leading jet and sub-leading jet	Model
UE,ISR/FSR	Soft physics effects modelling: underlying event and soft radiation	Mixed
Statistical components	Statistical uncertainty for each of the 10 bins	Stat/Meth

For the relative $\eta$-intercalibration described in Sect. 7.1 the dominant uncertainty source is due to MC modelling of jets at forward rapidities, where properties differ significantly for the generators under consideration (Pythia and Herwig). Other systematic uncertainty sources arise due to the modelling of the jet resolution, the trigger, and dijet topology selection. However, these components are negligible when compared to the MC modelling uncertainty.

The data-to-MC response ratio given in Eq. (13) for the direct balance in $Z$-jet events, the MPF technique in $\gamma \text {-jet}$ events, and the multijet balance method are combined as described in the previous Sect. 13.2. In this combination, the ability of the MC simulation to describe the data, the individual uncertainties of the in situ techniques and their compatibility, are considered. The uncertainties of the three central in situ methods combined here are described by a set of 54 systematic uncertainty sources listed in Table 10. The photon and electron energy scale uncertainties are treated as being fully correlated at this level. Components directly related to the dijet balance technique are $\eta$ dependent quantities, and are thus treated differently. Such parameters are not included in the list of the 54 components, although uncertainties related to their propagation through other methods are included. In Table 10, each uncertainty component is assigned to one of four categories, based on its source and correlations:

1. Detector description (Detector)
2. Physics modelling (Model)
3. Statistics and method (Stat/Meth)
4. Mixed detector and modelling (Mixed).

The motivation for these categories, and to some extend the guidance for assigning the 54 individual components to them, are given by considerations concerning the comparability of jet measurements and their uncertainties in different experiments. For example, the Detector and Stat/Meth categories can be considered largely uncorrelated between experiments, while the Model category is likely correlated.

### Combination results

**Fig. 36:**
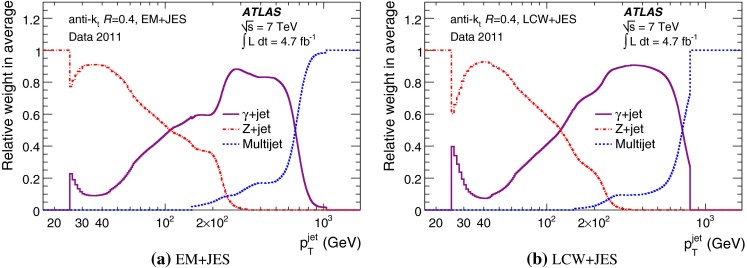
Weight carried by each in situ technique in the combination to derive the residual jet energy scale calibration as a function of the jet transverse momentum $p_{\mathrm {T}}^\mathrm {jet}$ for anti-$k_{t}$ jets with $R = 0.4$ calibrated with the **a** EM+JES and the **b** LCW+JES scheme. The $p_{\mathrm {T}}^\mathrm {jet}$ dependence of the weights is discussed in Sect. 13.4

Figure 36 shows the contribution of each in situ technique to the JES residual calibration, defined to be the fractional weight carried in the combination. In the region $p_{\mathrm {T}}^\mathrm {jet}\lesssim 100$ GeV, the $Z$-jet method has the highest contribution to the overall JES average. The contribution is 100 % for $p_{\mathrm {T}}^\mathrm {jet}$ below 25  GeV, the region covered only by $Z$-jet, about 90 % at $p_{\mathrm {T}}^\mathrm {jet}= 40$ GeV, and decreases to about 50 % at $p_{\mathrm {T}}^\mathrm {jet}= 100$ GeV. In order to prevent the uncertainties specific to the low-$p_{\mathrm {T}}^\mathrm {jet}$ region from propagating to higher $p_{\mathrm {T}}^\mathrm {jet}$ in the combination, the $Z$-jet measurements below and above $p_{\mathrm {T}}^\mathrm {jet}= 25$ GeV are treated separately, meaning no interpolation is performed across $p_{\mathrm {T}}^\mathrm {jet}= 25$  GeV, although the magnitude of the original systematic uncertainty sources is used, separately, in both regions.

The weaker correlations between the uncertainties of the $Z$-jet measurements, compared to ones from $\gamma \text {-jet}$, lead to a faster increase of the extrapolated uncertainties, hence to the reduction of the $Z$-jet weight in the region between 25 and 40$~\mathrm{GeV }{}$. In the region $100 \lesssim p_{\mathrm {T}}^\mathrm {jet}\lesssim 600$  GeV, the $\gamma \text {-jet}$ method dominates with a weight increasing from 50 % at $p_{\mathrm {T}}^\mathrm {jet}= 100$ GeV to about 80 % at $p_{\mathrm {T}}^\mathrm {jet}= 500$  GeV. For $p_{\mathrm {T}}^\mathrm {jet}\gtrsim 600$  GeV the measurement based on multijet balance becomes increasingly important and for $p_{\mathrm {T}}^\mathrm {jet}\gtrsim 800$ GeV it is the only method contributing to the JES residual calibration. The combination results and the relative uncertainties are considered in the $p_{\mathrm {T}}$ range from 17.5 GeV to 1 TeV, where sufficient statistics are available.

**Fig. 37:**
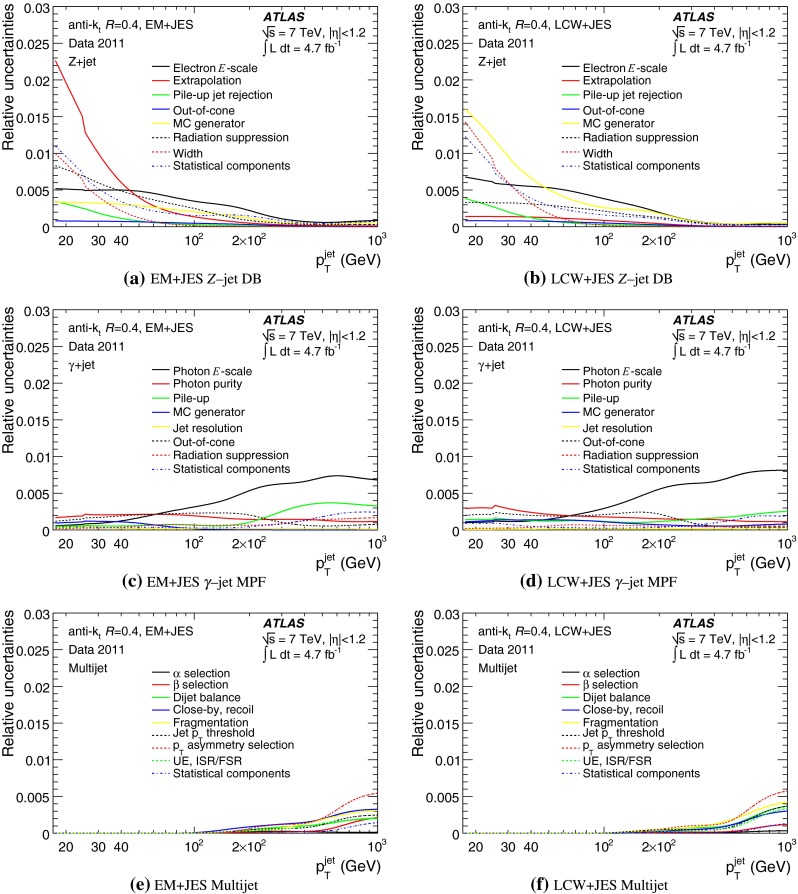
Individual uncertainty sources applicable to the combined response ratio as a function of the jet $p_{\mathrm {T}}$ for the three in situ techniques: **a**, **b** $Z$-jet direct balance, **c**, **d** $\gamma \text {-jet}$ MPF and **e**, **f** multijet balance for anti-$k_{t}$ jets with $R=0.4$ calibrated with the **a**, **c**, **e** EM+JES and the **b**, **d**, **f** LCW+JES scheme. The systematic uncertainties displayed here correspond to the components listed in Table 10

**Fig. 38:**
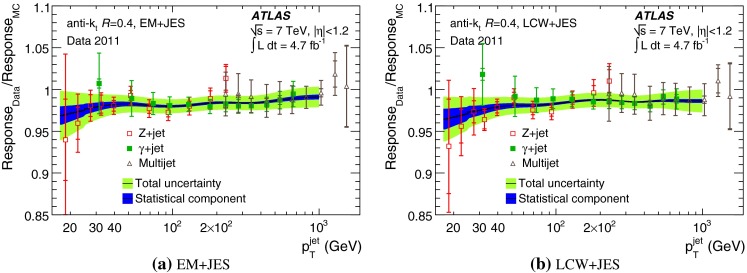
Ratio of the average jet response $\langle p_{\mathrm {T}}^\mathrm {jet}/p_{\mathrm {T}}^{\mathrm {ref}}\rangle$ measured in data to that measured in MC simulations for jets within $|\eta | < 1.2$ as a function of the transverse jet momentum $p_{\mathrm {T}}^\mathrm {jet}$. The data-to-MC jet response ratios are shown separately for the three in situ techniques used in the combined calibration: direct balance in $Z$-jet events, MPF in $\gamma \text {-jet}$ events, and multijet $p_{\mathrm {T}}$ balance in inclusive jet events. The *error bars* indicate the statistical and the total uncertainties (adding in quadrature statistical and systematic uncertainties). Results are shown for anti-$k_{t}$ jets with $R = 0.4$ calibrated with the **a** EM+JES and the **b** LCW+JES scheme. The *light band* indicates the total uncertainty from the combination of the in situ techniques. The *inner dark band* indicates the statistical component only

The individual uncertainty components for the final combination results,^15^ are shown in Fig. 37 for anti-$k_{t}$ jets with $R=0.4$ for the EM+JES and the LCW+JES calibration scheme and for each in situ technique.

The agreement between the in situ methods is good, with $\chi ^{2}/\mathrm{dof} < 1$ for most $p_{\mathrm {T}}$ bins, and values up to $\chi ^{2}/\mathrm{dof} = 1.5$ in only a few bins. The largest $\chi ^{2}/\mathrm{dof} = 2$ is found for anti-$k_{t}$ jets with $R=0.6$ calibrated with the LCW+JES scheme for $p_{\mathrm {T}}^\mathrm {jet}= 25$  GeV.

The final JES residual calibration obtained from the combination of the in situ techniques is shown in Fig. 38, together with statistical and systematic uncertainties. A general offset of about $-$2 % is observed in the data-to-MC response ratios for jet transverse momenta below 100  GeV. The offset decreases to about $-$1 % at higher $p_{\mathrm {T}}$ ($p_{\mathrm {T}}^\mathrm {jet}\gtrsim 200$). The JES uncertainty from the combination of the in situ techniques is about 2.5 % at $p_{\mathrm {T}}^\mathrm {jet}= 25$  GeV, and decreases to below 1 % for $55 \le p_{\mathrm {T}}^\mathrm {jet}< 500$  GeV. The multijet balance method is used up to 1  TeV, as at higher $p_{\mathrm {T}}$ values it has large statistical uncertainties. At 1 TeV the total uncertainty is about 1.5 %.

The results for the EM+JES and the LCW+JES calibration schemes for jets with $R=0.6$ are similar to those for $R=0.4$.

### Comparison of the ${\gamma }$-jet calibration methods

As discussed in Sect. 10, two different techniques exploiting the transverse momentum balance in $\gamma \text {-jet}$ events are used to probe the jet response, the direct balance (DB) and the missing momentum fraction (MPF) method. These methods have different sensitivities to parton radiation, pile-up interactions and photon background contamination, and hence different systematic uncertainties, as explored in Sect. 10.4.

**Fig. 39:**
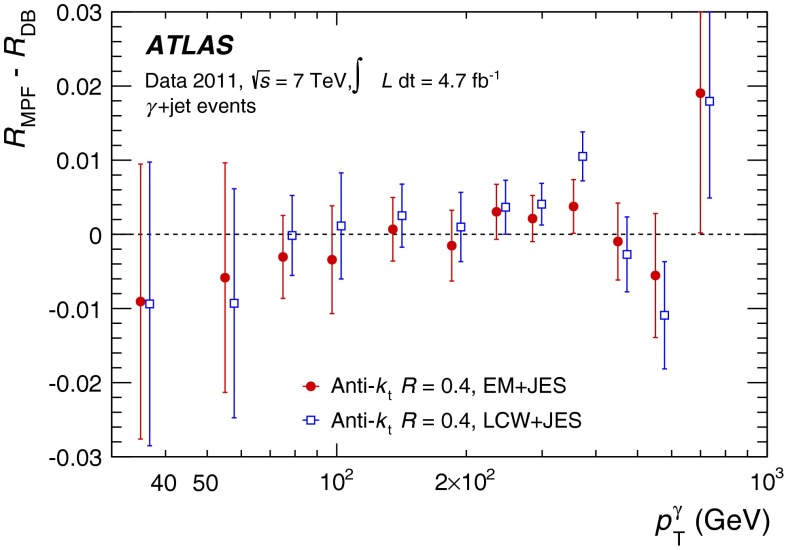
Difference between the data-to-MC response ratio $R$ measured using the direct balance (DB) and the missing momentum fraction (MPF) methods for jets reconstructed with the anti-$k_{t}$ algorithm with $R=0.4$ calibrated with the EM+JES and LCW+JES schemes. The *error bars* shown only contain the uncorrelated uncertainties

Since the MPF method uses the full hadronic recoil and not only the jet, a systematic uncertainty due to the possible difference in data and MC simulation of the calorimeter response to particles inside and outside of the jet needs to be taken into account. This systematic uncertainty contribution is estimated to be small compared to other considered uncertainties. However, in the absence of a more quantitative estimation, the full energy of all particles produced outside of the jet as estimated in the DB technique is taken as the systematic uncertainty. A comparison between the two results is shown in Fig. 39. The results are compatible within their uncorrelated uncertainties.

As the methods use similar datasets, the measurements are highly correlated and cannot easily be included together in the combination of the in situ techniques. In order to judge which method results in the most precise calibration, the combination described in Sect. 13.2 is performed twice, both for $Z$-jet, $\gamma \text {-jet}$ DB and multijet balance, and separately for $Z$-jet, $\gamma \text {-jet}$ MPF and multijet balance. The resulting combined calibration that includes the MPF method has slightly smaller uncertainties, by up to about 0.1 %, and is therefore used as the main result.

### Simplified description of the correlations

For some applications like parameterised likelihood fits it is preferable to have the JES uncertainties and correlations described by a reduced set of uncertainty components. This can be achieved by combining the least significant (weakest) nuisance parameters into one component while maintaining a sufficient accuracy for the JES uncertainty correlations.

The total covariance matrix $C^\mathrm{tot}$ of the JES correction factors can be derived from the individual components of the statistical and systematic uncertainties:

14 $$\begin{aligned} C^\mathrm{tot} = \sum _{k=1}^{N_\mathrm{sources}} C^{k}, \end{aligned}$$

where the sum goes over the covariance matrices of the individual uncertainty components $C^{k}$. Each uncertainty component $s^{k}$ is treated as fully correlated in $p_{\mathrm {T}}$ and the covariance of the $p_{\mathrm {T}}$ bins $i$ and $j$ is given by $C^{k}_{ij} = s^{k}_{i} s^{k}_{j}$. All the uncertainty components are treated as independent of one another, except for the photon and electron energy scales which are treated as correlated.^16^

A reduction of the number of nuisance parameters while retaining the information on the correlations can be achieved by deriving the total covariance matrix in Eq. (14) and diagonalising it:

$$\begin{aligned} C^\mathrm{tot} = S^{T} D~S. \end{aligned}$$

Here $D$ is a (positive definite) diagonal matrix, containing the eigenvalues $\sigma _{k}^{2}$ of the total covariance matrix, while the $S$ matrix contains on its columns the corresponding (orthogonal) unitary eigenvectors $V^k$. A new set of independent uncertainty sources can then be obtained by multiplying each eigenvector by the corresponding eigenvalue. The covariance matrix can be re-derived from these uncertainty sources using:

$$\begin{aligned} C^\mathrm{tot}_{ij} = \sum _{k=1}^{N_\mathrm{bins}} \sigma _{k}^{2}~V^k_i~V^k_j, \end{aligned}$$

where $N_\mathrm{bins}$ is the number of bins used in the combination.

A good approximation of the covariance matrix can be obtained by separating out only a small subset of $N_\mathrm{eff}$ eigenvectors that have the largest corresponding eigenvalues. From the remaining $N_\mathrm{bins}-N_\mathrm{eff}$ components, a residual, left-over uncertainty source is determined, with an associated covariance matrix $C'$. The initial covariance matrix can now be approximated as:

$$\begin{aligned} C^\mathrm{tot}_{ij} \approx \sum _{k=1}^{N_\mathrm{eff}} \sigma _{k}^{2}~V^k_i~V^k_j + C'. \end{aligned}$$

This approximation conserves the total uncertainty, while the precision on the description of the correlations can be directly determined by comparing the original full correlation matrix and the approximate one. The last residual uncertainty could in principle be treated either as correlated or as uncorrelated between the $p_{\mathrm {T}}$ bins. It is observed that treating this uncertainty source as uncorrelated in $p_{\mathrm {T}}$ provides a better approximation of the correlation matrix. This is expected, as this residual uncertainty source includes many orthogonal eigenvectors with small amplitudes and many oscillations, hence the small correlations. The original exact covariance matrix is thus decomposed into a part with strong correlations and another one with much smaller correlations. It is this residual uncertainty source that incorporates the part with small correlations.

**Fig. 40:**
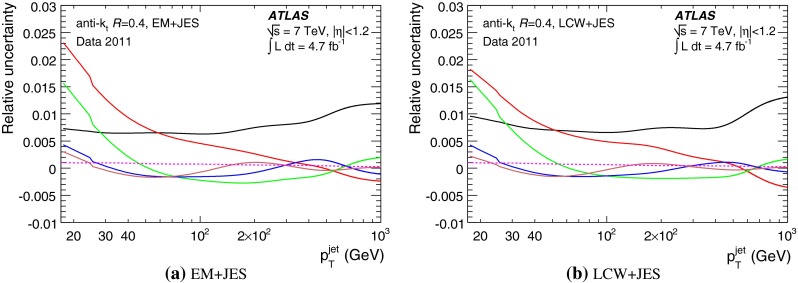
Systematic (effective) relative uncertainties displayed as a function of jet $p_{\mathrm {T}}$ for anti-$k_{t}$ jets with $R=0.4$ calibrated with the **a** EM+JES and the **b** LCW+JES calibration schemes for the reduced scheme with six nuisance parameters. Each *curve* can be interpreted as a $1\sigma$ JES systematic nuisance parameter, symmetric around zero. They represent eigenvectors of the covariance matrix (*continuous lines*) and the residual component (*dashed line*)

Figure 40 shows the obtained five eigenvectors $\sigma _kV^k$ and the residual sixth component, as a function of the jet $p_{\mathrm {T}}$. The $p_{\mathrm {T}}$-dependent sign of these eigenvectors allows to keep track of the (anti-)correlations of each component in different phase-space regions. This is necessary for a good description of the correlations of the total JES uncertainty. These six nuisance parameters are enough to describe the correlation matrix with sufficient precision at the level of percent. As explained above, the quadratic sum of these six components is identical to the quadratic sum of the original uncertainties shown in Fig. 37. In the high-$p_{\mathrm {T}}$ region above 300 GeV, one eigenvector has a significantly larger amplitude than all the others, see the black curve in Fig. 40, hence the strong correlations between the bins. Approximately 60–80 % of this component is due to the photon and electron energy scale uncertainties up to about 700  GeV (see Fig. 37c, d), while some other uncertainties contribute to it at higher $p_{\mathrm {T}}$.

**Fig. 41:**
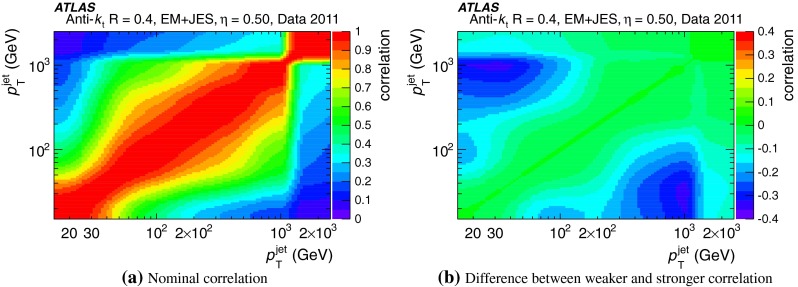
In **a**, the nominal JES correlation matrix is shown. The difference between the correlation matrices of the interpretations resulting in stronger and weaker correlations for anti-$k_{t}$ jets with $R=0.4$ calibrated using the EM+JES calibration scheme in the central calorimeter region ($\eta =0.5$) is depicted in **b**

**Fig. 42:**
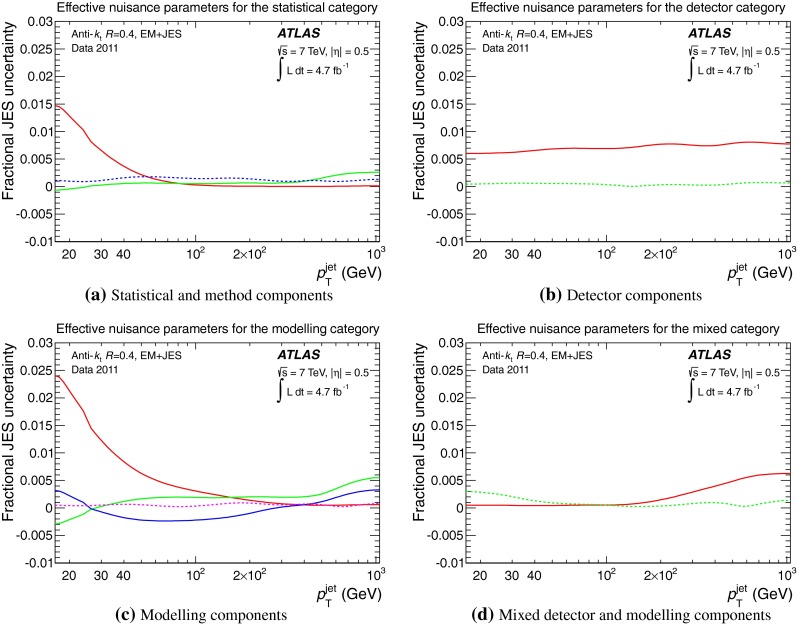
Relative uncertainties for reduced (effective) components within a single category displayed as a fraction of jet $p_{\mathrm {T}}$ for anti-$k_{t}$ jets with $R=0.4$, calibrated with the EM+JES scheme. The convention from Fig. 40 is followed here. The 54 nuisance parameters that are input to the reduction for each of the categories are listed in Table 10. The reduction is performed for all nuisance parameters belonging to any given category, which are statistical and method components (**a**), detector components (**b**), modelling components (**c**), and mixed detector and modelling components (**d**). Each of the *curves* can be interpreted as an effective $1\sigma$ JES systematic nuisance parameter, symmetric around zero. They represent eigenvectors of the covariance matrix (*continuous lines*) and the residual component (*dashed line*), for the specified category

### Jet energy scale correlation scenarios

The JES uncertainty and its correlations discussed so far can play a crucial role in physics analyses. In order to quantify these correlations, knowledge of the interdependence of the systematic uncertainty sources is needed. The limitations in this knowledge lead to uncertainties on the correlations.

The variation of the systematic uncertainty sources as a function of $p_{\mathrm {T}}$ and $\eta$ can be described as a nuisance parameter, as explained before. The total set of correlations can be expressed in the form of a correlation matrix calculated from the full set of nuisance parameters as presented in Sect. 13.4. The correlation matrix, derived assuming that the nuisance parameters are independent from each other, is shown in Fig. 41a.

The nuisance parameters are affected by the strength of the correlations between uncertainty components, which can be difficult to estimate. The investigation of alternative correlation scenarios for the components thus allows to determine the uncertainty on the global correlations shown in Fig. 41a.

Two additional configurations are specifically designed to weaken and to strengthen the global correlations. They cover the space of reasonable JES component dependencies. In a given physics analysis these scenarios can be used to examine how the final results are affected by variations of the correlation strengths. This allows propagation of the uncertainties on the correlations. The difference between the weaker and stronger correlation matrices is shown in Fig. 41b.

### Alternative reduced configurations

A global reduction of nuisance parameters, irrespective of the uncertainty source, is performed in order to reduce the number of these parameters required to represent the full correlation matrix, see Sect. 13.6. However, it is also useful to keep track of the physical meaning of the uncertainty components, e.g. for a proper combination of measurements from different experiments. In Sect. 13.3 each JES systematic uncertainty component is assigned to a representative category, as given in Table 10.

The same reduction technique discussed in Sect. 13.6 is applied independently to each set of uncertainty components within each individual category. The resulting reduced set of uncertainty components for the nominal configuration are shown in Fig. 42. This category reduction approach generally results in a larger number of nuisance parameters than the global reduction. This is because two components from different categories with very similar shapes can be globally combined without significant loss of information for the correlations. However, when the reduction is performed in categories, components may require a nuisance parameter not lose significant precision for the description of the global correlation.

This technique is applied to each of the correlation scenarios. Category reduction configurations are derived for the set of all parameters, the stronger correlation scenario, and the weaker correlation scenario. In each case, correlation matrices are compared to ensure that the reduction preserved correlation information to within a few percent. Table 11 lists the various configurations evaluated, together with the accuracy achieved with the reduction procedure.

**Table 11:** A summary of the various explored JES configurations. The precision of the reduction is defined by the largest deviation in correlations between the original full set of parameters and the reduced version. The full $p_{\mathrm {T}}$ phase space is considered in this determination. The values quoted are for jets in the region $|\eta |<1.2$ for anti-$k_{t}$ with $R=0.4$ calibrated with EM+JES scheme. The number of nuisance parameters quoted refers only to the parameters entering the reduction procedure, which are relevant to the in situ techniques

Configuration type	Reduction	$N_\mathrm{params}$	Reduction precision (%)
All parameters	None	54	100
	Global	6	97
	Category	11	95
Stronger correlations	None	45	100
	Global	6	97
	Category	12	96
Weaker correlations	None	56	100
	Global	6	97
	Category	12	95

## Comparison to jet energy scale uncertainty from single-hadron response measurements

The JES correction and uncertainty derived from in situ techniques exploiting the $p_{\mathrm {T}}$ balance between a jet and a reference object can be compared to the method where the jet energy scale is estimated from single-hadron response measurements, as described in Ref. [3]. In this method, jets are treated as a superposition of energy deposits of single particles. For each calorimeter energy deposition within the jet cone, the type of the particle inside the jet is determined, and the expected mean shift and the systematic uncertainty of the calorimeter response between data and MC simulation is evaluated. The corresponding uncertainty is derived from in situ measurements or systematic MC variations. This deconvolution method is described in Refs. [3, 4] and is used for the derivation of the JES uncertainty for the ATLAS 2010 data analysis.

Measurements of the calorimeter response to pions in the combined test-beam [89] are used for pions with momenta between 20 and 350  GeV.^17^ Single isolated hadrons with momenta up to 20  GeV are selected in a minimum bias sample produced in proton–proton collisions at $\sqrt{s} = 7$ TeV taken in 2011 and the calorimeter energy ($E$) in a narrow cone around an isolated track is compared to the track momentum ($p$) (see Refs. [4, 90] for more details). Effects from the noise thresholds and from the calorimeter acceptance are estimated by comparing the energy measured in calorimeter cells to the one measured in topo-clusters. In addition, the uncertainty on the absolute electromagnetic energy scale is considered and the response uncertainty of protons, anti-protons and neutral hadrons is evaluated using different hadronic shower models, again as described in Refs. [4, 90]. For hadrons with $p > 400$  GeV, for which no measurements are available in the combined test-beam, the uncertainty is conservatively estimated as 10 % to account for possible calorimeter non-linearities or longitudinal leakage.

The mean $E/p$ is well described by the MC simulation for $p>6$  GeV. However, for lower momenta ($1 \lesssim p < 6$  GeV) the data are shifted down with respect to the MC simulation by about 4 %. This is in contrast to the 2010 measurement, where an agreement within 3 % is found [4]. The worse data-to-MC agreement is due to the new corrections in the absolute electromagnetic energy scale obtained in situ using the $Z$ boson mass constraint reconstructed from $Z \rightarrow e^+ e^-$, the increased topo-cluster thresholds, and the use of a new Geant4 version.

**Fig. 43:**
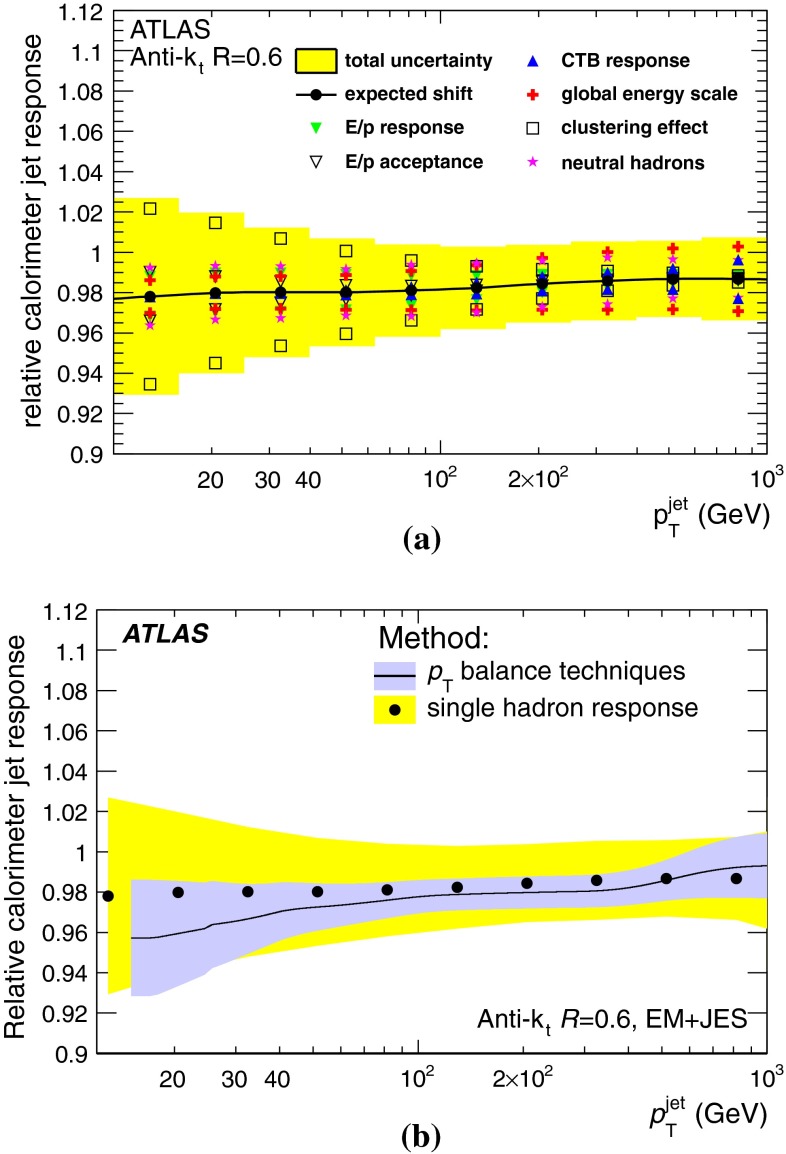
Relative calorimeter jet response ratio between data and MC simulations, as estimated from the single-hadron response measurements as a function of the jet transverse momentum, is shown in **a**. The total systematic uncertainty together with the uncertainty from the individual components is shown as a *lighter band*. The *black circles* denote the estimated mean shift of the calorimeter response to jets in data over the one in MC simulations. In **b**, the uncertainty from the single-hadron response measurements is shown as a *lighter* (*yellow*) *band*, while the JES uncertainty, as derived from the in situ methods based on $p_{\mathrm {T}}$ balance, is shown as a *dark* (*gray*) *band*. The *closed markers* denote the estimated shift of the calorimeter response to jets in data over the one in MC simulations, and the *line* shows the JES correction derived from the $p_{\mathrm {T}}$ balance in situ methods

Figure 43a shows the estimated calorimeter jet response ratio between data and MC simulation as estimated from the single-hadron response measurements as a function of the jet transverse momentum. A lower calorimeter response to jets in data than in the MC simulations is observed (black circles), consistent with that obtained using in situ techniques. The uncertainty on this ratio is about 4 % at very low and very high $p_{\mathrm {T}}$. It decreases to about 2 % between $100 \le p_{\mathrm {T}}< 600$  GeV. The individual uncertainty components are also shown. The dominant uncertainties at low $p_{\mathrm {T}}$ are those from noise threshold effects, which can be different for single isolated hadrons and hadrons inside jets. At high $p_{\mathrm {T}}$ the response differences between data and MC simulation as measured in the ATLAS combined test-beam and the uncertainty for hadrons with $p>400$  GeV are largest. The uncertainty on the global electromagnetic energy scale and the response uncertainty for neutral hadrons contribute about 1 %.

Figure 43b compares the JES uncertainty as obtained from single hadron response measurements to the one obtained from the in situ method based on the $p_{\mathrm {T}}$ balance between a jet and a well-measured reference object. For both methods the mean jet calorimeter response in data is observed to be shifted down by about 2 % with respect to the one in the MC-simulated events. However, the $p_{\mathrm {T}}$ balance methods give a considerably smaller uncertainty.

## Jet energy scale uncertainty from the W boson mass constraint

The mass of the $W$ boson ($m_\mathrm{W}$) provides a stable reference for the determination jet energy scale uncertainty. In events where a top pair ($t\bar{t}$) is produced, the hadronically decaying $W$ bosons give rise to two jets that can be well identified. A dedicated event reconstruction is developed in order to find the jets from the $W$ decay. The jet energy measurement can be assessed by measuring the residual difference between the observed and the simulated invariant $W$ mass spectrum.

$W$ provide a pure source of jets induced by quarks. A sizeable fraction of these jets are induced by charm quarks and contain charm hadrons. Given that an unbiased sample of charm jets can not be selected in data, all jets from $W$ decays are treated in the same way.

### Event samples

The dataset is selected using single-electron or single-muon triggers. Jets are reconstructed with the anti-$k_{t}$ algorithm with $R = 0.4$ starting from topo-clusters and are calibrated with the EM+JES scheme. Jets from the decay of heavy-flavour hadrons are selected by the so-called MV1 algorithm, a neural-network-based $b$-tagging algorithm described in Ref. [91]. It is used at an operating point with 70 % efficiency for *b*-jets, and a mistag rate of less than 1 %, as determined from simulated $t\bar{t}$ events.

Events with leptonically decaying $W$ bosons are selected as follows:

Candidate electrons with transverse momenta $p_{\mathrm {T}}> 25$ GeV are required to pass the tight ATLAS electron quality cuts [72]. Muons with transverse momentum $p_{\mathrm {T}}> 20$  GeV are required to pass ATLAS standard muon quality cuts [92]. Events with an electron (muon) are required to be triggered by an electron (muon) trigger with a threshold of 20 (18)  GeV, thus ensuring the trigger is fully efficient.

Events are required to have a missing transverse momentum ${E}_{\mathrm {T}}^{\mathrm {miss}}> 30$  GeV (${E}_{\mathrm {T}}^{\mathrm {miss}}> 20$  GeV) in the electron (muon) channel. The signal region for this analysis requires exactly one charged lepton and four or more jets. Two $b$-tagged jets are required in each event. ${E}_{\mathrm {T}}^{\mathrm {miss}}$ is calculated from the vector sum of the energy in the calorimeter cells associated to topo-clusters[93]. Additionally, the transverse mass of the reconstructed leptonic $W$ boson is required to pass $m_{\mathrm {T}}^{W} > 30$ GeV in the electron channel, or ${E}_{\mathrm {T}}^{\mathrm {miss}}+ m_{\mathrm {T}}^{W} > 60$ GeV in the muon channel. Here $m_{\mathrm {T}}^{W}$ is defined as:

$$\begin{aligned} m_{\mathrm {T}}^{W} = \sqrt{2 p_{\mathrm {T}}^{\ell } {E}_{\mathrm {T}}^{\mathrm {miss}}\left( 1 - \cos \left( \Delta \Phi \left( \ell ,E_{\mathrm {T}}^{\mathrm {miss}}\right) \right) \right) } , \end{aligned}$$

with the lepton transverse momentum $p_{\mathrm {T}}^{\ell }$ and the azimuthal angle $\Delta \Phi$ between the lepton and the missing transverse energy.

A cut is applied on each event to have fewer than seven reconstructed jets, to significantly reduce the number of possible jet pair combinations per event. The main background processes to $t\bar{t}$ are single-top production, multijet and $W$ boson production in association with jets. The $t\bar{t}$ signal purity is greater than 90 % after this selection.

### Reconstruction of the W boson

The reconstruction efficiency for hadronically decaying $W$ bosons is measured by the fraction of reconstructed jet pairs matching the same $W$ boson. This can be done by forming all possible light-quark jet pairs consisting of jets which are not $b$-tagged, and calculating their invariant mass $m_{\mathrm {jj}}$. Then, only pairs with $|m_{\text {jj}} - m_{W}^{\mathrm {MC}}| < 4 \sigma _{W}$ are considered as originating from $W$ boson decays. Here $m_{W}^{\mathrm {MC}}$ is the $W$ mass and $\sigma _{W}$ is the expected $m_\mathrm{W}$ resolution, both taken from MC-simulation samples. This relatively large window of about 11 GeV avoids biases in the reconstructed $W$ mass peak, and only about 3 % of true $W$ bosons are rejected by this mass cut

Two methods are used to select one jet pair per event. The first method is based on topological proximity in the detector, where the jet pair which minimises the distance between the two jets $\Delta R_\mathrm{jj}$, calculated in ($\eta ,\phi$) space as defined in Eq. (3) in Sect. 5.6, is selected. This reconstruction has an efficiency of 51 % in finding the signal jet pair at the level of the selection for reconstructible events. The second jet selection method is based on transverse momentum maximisation such that the two light-quark jets maximising the $p_{\mathrm {T}}$ of the reconstructed $W$ are taken as the two jets from the hadronic decay. This reconstruction has an efficiency of 55 %.

Jet pairs with $\Delta R_\mathrm{jj}< 0.7$ are rejected in order to avoid geometrically overlapping jets and to reduce the sensitivity to parton radiation in the $W$ mass spectrum.

In order not to be sensitive to the jet mass the reconstructed $W$ mass $m_{W}^{\text {rec}}$ is calculated as:

$$\begin{aligned} m_{W}^{\text {rec}}= \sqrt{2 E_{1}E_{2} \left( 1 - \cos {\theta _{1,2}} \right) } , \end{aligned}$$

where $E_1$, $E_2$ are the respective energies of the paired jets, and $\theta _{1,2}$ is the opening angle between them.

### Extraction of the relative light jet scale

The relative light-quark jet calibration ${\alpha _l}$ is defined by

$$\begin{aligned} {\alpha _l}= \frac{\alpha _{l}^\mathrm{data}}{\alpha _{l}^\mathrm{MC}}, \end{aligned}$$

where $\alpha _{l}^\mathrm{data}$ ($\alpha _{l}^\mathrm{MC}$) is the jet energy scale in the data (simulation). This analysis uses the expected dependency of the $W$ mass distribution on the ${\alpha _l}$ parameter. Templates for the $m_\mathrm{W}$ distributions are derived from MC simulations, where $\alpha _{l}^\mathrm{MC}$ is varied. This rescaling of $\alpha _{l}^\mathrm{MC}$ is applied before the event selection and the $W$ reconstruction steps. A set of $m_\mathrm{W}$ distributions are produced for different ${\alpha _l}$ values. In order to obtain the $m_\mathrm{W}$ distribution of an arbitrary ${\alpha _l}$ value, a bin-by-bin interpolation is performed using the two generated and adjacent ${\alpha _l}$ values.

A binned likelihood maximisation with a Poisson law is used. It identifies the ${\alpha _l}$ values whose associated $m_\mathrm{W}$ distribution fits the best to the observed $m_\mathrm{W}$ distribution. The analysis templates are defined for ${\alpha _l}$ values ranging from ${\alpha _l}$= 0.85 to ${\alpha _l}$ $=$ 1.15.

In order to test the consistency of the extraction method, an arbitrary jet energy scale is applied to one pseudo-experiment of arbitrary luminosity. The comparison is then done between the applied scale and the measured one. The difference between both is compatible with zero for a wide range of ${\alpha _l}$ hypotheses.

**Table 12:** Systematic uncertainties on the ${\alpha _l}$ measurement. Uncertainties lower than 0.05 % are not listed. The two different jet selection strategies for the $W$ boson reconstruction discussed in the text are topological proximity (“topo. prox.”) and $p_{\mathrm {T}}$-maximisation (“$p_{\mathrm {T}}$-max.”)

Effects	$\Delta$ ${\alpha _l}$ topo. prox. (%)	$\Delta$ ${\alpha _l}$ $p_{\mathrm {T}}$-max. (%)
Multijet background	$\pm 0.12$	$\pm 0.18$
Jet resolution	$\pm 0.39$	$\pm 0.80$
MC generator	$\pm 0.41$	$\pm 0.25$
Fragmentation	$\pm 0.65$	$\pm 0.68$
Parton radiation	$\pm 2.48$	$\pm 2.42$
Total	$\pm 2.63$	$\pm 2.65$

The expected statistical precision on ${\alpha _l}$ is determined using pseudo-experiments each one containing a number of events corresponding to the luminosity recorded in 2011. A pull variable is computed, reflecting the differences between the measured and the expected mean values scaled with the observed uncertainties. The mean pull is compatible with zero and its standard deviation with unity. The mean value of the uncertainties obtained from the different pseudo-experiments is taken as the expected statistical precision. It is 0.28 % for the maximum $p_{\mathrm {T}}$ reconstruction method and 0.29 % for the topological proximity reconstruction method.

**Fig. 44:**
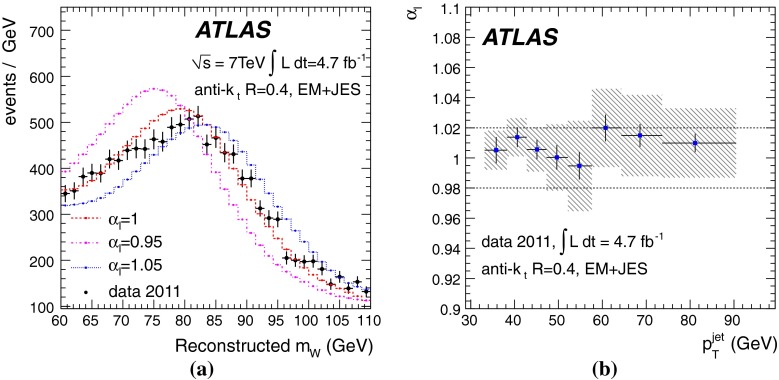
The three templates distributions for the reconstructed $W$ mass in $t\bar{t}$ events obtained by shifting the jet energy by a factor ${\alpha _l}= 0.95$, 1 and 1.05 in the Monte Carlo simulation with respect to the one in data (**a**). The Monte Carlo simulation templates are also compared to the data distribution. The ${\alpha _l}$ measurement as a function of mean jet $p_{\mathrm {T}}$ using the maximum $p_{\mathrm {T}}$ reconstruction approach, is shown in **b**. *Error bars* are statistical while *hashed rectangles* represent the total uncertainties

### Systematic uncertainties

The main sources of systematic uncertainties on the ${\alpha _l}$ measurement are summarised in Table 12 and presented for the topological proximity and the $p_{\mathrm {T}}$ maximisation reconstruction methods.

A variety of potential systematic effects are evaluated. The uncertainty from the shape of the multijet background, the uncertainty on the jet energy resolution, the jet reconstruction efficiency and the $b$-tagging efficiency and mistag rate. The uncertainties on the Monte Carlo simulation model are estimated in terms of generator variations, fragmentation uncertainty and parton radiation variation rate. In particular the parton radiation rate can alter the $t\bar{t}$ final states, inducing distortions in the reconstructed $m_\mathrm{W}$ distribution.

**Table 13:** The measurement of ${\alpha _l}$ using the closest proximity ($\Delta R_{\mathrm {jj}}^{\mathrm {min}}$) and the maximum $p_{\mathrm {T}}$ ($p_{\mathrm {T}}^{\mathrm {max}}$) approach, respectively, for the electron channel, the muon channel and both together. Uncertainties are statistical only

${\alpha _l}$	$e$ channel	$\mu$ channel	e + $\mu$ channels
$\Delta R_{\mathrm {jj}}^{\mathrm {min}}$	$1.0130 \pm 0.0048$	$1.0143 \pm 0.0038$	$1.0137 \pm 0.0031$
$p_{\mathrm {T}}^{\mathrm {max}}$	$1.0105 \pm 0.0045$	$1.0141 \pm 0.0038$	$1.0130 \pm 0.0028$

### Results

Figure 44a shows the observed $m_\mathrm{W}$ distribution from the maximum $p_{\mathrm {T}}$ reconstruction compared to three different templates. The relative scale correction ${\alpha _l}$ is extracted for electron and muon channels together as well as for the two channels separately. Results are summarised in Table 13.

In order to test the stability of the measurement, cross-checks are performed by relaxing the $\Delta R_\mathrm{jj}$ cut and by changing the $m_\mathrm{W}$ reconstruction definition. None of these changes affects the measured ${\alpha _l}$ by more than 0.15 %. Since the definition of $m_\mathrm{W}$ depends on $\Delta R_\mathrm{jj}$, a cross-check is done by an event re-weighting in MC simulation in order to reproduce the observed $\Delta R_\mathrm{jj}$ distribution in data. The effect on ${\alpha _l}$ is about 0.12 % for the two reconstruction methods.

The relative scale ${\alpha _l}$ is studied as a function of the mean $p_{\mathrm {T}}$, see Fig. 44, as well as a function of $\eta$ of the two jets coming from the $W$ boson decay. The tested $p_{\mathrm {T}}$ values range from 33 to 90 GeV. Templates of the $m_\mathrm{W}$ are produced for each bin of $p_{\mathrm {T}}$ or $\eta$. Taking into account systematical uncertainties, no significant dependence is observed with respect to the average $p_{\mathrm {T}}$ or $\eta$ of the two jets. The mean ${\alpha _l}$ is measured as ${\alpha _l}$ $= 1.0130 \pm 0.0028 \pm 0.027$.

**Fig. 45:**
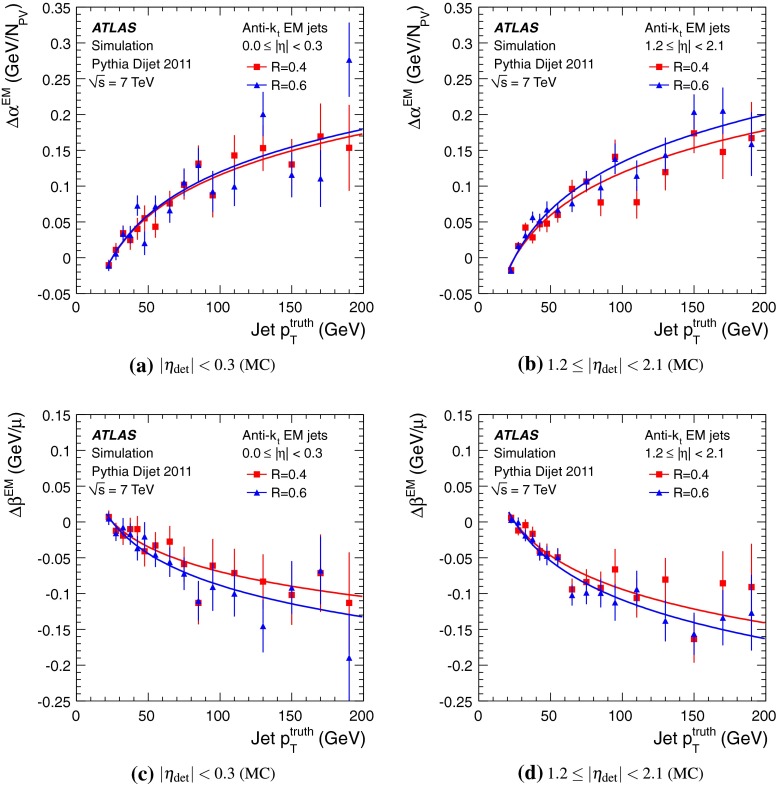
The difference from the average $\alpha ^{\mathrm{EM}}(\eta _\mathrm{det})$ of the in-time pile-up signal contribution per reconstructed primary vertex ($\Delta (\partial p_{\mathrm {T,\mathrm{EM}}}^{\mathrm {jet}}/\partial N_{\mathrm{PV}})(p_{\mathrm {T}}^\mathrm {truth})$) as a function of the true jet transverse momentum $p_{\mathrm {T}}^\mathrm {truth}$, for MC-simulated jets reconstructed with anti-$k_{t}$ $R = 0.4$ and $R = 0.6$ at the EM scale, in two different regions **a** $|\eta _\mathrm{det}| < 0.3$ and **b** $1.2 \le |\eta _\mathrm{det}| < 2.1$ of the ATLAS calorimeter. In **c** and **d**, the variations of the out-of-time pile-up signal contribution per interaction with $p_{\mathrm {T}}^\mathrm {truth}$ around its average $\beta ^{\mathrm{EM}}(\eta _\mathrm{det})$ ($\Delta (\partial p_{\mathrm {T,\mathrm{EM}}}^{\mathrm {jet}}/\partial \mu {})(p_{\mathrm {T}}^\mathrm {truth})$) are shown for the same jet samples and the same respective $\eta _\mathrm{det}$ regions. Logarithmic functions of $p_{\mathrm {T}}^\mathrm {truth}$ are fitted to the points obtained from MC simulations

The agreement between the jet energy scale in data and Monte Carlo simulation is found to be in agreement within the estimated uncertainties. The main systematic uncertainty is related to the modelling of additional parton radiation (see Table 12).

## Systematic uncertainties on corrections for pile-up interactions

### Event and object selection

The pile-up corrections for jets derived from MC simulation, as described in Sect. 6, can be validated with data samples of collisions events where a stable reference that is insensitive to pile-up can be used to assess the agreement of the Monte Carlo simulation with data. Of particular interest here are $\gamma \text {-jet}$ events in prompt photon production, as the reconstructed photon kinematics are not affected by pile-up, and its transverse momentum $p_{\mathrm {T}}^{\mathrm {\gamma }}$ provides the stable reference for the pile-up dependent response of the balancing jet in the ratio $p_{\mathrm {T}}^\mathrm {jet}/p_{\mathrm {T}}^{\mathrm {ref}}= p_{\mathrm {T}}^\mathrm {jet}/p_{\mathrm {T}}^{\mathrm {\gamma }}$. The $\gamma \text {-jet}$ sample is selected as detailed in Sect. 10.2.

Another per jet kinematic reference is provided by the track jets from the primary collision vertex introduced in Sect. 5.4. These are matched with calorimeter jets, and the transverse momentum ratio $p_{\mathrm {T}}^\mathrm {jet}/p_{\mathrm {T}}^{\mathrm {ref}}= p_{\mathrm {T}}^\mathrm {jet}/p_{\mathrm {T}}^{\mathrm {track\ jet}}$ is evaluated. The jet event sample needed for this evaluation can be extracted from samples with central jets in the final state. Both this and the $\gamma \text {-jet}$ data samples are mostly useful for validation of the pile-up correction methods, as the limited statistics and phase space coverage in 2011 data do not allow direct determination of the pile-up corrections from these final states in data.

To evaluate the pile-up corrections based on track jets, events with a calorimeter jet matching a $\mathrm {track\ jet}$ with $p_{\mathrm {T}}^{\mathrm {track}}> 20$  GeV are extracted from an event sample triggered by high-$p_{\mathrm {T}}$ muons, thus avoiding potential jet-trigger biases. A $\mathrm {track\ jet}$ is only associated with a calorimeter jet not overlapping with any reconstructed muon with $p_{\mathrm {T}}^{\mu } > 5$ GeV, to avoid potential biases from heavy-flavour jets containing semi-leptonic decays. The general matching criterion for $\mathrm {track\ jet}$s to calorimeter jets is based on the distance between the two jets $\Delta R$ in ($\eta ,\phi$) space, as defined in Eq. (3) in Sect. 5.6. Only uniquely matched track-jet–calorimeter-jet pairs with distances $\Delta R< 0.3$ are considered. Outside of the imposed requirement for calorimeter jet reconstruction in ATLAS in 2011 ($p_{\mathrm {T}}^\mathrm {jet}> 10$ GeV), no further cuts are applied on $p_{\mathrm {T}}^\mathrm {jet}$, to avoid biases in the $p_{\mathrm {T}}^\mathrm {jet}/p_{\mathrm {T}}^{\mathrm {track}}$ ratio, in particular at low $p_{\mathrm {T}}^{\mathrm {track}}$.

### Derivation of the systematic uncertainty

The systematic uncertainties introduced by applying the MC-simulation-based pile-up correction to the reconstructed $p_{\mathrm {T,\mathrm{EM}}}^{\mathrm {jet}}$ and $p_{\mathrm {T,\mathrm{LCW}}}^{\mathrm {jet}}$ for jets in collision data include the variation of the slopes $\alpha = \partial p_{\mathrm {T}}/\partial N_{\mathrm{PV}}$ and $\beta = \partial p_{\mathrm {T}}/\partial \mu$ with changing jet $p_{\mathrm {T}}$. While the immediate expectation from the stochastic and diffuse nature of the (transverse) energy flow in pile-up events is that all slopes in $N_{\mathrm{PV}}$ ($\alpha ^{\mathrm{EM}}$, $\alpha ^{\mathrm{LCW}}$) and $\mu$ ($\beta ^{\mathrm{EM}}$, $\beta ^{\mathrm{LCW}}$) are independent of this jet $p_{\mathrm {T}}$, Fig. 45 clearly shows a $p_{\mathrm {T}}^\mathrm {truth}$ dependence of the signal contributions from in-time and out-of-time pile-up for jets reconstructed on EM scale. A similar $p_{\mathrm {T}}^\mathrm {truth}$ dependence can be observed for jets reconstructed on LCW scale.

The fact that the variations $\Delta (\partial p_{\mathrm {T}}/\partial N_{\mathrm{PV}})$ with $p_{\mathrm {T}}^\mathrm {truth}$ are very similar for narrow ($R = 0.4$) and wide ($R = 0.6$) anti-$k_{t}$ jets indicates that this $p_{\mathrm {T}}$ dependence is associated with the signal core of the jet. The presence of dense signals from the jet increases the likelihood that small pile-up signals survive the noise suppression applied in the topological clustering algorithm, see Sect. 5.1. As the core signal density of jets increases with $p_{\mathrm {T}}$, the acceptance for small pile-up signals thus increases as well. Consequently, the pile-up signal contribution to the jet increases. This jet $p_{\mathrm {T}}$ dependence is expected to approach a plateau as the cluster occupancy in the core of the jet approaches saturation, which means that all calorimeter cells in the jet core survive the selection imposed by the noise thresholds in the topo-cluster formation, and therefore all pile-up scattered into these same cells contributes to the reconstructed jet $p_{\mathrm {T}}$. The jet $p_{\mathrm {T}}$ dependent pile-up contribution is not explicitly corrected for, and thus is implicitly included in the systematic uncertainty discussed below.

Since the pile-up correction is derived from MC simulations, it explicitly does not correct for systematic shifts due to mis-modelling of the effects of pile-up on simulated jets. The sizes of these shifts may be estimated from the differences between the offsets obtained from data and from MC simulations:

$$\begin{aligned}&\Delta \mathcal {O}^{\mathrm{EM}} = \left. \mathcal {O}^{\mathrm{EM}}(N_{\mathrm{PV}},\mu )\right| _{\mathrm {data}} - \left. \mathcal {O}^{\mathrm{EM}}(N_{\mathrm{PV}},\mu )\right| _{\mathrm {MC}} \\&\Delta \mathcal {O}^{\mathrm{LCW}} = \left. \mathcal {O}^{\mathrm{LCW}}(N_{\mathrm{PV}},\mu )\right| _{\mathrm {data}} - \left. \mathcal {O}^{\mathrm{LCW}}(N_{\mathrm{PV}},\mu )\right| _{\mathrm {MC}} \end{aligned}$$

To assign uncertainties that can cover these shifts, and to incorporate the results from each in situ method, combined uncertainties are calculated as a weighted RMS of $\Delta \mathcal {O}(N_{\mathrm{PV}},\mu )$ from the offset measurements based on $\gamma \text {-jet}$ and on track jets. The weight of each contribution is the inverse squared uncertainty of the corresponding $\Delta \mathcal {O}(N_{\mathrm{PV}},\mu )$. This yields absolute uncertainties in $\alpha$ and $\beta$, which are then translated to fractional systematic shifts in the fully calibrated and corrected jet $p_{\mathrm {T}}$ that depend on the pile-up environment, as described by $N_{\mathrm{PV}}$ and $\mu$.

**Fig. 46:**
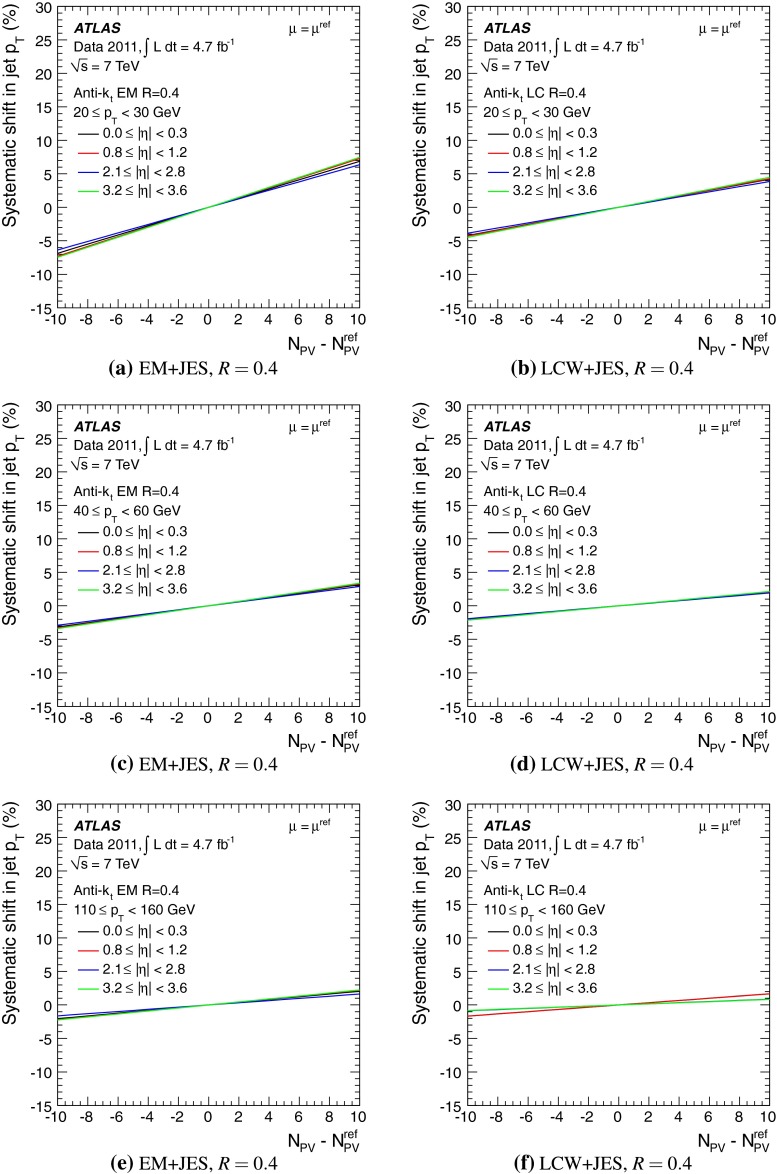
The fractional systematic shift due to mis-modelling of the effect of in-time pile-up on the transverse momentum $p_{\mathrm {T,\mathrm{EM+JES}}}^{\mathrm {jet}}$ of jets reconstructed with the anti-$k_{t}$ algorithm with $R = 0.4$, and calibrated with the EM+JES scheme, is shown as a function of $(N_{\mathrm{PV}}-N_{\mathrm{PV}}^\mathrm{ref})$ in **a**, **c**, and **e** for various $p_{\mathrm {T,\mathrm{EM+JES}}}^{\mathrm {jet}}$ bins. The same systematic shift is shown in **b**, **d**, and **f** for jets calibrated with the LCW+JES scheme, now in bins of $p_{\mathrm {T,\mathrm{LCW+JES}}}^{\mathrm {jet}}$

**Fig. 47:**
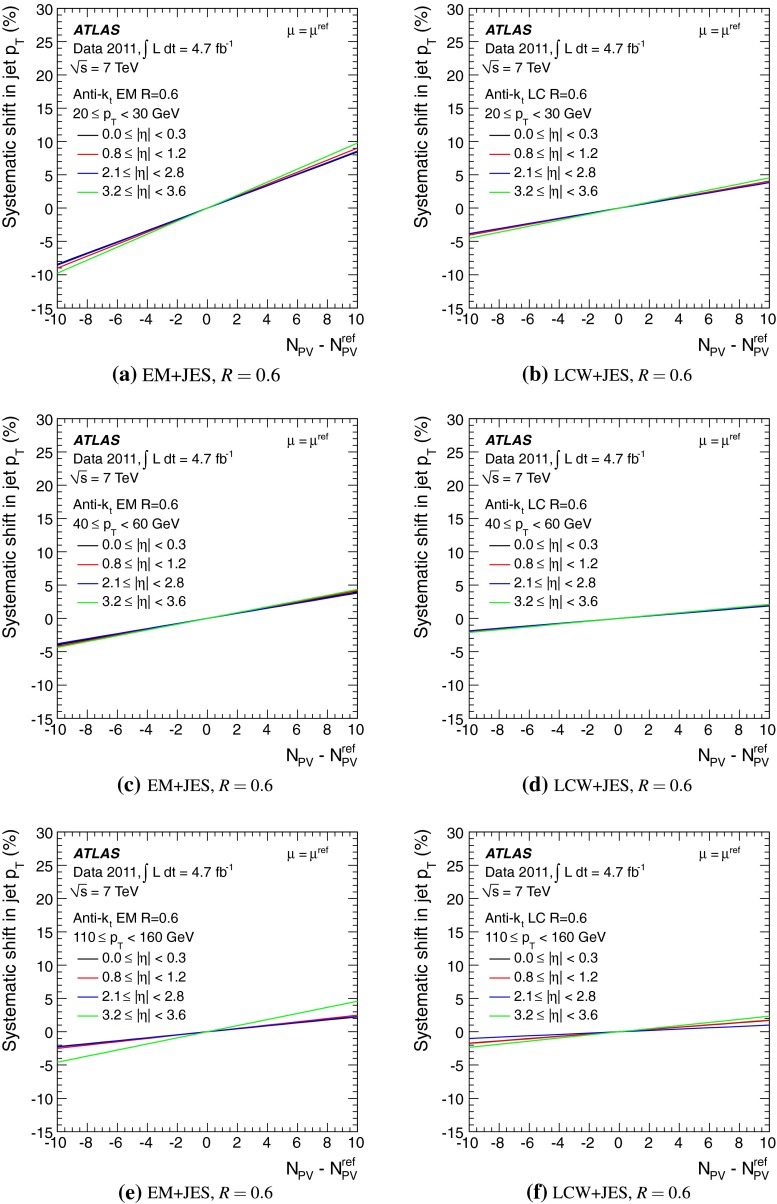
The fractional systematic shift due to mis-modelling of the effect of in-time pile-up on the transverse momentum $p_{\mathrm {T,\mathrm{EM+JES}}}^{\mathrm {jet}}$ of jets reconstructed with the anti-$k_{t}$ algorithm with $R = 0.6$ and calibrated with the EM+JES scheme, is shown as a function of $(N_{\mathrm{PV}}-N_{\mathrm{PV}}^\mathrm{ref})$ in **a**, **c**, and **e** for various $p_{\mathrm {T,\mathrm{EM+JES}}}^{\mathrm {jet}}$ bins. The same systematic shift is shown in **b**, **d**, and **f** for jets calibrated with the LCW+JES scheme, now in bins of $p_{\mathrm {T,\mathrm{LCW+JES}}}^{\mathrm {jet}}$

Figure 46 shows the fractional systematic shift in the $p_{\mathrm {T}}$ measurement for anti-$k_{t}$ jets with $R = 0.4$, as a function of the in-time pile-up activity measured by the displacement $(N_{\mathrm{PV}}-N_{\mathrm{PV}}^\mathrm{ref})$. The shifts are shown for various regions of the ATLAS calorimeters, indicated by $\eta _\mathrm{det}$, and in bins of the reconstructed transverse jet momentum $p_{\mathrm {T,\mathrm{EM+JES}}}^{\mathrm {jet}}$ for jets calibrated with the EM+JES scheme (Fig. 46a, c, e). Figure 46b, d, f show the shifts for jets reconstructed with the LCW+JES scheme in the same regions of ATLAS, in bins of $p_{\mathrm {T,\mathrm{LCW+JES}}}^{\mathrm {jet}}$. The same uncertainty contributions from wider jets reconstructed with the anti-$k_{t}$ algorithm with $R = 0.6$ are shown in Fig. 47.

Both the EM+JES and LCW+JES calibrations are normalised such that the pile-up signal contribution is 0 for $N_{\mathrm{PV}}= N_{\mathrm{PV}}^\mathrm{ref}$ and $\mu = \mu ^{\mathrm {ref}}$, so the fractional systematic shifts associated with pile-up scale linearly with the displacement from this reference. In general, jets reconstructed with EM+JES show a larger systematic shift from in-time pile-up than LCW+JES jets, together with a larger dependence on the jet catchment area defined by $R$, and the jet direction $\eta _\mathrm{det}$. In particular, the shift per reconstructed vertex for LCW+JES jets in the two lowest $p_{\mathrm {T,\mathrm{LCW+JES}}}^{\mathrm {jet}}$ bins shows essentially no dependence on $R$ or $\eta _\mathrm{det}$, as can be seen comparing Figs. 46b and 47b to Figs. 46d and 47d.

**Fig. 48:**
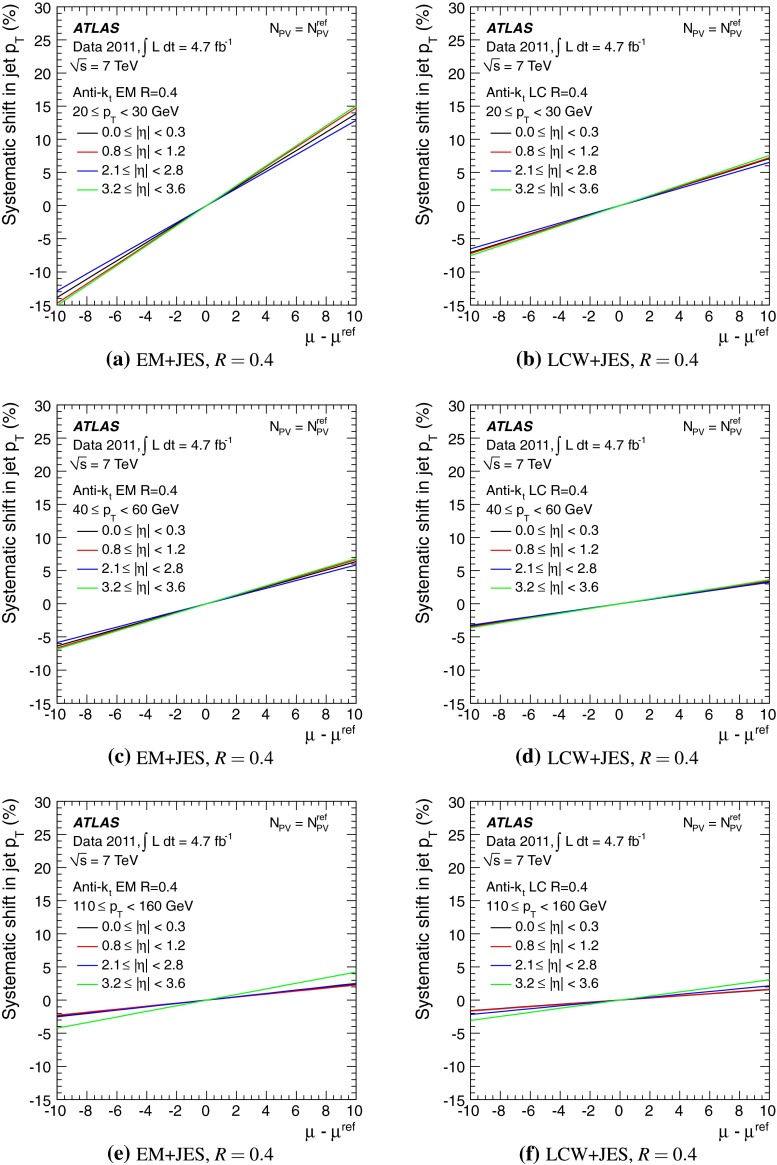
The fractional systematic shift due to mis-modelling of the effect of out-of-time pile-up on the transverse momentum $p_{\mathrm {T,\mathrm{EM+JES}}}^{\mathrm {jet}}$ of jets reconstructed with the anti-$k_{t}$ algorithm with $R = 0.4$ and calibrated with the EM+JES scheme, is shown as a function of $(\mu -\mu ^{\mathrm {ref}})$ in **a**, **c**, and **e** for various $p_{\mathrm {T,\mathrm{EM+JES}}}^{\mathrm {jet}}$ bins. The same systematic shift is shown in **b**, **d**, and **f** for jets calibrated with the LCW+JES scheme, now in bins of $p_{\mathrm {T,\mathrm{LCW+JES}}}^{\mathrm {jet}}$

**Fig. 49:**
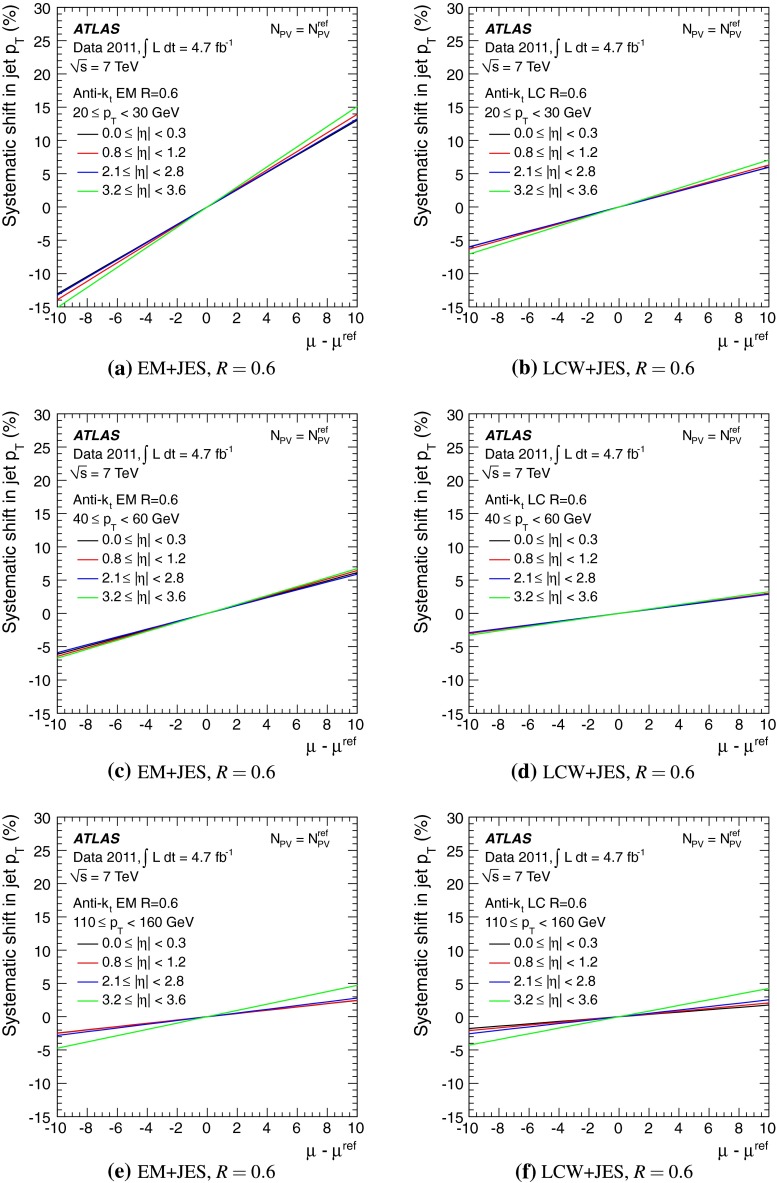
The fractional systematic shift due to mis-modelling of the effect of out-of-time pile-up on the transverse momentum $p_{\mathrm {T,\mathrm{EM+JES}}}^{\mathrm {jet}}$ of jets reconstructed with the anti-$k_{t}$ algorithm with $R = 0.6$ and calibrated with the EM+JES scheme, is shown as a function of $(\mu -\mu ^{\mathrm {ref}})$ in **a**, **c**, and **e** for various $p_{\mathrm {T,\mathrm{EM+JES}}}^{\mathrm {jet}}$ bins. The same systematic shift is shown in **b**, **d**, and **f** for jets calibrated with the LCW+JES scheme, now in bins of $p_{\mathrm {T,\mathrm{LCW+JES}}}^{\mathrm {jet}}$

The systematic shift associated with out-of-time pile-up, on the other hand, is independent of the chosen jet size, as shown in Fig. 48 for $R = 0.4$ and Fig. 49 for $R = 0.6$. Similar to the shift from in-time pile-up, the jets reconstructed with the LCW+JES scheme show smaller systematic shifts from out-of-time pile-up. The results shown in these figures also indicate that the shift from out-of-time pile-up is independent of the jet size. Note that both shifts contribute to the jet $p_{\mathrm {T}}$ reconstruction uncertainty in an uncorrelated fashion, which is justified as while $N_{\mathrm{PV}}$ and $\mu$ are correlated in a given sample, the corrections depending on them are derived independently.

### Summary on pile-up interaction corrections

Dedicated correction methods addressing the signal contributions from in-time and out-of-time pile-up to the jet energy measurement with the ATLAS calorimeters were developed using MC simulations to measure the change of the jet signal as function of the characteristic variables measuring the pile-up activity, which are the number of reconstructed primary vertices $N_{\mathrm{PV}}$ (in-time pile-up) and the average number of pile-up interactions per bunch crossing $\mu$ (out-of-time pile-up). The input to these corrections are the slopes $\alpha = \partial p_{\mathrm {T}}/\partial N_{\mathrm{PV}}$ and $\beta = \partial p_{\mathrm {T}}/\partial \mu$, which are determined in the simulation for two jet signal scales, the EM scale ($p_\mathrm{T, \mathrm{EM}}^\mathrm {jet}$) and the hadronic LCW scale ($p_\mathrm{T, \mathrm{LCW}}^\mathrm {jet}$), both as functions of the truth-jet $p_{\mathrm {T}}^\mathrm {truth}$ and the direction of the jet in the detector $\eta _\mathrm{det}$.

As an alternative to the approach based on MC simulation, the change of the reconstructed (calorimeter) jet $p_{\mathrm {T}}$ with $N_{\mathrm{PV}}$ and $\mu$ can be measured in data using the matching track jet’s $p_{\mathrm {T}}^{\mathrm {track\ jet}}$ as a kinematic reference independent of the pile-up activity. Furthermore, $\gamma \text {-jet}$ events can be used in the same manner, with the photon $p_{\mathrm {T}}$ providing the reference in this case. These experimental methods are restricted by the coverage of the ATLAS tracking detector (track jets), and the lack of significant statistics for events with jets at higher $\eta _\mathrm{det}$ in $\gamma \text {-jet}$ events in 2011.

Comparing the in situ measurements of $\alpha$ and $\beta$ with the corresponding simulation and the findings from the approach solely based on MC simulations allows the determination of systematic biases due to mis-modelling of the effects of pile-up on simulated jets. To cover these biases, uncertainties are assessed as functions of $N_{\mathrm{PV}}$ and $\mu$. These uncertainties amount to less than 0.3 % (0.5 %) of the calibrated jet $p_{\mathrm {T}}$ per reconstructed vertex for central anti-$k_{t}$ jets with $R = 0.4 (0.6)$ with $20 < p_{\mathrm {T}}< 30$ GeV and for $\mu = \mu ^{\mathrm {ref}}$, and about 0.7 % per interaction for jets in the same phase space at $N_{\mathrm{PV}}= N_{\mathrm{PV}}^\mathrm{ref}$, independent of the jet size. The uncertainty contribution in the forward direction can be significantly larger, by up to a factor of two, especially at higher jet $p_{\mathrm {T}}$, where the uncertainty in the central detector is smaller than 0.1 % (0.2) % per vertex and 0.2 % per interaction. These generally small uncertainties can be added in quadrature to give a total fractional uncertainty for each pile-up condition ($N_{\mathrm{PV}}$,$\mu$).

A residual jet $p_{\mathrm {T}}$ dependence of the pile-up correction is observed in MC simulation (see Fig. 45), but not yet fully confirmed in data due to limited size of the data set. It is therefore not explicitly addressed in the correction procedure, rather it is implicitly included into the systematic uncertainties. This dependence, which is not expected for a purely stochastic and diffuse signal contribution from both in-time and out-of-time pile-up, is introduced by the topo-clusters formation in the calorimeter, which enhances the survivability of small (pile-up) signals if higher density signals such as those in the core of a jet are close by. At very high jet $p_{\mathrm {T}}$, this dependence reaches a plateau, since the jet core gets so dense that all calorimeter cells contribute to the jet signal, and therefore all signal generated by pile-up in these cells is directly included in the jet signal.

In summary, the pile-up signal contribution to jets in the ATLAS detector is well understood. The correction based on MC simulations controls this contribution to a high precision with uncertainties of less than 1 % per reconstructed primary vertex and additional proton–proton collision per bunch crossing, yielding a small fractional contribution to the overall jet energy scale uncertainty over the whole phase space, except for the very forward region, where this uncertainty can be more significant.

**Fig. 50:**
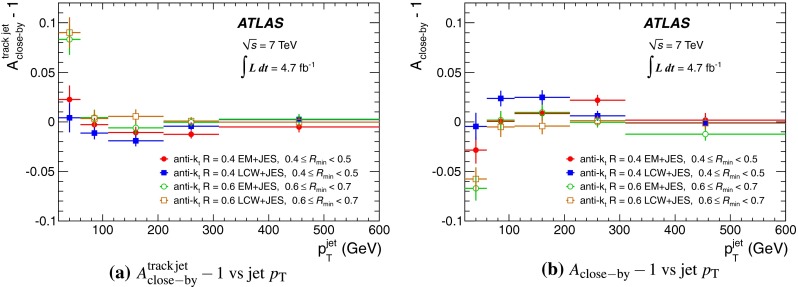
In **a**, the deviation from unity of the data-to-MC ratio of the track-jet $p_{\mathrm {T}}$ for non-isolated jets divided by the track-jet $p_{\mathrm {T}}$ for isolated jets, is shown as a function of the jet $p_{\mathrm {T}}$. The deviation from unity of the data-to-MC ratio of the relative response of non-isolated jets with respect to that of isolated jets as a function of the jet $p_{\mathrm {T}}$ is shown in **b**. As described in the text, the distributions show the ratios given in **a** Eq. (16) and **b** Eq. (15) for the four jet calibration schemes. Only statistical uncertainties are shown

## Close-by jet effects on jet energy scale

The variation of the jet energy response due to nearby jets and the associated systematic uncertainty are reported in Ref. [3], using the data collected in 2010. The same analysis is performed to reassess this uncertainty for the 2011 data.

The analysis uses track jets from the primary vertex, as defined in Sect. 5.4, as a kinematic reference. The calorimeter jet’s transverse momentum $p_{\mathrm {T}}^\mathrm {jet}$ relative to the track-jet transverse momentum $p_{\mathrm {T}}^{\mathrm {track\ jet}}$ provides an in situ validation of the calorimeter jet response and the evaluation of the systematic uncertainty. The relative response measurement is performed in bins of $R_\mathrm{min}$, the distance in ($\eta ,\phi$) space from the jet to the closest other jet with $p_{\mathrm {T}}>7$ GeV at the EM scale. The response to track jets is also evaluated for the non-isolated condition $R_\mathrm{min} < 2.5 \times R$, where $R$ is the distance parameter used in the anti-$k_{t}$ jet reconstruction, and the associated systematic uncertainty is assessed. In the relative response measurement, the track jet is matched to the calorimeter jet with the distance requirement $\Delta R<0.3$, where $\Delta R$ is measured according to Eq. (3) (Sect. 5.6) in ($\eta ,\phi$) space. When two or more jets are matched within the $\Delta R$ range, the closest matched jet is taken.

The calorimeter jet response relative to the matched track jet, defined as the $p_{\mathrm {T}}$ ratio of the calorimeter to the track jet as a function of $p_{\mathrm {T}}^\mathrm {jet}$,

$$\begin{aligned} r^\mathrm{calo/\mathrm{track\,jet}}=p_{\mathrm {T}}^\mathrm {jet}/p_{\mathrm {T}}^{\mathrm {track\ jet}}, \end{aligned}$$

is examined for different $R_\mathrm{min}$ values measured for the two close-by calorimeter jets.^18^ The ratio of calorimeter jet response between non-isolated (i.e, small $R_\mathrm{min}$) and isolated (large $R_\mathrm{min}$) jets, given by

$$\begin{aligned} r^\mathrm{calo/\mathrm{track\,jet}}_\mathrm{non-iso/iso} = \dfrac{r^\mathrm{calo/\mathrm{track\,jet}}_\mathrm{non-iso}}{r^\mathrm{calo/\mathrm{track\,jet}}_\mathrm{iso}} , \end{aligned}$$

is compared between data and MC simulation. The relative difference between them,

15 $$\begin{aligned} A_\mathrm{close-by} = \dfrac{\left. r^\mathrm{calo/\mathrm{track\,jet}}_\mathrm{non-iso/iso}\right| _\mathrm{Data}}{\left. r^\mathrm{calo/\mathrm{track\,jet}}_\mathrm{non-iso/iso}\right| _\mathrm{MC}} , \end{aligned}$$

is assumed to represent the calorimeter JES uncertainty due to close-by jets. This uncertainty, convolved with the systematic uncertainty of the response to a track jet with a nearby jet, and evaluated in a similar way as the data-to-MC difference between the average $p_{\mathrm {T}}$ ratio of the non-isolated to isolated track jets, provides the total JES systematic uncertainty due to the close-by jet effect.

### Samples and event selection

Data collected with four single-jet, pre-scaled triggers with jet-$p_{\mathrm {T}}$ thresholds of 10, 30, 55 and 135 GeV are used in the analysis. As in the MJB analysis discussed in Sect. 11, the data from a given trigger are used in a certain non-overlapping jet-$p_{\mathrm {T}}$ range where the trigger is greater than 99 % efficient. For MC simulation, the baseline Pythia samples described in Sect. 3 are used.

Events passing the trigger selections are required to satisfy the same primary vertex and event cleaning criteria for jets due to noise and detector problems as those used in the MJB analysis (see Sect. 11.2). Finally, events that contain at least two jets with calibrated $p_{\mathrm {T}}>20$  GeV and rapidity $|y|<2.8$ are selected for the analysis.

The track jets are reconstructed from the selected tracks by using the anti-$k_{t}$ algorithm with $R = 0.4$ and $R = 0.6$, as described in Sect. 5.4. In the analysis presented below, track jets with $p_{\mathrm {T}}>10$ GeV and $|\eta |<2.0$, composed of at least two tracks, are used. The close-by jet energy scale uncertainty is therefore assessed in the region of $|\eta |<2.0$ where the calorimeter jets and track jets can be matched in $\eta$ and $\phi$.

### Non-isolated jet energy scale uncertainty

The average track-jet transverse momentum is examined as a function of the calorimeter jet $p_{\mathrm {T}}$ for different $R_\mathrm{min}$ values starting from the jet radius in bins of $\Delta R_\mathrm{min}=0.1$. The ratio of the average track-jet $p_{\mathrm {T}}$ between the non-isolated and isolated track jets $p_{\mathrm {T}}^{\text {non-iso}}/p_{\mathrm {T}}^\mathrm{iso}$ in bin of the calorimeter $p_{\mathrm {T}}$, is used to quantify the uncertainty in the response to track jets. This comparison is shown in Fig. 50a as a deviation from unity of the data-to-MC ratio:

16 $$\begin{aligned} A_{\text {close-by}}^\mathrm{track\,jet} = \dfrac{\left. p_{\mathrm {T}}^{\text {non-iso}}/p_{\mathrm {T}}^\mathrm{iso}\right| _\mathrm{Data}}{\left. p_{\mathrm {T}}^\mathrm{non-iso}/p_{\mathrm {T}}^\mathrm{iso}\right| _\mathrm{MC}} \end{aligned}$$

to quantify the uncertainty in the response to track jets in the small $R_\mathrm{min}$ range of $R\le R_\mathrm{min}<R+0.1$. The $A_{\text {close-by}}^{\mathrm{track\,jet}}$ has a strong $R_\mathrm{min}$ dependence, especially at small $\Delta R_\mathrm{min}$ range where the close-by jet overlaps the probe jet, and the dependence is more significant for jets with $R = 0.6$. The agreement between data and MC simulations improves with increasing $R_\mathrm{min}$.

The calorimeter jet response relative to the matched track jet ($r^\mathrm{calo/\mathrm{track\,jet}}$) is investigated as a function of $p_{\mathrm {T}}$, in terms of the non-isolated jet response relative to the isolated jet response $r^\mathrm{calo/\mathrm{track\,jet}}_\mathrm{non-iso/iso}$, for data and MC simulations. The data-to-MC ratio $A_\mathrm{close-by}$ of $r^\mathrm{calo/\mathrm{track\,jet}}_\mathrm{non-iso/iso}$ is shown in Fig. 50b as the deviation from unity for the range of $R\le R_\mathrm{min}<R+0.1$. As already seen in the track-jet response in Fig. 50a, there is a strong $R_\mathrm{min}$ dependence on $A_\mathrm{close-by}$ within the small $R_\mathrm{min}$ range mentioned above. The deviation of $A_\mathrm{close-by}$ from unity is added in quadrature with the track-jet response uncertainties obtained above to get the overall JES uncertainties due to close-by jet effects. The convoluted uncertainty is about 3.5 % (10 %) at $R_\mathrm{min}<0.5$ (0.7) for $R = 0.4$ (0.6) jets with $p_{\mathrm {T}}=30$ GeV, and becomes smaller than 1 % at $R_\mathrm{min}$ above 0.8 for both sizes of jets. The uncertainty decreases with increasing jet $p_{\mathrm {T}}$ and becomes about 2 % (4 %) at $R_\mathrm{min}<0.5$ (0.7) for $R = 0.4$ (0.6) jets with $p_{\mathrm {T}}=100$ GeV.

## Jet response difference for quark and gluon induced jets and associated uncertainty

All jet calibration schemes developed in ATLAS achieve an average response of the calorimeter to jets near unity for jets in the inclusive jet sample. However, the calorimeter response to jets also exhibits variations that can be correlated to the flavour of the partons (i.e., light or heavy quarks, or gluons) produced in the sample under study. This dependence is to a large extent due to differences in fragmentation and showering properties of jets loosely labelled as originating from a light quark or a gluon.

In this section, the dependence of the jet energy scale on whether a jet originates from a light quark or a gluon is studied. Also, a systematic uncertainty that accounts for the sample dependence of the jet energy scale is established using different MC simulations. In addition, jet properties that can be shown to discriminate between jets initiated by light quarks and gluons are used to build a light-quark/gluon tagger [3, 94]. The focus in this section is on understanding how the JES is affected by a selection based on the light-quark/gluon tagger, and the implications for the sample-dependent systematic uncertainty described if jets are tagged using this tagger. Details of the procedure to built a quark-gluon tagger can be found in Ref. [95].

### Event selection

#### Jet and track selection

Calorimeter jets with transverse momentum $p_{\mathrm {T}}>20~\mathrm{GeV }$ and $|\eta |<4.5$ are reconstructed using the anti-$k_{t}$ jet algorithm with $R=0.4$.

The variables described in Sect. 18.3 are constructed to describe the properties of jets. They are based on tracks with $p_{\mathrm {T}}^{\mathrm {track}}>1$  GeV that are associated to jets if they are within a distance $\Delta R= R$ (equal to the distance parameter $R$ used to build the jet) of the jet axis. The tracks are further selected as described in Sect. 5.4, with slightly modified quality requirements in order to provide an even stronger association to the primary vertex (impact parameters $z_0 \sin (\theta )<1$ mm and $d_0<1$ mm).

#### Jet flavour definition

Jets are labelled by partonic flavour, if they have $p_{\mathrm {T}}>40~\mathrm{GeV }$ and $|\eta |<2.1$. They are matched to the highest-energy parton found inside the cone of the jet. This parton can be produced directly off the hard scatter, or by radiation.

This definition of partonic jet flavour is not theoretically sound, and that may have implications when attempting to apply this labelling to physics analyses. However, several studies with MadGraph [34] have demonstrated that this definition is not changed by the parton shower model choices, and is equivalent to a matrix-element-based labelling for over 95 % of jets. Since the partonic flavour of a jet can only be easily defined in leading order, and since only a labelling indicating differences in jet properties is required for the performance evaluations presented in this paper, this definition is sufficient.

#### Dataset for flavour studies

Two main event samples are used. The first selects inclusive jet events (dijet sample). The second selects jets with a high-transverse momentum photon back-to-back with a jet ($\gamma \text {-jet}$ sample). Both samples are defined using standard data-quality criteria and the requirement of a primary vertex with at least three associated tracks.

Central jet triggers are used for the dijet sample selection. These triggers provide a fully efficient jet selection for $p_{\mathrm {T}}> 40$ GeV. Jet triggers with $p_{\mathrm {T}}$ thresholds less than 500  GeV are pre-scaled, so that only a fraction of the events in this kinematic regime are recorded.

The $\gamma \text {-jet}$ sample is selected as described in Sect. 10. In addition, a photon with $p_{\mathrm {T}}>45$  GeV in the event is required to be back-to-back (azimuthal distance $\Delta \phi >2.8$ rad) to the leading jet. The sub-leading jet is required to have no more than 30 % of the photon $p_{\mathrm {T}}$.

**Fig. 51:**
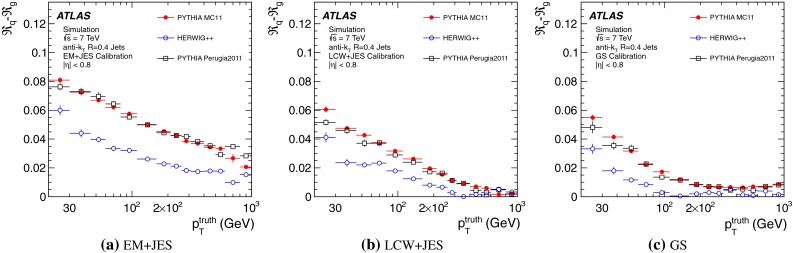
Difference in jet response $\mathcal {R} = p_{\mathrm {T}}^\mathrm {jet}/p_{\mathrm {T}}^\mathrm {truth}$ of isolated jets initiated by light quarks and gluons as a function of the true jet $p_{\mathrm {T}}$, for anti-$k_{t}$ jets with $R=0.4$ in the barrel calorimeter. Three different calibration schemes are shown for **a** the EM+JES calibration, **b** the LCW+JES calibration, and **c** the alternative Global Sequential (GS) [3] scheme. Three different MC simulation samples are also shown, Pythia (*solid red circles*), Herwig++ (*open blue circles*) and Pythia Perugia2011 (*open black squares*)

### Calorimeter response to quark and gluon induced jets

Jets labelled as originating from light quarks have significantly different response ($p_{\mathrm {T}}^\mathrm {jet}/p_{\mathrm {T}}^\mathrm {truth}$) from those labelled as originating from gluons in the MC simulation. This difference is a result of a difference in fragmentation that can be correlated to differences in observable properties of the two types of jets. Gluon jets tend to have more particles, and as a result, those particles tend to have lower $p_{\mathrm {T}}$ than in the case of light-quark jets. Additionally, gluon jets tend to have a wider angular energy profile before interacting with the detector.

The harder particles in light-quark jets have a higher probability of penetrating further into the calorimeter, and thus more often reaching the hadronic calorimeter layers. The lower response of the calorimeter for low-$p_{\mathrm {T}}$ particles combined with threshold and response effects related to the energy density inside the jet suggest that gluon jets should have a lower response than light-quark jets. The difference in calorimeter response in MC simulations between isolated light-quark and gluon jets is shown in Fig. 51, for anti-$k_{t}$ jets with $R = 0.4$ in the barrel calorimeter ($|\eta _\mathrm{det}|<0.8$).

Independent of the calibration scheme, the flavour-dependent response difference is largest at low $p_{\mathrm {T}}$ (up to 8 % for EM+JES), and decreases to a few percent at high $p_{\mathrm {T}}$. A more sophisticated calibration scheme like LCW+JES reduces the differences, because it exploits signal features of individual particle showers in the calorimeter for calibration, and thus partly compensates for variations in jet fragmentation and directional energy flow in the jet. Even more so, the Global Sequential (GS) calibration introduced in Ref. [3], which can be applied on top of the (standard) EM+JES or LCW+JES calibration, or just to jets at the EM scale as done for the studies discussed here, shows the best performance at low $p_{\mathrm {T}}$. This is due to its explicit use of a jet width variable which is strongly related with the transverse structure of the jet and is thus sensitive to differences between jets initiated by light-quarks and gluons. The response difference between light-quark- and gluon-initiated jets is reduced by roughly 1 % for anti-$k_{t}$ jets with $R=0.6$, because the larger jet area diminishes the effect of the energy loss of the broader jet.

The differences in response between jets initiated by light quarks and gluons can impact analyses in which the flavour composition of the sample is not well known. The corresponding JES uncertainties can be reduced if the flavour composition of the analysis sample is known and the accuracy of the MC description of the data can be established. This uncertainty can be extracted directly from Fig. 51 and amounts to about 2 % at low $p_{\mathrm {T}}$ and 0.5 % at high $p_{\mathrm {T}}$ for the EM+JES calibration, if the flavour composition of the sample is known within 25 %. It can be reduced by a factor of two at low $p_{\mathrm {T}}$ and even more at high $p_{\mathrm {T}}$ through the use of one of the more sophisticated calibration schemes.

These response differences between jets initiated by light quarks and gluons result in a sample dependence of the energy scale and suggests that the JES calibration determined from in situ techniques might only be applicable within a larger systematic uncertainty to different jet samples. With the techniques commissioned up to date, the 2011 dataset only allows for a coarse validation of the differences in the jet energy scale between light-quark- and gluon-initiated jets. MC simulations are instead used to understand the impact of systematic effects in the response differences between light-quark and gluon jets.

Figure 51 shows the jet response difference between jets initiated by light quarks and gluons in the central $|\eta _\mathrm{det}|$ region of ATLAS for Pythia (standard ATLAS MC11 tune), Pythia (Perugia2011 tune) and Herwig++. Comparisons between the first two simulations show the impact of the underlying event tune on the response differences. Comparisons between Pythia and Herwig++ provide an estimate of the impact of differences in the modelling of the parton shower, fragmentation and hadronisation for generators modelling the jet fragmentation well within the constraints provided by data. The differences in the response between these two models are large, while the effect of the underlying event tune is small, as can be seen by comparing the standard Pythia MC11 tune with the Perugia2011 tune.

**Fig. 52:**
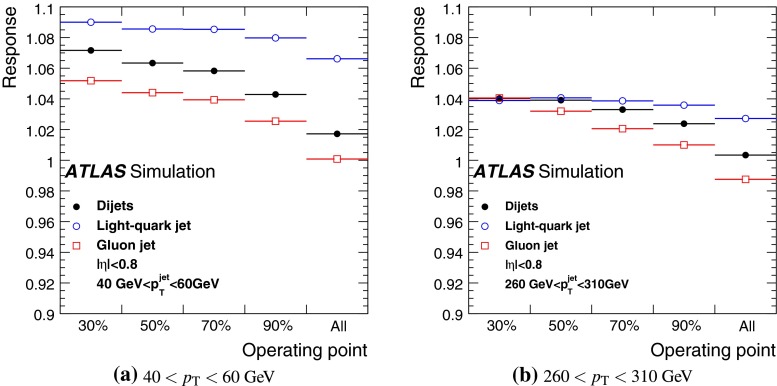
Jet $p_{\mathrm {T}}$ response for the two leading jets in the dijet sample for different tagger light-quark operating points for jets with **a** $40~\mathrm{GeV }<p_{\mathrm {T}}<60~\mathrm{GeV }$ and **b** $260~\mathrm{GeV }<p_{\mathrm {T}}<310~\mathrm{GeV }$, and $|\eta _\mathrm{det}| <0.8$. Jets are labelled as light quark or gluon using the MC-simulation record and are further required to be isolated

Further analysis of the large differences between Pythia and Herwig++ indicate that the cause is almost exclusively the difference in the response to gluon jets. This leads to a sizable response difference for the inclusive jet sample, which in the lower-$p_{\mathrm {T}}$ region has mainly gluon-initiated jets in the final state. Significantly smaller differences are observed in the samples used to calibrate the absolute jet response in the lower-$p_{\mathrm {T}}$ regime, like $\gamma \text {-jet}$ and $Z$-jet, which have a dominant contribution from light-quark jets.

The systematic effect illustrated by the difference between the two MC simulations can be included as an additional systematic uncertainty. For this, the response variation $\Delta \mathcal {R}_{\mathcal {S}}$ for a given event sample $\mathcal {S}$ can be written as

17 $$\begin{aligned} \Delta \mathcal {R}_\mathcal {S}&= \Delta f_g(\mathcal {R}_g-1)+\Delta f_{uds}(\mathcal {R}_{uds}-1)\nonumber \\&+ f_g\Delta \mathcal {R}_g + f_{uds}\Delta \mathcal {R}_{uds} + f_b\Delta \mathcal {R}_b + f_c\Delta \mathcal {R}_c ,\nonumber \\ \end{aligned}$$

where $\mathcal {R}_{g}$, $\mathcal {R}_{uds}$, $\mathcal {R}_{l}$, and $\mathcal {R}_{b}$ refer to the response to jets initiated by gluons, light ($u$, $d$, $s$) quarks, $c$-quarks, and $b$-quarks, with $\Delta$ denoting the uncertainty on the respective variable. The fractions $f_{x}$ refer to the fractions of jets with a given partonic flavour $x \in \{ g, uds, c, b \}$ in the sample $s$. Under the simplifying assumption that the jet energy scale uncertainty is established in situ for light-quark jets and that it is the same for jets from $b$-quarks and $c$-quarks, Eq. (17) can be simplified to

18 $$\begin{aligned} \Delta \mathcal {R}_{\mathcal {S}}=\Delta f_q(\mathcal {R}_q-\mathcal {R}_g)+\Delta \mathcal {R}_q+f_g\Delta \mathcal {R}^\mathrm{ex}_{g} , \end{aligned}$$

where $\Delta \mathcal {R}_q\equiv \Delta \mathcal {R}_{uds}\equiv \Delta \mathcal {R}_b\equiv \Delta \mathcal {R}_c$ and $f_{q} = f_{uds} + f_{l} + f_{b} = 1 - f_{g}$. The additional term $\Delta \mathcal {R}^\mathrm{ex}_{g}$ reflects an additional variation that represents the uncertainty on the response of gluon jets that arises from the systematic effects captured by the different MC simulations. Note that the first term of this equation is used to estimate the effect of the results shown in Fig. 51 on the systematic uncertainty of the jet energy scale in a sample of imprecisely known flavour composition.

The additional term $\mathcal {R}^\mathrm{ex}_{g}$ was not added to the 2010 ATLAS jet energy scale uncertainty for simplicity, since it was much smaller than the dominant contributing effects. The improvements in the jet energy measurement achieved with the 2011 dataset require this more careful treatment. Using the response difference $\mathcal {R}_{q} - \mathcal {R}_{g}$ with the EM+JES calibration at low $p_{\mathrm {T}}$ shown in Fig. 51, the uncertainty on $\mathcal {R}^\mathrm{ex}_{g}$ amounts to about 3 % in a sample with 75 % gluon content, which is close to the inclusive jet sample. It is reduced to about 1 % in a sample with 25 % gluon content, as expected for $t\bar{t}$ with radiation. The uncertainty at high $p_{\mathrm {T}}$ is smaller than 1 %. This term in the uncertainty can also be reduced by a factor of 2 or more when using the more evolved calibration schemes LCW+JES or GS.

The in situ jet energy scale uncertainty is derived using $\gamma \text {-jet}$ and $Z$-jet samples, which at low $p_{\mathrm {T}}$ are dominated by light-quark jets. The expression for the total uncertainty presented here could be generalised to account for the fact that there is some gluon-initiated jet contamination, and that the uncertainty on the light-quark jet response $\Delta \mathcal {R}_q$ cannot be established using these samples alone. However, the approximation that the $\gamma \text {-jet}$ and $Z$-jet sample are pure light-quark jet samples is most accurate at low $p_{\mathrm {T}}$, where the gluon jet response uncertainty is largest. Thus, this approximation leads to inaccuracies that are significantly smaller than other systematic uncertainties in the average jet response.

### Discrimination of light-quark and gluon induced jets

As indicated before, the differences between light-quark and gluon jets lead to (average) differences in observable final-state jet properties. Jets initiated by gluons are expected to be broader, with more low-$p_{\mathrm {T}}$ particles than those initiated by light quarks. Relevant observables like the jet width $w_{\mathrm {jet}}$, as reconstructed using the $p_{\mathrm {T}}$ flow of tracks associated with the jet, and the number of those tracks $n_{\mathrm {trk}}$, are already used to measure the average flavour fractions in different data samples [3]. They are identified as powerful discriminators for the purpose of understanding partonic flavour in previous studies [94]. More details on the quark-gluon tagger performance in the ATLAS detector can be found in Ref. [95].

These jet properties, reconstructed using selected high-quality tracks, are further exploited to build a likelihood discriminator or a light-quark/gluon tagger. Two-dimensional $(n_{\mathrm {trk}},w_{\mathrm {jet}})$ distributions are determined for data and MC simulations using the inclusive jet and $\gamma \text {-jet}$ event samples. The different fractions of light-quarks and gluons in these samples, which in MC simulation are extracted from Pythia with the ATLAS MC11 tune, are then reflected by variations in the $(n_{\mathrm {trk}},w_{\mathrm {jet}})$ distributions, and the expected “pure” jet sample properties can be extracted. This procedure is applied both in data and MC simulations, and both data-driven and MC-based taggers are built. Operating points are defined at fixed light-quark jet efficiencies of 30, 50, 70 and 90 %, using the same extracted $(n_{\mathrm {trk}},w_{\mathrm {jet}})$ distributions.

The quark/gluon tagger essentially selects jets with both decreasing $n_{\mathrm {trk}}$ and $w_{\mathrm {jet}}$ as the operating point tightens, to achieve a higher gluon jet rejection at the expense of a lower light-quark jet efficiency. It can then be expected that jets selected with different operating points of the tagger have different jet energy scales. This is shown in Fig. 52, where the response as a function of the operating point used to select jets in an inclusive MC-simulation sample is shown for two $p_{\mathrm {T}}$ bins for jets calibrated with the EM+JES calibration.

Even choosing a high efficiency operating point increases the sample response significantly, particularly at low $p_{\mathrm {T}}$, compared to the inclusive sample. The difference in response between light-quark and gluon jets is largest for the inclusive sample, and basically vanishes for the tightest operating point at high jet $p_{\mathrm {T}}$. This is expected, since it is shown in Fig. 51 that applying a $n_{\mathrm {trk}}$- and $w_{\mathrm {jet}}$-based JES correction like GS removes the response differences between light-quark and gluon jets at high $p_{\mathrm {T}}$. In addition, these jets are selected by the likelihood because they have quite similar (quark-jet-like) observable properties.

To gain confidence that the change in jet response does not affect analyses using the tagger, it is necessary to demonstrate that the agreement of the jet energy scale between MC simulations and data does not change when the likelihood cut corresponding to each operating point is applied. This is verified using the $\gamma \text {-jet}$ balance technique described in Sect. 10, which finds changes of the data-to-MC agreement to be below 1 %.

The same $p_{\mathrm {T}}$-balancing technique allows for a study of the dependence of the JES on the tagger operating point in a specific sample, but not for an investigation of the light-quark and gluon jet responses directly. This is controlled through the sample- and flavour-dependent systematic uncertainties described in the previous section and summarised in Eq. (18). The first term in this equation is based on the differences between light-quark and gluon JES, which become smaller when the tagger is used, as shown in Fig. 52. The second term is calculated comparing Herwig++ and Pythia in the dijet sample. Both comparisons are performed for tagged jets, and they demonstrate that these uncertainties are actually smaller after the application of the tagger than before. The use of the uncertainties derived in the previous section is thus conservative for tagged jets, and the validation in the gluon jet sample is sufficient.

### Summary of the jet flavour dependence analysis

The dependence of the jet energy scale on the flavour of the originating parton of the jet is evaluated in MC simulations. This difference, which enters the JES systematic uncertainty, is shown to be sensitive to certain details of the modelling of the decay and fragmentation of jets in the MC generators. An additional term is derived that needs to be added to the JES uncertainty to account for this dependence. It amounts to about 3 % in a sample with a 75 % gluon content (close to the inclusive jet sample) and is reduced to about 1 % in a sample with 25 % gluon content at low $p_{\mathrm {T}}$ when using the EM+JES calibration scheme. The uncertainty at high $p_{\mathrm {T}}$ is smaller than 1 %. This contribution to the JES uncertainty can also be reduced by a factor of two or more when using the more sophisticated calibration schemes and is included as a part of the combined ATLAS jet energy scale uncertainty.

The flavour dependence of the JES arises to a great extent from differences in observable properties of jets, such as the number of tracks and the jet width measured with tracks. These properties can be used to reduce this dependence, as well as to discriminate between light-quark and gluon jets. The properties are used in ATLAS to build a quark/gluon jet tagger exploiting the differences in flavour composition between an inclusive jet and a $\gamma \text {-jet}$ sample, in data as well as in MC simulations. The JES dependence on the choice of operating point used in the tagger yields a data-to-MC difference of less than 1 %. Furthermore, the sample dependent uncertainties become smaller once jets are tagged, since the fragmentation is constrained to a specific phase space for which differences between light-quark and gluon jets between different MC generator models are smaller.

## Jets with heavy-flavour content

In this section the measurement of the jet energy is studied for jets from heavy-flavour decays. The main observable used in the corresponding analysis based both on MC simulations and in situ techniques is the ratio $r_\mathrm{trk}$ of the sum of transverse momentum vectors $\vec {p}_{\mathrm {T}}^{\;\mathrm {track}}$ from all tracks in the jet cone to the calorimeter jet transverse momentum $p_{\mathrm {T}}^\mathrm {jet}$,

19 $$\begin{aligned} r_\mathrm{trk}= \dfrac{| \sum \vec {p}_{\mathrm {T}}^{\;\mathrm {track}}|}{p_{\mathrm {T}}^\mathrm {jet}}. \end{aligned}$$

These studies assess the jet energy measurement in the calorimeter in light-jet-enriched samples as well as for *b*-jet-enriched samples in an inclusive jet sample and in an event sample where a top-quark pair is produced ($t\bar{t}$). The uncertainty on the *b*-jet energy measurement is thus evaluated over a wide range of $p_{\mathrm {T}}$ and under different background conditions. Furthermore, the $p_{\mathrm {T}}$ imbalance in a dijet system is used to validate the description of the kinematics of the neutrino coming from $b$-quarks decaying semileptonically in the MC simulation.

In the following jets originating from a $b$-quark (*b*-jets) and identified by means of $b$-tagging techniques are referred to as “$b$-tagged jets”. The notation “inclusive jets” is used to denote a mixture of jets initiated by light quarks, $b$-quarks, and gluons. All types of jets originating from $b$-quarks, including those containing semileptonic $b$-quark decays, are referred to as “inclusive *b*-jets”.

Since an unbiased sample of jets induced by charm quarks can not be selected in the data, no dedicated studies for charm jets have been performed. Charm jets are considered to be light jets and are treated as described in Sect. 18.

### Jet selection and response definition

Jets with a calibrated transverse momentum $p_{\mathrm {T}}^\mathrm {jet}> 20$ GeV and a pseudorapidity $|\eta | < 2.5$ are used in this study.

Two aspects of the jet energy scale are studied separately: the response to particles absorbed in the calorimeter and the detector response to all produced particles including muons and neutrinos. The former is characterised by the calorimeter response $\mathcal {R}^\mathrm{calo}=p_{\mathrm {T}}^\mathrm {jet}/p_{\mathrm {T}}^{\mathrm {truth}}$, where $p_{\mathrm {T}}^{\mathrm {truth}}$ is the $p_{\mathrm {T}}$ of a matched truth jet built from stable final-state particles, as defined in Sect. 5.5, with the exclusion of muons and neutrinos. The latter is characterised by the all-particle response $\mathcal {R}^\mathrm{all}=p_{\mathrm {T}}^\mathrm{jet+\mu }/p_{\mathrm {T}}^\mathrm{truth, all}$, where $p_{\mathrm {T}}^\mathrm{jet+\mu }$ includes selected reconstructed muons inside the jet and $p_{\mathrm {T}}^\mathrm{truth, all}$ is the $p_{\mathrm {T}}$ of a matched truth jet built from all stable final-state particles.

The jet energy scale of $b$-tagged jets in the dijet sample is studied using different $b$-tagging algorithms. For each algorithm, different operating points resulting in different efficiencies and purities are studied, as detailed in Sect. 19.3. In the MC simulation, the flavour of jets is determined as described in Ref. [91], by the presence of a heavy-flavour quark matched geometrically to the reconstructed jet, using the distance $\Delta R$ in ($\eta ,\phi$) space, see Eq. (3) in Sect. 5.6.

In the $t\bar{t}$ sample $b$-tagged jets are selected by means of the MV1 tagger [91]. The MV1 tagger uses the results from three $b$-tagging algorithms exploiting secondary-vertex and track impact-parameter information, which are input to a neural network to derive a likelihood discriminant to select *b*-jets. In this analysis, a jet is experimentally identified as a *b*-jet if the MV1 tagger weight ($w_{\text {{MV1}}}$) exceeds a threshold value of 0.6. This corresponds to 70 % per-jet efficiency for selecting *b*-jets from $t\bar{t}$ decays, and a per-jet rejection factor for light-quark jets of about 130. To adjust the MC simulations to the $b$-tagging performance in data, a dedicated $b$-tagging efficiency correction [91] is applied to the simulation and the related systematic uncertainties are evaluated.

The influence of nearby jets on the measurements is studied by applying an isolation requirement which rejects jets that are separated from the nearest other jet by a distance $\Delta R< 2 R$. The influence of this requirement is found to be negligible in the analyses presented, so the requirement is omitted in the results shown.

**Fig. 53:**
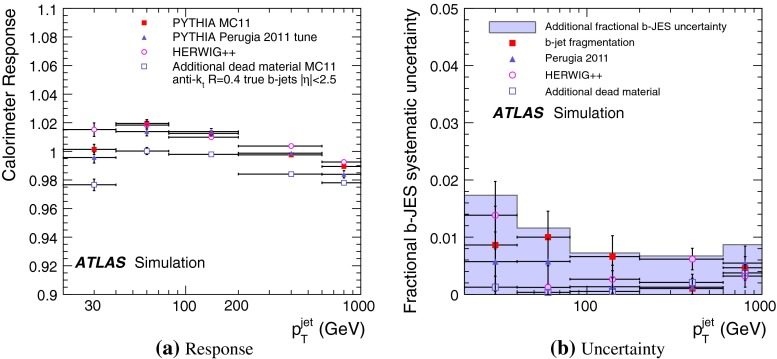
Average response to *b*-jets in some of the different samples used to calculate the *b*-jet energy scale systematic uncertainties is depicted in **a**. The resulting uncertainties in the ratio of the *b*-jet response to the response of jets in an inclusive sample are shown in **b**. These results are obtained for *b*-jets built with the anti-$k_{t}$ algorithm with resolution parameter $R = 0.4$

The jet vertex fraction $\mathrm {JVF}$ introduced in Sect. 8.2.3 is used to quantify the amount of energy in a jet coming from pile-up interactions.

### Track selection

Tracks are associated to jets by requiring that the opening angle between the track and the jet direction be $\Delta R(\mathrm{jet,track})<0.4$, measured in ($\eta ,\phi$) space. Tracks are required to pass the track selection criteria presented in Sect. 5.4 in the context of track jets. This assures an appropriate reconstruction quality and that the selected tracks come from the primary hard-scattering vertex.

### Event selection

Events are initially selected by means of single-jet and single-lepton triggers. A primary vertex reconstructed from at least five tracks, which is consistent with the position and transverse size of the beam, is required. Analysis specific selections are described below.

#### Jet sample selection

Four complementary event selections are used for studies in the dijet sample:

1. An inclusive selection is used to study the energy calibration in the inclusive jet sample, and uses 11 single-jet triggers to cover the full $p_{\mathrm {T}}$ range, to cope with the reduced data rate allowed for lower-$p_{\mathrm {T}}$ triggers.
2. Two $b$-tagged jet selections are used to study the energy calibration of *b*-jets.
  - An inclusive $b$-tagged sample is selected using five different single-jet triggers, since the range of $p_{\mathrm {T}}$ for *b*-jet studies is limited by the low trigger rates at low $p_{\mathrm {T}}$ and by the measurements of $b$-tagging efficiencies at high $p_{\mathrm {T}}$.
  - A semileptonic $b$-tagged sample is selected using a single muon–jet trigger, requiring a muon candidate inside a jet, which is less heavily pre-scaled, increasing the size of the sample collected with respect to a sample collected with a single-jet trigger.
3. A dijet selection is used to study the impact of semileptonic decays into muons and neutrinos.

Only one trigger is used to collect events in a specific $p_{\mathrm {T}}$ bin. This procedure is found to be compatible within statistical uncertainties with a procedure that combines all jet triggers in each $p_{\mathrm {T}}$ bin by weighting contributing events according to the integrated luminosity collected by the trigger that allowed the event to be recorded.

The measurement in the dijet sample is performed as a function of the average $p_{\mathrm {T}}$ ($p_{\mathrm {T}}^\mathrm {avg}$) of the two leading jets, including the muon candidate if one is reconstructed inside the jet. The estimated muon energy loss in the active layers of the calorimeter is subtracted to avoid double counting.

The measurement in the inclusive samples is performed as a function of $p_{\mathrm {T}}^\mathrm {jet}$. The dijet event selection further requires:

1. At least two jets are reconstructed with $p_{\mathrm {T}}^\mathrm {jet}>20 ~\mathrm{GeV }$, $|\eta |<1.2$ and $|\mathrm {JVF}|>0.75$.
2. The two leading (in $p_{\mathrm {T}}$) jets are $b$-tagged with the MV1 algorithm ($w_{\text {{MV1}}}>0.6$).
3. At least one of the jets with a muon candidate within $\Delta R<0.4$ passes the selection described in Ref. [91].
4. No third-leading jet reconstructed in the event with $|\mathrm {JVF}|>0.6$ and $p_{\mathrm {T}}^\mathrm {jet}> \max (12~\mathrm{GeV }, 0.25 \cdot p_{\mathrm {T}}^\mathrm {avg})$.
5. The azimuthal distance between the two leading jets is $\Delta \phi _{jj}$ $> 2.5$.

The selection on the inclusive samples requires at least one jet with $p_{\mathrm {T}}^\mathrm {jet}>25$  GeV and $|\eta |<2.5$, and the $|\mathrm {JVF}|>0.75$ cut. The muon selection is unchanged and different $b$-tagging algorithms and operating points are studied, since the neutrino energy is expected to be largely independent of the tagging algorithm, while JES is not.

The *b*-jet purity of these samples is measured with MC simulations to vary from 50 to 70 % for the inclusive selection, 60–80 % for the semileptonic selection, and to be above 80 % for the dijet selection for the operating points studied. Observations at high $p_{\mathrm {T}}\gtrsim 200$  GeV suggest that the purity might be underestimated by as much as 10 % [96]. Uncertainties on the efficiency of the tagging algorithm to identify *b*-jets and *c*-jets can also impact these purity estimates by up to about 10 % [91]. Despite these systematic effects, the purity of these samples remains sufficiently large for the validation purposes of this study.

#### Top-quark pair sample selection

Top-quark pair events where one of the $W$ bosons produced by the top-quark decays to an electron or a muon are selected by the following requirements (see Ref. [45] for further details)

1. A single-lepton trigger is present.
2. Exactly one electron with transverse energy above $25\,~\mathrm{GeV }$, within pseudorapidity range of $|\eta |$ less than 2.47, and outside the region of transition between the barrel and the endcap calorimeters, $1.37 \le |\eta | < 1.52$ is reconstructed; or, exactly one muon with transverse momentum above $20 ~\mathrm{GeV }$ is reconstructed within $|\eta |<2.5$. The reconstructed charged lepton has to match the trigger object corresponding to the required triggers that passed.
3. For the $t\bar{t}\rightarrow \text{ e+jets }$ channel the transverse $W$ boson mass $m_{\text {T}}$($W$), reconstructed from the electron and $E_{\mathrm {T}}^{\mathrm {miss}}$, should be $m_{\text {T}}(W)> 25~\mathrm{GeV }$, with $E_{\mathrm {T}}^{\mathrm {miss}}>35$  GeV. Alternatively, for the $t\bar{t}\rightarrow \mu \text{+jets }$ channel, $E_{\mathrm {T}}^{\mathrm {miss}}>25$  GeV and $E_{\mathrm {T}}^{\mathrm {miss}}+m_{\text {T}}(W)>60$  GeV are required.
4. At least four jets with $p_{\mathrm {T}}^\mathrm {jet}> 25~\mathrm{GeV }$, $|\mathrm {JVF}|>0.75$, and $|\eta |<2.5$ are required. Among these, at least two jets should be $b$-tagged using the MV1 $b$-tagging algorithm ($w_{\text {{MV1}}}>0.6$).

After this selection the background contamination in the $t\bar{t}$ sample is expected to be of order 10 % and to mainly consist of events from $W$/$Z$+jets and single top-quark production. The contribution from multijet background after the requirement of two $b$-tagged jets is expected to be about 4 %. The background contamination in the selected data sample has no sizable impact in the studies performed, and it is considered as an additional systematic uncertainty.

### MC-based systematic uncertainties on the calorimeter *b*-jet energy scale

The uncertainties on the *b*-jet transverse momentum measurement are studied using systematic variations in the MC simulation. The *b*-jet can be either reconstructed using a calibration with respect to all stable particles to study the all-particle energy scale, or excluding muons and neutrinos to study the calorimeter energy scale, as described in Sect. 5. The former definition is currently most relevant for $b$-tagging calibration analyses [91], and further discussed in Sect. 19.8.

The uncertainty in the calorimeter response to *b*-jets can be estimated using a combination of different MC simulations as reported in Ref. [3]. Figure 53a shows the calorimeter response to *b*-jets for various MC simulations.

The corresponding systematic uncertainties associated with the *b*-jet energy measurement are shown in Fig. 53b. These uncertainties need to be considered in addition to those established for an inclusive jet sample, since *b*-jet specific effects are not taken into account in that analysis. These uncertainties can be applied to any sample of *b*-jets, whether a specific analysis uses tagging or not, and are of a size comparable to the uncertainties in the in situ measurements presented later in this paper.

Two key changes are made in this analysis with respect to what is reported in Ref. [3]. The dead material uncertainty, which is large in Fig. 53a, but does not contribute significantly to the systematic uncertainty reported in Fig. 53b, is calculated as an additional change in the response expected from dead material effects for a *b*-jet sample with respect to an inclusive sample (or a pure light-quark sample for comparable results). This is possible in 2011 because in situ jet energy scale corrections and uncertainties exist which are already accounting for a potential mis-modelling of the dead material in the MC simulation. The uncertainty component derived from the propagation of single-particle uncertainties to jets is also removed, while it contributes 0.5 % in 2010 data. This result relies again on in situ studies, since differences in the calorimeter response between data and MC simulations are already taken into account in those studies. Residual effects that could give rise to an additional systematic uncertainty component for *b*-jets are constrained using a single-particle evaluation and are shown in Sect. 21.

**Fig. 54:**
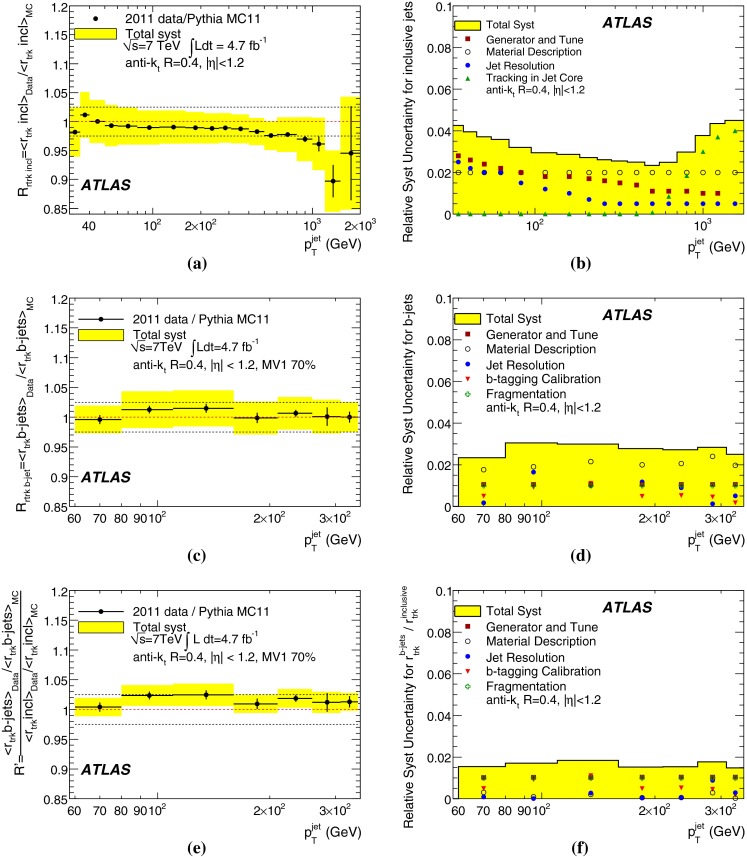
Ratio of the average $r_\mathrm{trk}$ given in Eq. (19) in data and MC simulations for **a** inclusive jets and **c** tagged *b*-jets. In **e**, the $b$-tagged to inclusive sample ratio variable $R'$ from Eq. (21) is shown. The contributions of the systematic uncertainties to the total uncertainty in the different measurements are shown in **b**, **d**, and **f**, respectively. Jets within $|\eta |<1.2$ are used

**Fig. 55:**
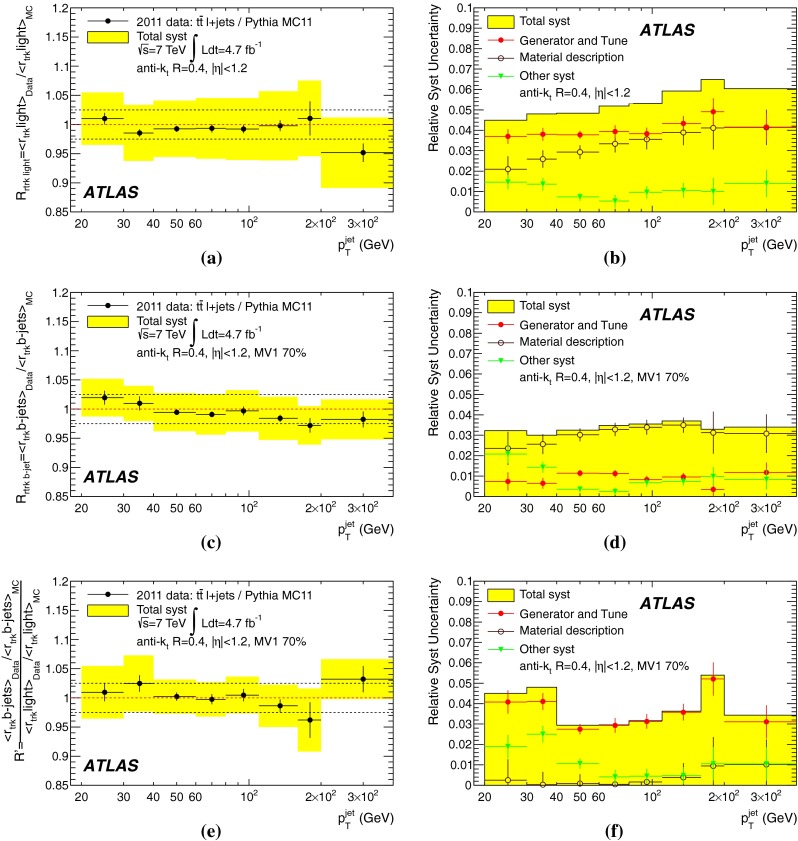
Ratio of the average $r_\mathrm{trk}$ given in Eq. (19) in $t\bar{t}$ events in data and MC simulations for **a** light-jets and tagged **c** *b*-jets. In **e**, the ratio of $R_{r_\mathrm{trk}}$ from Eq. (20) between the *b*-jet and the light-jet sample is shown. The total systematic uncertainty is shown as a *band*, and the *dotted lines* correspond to unity and the 2.5 % deviation from unity. The contributions of the systematic uncertainties to the total uncertainty in the different measurements are shown in **b**, **d**, and **f**, respectively. The contributions to the total systematic uncertainty due to the jet resolution, $b$-tagging calibration, background contamination and the modelling of the initial- and final-state radiation are grouped under “Other systematics”. Jets with $|\eta |<1.2$ are used

### Calorimeter jet energy measurement validation using tracks

The calorimeter jet energy scale can be probed by comparing the measured jet energy to that of a well-calibrated reference object with independent systematic uncertainties. Charged-particle tracks are well measured with uncertainties independent of the calorimeter, and can be associated with jets, are used here. The mean value of $r_\mathrm{trk}$, defined in Eq. (19) is primarily sensitive to the particle composition of the jet and thus should be well described by any well-tuned event generator. In computing $\langle r_\mathrm{trk}\rangle$ it is important to truncate the $r_\mathrm{trk}$ distribution (here with $r_\mathrm{trk}<3$) to avoid contributions from fake tracks with unphysically large $p_{\mathrm {T}}$.

**Fig. 56:**
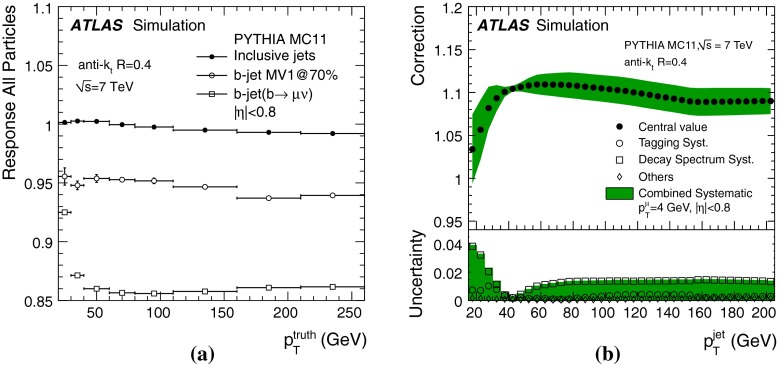
Average jet response as a function of true transverse momentum of jets built using all stable particles, for a sample of inclusive jets (*solid circles*), a sample of *b*-jets tagged with the MV1 tagging algorithm (*open circles*) and a sample of semileptonically decaying *b*-jets with a reconstructed muon inside (*open squares*), is shown in **a**. The resulting semileptonic correction, as a function of calorimeter jet $p_{\mathrm {T}}$, used to transform the $p_{\mathrm {T}}$ of a jet in the semileptonic sample to the $p_{\mathrm {T}}$ of a jet in an inclusive sample of *b*-jets, is displayed in **b**. Associated systematic uncertainties are shown around the central value, and the combined uncertainty is shown as a *coloured band*

To verify the description of the calorimeter energy measurement in MC simulations, the double ratio of the charged-to-total momentum obtained in data to that obtained in Monte Carlo simulation is studied:

20 $$\begin{aligned} R_\mathrm{r_\mathrm{trk}} \equiv \frac{\langle {r_\mathrm{trk}}\rangle _\mathrm{Data} }{\langle {r_\mathrm{trk}}\rangle _\mathrm{MC}}. \end{aligned}$$

The ratio is evaluated for inclusive jets ($R_\mathrm{r_\mathrm{trk}, inclusive}$), $b$-tagged jets ($R_{\mathrm{r_\mathrm{trk},}\text {\textit{b}-jet}}$) and $b$-tagged jets with a reconstructed muon inside ($R^{\mu \nu }_{r_\mathrm{trk},\text {\textit{b}-jet}}$, in the dijet sample only). The calorimeter response ratio $R'$ of $b$-tagged jets relative to inclusive jets is then defined using Eq. (20) from each respective sample,

21 $$\begin{aligned} R' \equiv \frac{R_{r_\mathrm{trk},\text {\textit{b}-jet}}}{R_\mathrm{r_\mathrm{trk}, inclusive}} . \end{aligned}$$

This ratio is used to test the relative systematic uncertainty between $b$-tagged and inclusive jets. In the $t\bar{t}$ sample, where the fraction of *b*-jets is large ($\approx$50 %), the light jets (non $b$-tagged) component is used in the denominator instead of the inclusive one. It is mainly comprised of jets from the $W$ boson decay but also to a lesser extent of gluon jets from initial- and final-state radiation. As a consequence, when comparing the results obtained in the $t\bar{t}$ and the dijet analyses, the difference in terms of jet flavour components entering the calculation of $R_\mathrm{r_\mathrm{trk}, inclusive}$ needs to be taken into consideration.

### Systematic uncertainties

Systematic uncertainties in the $r_\mathrm{trk}$ measurement arise from the modelling of the jet (and *b*-jet) fragmentation, $b$-tagging calibration, jet resolution and track reconstruction efficiency. In addition, for high-$p_{\mathrm {T}}$ jets ($p_{\mathrm {T}}>500$  GeV) an efficiency loss in the tracking in the jet core is observed in MC simulations, and a systematic uncertainty is added to account for potential mis-modelling of this effect. These uncertainties are assumed to be uncorrelated. The resulting fractional systematic uncertainties on $r_\mathrm{trk}$ and $R'$ are shown in Fig. 54b, d, f for the inclusive jet sample, and in Fig. 55b, d, f for the $t\bar{t}$ sample. They are determined as follows.

1. **MC generator and tunes** These systematic uncertainties capture the effects of differences in $p_{\mathrm {T}}^\mathrm{track}$ caused by different fragmentation models. Differences in the calorimeter response, caused by the different particle spectra, can also impact the $r_\mathrm{trk}$ measurement in certain MC simulations and should not be part of the uncertainty, since such shifts are measurable in the data. The $r_\mathrm{trk}$ distribution is, thus, calculated from the various samples described in Sect. 3 using $p_{\mathrm {T}}^\mathrm{truth}$ in the denominator, even though only small differences are observed in most samples when including calorimeter effects, i.e. using the jet $p_{\mathrm {T}}$ reconstructed with the calorimeters ($p_{\mathrm {T}}^\mathrm{calo}$). In the top pair analysis, differences between MC@NLO and POWHEG+Herwig are considered as process or generator systematic uncertainties. Fragmentation and decay systematic uncertainties are evaluated taking the difference between Pythia and Herwig. In the dijet analysis, differences between Pythia and Herwig++ set the systematic uncertainties from uncertainties in the decay models. The updated fragmentation tune in Herwig++ prevents this comparison from being a conservative measure of the *b*-jet fragmentation systematic uncertainties. These are evaluated using comparisons to the Bowler–Lund [97] and Professor tunes [98].
2. ***b*** **-tagging calibration** The scale factors that correct the $b$-tagging efficiencies in MC simulations to match the measured values are varied within their total uncertainty.
3. **Material description** The knowledge of the tracking efficiency modelling in MC simulations is evaluated in detail in Ref. [99]. The systematic uncertainty on the tracking efficiency for isolated tracks increases from 2 % ($|\eta ^\mathrm{track}| < 1.3$) to 7 % ($2.3 \le |\eta ^\mathrm{track}| <2.5$) for tracks with $p_{\mathrm {T}}> 500$  MeV. The resulting effect on $r_\mathrm{trk}$ is about 3 % for $0 \le |\eta \,| < 2.1$ and about 4 % for $2.1 \le |\eta \,| < 2.5$.
4. **Tracking in jet core** High track densities in the jet core influence the tracking efficiency due to shared hits between tracks, fake tracks and lost tracks. The number of shared hits is well described in the MC simulation. The $p_{\mathrm {T}}$ carried by fake tracks is negligible. A relative systematic uncertainty of 50 % on the loss of efficiency obtained in the simulation is assigned to account for potential mis-modelling of this effect.
5. **Jet energy resolution** The jet energy resolution in MC simulations is degraded by about 10 %.
6. **Background contamination** For the $t\bar{t}$ sample the analysis is repeated including the expected background contamination (except the multijet contribution) and the full difference is taken as an estimate of the systematic uncertainty.

**Fig. 57:**
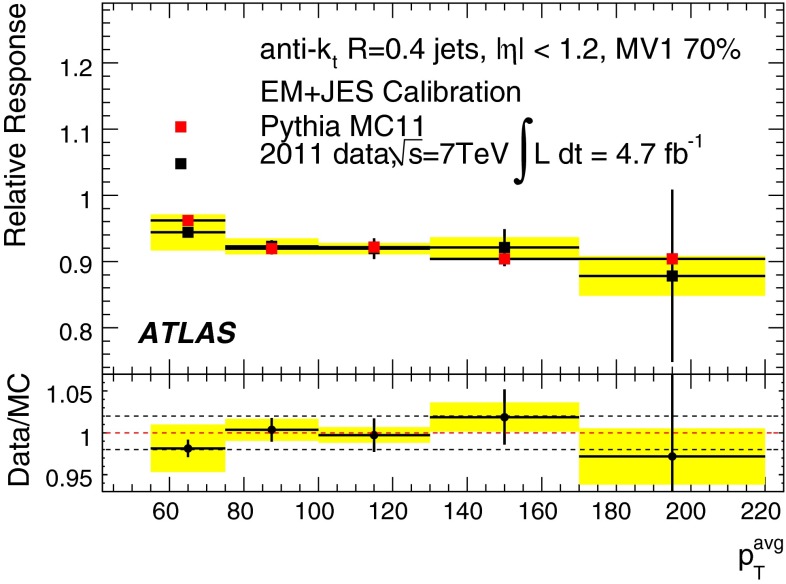
Relative response of the semileptonic sample with respect to the inclusive *b*-jet sample as calculated from the dijet $p_{\mathrm {T}}$ asymmetry. The uncertainty band around the data denotes systematic uncertainties in the asymmetry measurement

**Fig. 58:**
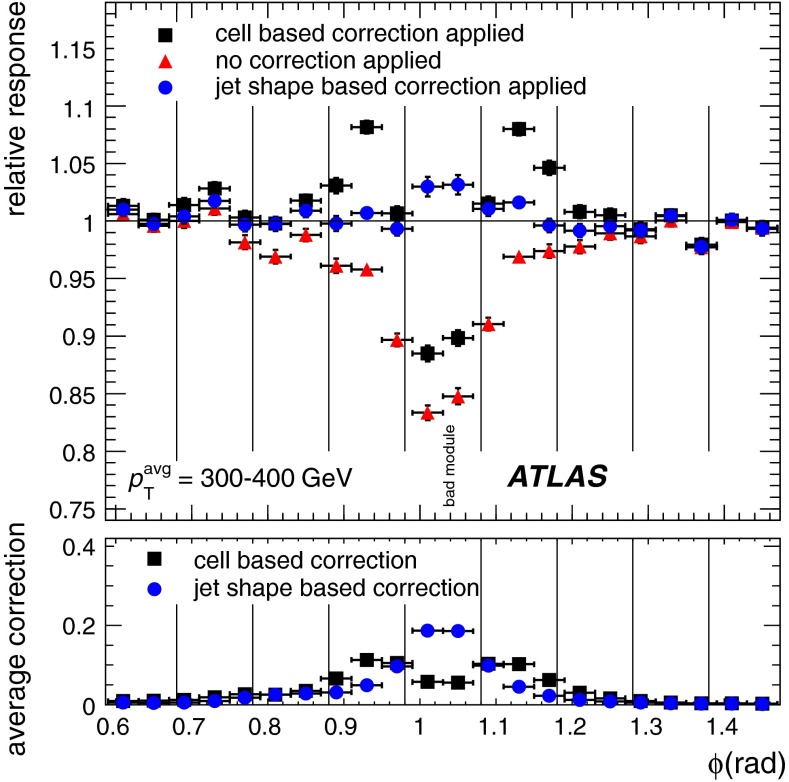
Average relative response of the probe jets with respect to the tag jets as a function of various impact points in the azimuthal direction ($\phi$) of the probe jet. The average $p_{\mathrm {T}}$ of the two leading jets is $300 \le p_{\mathrm {T}}^\mathrm {avg}< 400$ GeV. The *vertical solid lines* indicate the location of the Tile calorimeter modules. The non-operating Tile calorimeter module is at $\phi = 1.03$. The markers indicate the results for no correction (*triangles*), the cell-based corrections (*squares*) and the corrections based on the jet shape (*circles*). The *lower* part of the figure shows the respective average values of the two corrections as a function of the azimuthal angle of the probe jet

The dominant contributions to the systematic uncertainty in the $t\bar{t}$ analysis are due to variations in the detector material and fragmentation/decay models. In the dijet sample, the material, fragmentation and decay uncertainties also dominate the systematic uncertainties, except at $p_{\mathrm {T}}\gtrsim 500$  GeV where the uncertainty caused by the loss of efficiency in the jet core dominates. In Fig. 55, the contributions to the total systematic uncertainty due to the jet resolution, $b$-tagging calibration, background contamination and due to the modelling of the initial- and final-state radiation are labelled as “other” systematic uncertainties.

For $R'$, the tracking components (the material description, impacting the tracking efficiency) of the systematic uncertainty entering both the numerator and denominator are correlated and thus approximately cancel. A similar consideration holds for the jet energy resolution. The most significant systematic uncertainties on $R'$ are due to the choice of the MC generator and the fragmentation and decay models.

### Results

Figure 54a, c show the ratio of the average of the $r_\mathrm{trk}$ distribution in data and MC simulations for jets in the inclusive jet sample with $|\eta |< 1.2$. Figure 54b, d show the different components of the associated systematic uncertainty, as discussed in Sect. 19.6.

The study in the sample without $b$-tagging covers up to approximately 2 TeV, and provides a cross check over almost the full range of calibrated $p_{\mathrm {T}}$ studied in situ through the analyses used to establish the systematic uncertainty on the jet energy scale in ATLAS. No $p_{\mathrm {T}}$ dependence is observed and agreement is found between data and MC simulations within systematic uncertainties. Similar results are found in higher $|\eta |$ regions.

**Fig. 59:**
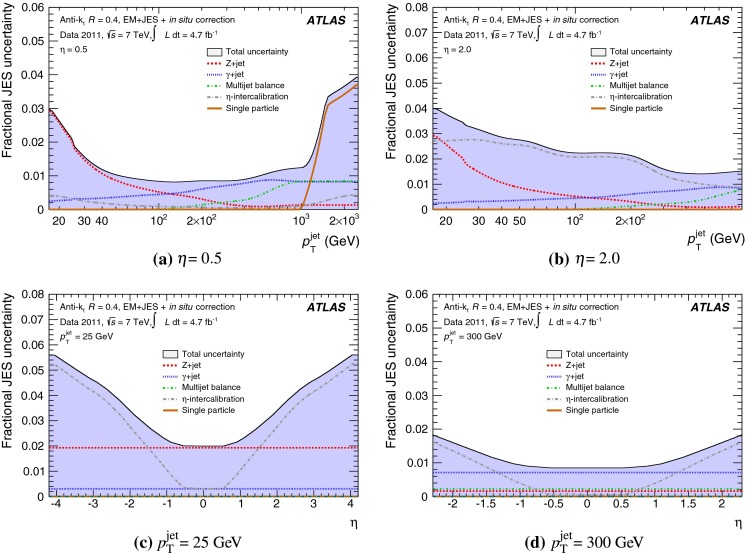
Fractional in situ jet energy scale systematic uncertainty as a function of **a**, **b** $p_{\mathrm {T}}^\mathrm {jet}$ and **c**, **d** jet pseudorapidity for anti-$k_{t}$ jets with distance parameter of $R=0.4$ calibrated using the EM+JES calibration scheme. The contributions from each in situ method are shown separately. Uncertainties from pile-up, flavour, and topology are not included

Agreement of the MC simulations with the data for the $r_\mathrm{trk}$ measurements is found within systematic uncertainties across all $p_{\mathrm {T}}$ for inclusive jets and for $p_{\mathrm {T}}^\mathrm {jet}< 400$  GeVfor $b$-tagged jets. The relative response $R'$ between $b$-tagged and inclusive jets is shown in Fig. 54e and the uncertainty band corresponds to the relative *b*-jet energy scale uncertainty with respect to the inclusive jet sample. Figure 54f shows the different components of the associated systematic uncertainty. A difference between data and MC simulations is found but almost covered by the systematic uncertainties. This difference is partially caused by the overall 1 % shift found in the inclusive sample. Similar results are found in the sample of *b*-jets decaying to muons selected in the dijet sample, with a larger difference between data and MC simulations of up to 4 % in the lowest $p_{\mathrm {T}}$ bin probed. However, the uncertainties in the modelling are also somewhat larger, limiting the constraints on the jet energy scale of these jets to approximately 3 %.

The corresponding results from the same study performed in the $t\bar{t}$ sample are shown in Fig. 55.

The results in this sample are consistent with those obtained in the dijet sample, except for the better agreement between data and MC simulations in the light-jet sample, which also leads to better agreement in the *b*-jet to light-jet sample results. The systematic uncertainties are also comparable, despite the different methods used in their evaluation. The uncertainty in the in situ technique used to assess the *b*-jet energy scale is estimated to be approximately 2.5 and 3 % in the ranges $|\eta \,| < 1.2$ and $1.2 \le |\eta \,| < 2.5$, respectively, for jets with $p_{\mathrm {T}}^\mathrm {jet}< 400$  GeV from these studies.

**Fig. 60:**
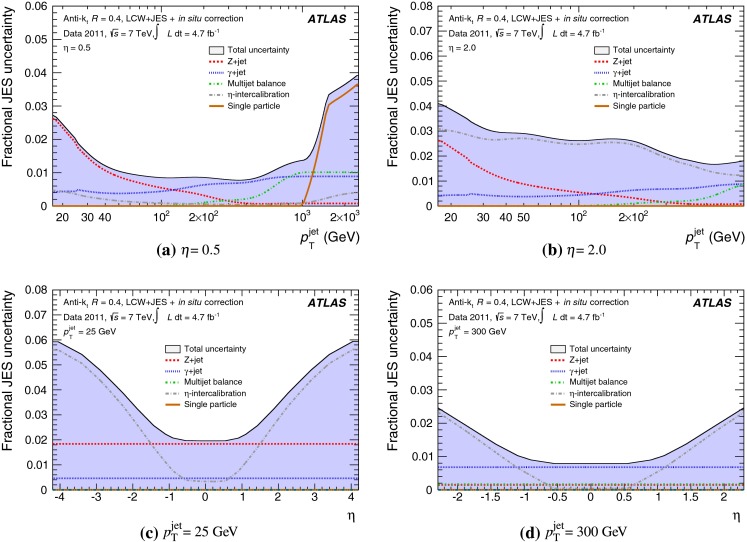
Fractional in situ jet energy scale systematic uncertainty as a function of **a**, **b** $p_{\mathrm {T}}^\mathrm {jet}$ and **c**, **d** jet pseudorapidity for anti-$k_{t}$ jets with distance parameter of $R=0.4$ calibrated using the LCW+JES calibration scheme. The contributions from each in situ method are shown separately. Uncertainties from pile-up, flavour, and topology are not included

**Table 14:** Summary of the in situ EM+JES and LCW+JES jet energy scale systematic uncertainties for different $p_{\mathrm {T}}^\mathrm {jet}$ and $|\eta |$ values for anti-$k_{t}$ jets with $R=0.4$ and $R=0.6$. These values do not include pile-up, flavour or topology uncertainties

$|\eta |$ region	$p_{\mathrm {T}}^\mathrm {jet}=20$ GeV (%)	$p_{\mathrm {T}}^\mathrm {jet}=40$ GeV (%)	$p_{\mathrm {T}}^\mathrm {jet}=200$ GeV (%)	$p_{\mathrm {T}}^\mathrm {jet}=800$ GeV (%)	$p_{\mathrm {T}}^\mathrm {jet}=1.5$ TeV (%)
Fractional EM+JES JES uncertainty for $R=0.4$					
$|\eta |=0.1$	2.6	1.2	0.8	1.3	3.2
$|\eta |=0.5$	2.6	1.2	0.8	1.3	3.2
$|\eta |=1.0$	2.8	1.4	1.0	1.3	3.2
$|\eta |=1.5$	3.2	2.0	1.5	1.4	3.3
$|\eta |=2.0$	3.8	2.9	2.1	1.6	
$|\eta |=2.5$	4.3	3.8	2.8		
$|\eta |=3.0$	4.7	4.5	3.4		
$|\eta |=3.5$	5.1	4.9	4.6		
$|\eta |=4.0$	5.7	5.1	4.9		
Fractional LCW+JES JES uncertainty for $R=0.4$					
$|\eta |=0.1$	2.4	1.2	0.8	1.3	3.2
$|\eta |=0.5$	2.5	1.2	0.8	1.3	3.2
$|\eta |=1.0$	2.6	1.4	1.1	1.3	3.2
$|\eta |=1.5$	3.1	2.1	1.7	1.4	3.3
$|\eta |=2.0$	3.9	2.9	2.6	1.8	
$|\eta |=2.5$	4.6	3.9	3.4		
$|\eta |=3.0$	5.2	4.6	3.9		
$|\eta |=3.5$	5.8	5.2	4.5		
$|\eta |=4.0$	6.2	5.5	5.1		
Fractional EM+JES JES uncertainty for $R=0.6$					
$|\eta |=0.1$	2.7	1.4	0.8	1.8	3.3
$|\eta |=0.5$	2.7	1.5	0.8	1.8	3.3
$|\eta |=1.0$	2.8	1.6	0.9	1.8	3.3
$|\eta |=1.5$	3.0	1.9	1.3	1.9	3.3
$|\eta |=2.0$	3.6	2.6	1.9	2.0	
$|\eta |=2.5$	4.3	3.4	2.4		
$|\eta |=3.0$	5.2	4.1	3.0		
$|\eta |=3.5$	5.7	4.7	3.8		
$|\eta |=4.0$	5.9	4.8	4.6		
Fractional LCW+JES JES uncertainty for $R=0.6$					
$|\eta |=0.1$	2.3	1.3	0.8	1.6	3.2
$|\eta |=0.5$	2.3	1.3	0.8	1.6	3.2
$|\eta |=1.0$	2.4	1.4	1.0	1.6	3.2
$|\eta |=1.5$	2.7	1.8	1.6	1.7	3.2
$|\eta |=2.0$	3.3	2.4	2.2	1.9	
$|\eta |=2.5$	4.4	3.3	2.8		
$|\eta |=3.0$	6.0	4.6	3.3		
$|\eta |=3.5$	7.0	5.6	3.8		
$|\eta |=4.0$	7.2	6.0	4.7		

**Fig. 61:**
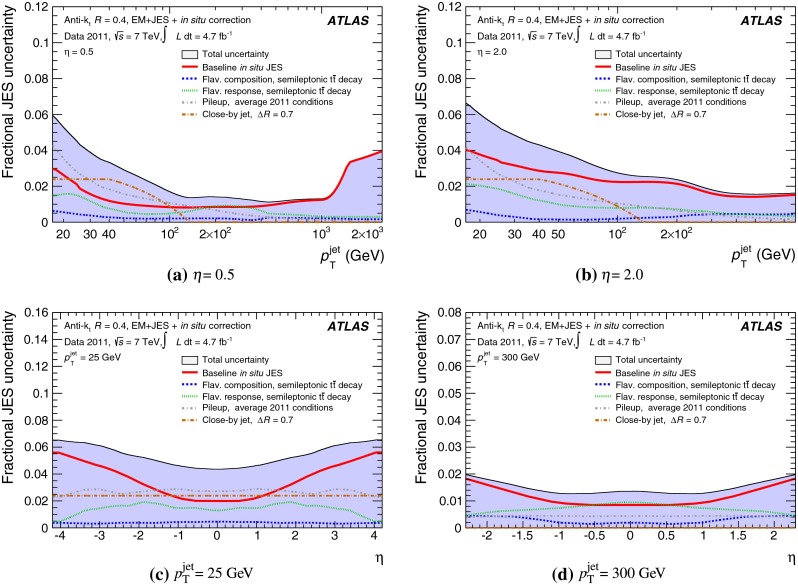
Sample-dependent fractional jet energy scale systematic uncertainty as a function of **a**, **b** $p_{\mathrm {T}}^\mathrm {jet}$ and **c**, **d** jet pseudorapidity for anti-$k_{t}$ jets with distance parameter of $R=0.4$ calibrated using the EM+JES calibration scheme. The uncertainty shown applies to semileptonic top-decays with average 2011 pile-up conditions, and does not include the uncertainty on the jet energy scale of *b*-jets

**Fig. 62:**
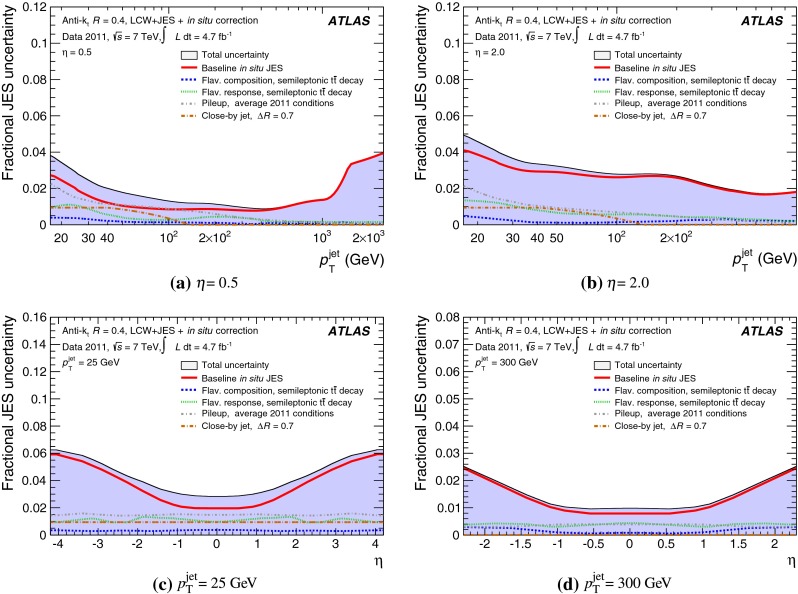
Sample-dependent fractional jet energy scale systematic uncertainty as a function of **a**, **b** $p_{\mathrm {T}}^\mathrm {jet}$ and **c**, **d** jet pseudorapidity for anti-$k_{t}$ jets with distance parameter of $R=0.4$ calibrated using the LCW+JES calibration scheme. The uncertainty shown applies to semileptonic top-decays with average 2011 pile-up conditions, and does not include the uncertainty on the jet energy scale of *b*-jets

**Fig. 63:**
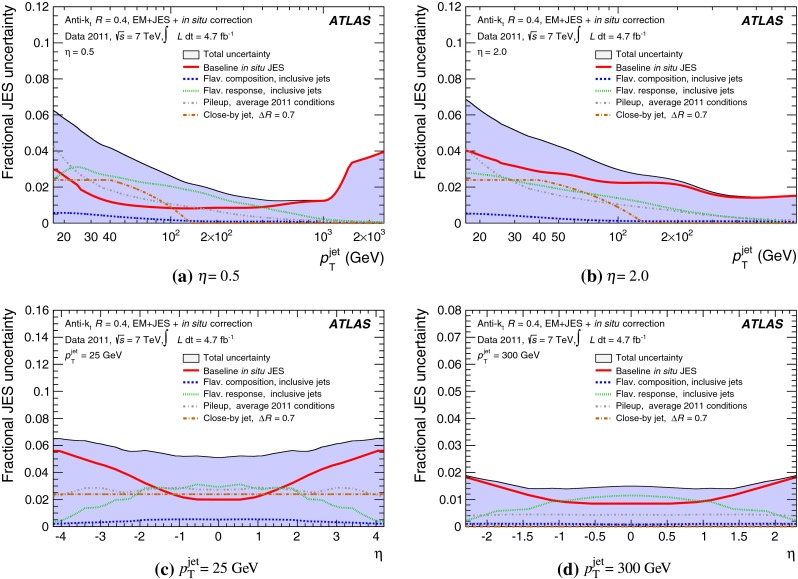
Sample-dependent fractional jet energy scale systematic uncertainty as a function of **a**, **b** $p_{\mathrm {T}}^\mathrm {jet}$ and **c**, **d** jet pseudorapidity for anti-$k_{t}$ jets with distance parameter of $R=0.4$ calibrated using the EM+JES calibration scheme. The uncertainty shown applies to inclusive QCD jets with average 2011 pile-up conditions, and does not include the uncertainty on the jet energy scale of *b*-jets

**Fig. 64:**
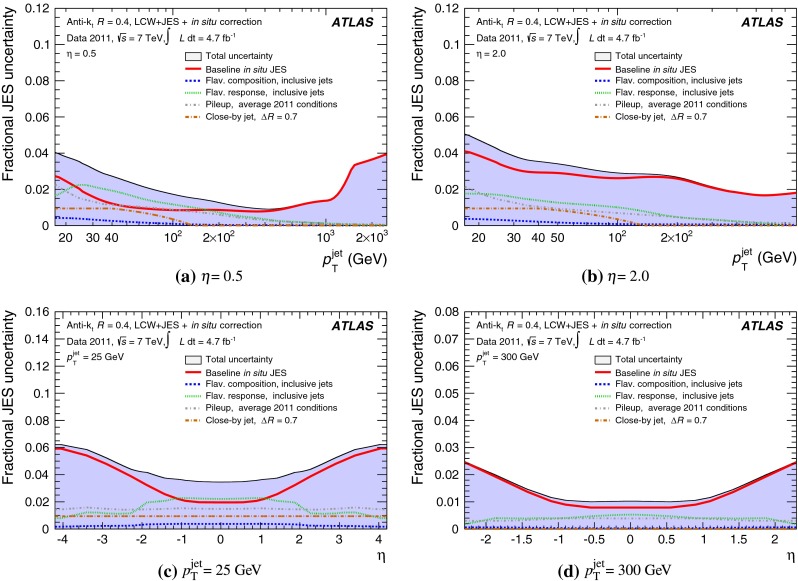
Sample-dependent fractional jet energy scale systematic uncertainty as a function of **a**, **b** $p_{\mathrm {T}}^\mathrm {jet}$ and **c**, **d** jet pseudorapidity for anti-$k_{t}$ jets with distance parameter of $R=0.4$ calibrated using the LCW+JES calibration scheme. The uncertainty shown applies to inclusive QCD jets with average 2011 pile-up conditions, and does not include the uncertainty on the jet energy scale of *b*-jets

**Fig. 65:**
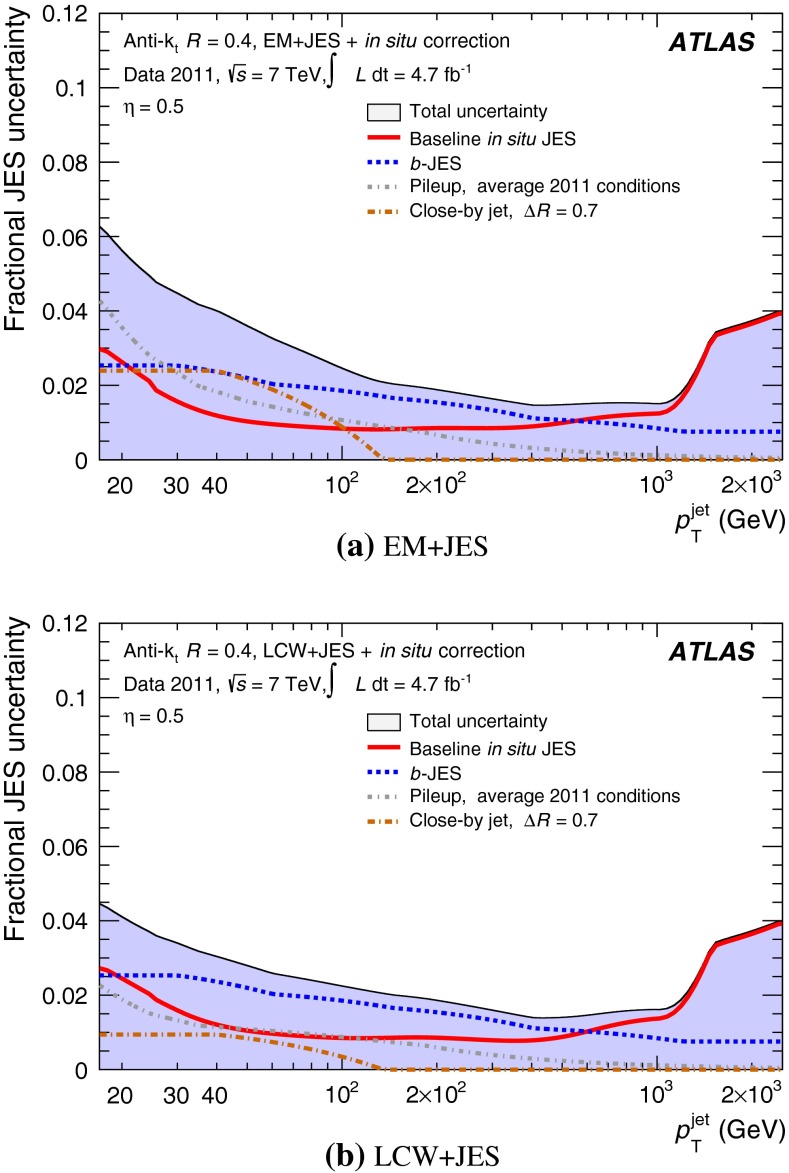
Fractional jet energy scale systematic uncertainty as a function of $p_{\mathrm {T}}^\mathrm {jet}$ for anti-$k_{t}$ jets with distance parameter of $R=0.4$ calibrated using the **a** EM+JES and **b** LCW+JES calibration schemes. The uncertainty shown applies to *b*-jets with average 2011 pile-up conditions

### Semileptonic correction and associated uncertainties

The study of the all-particle response $\mathcal {R}^\mathrm{all}$ of *b*-jets, i.e. the energy scale calculated with respect to jets built using all stable particles, is also necessary for many analyses, given that about 40 % of *b*-jets decay semileptonically, thus having a non-negligible amount of their energy carried by neutrinos. In particular, the study of the $b$-tagging efficiency in a sample of *b*-jets decaying semileptonically to muons [91] requires a correction that maps the all-particle jet energy scale of that sample to that of an inclusive sample of *b*-jets. This correction and its systematic uncertainties are estimated in this section. The correction also has applications beyond the $b$-tagging calibration since it can also be used to improve the reconstruction of *b*-jets identified as semileptonic. The study of the all-particle energy scale in this section is performed independently of the study of the calorimeter energy scale, even though the two are not straightforward to decouple in in situ studies.

Figure 56a shows the all-particle response for an inclusive jet sample, a sample of *b*-jets tagged with the MV1 algorithm and a sample of *b*-jets containing a muon from a semileptonic $b$ decay. The semileptonic *b*-jets sample is selected using hadron-level information, and no $b$-tagging is imposed. However, the muon is required to pass kinematic and quality cuts detailed in Ref. [91]. The effect of neutrinos is clearly visible in both the tagged *b*-jets sample and more significantly in the semileptonic *b*-jets sample. The increase at low $p_{\mathrm {T}}$ in the semileptonic sample arises from biases created by the muon kinematic cuts.

The response of semileptonically decaying *b*-jets is corrected to that of an inclusive $b$-tagged jet sample. The correction is constructed using techniques similar to those used in the EM+JES calibration introduced in Sect. 5. This correction is shown in Fig. 56b, as a function of calibrated jet $p_{\mathrm {T}}$ for fixed muon $p_{\mathrm {T}}$ and jets with $|\eta |<0.8$. The correction is not explicitly dependent on $p_{\mathrm {T}}^{\mu }$ even though it enters in the calculation of the reconstructed jet $p_{\mathrm {T}}$ used to compute the correction.

Systematic uncertainties in this correction need to account for our knowledge of *b*-jet fragmentation and decay, as well as the effect of the muon spectrum and muon reconstruction. These uncertainties are presented in Ref. [91]. Since only one correction is calculated and used for all tagging algorithms and operating points commissioned up to date, an additional systematic uncertainty that covers the spread of the corrections for all these different operating points is added. All uncertainties are combined in quadrature. Only the most significant uncertainties are included in Fig. 56b, namely the uncertainty that arises from the different correction for different operating points, and the uncertainty that arises from the limitations in the knowledge of the muon momentum spectrum in the centre-of-mass energy of the decaying hadron. These uncertainties are estimated by reweighting that spectrum to match a measurement obtained in $e^+e^-$ scattering [100]. Due to the significant differences between that spectrum and the one found in Pythia, these variations are considered sufficient. All other uncertainties are combined and shown in the figure under the same curve.

The uncertainty is about 1.5 % for most $p_{\mathrm {T}}$ values in the central region, except at low $p_{\mathrm {T}}$ where it increases to about 4 %. The behaviour is similar at larger $\eta$, except in the most forward bin ($2.1<|\eta |<2.5$), where variations across tagging operating points cause the uncertainty to increase to about 2 %.

### Semileptonic neutrino energy validation using dijet balance

The modelling of the energy carried by the neutrino in the inclusive *b*-jet sample and in the semileptonic *b*-jet sample can be validated using the $p_{\mathrm {T}}$ balance of a dijet system. The same technique is used in Ref. [3] to validate the variation of the calorimeter response as a function of different jet properties. The response in data is calculated using the asymmetry in the jet $p_{\mathrm {T}}$ of the two jets in the dijet system. The two jets are required to be $b$-tagged, and the probe jet is required to have a selected reconstructed muon within $\Delta R< 0.4$. The relative response, calculated from the asymmetry, is sensitive to the energy carried by the neutrino, but also to the response differences between the $b$-tagged and semileptonic *b*-jet samples. These differences, however, are well modelled in the MC simulation, as shown in Sect. 19.7.

Figure 57 shows the relative response of semileptonic *b*-jets with respect to inclusive *b*-jets obtained in data and MC simulations using dijet balance.

The presence of neutrinos in the *b*-jet decay causes the estimated relative response to be below 1. The uncertainty band around the data represents systematic uncertainties in the imbalance. These are calculated through variations in the soft-radiation cut in the selection (i.e. the $p_{\mathrm {T}}$ used for the veto on the third leading jet) as Sect. 8.4. An additional contribution to the uncertainty is added to the first $p_{\mathrm {T}}$ bin to account for differences between data and MC simulations in the turn-on of the efficiency curve for the muon-jet trigger used in this analysis. Agreement is found between data and MC simulations, validating the description of this process that is exploited to develop the semileptonic correction presented in the previous Sect. 19.8.

### Conclusions on heavy-flavour jets

The uncertainty on the jet energy measurement is studied for light jets as well as inclusive and semileptonic *b*-jets. In the inclusive jet sample the jet energy scale is probed using tracks associated with jets over a wide range of jet $p_{\mathrm {T}}$. Comparisons between data and MC simulations show agreement within systematic uncertainties of approximately 3 % with weak dependence on the transverse momentum of the jets. The *b*-jet energy scale is also probed using tracks associated with $b$-tagged jets in the data. The results in the $t\bar{t}\rightarrow \textit{l}\text{+jets }$ and inclusive jet samples suggest that the jet energy scale of *b*-jets is well described by the MC simulation, within systematic uncertainties of about 2–3 %.

In the MC simulation a correction for semileptonic *b*-jets decaying to muons is derived, which adjusts the transverse momentum measurement to that in an inclusive sample of *b*-jets. The systematic uncertainties on this correction are also derived using MC simulations. They are found to be about 2 %. The uncertainty in the jet energy measurement due to effects specific to *b*-jets is also determined using Monte Carlo simulations. This uncertainty ranges from 1 to 3 %.

The energy scale of semileptonic *b*-jets decaying to muons is probed in the dijet sample in parallel with a study of the energy carried by the accompanying neutrino. The latter confirms the results found in MC simulations within systematic uncertainties of about 3 %.

## Jet response in problematic calorimeter regions

At the end of the 2011 data taking period 11 out of 256 modules of the ATLAS central hadronic Tile calorimeter were not operational. Moreover, during the data taking, some Tile calorimeter modules occasionally became non-operational for short periods of time, e.g. due to trips of the high voltage. In this section the impact of non-operating Tile modules on the jet energy measured is studied using a tag-and-probe technique based in the $p_{\mathrm {T}}$ balance of the two leading jets in the event following Sect. 8.1.1. The response of the tag jet, required to be in a fully operational part of the calorimeter, is used to test the response of a probe jet that impinges close to and in the region of the non-operating Tile module.

The performance of two reconstruction algorithms that correct for non-operating parts of the calorimeters based on the energy deposits in nearby cells or the average transverse jet shape is assessed.

### Correction algorithms for non-operating calorimeter modules

#### Correction based on calorimeter cell energies

This correction is implemented in the standard ATLAS calorimeter energy reconstruction. It estimates the energy density of a non-operating Tile calorimeter cell on the basis of energy measured by the two neighbouring cells that belong to the same Tile calorimeter layer sub-detector as the non-operating cell. The energy density of the non-operating cell is estimated as the average (arithmetic mean) of the energy density of the neighbouring cells. This correction is called $\mathrm {BCH}_\mathrm{cor,cell}$ correction in the following.

#### Corrections based on jet shapes

This correction is applied after jet reconstruction. The expected average jet shape is used to estimate the energy deposited in the non-operating Tile calorimeter cells. The correction is derived from MC simulations where all calorimeter modules are operational. It is calculated as a function of the transverse momentum and the pseudorapidity of the jet, the calorimeter type, the calorimeter layer and the angular distance between the jet axis and the cell centre in the ($\eta ,\phi$) space ($\Delta R$ in Eq. (3) in Sect. 5.6). It is applied for both LAr and Tile calorimeter cells and is called $\mathrm {BCH}_\mathrm{cor,jet}$ in the following.

In predefined bins of $p_{\mathrm {T}}^\mathrm {jet}$ and $\eta _\mathrm{det}$ and for all the calorimeter cells that belong to the jets the average relative energy (defined as $E_\mathrm {cell} / E_\mathrm {jet}$) in each calorimeter type, layer and $\mathrm {d}R$ bin is calculated. For all non-operational calorimeter cell in a jet the following correction is calculated:

$$\begin{aligned} \mathrm {BCH}_\mathrm{cor,jet}{} = \sum _{\mathrm {bad\ cells}} \frac{E_{\mathrm {cell}}}{E_\mathrm {jet}} \end{aligned}$$

and the energy of the jet is corrected with:

$$\begin{aligned} E_{\mathrm {jet}}^{\mathrm {corrected}} = \frac{E_{\mathrm {jet}}^{\mathrm {uncorrected}}}{1 - \mathrm {BCH}_\mathrm{cor,jet}}. \end{aligned}$$

### Performance of the bad calorimeter region corrections

The performance of the correction methods can be assessed using a tag-and-probe technique in events with two jets with high transverse momentum. The dependence of the relative jet response between the tag and the probe jets is studied as a function of the azimuthal angle of the probe jet.

The tag jet is selected such that it hits a fully operating part of the ATLAS calorimeter and is inside a central $\eta$ region ($|\eta | < 1.6$). Jets in the gap between Tile Long Barrel and Tile Extended Barrel (i.e. jets with axes pointing to the region $0.8 \le \eta < 1.2$) are excluded. The probe jet is chosen such that its axis points to the vicinity of the non-operating Tile module. Only probe jets with $0.1 \le |\eta | <0.8$ are used.

Figure 58 shows the jet response of the probe jet in the region of a missing Tile module and in the neighbouring regions for events where the average jet $p_{\mathrm {T}}$ of the two leading jets is between 300 and 400 GeV. A decrease of the probe jet response by about 15 % is observed in the region with the non-operating Tile calorimeter module when no correction is applied. This reduces to only about 10 % for the cell-based correction. However, an overcorrection by about 10 % is observed in the vicinity of the region with the missing Tile module. The correction based on the jet shape performs much better. There is no overcorrection in the vicinity of the problematic module and the probe jet energy is compensated much better if the jet axis falls into the module. There is only a small overcorrection by a few percent in the vicinity of the non-operating module.

#### Conclusion on bad calorimeter regions

The corrections for missing Tile calorimeter modules show a good performance. The average jet response variations close to the missing calorimeter are evaluated with a tag-and-probe technique in data. The jet response variation is about 5–10 %. The correction using jet shape information shows a better performance than the correction simply averaging the energy deposition in the neighbouring calorimeter cells.

The Monte Carlo simulation includes the missing Tile calorimeter modules and describes the jet response variations in data. The remaining differences are included in the JES uncertainty derived from the in situ techniques.

## Summary of the total jet energy scale systematic uncertainty

Figures 59 and 60 show the fractional jet energy scale uncertainty from the in situ measurements as a function of $p_{\mathrm {T}}^\mathrm {jet}$ for four representative values of $\eta$, and as a function of $\eta$ for two representative values of $p_{\mathrm {T}}^\mathrm {jet}$. The total uncertainty is given by the absolute (JES) and the relative in situ calibration uncertainties added in quadrature. For jets in the central region it amounts to 3 % at $p_{\mathrm {T}}^\mathrm {jet}\approx 17$  GeV, falling to 2 % at $p_{\mathrm {T}}^\mathrm {jet}\approx 25$ GeV, and is below 1 % for $55 \le p_{\mathrm {T}}^\mathrm {jet}<500$  GeV. The uncertainty increases for forward jets ($|\eta |>1.2$) due to the uncertainty on the modelling of the parton radiation altering the dijet $p_{\mathrm {T}}$ balance in the $\eta$-intercalibration technique. For very forward low-$p_{\mathrm {T}}$ jets ($p_{\mathrm {T}}\approx 25$ GeV, $|\eta |\approx 4$), the uncertainty can be as large as 6 %. The in situ JES uncertainty is similar for the EM+JES and LCW+JES calibration schemes.

For jets with $p_{\mathrm {T}}^\mathrm {jet}> 1$ TeV the JES uncertainty is derived from single-hadron response measurements [4], given the large statistical error of the multijet balance technique beyond $p_{\mathrm {T}}^\mathrm {jet}> 1$ TeV. The uncertainties from the in situ techniques are kept fixed at $p_{\mathrm {T}}^\mathrm {jet}= 1$ TeV and subtracted in quadrature from the uncertainty of the single-hadron response measurements, which is the dominant contribution at high $p_{\mathrm {T}}^\mathrm {jet}$ in 2010 and 2011.

Table 14 presents a summary of the total in situ JES uncertainties in representative $\eta$ and $p_{\mathrm {T}}^\mathrm {jet}$ regions for anti-$k_{t}$ jets with $R=0.4$ and $R=0.6$ calibrated with the EM+JES and LCW+JES schemes.

The total in situ calibration uncertainty (labelled “baseline in situ JES”) together with the additional uncertainties that depend on the event sample used in the physics analysis is shown in Figs. 61, 62, 63 64 for two illustrative samples. The procedure to estimate those uncertainties^19^ is detailed in Sect. 18.

Figures 61 and 62 show the flavour response uncertainty and the flavour composition uncertainties for light jets in an event sample with top-quark pairs decaying semileptonically. Semileptonic decays are selected in the MC simulation samples based on truth information, and electrons are not considered as jets when estimating the jet response. The MC generator used to evaluate the sample response and the gluon fraction is MC@NLO, while the gluon fraction uncertainty is derived using the difference in gluon fractions between the ACERMC and POWHEG generators. The average gluon fraction uncertainty ranges from 2 to 10 % depending on the jet transverse momentum and pseudorapidity. For differential measurements, the gluon fraction and its uncertainty can also be determined as a function of the property measured (e.g. number of jets). Figure 65 shows the total uncertainty for *b*-jets in the case of jets with $R =0.4$ calibrated using the EM+JES and LCW+JES schemes.

Figures 63 and 64 show the flavour uncertainties for an event sample of inclusive jets. The sample response and gluon fraction are evaluated using the Pythia nominal sample, while the gluon fraction uncertainty is derived considering the average difference in the fraction of gluons between the Pythia nominal sample and samples producing using the POWHEG (interfaced with Pythia for parton showering and hadronisation) and the Herwig++ generators. The gluon fraction uncertainty in the inclusive jet case is up to 7 % but decreases rapidly with jet $p_{\mathrm {T}}$ to less than 2 %.

A conservative topology uncertainty due to close-by jets is shown assuming the presence of a close-by jet with $R_\mathrm{min}{} =0.7$. The pile-up uncertainties are given for the average conditions of $N_{\mathrm{PV}}= 10$ and $\mu = 8.5$ in the 2011 dataset, with an RMS of 3 for both $N_{\mathrm{PV}}$ and $\mu$.

The total uncertainty is calculated by adding all uncertainty sources in quadrature. The uncertainty for jets calibrated with the LCW+JES scheme is significantly smaller than the one for the EM+JES scheme, mainly because this scheme reduces the sensitivity to the jet flavour.

## Conclusions

The ATLAS jet energy scale (JES) and its systematic uncertainty are determined for jets produced in proton–proton collisions with a centre-of-mass energy of $\sqrt{s}=7$ TeV using the full 2011 dataset that corresponds to an integrated luminosity of $4.7$ $\,\,\text{ fb }^{-1}$. Jets are reconstructed from clusters of calorimeter cells with the anti-$k_{t}$ algorithm with distance parameters $R=0.4$ or $R=0.6$. The uncertainty of the jet energy measurement is evaluated for jets with calibrated transverse momenta $p_{\mathrm {T}}^\mathrm {jet}> 20$ GeV and pseudorapidities $|\eta |<{4.5}$ using a combination of in situ techniques exploiting the transverse momentum balance between a jet and a reference object.

For central jets ($|\eta |<{1.2}$) with ${20} \le p_{\mathrm {T}}^\mathrm {jet}<{800}\, ~\mathrm{GeV }$, photons or $Z$ bosons are used as reference objects. A system of low-$p_{\mathrm {T}}$ jets is used to extend the JES validation up to the  TeV regime. The smallest JES uncertainty of less than 1 % is found for jets with ${55} \le p_{\mathrm {T}}^\mathrm {jet}<{500}\, ~\mathrm{GeV }$. For jets with $p_{\mathrm {T}}{} = 20$ GeV the uncertainty is about 3 %. For $p_{\mathrm {T}}^\mathrm {jet}> 1$ TeV the JES uncertainty is estimated from single-hadron response measurements in situ and in beam tests and is about 3 %. The JES uncertainty for forward jets is derived from dijet $p_{\mathrm {T}}$ balance measurements. The resulting uncertainty is largest for low-$p_{\mathrm {T}}$ jets at $|\eta |=4.5$ and amounts to 6 %.

From the uncertainties of the in situ techniques used to assess the JES uncertainty, the correlation of the uncertainties in $p_{\mathrm {T}}^\mathrm {jet}$ and $\eta$ are derived and made available for physics analysis as a set of systematic uncertainty sources.

**Fig. 66:**
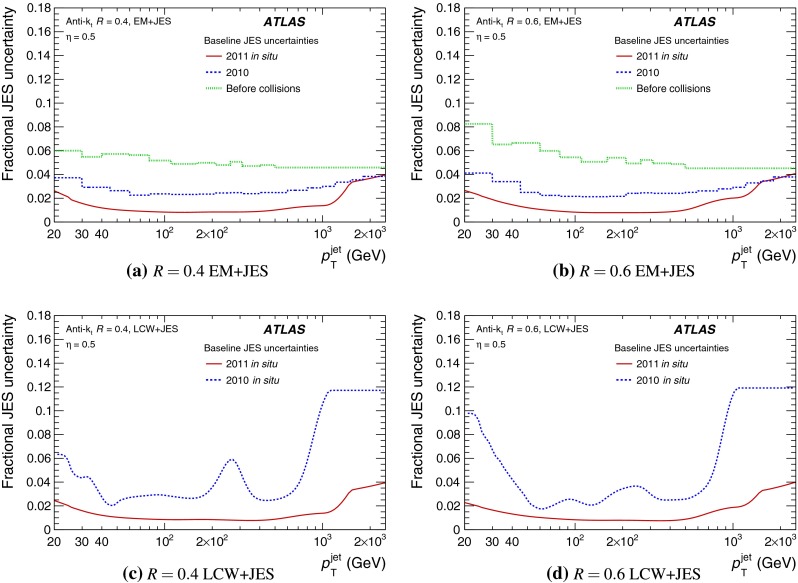
Fractional jet energy scale systematic uncertainty for inclusive jets as a function of $p_{\mathrm {T}}^\mathrm {jet}$ for jets with **a**, **c** $R=0.4$ and **b**, **d** $R=0.6$ calibrated with the **a**, **b** EM+JES and **c**, **d** LCW+JES schemes and with $\eta = 0.5$

The effect of multiple proton–proton interactions is corrected for as a function of the measured and the expected numbers of pile-up events, and an uncertainty is evaluated using in situ techniques. Additional JES uncertainties due to specific event topologies, such as close-by jets or selections of event samples with an enhanced content of jets originating from light quarks or gluons, are also discussed. These uncertainties depend on the event sample used in a given physics analysis and are evaluated for representative examples. For an event sample of semileptonically decaying top-pairs, assuming average 2011 pile-up conditions, the total JES uncertainty accounting for all effects is below 3 % for ${60} \le p_{\mathrm {T}}^\mathrm {jet}<{1000}\, ~\mathrm{GeV }$ when using the EM+JES calibration scheme, and it is further reduced to below 2.5 % if using the more refined LCW+JES calibration scheme. In the case of a sample of inclusive QCD jets under the same conditions, the total JES uncertainties for the EM+JES and LCW+JES calibration schemes are below 3.5 and 2 %, respectively.

## Appendix A: Comparison of the ATLAS JES uncertainty with previous calibrations

The progress of the JES uncertainty is demonstrated in Fig. 66. The label “2011 in situ” refers to the uncertainty documented in this paper, the uncertainty estimate on the 2010 data-set is detailed in Ref. [3] while the uncertainty determined before LHC collisions is described in Ref. [101]. The label “2010 in situ” refers to the uncertainty derived from in situ techniques in the 2010 data-set that is discussed as cross-check to the uncertainty derived from the single-hadron response in Ref. [3].

## Footnotes

1 ATLAS uses a right-handed coordinate system with its origin at the nominal interaction point (IP) in the centre of the detector and the $z$-axis along the beam pipe. The $x$-axis points from the IP to the centre of the LHC ring, and the $y$-axis points upward. Cylindrical coordinates $(r,\phi )$ are used in the transverse plane, $\phi$ being the azimuthal angle around the beam pipe. The pseudorapidity is defined in terms of the polar angle $\theta$ as $\eta =-\ln \tan (\theta /2)$.

2 The shaped pulse has a duration exceeding the charge collection time.

3 See the discussion of “truth jets” in Sect. 5.5 for the definition of stable particles.

4 Muons can generate an observable signal in some of the ATLAS calorimeters, but it is generally small and usually not proportional to the actual muon energy loss. Their contribution to the truth-jet energy, which can be large, is excluded to avoid biases and tails in the response function due to occasionally occurring high-$p_{\mathrm {T}}$ muons in the MC-simulated calibration samples.

5 The particular choice for a working point, here ($N_{\mathrm{PV}}^\mathrm{ref}= 4.9, \mu ^{\mathrm {ref}}= 5.4$), is arbitrary and bears no consequence for the correction method and its uncertainty.

6 The relative jet response is measured independently for each $\eta _\mathrm{det}$ hemisphere of the detector to accommodate asymmetries introduced by the actual collision vertex position during data taking.

7 This term prevents the minimisation from choosing the trivial solution, which is all $c_{ik} = 0$.

8 A width of $\sigma _{\log p_{\mathrm {T}}} = 0.25$ is used for the $p_{\mathrm {T}}$ interpolation and $\sigma _\eta = 0.18$ for the $\eta _\mathrm{det}$ interpolation.

9 This continuous Poisson function is obtained by extending the discrete Poisson distribution to real values by replacing the factorials in the discrete Poisson function with Euler’s Gamma function. This function has only one free parameter ($\lambda$). A linear transformation of the $x$-scale ($x' = a*x$) is introduced and the mean and width of this function are expressed in terms of $\lambda$ and $a$.

10 Any dependence of the MPF response on the jet reconstruction algorithm is introduced solely by the event selection.

11 For MPF, a less strict criterion can be used, since this technique is less sensitive to soft radiation.

12 As for all $p_{\mathrm {T}}$ balance evaluations between a reference and a probe object, the expectation value of this balance is not unity, due to physics effects (e.g., ISR) and jet reconstruction inefficiencies (e.g., out-of-cone energy losses). The ability of the MC simulation to reproduce all of these effects is further discussed in the context of the evaluation of the systematic uncertainties in Sect. 13.3.

13 Since $\langle p_{\mathrm {T}}^\mathrm {jet}/p_{\mathrm {T}}^{\mathrm {ref}}\rangle$ is close to unity for all $p_{\mathrm {T}}^{\mathrm {ref}}$ bins, the bin barycentre $\langle p_{\mathrm {T}}^{\mathrm {ref}}\rangle$ is a good approximation of $\langle p_{\mathrm {T}}^\mathrm {jet}\rangle$. In the following $p_{\mathrm {T}}^\mathrm {jet}$ is used.

14 This uncertainty is very small, and the corresponding variations are not significant, even when the evaluation is performed on the full $p_{\mathrm {T}}$ range.

15 The uncertainties apply to the overall result of the combination of the in situ techniques and differ from the original uncertainties of the in situ methods, as they are convoluted with the corresponding weights.

16 A single systematic uncertainty source is assigned to account for both the photon and electron energy scales by first adding the photon and electron scales linearly, deriving the full covariance matrix, and add it linearly to the covariance matrix of the other uncertainty components.

17 The MC simulation was updated from the version used for the combined test-beam studies to the version used for the collision data simulation.

18 Unless otherwise stated, both calorimeter jets are used in the jet response measurement if each of them can be matched to a track jet.

19 If no information on the fraction of gluons or its uncertainty is available for a given analysis sample, a gluon fraction of 50 % with 50 % uncertainty is used, representing an unknown flavour composition for the sample.
